# Checkpoint independence of most DNA replication origins in fission yeast

**DOI:** 10.1186/1471-2199-8-112

**Published:** 2007-12-19

**Authors:** Katie L Mickle, Sunita Ramanathan, Adam Rosebrock, Anna Oliva, Amna Chaudari, Chulee Yompakdee, Donna Scott, Janet Leatherwood, Joel A Huberman

**Affiliations:** 1 Department of Microbiology and Molecular Genetics, SUNY at Stony Brook, Stony Brook, New York 11794-5222, USA; 2 Department of Cancer Biology, Roswell Park Cancer Institute, Buffalo, New York 14263-0001, USA; 3 Department of Microbiology, Faculty of Science, Chulalongkorn University, Phyathai Rd., Patumwan, Bangkok 10330, Thailand

## Abstract

**Background:**

In budding yeast, the replication checkpoint slows progress through S phase by inhibiting replication origin firing. In mammals, the replication checkpoint inhibits both origin firing and replication fork movement. To find out which strategy is employed in the fission yeast, *Schizosaccharomyces pombe*, we used microarrays to investigate the use of origins by wild-type and checkpoint-mutant strains in the presence of hydroxyurea (HU), which limits the pool of deoxyribonucleoside triphosphates (dNTPs) and activates the replication checkpoint. The checkpoint-mutant cells carried deletions either of *rad3 *(which encodes the fission yeast homologue of ATR) or *cds1 *(which encodes the fission yeast homologue of Chk2).

**Results:**

Our microarray results proved to be largely consistent with those independently obtained and recently published by three other laboratories. However, we were able to reconcile differences between the previous studies regarding the extent to which fission yeast replication origins are affected by the replication checkpoint. We found (consistent with the three previous studies after appropriate interpretation) that, in surprising contrast to budding yeast, most fission yeast origins, including both early- and late-firing origins, are not significantly affected by checkpoint mutations during replication in the presence of HU. A few origins (~3%) behaved like those in budding yeast: they replicated earlier in the checkpoint mutants than in wild type. These were located primarily in the heterochromatic subtelomeric regions of chromosomes 1 and 2. Indeed, the subtelomeric regions defined by the strongest checkpoint restraint correspond precisely to previously mapped subtelomeric heterochromatin. This observation implies that subtelomeric heterochromatin in fission yeast differs from heterochromatin at centromeres, in the mating type region, and in ribosomal DNA, since these regions replicated at least as efficiently in wild-type cells as in checkpoint-mutant cells.

**Conclusion:**

The fact that ~97% of fission yeast replication origins – both early and late – are not significantly affected by replication checkpoint mutations in HU-treated cells suggests that (i) most late-firing origins are restrained from firing in HU-treated cells by at least one checkpoint-independent mechanism, and (ii) checkpoint-dependent slowing of S phase in fission yeast when DNA is damaged may be accomplished primarily by the slowing of replication forks.

## Background

The first step in the initiation of DNA replication is the binding to origins of the heterohexameric Origin Recognition Complex (ORC). Subsequently ORC, in collaboration with other proteins, promotes the binding of another heterohexameric complex composed of MiniChromosome Maintenance (MCM) proteins. The combination of ORC, the MCM complex, and certain additional proteins is called a "pre-replication complex" (pre-RC). During S phase, initiation of replication is triggered by Cyclin-Dependent Kinase (CDK) and Dbf4- (or Dfp1-) Dependent Kinase (DDK) at origin-bound pre-RCs (reviewed in [1]).

In the budding yeast, *Saccharomyces cerevisiae*, replication origins consist of 100–200 bp of DNA, which include an 11-bp AT-rich ORC-binding consensus sequence (reviewed in [1]). In other studied eukaryotic organisms, origins are not so clearly defined. For example, *S. pombe *origins are larger (500–2000 bp) and have no apparent consensus sequence. However an AT-rich region is necessary for ORC binding [2-4], and asymmetric AT-rich sequence motifs (with A residues primarily in one strand and T residues primarily in the complementary strand) are present and redundantly contribute to origin function [5-10]. In contrast to the high efficiency of many *S. cerevisiae *origins, *S. pombe *origins display a wide range of efficiencies, and few, if any, are capable of firing in more than 70% of S phases [11-15]. It appears that replication origins in other eukaryotic organisms share with *S. pombe *origins the characteristics of absence of a consensus sequence and frequent inefficiency (reviewed in [16,17]). That is, a large number of sequences have the potential to function as replication origins, but only a small subset of such sequences are used by a single cell in any given cell cycle, and the subset selected is highly variable from cell to cell.

Previously, a small number of *S. pombe *replication origins was studied intensely by autonomously replicating sequence (ARS) assays, which identify the *cis*-acting DNA sequences important for origin function when the origin region is relocated to a plasmid. A few *S. pombe *replication origins were also studied by two-dimensional (2D) agarose gel electrophoretic analyses, which permit measurements of replication fork direction and firing efficiency of origins in their endogenous chromosomal locations. These investigations revealed that most of these previously studied *S. pombe *origins fire early in S phase. However, a few sequences with positive activity in ARS assays proved to be replicated in late S phase – primarily passively – by forks coming from nearby earlier-firing origins [18]. The studied late-replicating potential origins include *ars727*, *ars2-2*, and the telomere-associated sequences at the heterochromatic ends of chromosomes 1 and 2 ([18]; A. Chaudari and J. A. Huberman, unpublished). Surprisingly, most of the heterochromatic regions of the *S. pombe *genome – centromeres, the silent mating type locus, and ribosomal DNA (rDNA) – proved to replicate in early S phase. The only tested heterochromatic region that replicates in late S phase is the telomeres [18,19].

The conclusions listed in the preceding three paragraphs were based on studies of about 20 origins. To find out whether these conclusions applied to all *S. pombe *origins or just a subset, two laboratories have recently used genome-wide computer analyses. Analyzing the few known *S. pombe *origins led Segurado *et al. *[20] to conclude that origins have an unusually high A+T content. They developed an algorithm based on AT content to generate a list of 387 predicted origins (which they called "AT islands"), each of which contains an unusually high A+T content distributed over a broad region (up to 1 kb). Eighteen of twenty predicted origins that were randomly selected from their list of 387 had detectable *in vivo *initiation activity when tested by 2D gel electrophoresis, suggesting that this simple computational approach could be a surprisingly accurate predictor of origin locations in *S. pombe*. Dai *et al. *[10] noticed that the base composition and sequence properties of known origins (high AT; frequent runs of A or T residues) closely resembled those of the longer and more AT-rich intergenic regions. Indeed, when they tested all of the intergenic regions in a 68-kb stretch on chromosome 2, they found that 14 of the 26 intergenic regions exhibited detectable ARS activity. The remaining inactive intergenic regions were shorter and/or less AT-rich than the active ones. These results suggested that about half of *S. pombe *intergenic regions (about 2500) may occasionally function as origins. This number is much greater than the previous estimates of 250 [21] to 700 [22,23] origins in the genome. The large number of potential origins in the genome, combined with the fact that 10 of the 14 active ARS elements in the 68-kb studied region displayed only weak activity suggested that origin usage in single cells during single S phases may be determined stochastically [10].

Indeed, the conclusion that replication origins in *S. pombe *are generally inefficient and fire stochastically, without relationship to the firing frequency of their neighbors and without relationship to their firing in previous generations, was strengthened by the results of another genome-wide approach, DNA fiber fluorography, which directly demonstrated the low efficiencies of individual origins and the lack of coordination between them (i.e., stochastic firing) [13].

Other laboratories have employed microarrays to obtain genome-wide information about the locations and efficiencies of origins in synchronized fission yeast cells entering S phase in the presence of HU, which starves cells for dNTPs and thus slows replication fork movement. Under these conditions, the replication checkpoint is activated. For this reason, all of the microarray analyses have compared results from wild-type cells with results from cells bearing mutations that inactivate the replication checkpoint.

The replication checkpoint responds to stalled replication forks (for example, forks that stall when cells are treated with HU or with a DNA-alkylating agent such as methyl methane sulfonate, MMS). Stalled forks, in combination with other proteins, activate an upstream kinase, Rad3 in *S. pombe *or Mec1 in *S. cerevisiae*. Both are homologues of mammalian ATR. The upstream kinase activates a downstream kinase, Cds1 in *S. pombe *or Rad53 in *S. cerevisiae*. These are structural homologues of mammalian Chk2 and functional analogs of both Chk2 and Chk1. Indeed, when replication forks stall as a consequence of template DNA methylation by MMS, the replication checkpoint is activated, and progress through S phase is slowed in both budding [24,25] and fission yeast [26-29]. Impeding replication forks also leads to checkpoint-dependent replication slowing in mammalian cells [30-32].

In mammalian cells, checkpoint-dependent slowing of S phase is accomplished by a combination of checkpoint-dependent inhibition of origin firing [16,31-33] and checkpoint-dependent inhibition of replication fork movement [32,33]. However, in budding yeast the slowing of S phase in response to MMS treatment is accomplished entirely by inhibition of replication origin firing; no checkpoint-dependent fork inhibition is detectable [25]. The mechanism by which S phase is slowed in MMS-treated fission yeast cells is not clear. The evidence available before the advent of microarray studies suggested that, as in budding yeast, slowing of replication in fission yeast probably depended on inhibition of origin firing, because, when the replication checkpoint is activated by treating *S. pombe *cells with HU, the replication of two late potential origins (*ars2-2 *and telomeres) is retarded in checkpoint-dependent fashion [18]. However, results from the new microarray studies, which are reviewed below, have not been entirely consistent with each other or with these prior studies and have led to greater confusion.

In the first microarray study, Feng *et al*. [34] took advantage of the fact that replication forks unwind parental strands, generating single-stranded DNA (ssDNA). Thus, if cells enter S phase in the presence of HU, those origins capable of firing in early S phase generate small regions of single-stranded DNA (ssDNA), while regions far from early-firing origins remain double-stranded. Feng *et al. *[34] looked for regions of ssDNA in both HU-treated *S. cerevisiae *and in HU-treated *S. pombe *cells. Furthermore, they examined both wild-type cells and replication-checkpoint-mutant cells.

To study the effects of the replication checkpoint on origin firing in *S. cerevisiae*, Feng *et al. *[34] compared HU-treated wild-type with HU-treated *rad53 *mutant cells. They found that 2/3 of *S. cerevisiae *replication origins are restrained from firing by the replication checkpoint. An even more recent study, which employed copy number measurements rather than measurements of single-stranded DNA, confirmed that 2/3 or more of *S. cerevisiae *replication origins are checkpoint-restrained [35].

When Feng *et al. *[34] applied the same procedure to *S. pombe *and smoothed the results over a 12-kb window, 321 ssDNA peaks (putative origins) were identified in cells lacking the Rad53 homologue, Cds1. Of these, 125 (39%) were specific to *cds1*Δ cells (apparently checkpoint-restrained). These observations suggested, therefore, that in *S. pombe *a smaller (but still significant) proportion of origins is restrained by the replication checkpoint than in *S. cerevisiae*. In both yeasts, ssDNA accumulation was much greater in the checkpoint-deficient strains, presumably due to the roles of Cds1 and Rad53 in stabilizing replication forks (reviewed in [36]).

In the second microarray study, Heichinger *et al. *[14] measured changes in copy number at each position in the genome as a synchronized population of fission yeast cells entered S phase in the absence or presence of HU. The investigators made similar measurements for checkpoint-mutant (*rad3*Δ) cells in the presence of HU. The results of all three experiments were in good agreement with each other, and they permitted the identification of 401 relatively strong origins plus 503 putative weaker origins. Surprisingly, only about 2% of these origins appeared to be inhibited by the replication checkpoint – in contrast to the ssDNA measurements of Feng *et al. *[34], which had suggested that ~39% of fission yeast replication origins are checkpoint-restrained.

The third microarray investigation, by Hayashi *et al. *[15], provided additional important information but no clear resolution to the apparent conflict between the checkpoint-mutant results of Feng *et al. *[34] and Heichinger *et al. *[14]. Hayashi *et al*. [15] used immunoprecipitation in combination with microarrays to localize pre-RCs (binding sites for both ORC and MCM polypeptides). A total of 460 pre-RCs was found. Then microarrays were used to identify those pre-RCs where significant incorporation of the thymidine analogue, 5-bromo deoxyuridine (BrdU), took place when synchronized cells entered S phase in the presence of HU. Of the 460 pre-RCs, 307 incorporated significant BrdU and were considered to be early-firing and/or strong origins. Little or no BrdU incorporation was detected at the remaining 153 pre-RCs, which were consequently characterized as late-firing and/or weak origins. Hayashi *et al. *[15] found that there was increased incorporation of BrdU at about 22% of origins (mostly weak/late origins) in checkpoint-mutant (*cds1*Δ) cells compared to wild-type cells, and such checkpoint-mutant-induced BrdU incorporation was especially frequent in sub-telomeric regions. However, at most of these apparently checkpoint-inhibited origins, the extent of BrdU incorporation in *cds1*Δ cells was significantly less than for the early/strong class of origins and could only be detected by dividing the signal from *cds1*Δ cells (small) by the signal from wild-type cells (even smaller) [15].

Thus the three microarray studies appeared to reach rather different conclusions regarding the extent to which fission yeast replication origins are restrained by the replication checkpoint. The two studies that appeared to detect a large fraction (22% or 39%) of checkpoint-restrained origins were both based on comparisons between wild-type and *cds1*Δ cells. The single study that found only a small fraction (2%) of checkpoint-restrained origins employed *rad3*Δ cells. Each of the three studies employed a different procedure to measure extents of replication in wild-type and checkpoint-mutant cells. Could these experimental differences explain the different results obtained?

Here we present the results and conclusions of our own microarray-based measurements of DNA replication, as synchronized wild type, *cds1*Δ and *rad3*Δ cells enter S phase in the presence of HU. Our results provide an explanation for the differences between the earlier microarray studies. Our findings also lead to interesting conclusions regarding the control of replication timing, the mechanisms by which a subset of origins is restrained from firing in HU-treated cells, the mechanism by which fission yeast cells retard S phase in response to DNA damage, and the relationships between checkpoint regulation of origins and their chromatin structure.

## Results

### Retardation of DNA replication by hydroxyurea

We synchronized wild-type and checkpoint-mutant (*cds1*Δ and *rad3*Δ) cells using the *cdc25-22 *block-and-release method [37]. After release from the G2 block, these cells normally proceed synchronously through the M, G1 and S phases. In our experiments, however, we added HU (15 mM) at the time of release from the G2 block, so the cells entered S phase in the presence of HU, which dramatically slows replication forks [38-40]. We harvested cells at zero, two, and four hours after the addition of HU.

**Figure 1:**
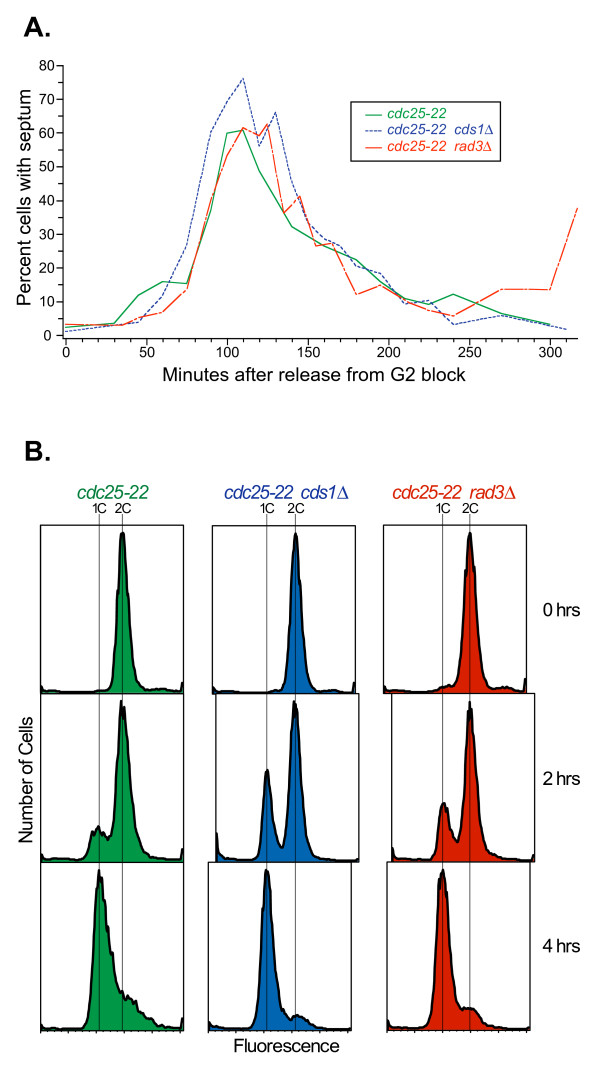
**Synchronization of fission yeast cells by release from G2 arrest into HU**. Three isogenic fission yeast strains – JLP1164 (*cdc25-22*; green), JLP1257 (*cdc25-22 cds1*Δ; blue), and JLP1260 (*cdc25-22 rad3*Δ; red) were arrested in G2 phase, then released (at time 0) into the cell cycle in the presence of 15 mM HU. (A) Measurements of septation indices at the indicated times after release. (B) Flow cytometric measurements of DNA content, using a fluorescent DNA-specific stain (Sytox Green), at the indicated times after release. See the text for interpretation.

In HU-treated cells, septum formation proceeds independently of DNA synthesis. We found that septum formation peaked at 60–80% at 100–125 minutes, indicating good synchronization (Figure 1A). We used flow cytometry to measure the amount of replication per cell during the course of our experiment (Figure 1B). At zero hours, most cells were arrested in G2 with a 2C DNA content. At two hours, a 1C peak had started to form as a consequence of cytokinesis. At four hours after HU addition, the major peak was at 1C. In the case of wild-type cells, spreading of this 1C peak toward 2C was evident. This was a consequence of some of the cells progressing into S phase to variable extents, despite the presence of HU. Such spreading was not evident in the checkpoint-mutant cells, consistent with a previous demonstration that replication proceeds more slowly in HU-treated checkpoint-mutant cells than in HU-treated wild-type cells [18].

### Genome-wide measurement of extent of replication in hydroxyurea

To determine origin usage in the presence of HU, we employed microarrays to measure the amount of replication as a change in copy number at separate locations along the fission yeast genome. Our microarray probes were constructed by PCR and corresponded to the complete set of fission yeast predicted mRNAs (4,824 probes), other predicted RNAs and intergenic regions (504 probes), and some introns (79 probes). The probes ranged in size from 100 bp to 1200 bp and were primarily directed against the 3' ends of ORFs. Importantly, the spatial positions of the probes on the microarrays were random with respect to the chromosomal locations of the corresponding genes.

We arrested the *cdc25*-22 mutant strains in G2 by incubation at restrictive temperature and then released them into HU (time 0). We isolated DNAs from 0-hr, 2-hr and 4-hr cells. DNA from 0-hr cells (which were in G2) was used as a control, because all regions of the genome have the same copy number in G2 phase. The DNAs from 2-hr or 4-hr cells were labeled with Cy3 fluorescent dye, while the control DNA was labeled with Cy5. Cy3-labeled DNAs were mixed with Cy5-labeled DNAs and simultaneously hybridized to the microarray. We determined the ratio of Cy3 to Cy5 hybridization for each probe in the microarray. These ratios were then normalized to a genome average value of 1.0. The results from multiple hybridizations were averaged together, and the average values were plotted against the position of each probe in its chromosome. Complete collections of plots at a resolution of 150 kb per plot are available online as Additional Files 1, 2 and 3, for chromosomes 1, 2 and 3, respectively. The normalized, averaged values employed in the plots are also available online as Additional Files 4, 5 and 6 (tables of data for chromosomes 1, 2 and 3, respectively). The raw microarray data have been deposited in the ArrayExpress public repository [41] under accession number E-MEXP-1127.

**Figure 2:**
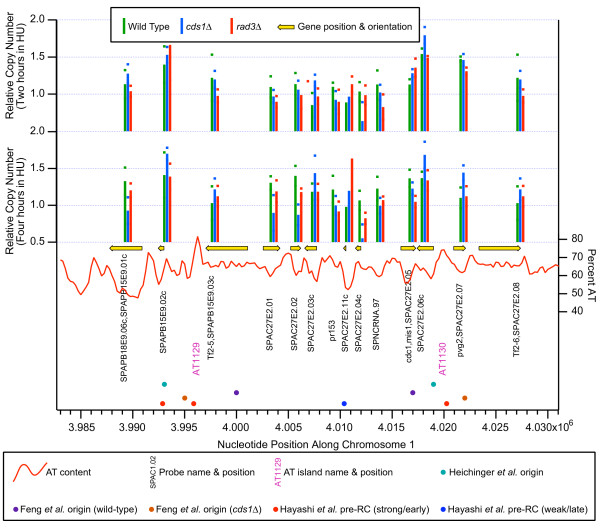
**Microarray measurements of copy number increases on a segment of DNA in the right arm of chromosome 1**. For clarity, the symbols used in this graph are explained in the keys (boxes) above and below the graph. They are also explained here. The information in this legend applies not only to Fig. 2 but also to Figs. 7 and 8, and to Additional Files 1, 2, 3 and 11, 12, 13, 14. The upper set of vertical lines shows relative copy numbers of the indicated probes 2 hrs after release from the G2 block, while the lower set of vertical lines shows results for 4-hr cells. Green lines: wild-type cells (JLP1164), plotted at their correct positions. Blue lines: *cds1*Δ (JLP1257), plotted to the right of their correct positions. Red lines: *rad3*Δ (JLP1260), plotted further to the right of their correct positions. The horizontal offsets of the blue and red lines were introduced to permit simultaneous visualization of the results for all three strains (wild-type, *cds1*Δ and *rad3*Δ). The heights of most lines represent the averages of 3–5 independent hybridizations to the same probe. Due to probe-specific experimental noise, for a few probes the number of successful hybridizations was only one or two. The average deviations of the individual hybridizations from the average value are indicated by appropriately colored small rectangles above and below the end point of each vertical line. For probes with only a single successful hybridization, no rectangle is shown. Because the probes mostly correspond to predicted gene locations, they are not uniformly spaced along the DNA. The names of the probes are indicated in black vertical type. In most cases, the names of the probes are the same as the names of the genes in which they are located. These genes are shown as thick yellow arrows above the corresponding probe names. The red, continuous graph indicates AT content in a sliding 500-bp window along the genome. Lower down, the names and locations of AT islands [20] are shown in magenta. The AT islands are numbered sequentially from left to right in each chromosome (except for a few cases where their chromosomal locations have been altered due to improved sequencing results since the original assignment of AT island names [20]). A "+" after the AT island name (not present in Fig. 2, but present at some locations in Figs. 7 and 9 and Additional File 13) indicates that a restriction fragment containing the AT island has been tested by 2D gel electrophoresis and found to have origin activity. If the origin associated with an AT island was previously studied and assigned a name, we show that name immediately after the "+". Conversely, a "-" after the AT island name (not present in Fig. 2, but present at a subset of locations in Additional Files 1, 2, 3 and 11) indicates that a restriction fragment containing the AT island failed to show origin activity by 2D gel electrophoresis. Below the AT island names are light blue circles, which indicate each of the 401 locations identified as "stronger origins" by Heichinger *et al. *[14]. Further down are purple and orange circles, which show the positions identified as origins by Feng *et al. *[34] using the ssDNA method, in wild-type or *cds1*Δ cells, respectively. At the bottom of the graph are red and dark blue symbols, which are circles in the case of Fig. 2 but could also be squares (as in Fig. 8A, C). The red circles/squares indicate the positions of pre-RCs which were found to be active by BrdU incorporation and were classified as strong/early origins [15] The blue circles/squares indicate pre-RCs which did not score as active by BrdU incorporation and were classified as late/weak origins [15]. Circles indicate pre-RCs whose signal strength was the same in wild-type and *cds1*Δ cells, while squares indicate pre-RCs whose signal strength was higher in *cds1*Δ cells than in wild-type cells. The latter were considered to indicate checkpoint-restrained origins [15].

A sample plot, from the right arm of chromosome 1, is shown in Fig. 2 at higher resolution (45 kb from the left to the right end of the plot). The symbols are explained in the keys above and below the figure and in the figure legend. Because the probes mostly correspond to sequences within genes, they are not uniformly spaced along the DNA. In Fig. 2 and elsewhere we present unsmoothed results to prevent possible information loss, and also because occasional large intergenic gaps between probes (several such gaps are visible in Fig. 2) would make smoothing misleading.

In Fig. 2, a relative copy number of 1.0 indicates an average amount of replication at that time point. Because all experiments were carried out with cells entering S phase in the presence of HU, the extent of replication of the total genome at each time point is, in most cases, very small (Fig. 1B). Therefore, probes located at or very close to origins that fired in 100% of cells in the presence of HU should have relative copy numbers close to 2.0. In contrast, probes that didn't replicate at all under these conditions should have relative copy numbers close to 0.5. Since both the 2-hour and 4-hour data sets were normalized to 1.0, those probe sequences that replicated more than average between 2 hours and 4 hours increased in value between 2 and 4 hours; conversely, the probe sequences that replicated less than average decreased in value between 2 and 4 hours. This behavior is a mathematical consequence of our normalization procedure and does not indicate that DNA was lost from any region of the genome between 2 and 4 hours.

As is evident in Fig. 2, in most cases neighboring probes generated similar copy number values. Furthermore, the values for the wild-type, *cds1*Δ and *rad3*Δ strains were also frequently similar. However, in a few cases single probes in single strains generated values out of line with their neighbors. We suspect that these unusual values reflect experimental noise, but it is also possible that some of the apparently anomalous values may be significant. Additional investigations are needed to distinguish between these possibilities.

The results shown in Fig. 2 demonstrate the generally good agreement between the published genome-wide studies. Hayashi *et al. *[15] mapped pre-RCs using a tiled, high-resolution (250-bp) microarray. In contrast, the other microarray analyses, including ours, employed lower-resolution microarrays with probes located at ORFs. In the case of Heichinger *et al. *[14], probes were also located in intergenic regions. Consequently, the pre-RC locations determined by Hayashi *et al. *[15] are probably more accurate than the origin locations determined in the other studies, especially since the other studies (except ours) used peak-detection algorithms that employed sliding windows analyzing smoothed data. For these reasons, we interpret the new results and findings from previous studies shown in Fig. 2 to indicate that all studies are in agreement that (i) there are one or two replication origins, active in HU, located at or near AT1129, and (ii) there is another HU-active origin located at or near AT1130. In addition, our data suggest the possible presence of a weaker origin that fires later in wild-type cells (signals stronger at four hours than two hours) at approximately 4.005 Mbp, close to probe SPAC27E2.02. Hayashi *et al. *[15] identified a pre-RC close to 4.010 Mb (dark blue dot), but did not detect significant BrdU incorporation at this position in the presence of HU (which is why the circle is dark blue rather than red; Fig. 2 legend), consistent with the other microarray studies, including ours, which also did not detect much replication at this position.

### Extensive correspondence between predicted origins identified by five genome-wide approaches

We used our data to evaluate the extent of replication in HU, under our experimental conditions, of origins predicted by the four previous genome-wide analyses – the AT islands defined by Segurado *et al. *[20], the putative origins identified by Feng *et al. *[34] and Heichinger *et al. *[14], and the pre-RCs found by Hayashi *et al. *[15]. We also identified 22 additional putative origins (regions of clear replication activity according to our data) that were not detected in the previous studies. We compared the locations of AT islands, putative origins and pre-RCs with our HU copy number results (as in Fig. 2), and we classified them into six categories – strong, medium, weak, very weak, below detection limit, and ambiguous – according to the criteria detailed in Methods. Briefly, these criteria were based on the fact that our probes were specific for ORFs, but origins are located in the intergenic regions between ORFs. Consequently, each putative origin was flanked by two probes (or more, in cases where the location of the putative origin was poorly defined). For each probe, there were two time points (two hours and four hours) and three cell strains. For an origin to be classed as "strong", we required that either the wild-type signal or the signals for both checkpoint-mutant strains had to be greater than 1.5 at one or more of the four possible position/time combinations (left probe, 2 hours; left probe, 4 hours; right probe, 2 hours; right probe, 4 hours). For an origin to be classed as "very weak", the signal for the wild-type and/or both checkpoint-mutant strains needed to exceed 1.1 at one or more position/time combination(s). Our definitions of "weak" and "medium" origins are intermediate between those of "very weak" and "strong" (see Methods for details).

The results of our comparisons for the full lengths of all three chromosomes are shown in tabular form in Additional File 7. As an example of the information provided by Additional File 7, in Table 1 we show a small subset of that information – just our classifications of the putative origins within the region on chromosome 1 that is pictured in Figure 2. We encourage readers to examine the complete data for all three chromosomes in Additional File 7.

**Table 1:** The data from Additional File 7 applicable to Figure 2.

**Start Position**	**End Position**	**Start Gene**	**End Gene**	**AT Number**	**AT Function**	**Feng et al. WT Function**	**Feng et al. cds1 Function**	**Heichinger et al. Number**	**Heichinger et al. Efficiency**	**Hayashi et al. PreRC Number**	**Hayashi et al., Activity**	**Mickle et al. Function**
3989260	3993040	SPAPB15E9.01c	SPAPB15E9.02c					Ori1129	59	1148	1	strong
3993040	3997640	SPAPB15E9.02c	SPAPB15E9.03c	AT1129	strong	strong	strong			1149	1	strong
4003300	4005680	SPAC27E2.01	SPAC27E2.02									medium
4007292	4010577	SPAC27E2.03c	SPAC27E2.11c							1150	0	weak
4017870	4021600	SPAC27E2.06c	pvg2	AT1130	strong	strong	strong	Ori1130	31	1151	1	strong

This table displays a small subset (only the information relevant to Fig. 2) of the information contained in Additional File 7. For each of the putative origins identified and evaluated in Fig. 2, the first two columns of Table 1 show the start (left side) and end (right side) of the positions of the PCR probes located in the genes flanking the origin. The next two columns show the names of those two genes (which are also shown in Fig. 2). Where the origin being evaluated is an AT island, the next two columns show the name of the AT island (according to [20]) and its functional classification by our criteria (see Methods). The following two columns show the classification of origins identified by Feng *et al. *in wild-type or in *cds1*Δ cells. For origins identified by Heichinger *et al.*, the following two columns show the Ori number assigned to the origin and the efficiency of the origin during a mitotic S phase as evaluated by Heichinger *et al. *[14]. For pre-RCs identified by Hayashi *et al. *[15], the next two columns show the number of the pre-RC and whether the pre-RC was scored by Hayashi *et al. *as strong/early (1) or weak/late (0). The final column shows our (Mickle *et al.*) classification of the putative origin.

Additional File 8 is a table summarizing the classifications of origin strengths (from Additional File 7) by chromosome and by type of origin. One of the interesting results in this table is that at least 83% of every type of origin was classified as functional ("very weak", "weak", "medium", or "strong") under our experimental conditions, implying extensive agreement between our results and those of the other studies [20,34,14,15].

**Figure 3:**
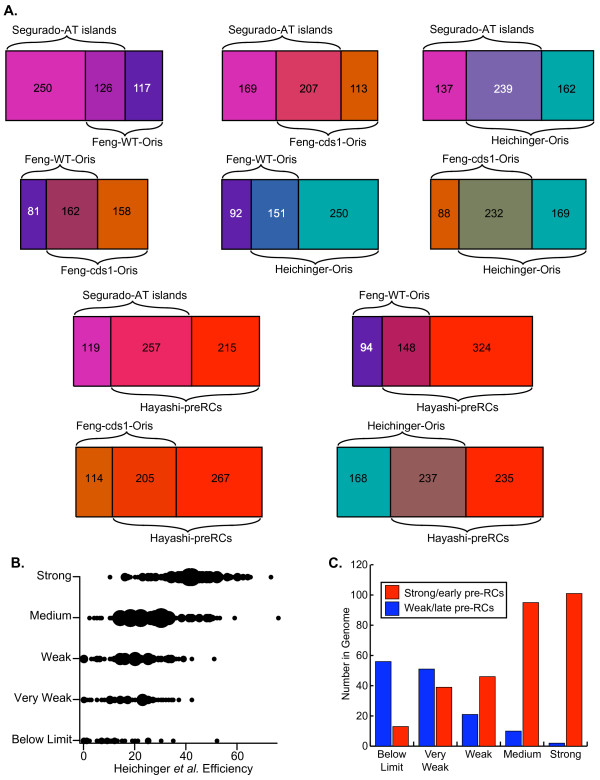
**Correlations between our results and published studies of fission yeast replication origins**. (A) Rectangular Venn diagrams showing the extents to which previously published locations of potential origins correlate with each other. The number in each box is the number of potential origins in the category represented by that box. The area of each box is proportional to the number in the box. The colors in the left- and right-hand boxes are coded in the following way: magenta represents AT islands, purple represents the origins identified by Feng *et al. *[34] in wild-type cells, orange represents the origins identified by Feng *et al. *in *cds1*Δ cells, light blue indicates origins identified by Heichinger *et al. *[14], and red indicates pre-RCs identified by Hayashi *et al. *[15]. The colors in the middle boxes are intended to be blends of the colors in the left- and right-hand boxes, and they are intended to indicate that the middle box in each diagram represents the set of potential origins that was identified in common by the two studies represented by the left- and right-hand boxes. The left- and right-hand boxes, in contrast, show the numbers of potential origins in the indicated studies that did *not *co-localize with each other. (B) Correlation between the origin efficiencies determined by Heichinger *et al. *[14] and us. As described in the text, the signal strength that we determined at the position of each of the origins identified by Heichinger *et al. *was assigned to one of five categories (ranging from below limit to strong; vertical axis) and plotted as a small black circle against the mitotic efficiency of that origin measured by Heichinger *et al. *(horizontal axis). In cases where more than one origin had identical efficiencies as scored by us and by Heichinger *et al.*, the size of the circle was proportionally enlarged. (C) Correlation between the strong/early or weak/late classifications of pre-RCs by Hayashi *et al. *[15] and our classifications of origin strength. The numbers of weak/late pre-RCs from the study of Hayashi *et al. *that fell into each of our classifications are plotted as blue bars against the classifications. The strong/early pre-RCs are similarly plotted as red bars.

Additional File 9 summarizes the extents of overlap between the origin locations predicted by the other studies [20,34,14,15]. Figure 3 displays some of the combined results for all evaluated origin positions from Additional Files 7, 8, 9 in graphic form. The rectangular Venn diagrams in Fig. 3A show – in pair-wise combinations – the extents to which the origins predicted by the other studies co-localize with each other. The proportion of co-localization (73%) proved to be highest for the 320 origins identified by Feng *et al. *[34] in *cds1*Δ cells, when compared with the 401 predicted by Heichinger *et al. *[14]. In other cases, the proportion of co-localization was less (Fig. 3A).

Since all of the previously predicted origins co-localize by 83% or greater with chromosomal positions identified as functional in our studies (Additional File 8), we conclude that the 655 putative origins that we identified as functional under our conditions (Additional Files 7 and 8) represent a more complete picture of mapped fission yeast replication origin locations than obtained from any single one of the previous studies. Note, however, that Heichinger *et al*. [14] detected 503 weaker origins in addition to their 401 stronger origins. We were not able to compare our results with their 503 weaker origins, because the coordinates of their weaker origins were not provided in their publication [14]. It is likely, however, that our 655 functional origins are mostly a subset of their 904 (401 + 503) origins.

We have also compared our classification of origin efficiencies (from very weak to strong) with the quantitative measurements of origin efficiency reported by Heichinger *et al. *[14]. For each of their 401 published origins [14], we plotted (Fig. 3B) the strength of the origin according to our rough classification (Additional File 7) against the efficiency of the origin during mitotic S phases as determined by Heichinger *et al. *([14]; these data are also listed in Additional File 7). In many cases, two or more origins had the same classification in our results and the same efficiencies according to Heichinger *et al. *[14]. In these cases, the black circle representing the data point in Fig. 3B was enlarged in proportion to the number of origins having the indicated characteristics. The results in Fig. 3B indicate a fairly good correlation between our rough efficiency classifications and the efficiency measurements of Heichinger *et al. *[14].

On the basis of extent of incorporation of BrdU in the presence of HU, Hayashi *et al. *[15] divided the pre-RCs that they identified into two classes: strong/early (identified by red circles and squares in our Figs. 2 and 6) and weak/late (identified by dark blue circles and squares in our Figs. 2, 6 and 8). As shown in Fig. 3C (based on results in Additional File 7), these two classes corresponded well with our five classes. The origins that we classified as below limit or very weak were mostly weak/late according to Hayashi *et al.*, while the origins that we classified as medium or strong were almost entirely strong/early by their classification [15].

**Figure 4:**
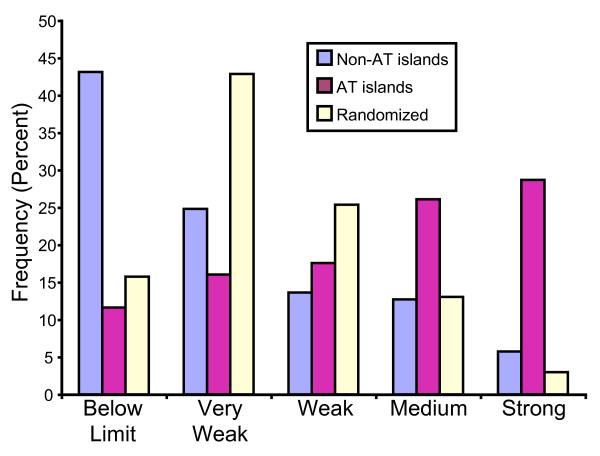
**AT islands correlate with strong potential origin activity**. The signals from the probes flanking the 387 AT island positions were evaluated and assigned to one of the five categories (below limit to strong; horizontal axis), and the percentage of AT islands in each of the five categories was determined (magenta bars; vertical axis). Similarly, the percentages of signals in each category from the 4636 pairs of adjacent probes that did *not *flank AT islands were also plotted (blue bars). To obtain the randomized results (cream-colored bars), the six datasets (wild-type, *cds1*Δ, and *rad3*Δ at 2 and 4 hours) were randomized for position within each dataset, and the scores for all positions were re-calculated. This was repeated ~1000 times, and the resulting average percentages are shown.

The results in Fig. 4 show that there is also a good correlation between the AT islands identified by Segurado *et al. *[20] and our classifications. AT islands tend to be stronger rather than weaker origins. The behavior of AT islands is clearly different from that of all the non-AT-island positions in the genome, which most frequently score as below limit or very weak. The different behaviors of AT islands and non-AT-island positions are not the consequence of a random distribution of copy number signals of varying strengths along the genome, because when our copy number signals are randomized with respect to position, distributions like the one illustrated by the cream-colored bars in Fig. 4 are obtained. The cream-colored distribution is, in fact, the average of approximately 1000 different randomizations of the copy number signal data with respect to position.

**Figure 5:**
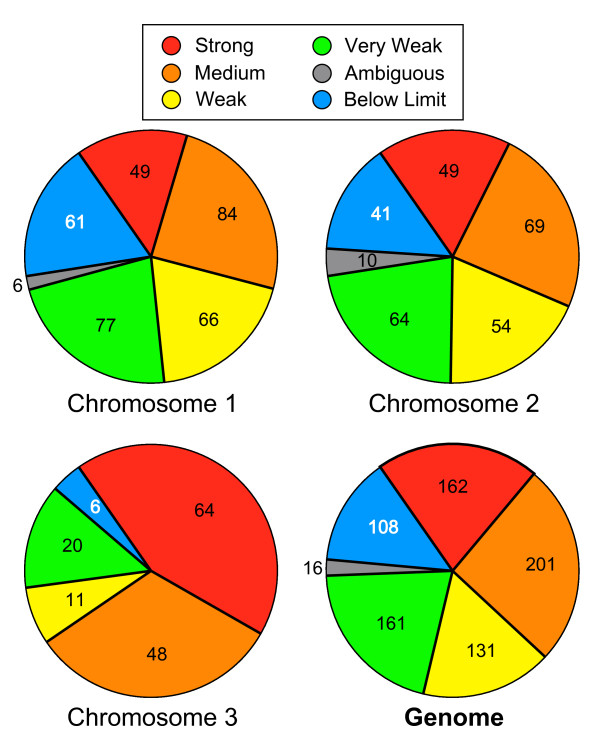
**Distributions of origin efficiencies on the three chromosomes and in the genome**. These pie charts show the distributions of predicted origins with the indicated efficiencies on the three fission yeast chromosomes and also in the whole genome. The whole genome scores are the sums of the scores for the three chromosomes.

Fig. 5, which is based on data in Additional Files 7 and 8, reveals that, although the proportions of strong, medium, weak and very weak origins are similar in chromosomes 1 and 2, origins in chromosome 3 are more likely to be strong or medium and less likely to be weak, very weak or below-limit. This difference between chromosomes may be related to the fact that chromosome 3 is smaller than the other two chromosomes.

**Figure 6:**
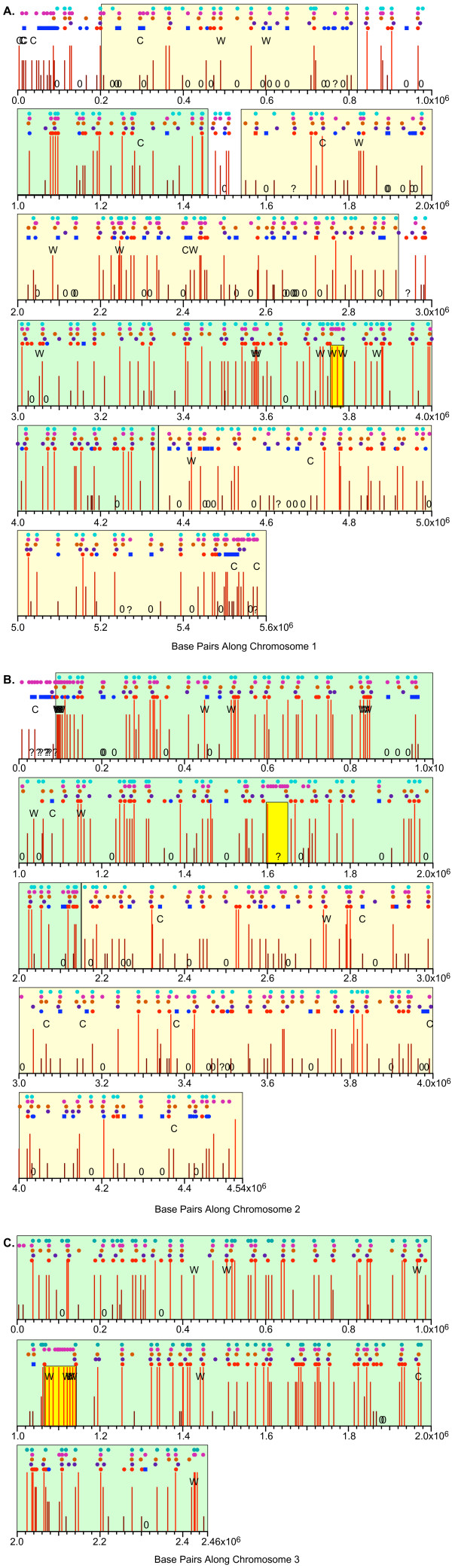
**Locations and efficiencies of putative origins on the three chromosomes**. The chromosomes are shown as consecutive horizontal lines, 1 Mbp per line. The position of the centromere on each chromosome is indicated by a light yellow rectangle. The positions of origins classified as strong, medium, weak or very weak are identified by vertical lines. The lines range in color from red (strong) to brown (very weak) and from long (strong) to short (very weak). The positions of potential origins below the detection limit are indicated by the text character, "0", and ambiguous origins (where our probes were too widely spaced to permit confident evaluation) are shown by the character, "?". Small circles above each chromosome line indicate the positions of origins identified by Heichinger *et al. *([14]; top row of circles; light blue), AT islands (next row of circles; magenta), origins identified by Feng *et al. *[34] in *cds1*Δ cells (next row; orange) or in wild-type cells (next row; purple), and pre-RCs identified by Hayashi *et al. *([15]; bottom row; dark blue or red circles or squares). For the pre-RCs, the colors blue and red distinguish the pre-RCs that are late/weak or early/strong, respectively. The circles represent pre-RCs that are not affected by deletion of *cds1*, while the squares indicate pre-RCs that replicate to a greater extent in *cds1*Δ cells than in wild-type cells [15]. The positions of origins where the signals (our measurements; Additional Files 4, 5, 6) for both checkpoint-mutant strains were significantly greater than the signal for wild-type cells are indicated by the text character, "C", and the positions of origins with the opposite characteristic (wild-type signal significantly greater than the signals from both checkpoint-mutant strains) are shown by the text character, "W". A pale green background indicates a large region with a high frequency of stronger origins. A pale yellow background indicates a large region with a high frequency of weaker origins. (A) chromosome 1; (B) chromosome 2; (C) chromosome 3.

To test whether the two larger chromosomes contain regions that are as rich in strong origins as chromosome 3, we displayed our origin classifications (Additional File 7) according to the position of each origin within its chromosome. Fig. 6 shows that the results from previous studies (light blue circles, Heichinger *et al. *[14]; magenta circles, Segurado *et al. *[20]; orange circles, the *cds1*Δ strain of Feng *et al. *[34]; purple circles, the wild-type strain of Feng *et al. *[34], and dark blue and bright red circles or squares, the pre-RCs identified by Hayashi *et al. *[15]) frequently resemble each other (as indicated by lining up vertically) and frequently correspond to regions that replicated in HU in our studies (vertical lines). It is also evident that the frequencies of regions significantly replicated in HU vary along the genome. Consistent with the pie graph in Fig. 5, chromosome 3 (Fig. 6C) contains high proportions of strong and medium origins (taller, redder lines). Regions with similarly high proportions of strong and medium origins are also evident in chromosomes 1 and 2 (indicated by pale green background). Unlike chromosome 3, however, the larger chromosomes also contain regions with reduced frequencies of strong and medium origins (indicated by pale yellow background). Note that the difference between chromosome 3 and the other chromosomes extends across the whole of chromosome 3 and does not simply reflect differences between subtelomeric regions at the ends of chromosomes 1 and 2 and the rDNA-adjacent regions at the ends of chromosome 3. The regional variations evident in Fig. 6 are similar in position and extent to the regional variations noted by Heichinger *et al. *[14] and by Hayashi *et al. *[15].

### Extensive correspondence between origins detected by microarrays and previous 2D gel and molecular combing studies of origin function

In Additional File 10 we list the 54 regions of which we are aware that have previously been tested by 2D gel electrophoresis for origin activity in a chromosomal context. 51 of these regions are in positions where our probe density is sufficient to permit evaluation of potential origin function. Of these, 43 regions appeared to be functional (though usually weak) origins by 2D gel electrophoresis, because they displayed bubble arcs as well as Y arcs. The same 43 regions were classified as potentially functional origins (relative copy number > 1.1) according to our microarray data. In three other cases (AT1022, *ars2-2*, and telomere-associated sequences) the opposite occurred – the potential origin was non-functional according to our results, and it was also non-functional by 2D gel electrophoresis (no detectable bubble arc). Thus in 46 cases out of 51 (90%), there was good correspondence between our results and 2D gel tests of origin function.

**Figure 7:**
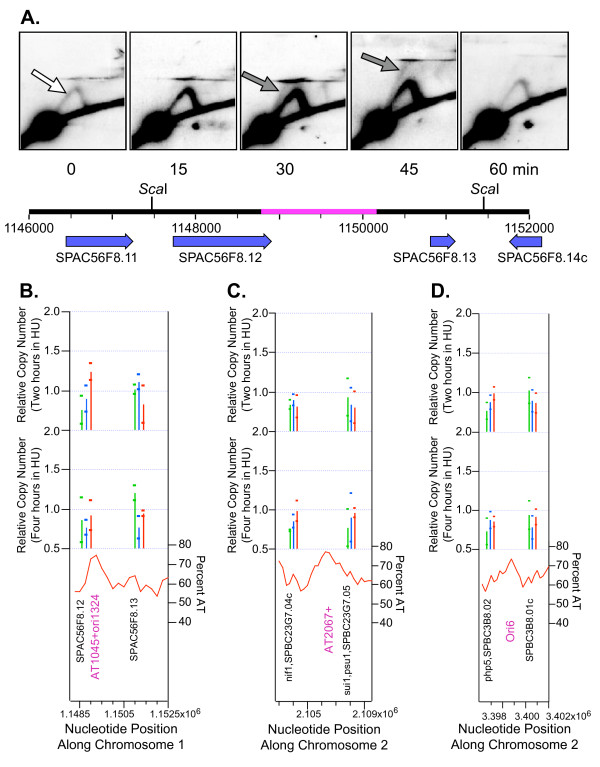
**Origins that do not replicate in HU may normally replicate in late S phase**. (A) 2D gel analysis revealed that a *Sca*I restriction fragment containing AT1045 near its center replicated only slightly in HU, producing a faint Y arc (white arrow; 0 minutes), but replicated more extensively after HU was removed (15–60 minutes), with a weak bubble arc indicative of origin activity evident at 30 and 45 minutes (dark arrows). The diagram underneath the 2D gel pictures illustrates a 6-kb segment of genomic DNA containing the studied *Sca*I restriction fragment. The scale indicates the nucleotide position along chromosome 1. Genes, along with their names and directions of transcription, are indicated below the scale. The magenta stretch in the middle of the *Sca*I restriction fragment indicates the central third of that fragment, within which an origin – if present – is likely to be capable of generating detectable bubble arcs [58]. (B) Microarray results for the two probes flanking AT1045. The wild-type (green) signal for the right-hand probe exceeded the threshold of 1.1 at 4 hours, leading to the classification of "very weak". (C) A restriction fragment containing AT2067 was previously tested and shown to have origin activity [20]. However, our microarray results (all signals below 1.0) suggest that AT2067 does not replicate in HU. AT2067 may replicate in late S phase (only after HU removal), as in the case of AT1045. (D) Microarray measurements for the two probes flanking Ori6. DD Dubey, VK Srivastava, AS Pratihar and MP Yadava (submitted for publication; personal communication) used 2D gel electrophoresis to carefully study a 75-kb stretch of chromosome 2. They found six origins (Ori1-Ori6) capable of generating bubble arcs (summarized in Additional File 10). In HU synchronization experiments, they found that Ori6 replicated only after release from HU. This is consistent with our microarray measurements, which show little or no replication of this region in the continued presence of HU. In panels (B)-(D) the small symbols indicating the origins detected experimentally by other laboratories [34] [14] [15] are not shown.

Two of the apparent exceptions are AT2067 (Fig. 7C) and Ori6 (Fig. 7D), both on chromosome 2 (see Additional File 10 for references). These did not generate copy numbers > 1.1 in our experiments but did display bubble arcs in 2D gel analyses. The origin at AT1045 (Fig. 7B) behaved similarly. It replicated only very weakly (Additional File 10 and Fig. 7B) but nevertheless displayed a readily detectable bubble arc in 2D gel analyses (Fig. 7A). For the experiment in Fig. 7A, we employed an HU-block-and-release synchronization protocol. We treated log-phase wild-type (checkpoint-competent) cells with HU for 3 hours. The HU treatment caused replication forks to stall, and most cells were arrested in very early S phase, with replication forks stalled a short distance away from early-firing origins. Then, at the 0-minute time point, we removed HU, and replication forks were able to resume moving. At the 0-minute time point, only a few cells in the population contained replication forks within the *Sca*I fragment that encompasses AT1045 (see the diagram under the 2D gel panels in Fig. 7A). These forks came from neighboring origins, and they generated Y-shaped replication intermediates as they moved through the restriction fragment. These produced a faint "Y arc" after 2D gel electrophoresis (white arrow in the 0-minute time point of Fig. 7A). Fifteen minutes after HU removal, the Y arc signal became stronger. At 30 and 45 minutes after HU removal, the Y arc signal became even stronger, and a "bubble arc" appeared (gray arrows in Fig. 7A). The bubble arc indicated that, in some of the cells, AT1045 functioned as an origin after cells were released from the HU block. Thus, AT1045 is a late-firing origin. These results explain why our microarray analyses detected only very weak replication at AT1045. The HU-treated cells used for our microarray experiments corresponded to the HU-treated cells analyzed at the 0-minute time point in Fig. 7A. In the microarray experiments, the region encompassing AT1045 would have replicated in only a small fraction of the cells. The same rationale (origin firing only after removal of the HU block) can explain the exceptional case of AT2067 (Additional File 10; Fig. 7C), and it certainly explains the exceptional case of Ori6 (Additional File 10; Fig. 7D). A 2D gel analysis of an Ori6-containing restriction fragment was carried out by D. D. Dubey and colleagues (personal communication; submitted for publication) on cells synchronized by HU-block-and-release, as in Fig. 7A. Results similar to those in Fig. 7A were obtained. The restriction fragment containing Ori6 showed maximum signals from bubble and Y arcs at 45–60 minutes after release from the HU block, but showed very little signal from such arcs at 0 minutes. These examples therefore suggest that *S. pombe *cells may contain many late-firing origins that escape detection when analysis is confined to HU-blocked cells. Since all of the microarray experiments, ours included, employed HU-blocked cells, the current set of microarray experiments is likely to have overlooked those origins that fire only in late S phase.

Some of the additional apparent exceptions to the general correlation between our microarray results and 2D gel electrophoretic results are dealt with in the figure of Additional File 11, and some further examples of correlation between late-replicating origins and microarray results are provided in Additional File 12. The extensive correlation between molecular combing analyses on chromosome 3 [13] and microarray results is described in Additional File 13. The general conclusions from Figure 7 and Additional Files 10, 11, 12, 13 are: (i) the correspondence between 2D gel measurements and microarray measurements is excellent and may reach as high as 98%, (ii) most apparent exceptions to this correlation appear to be a consequence of the failure of microarray measurements on HU-blocked cells to detect certain origins that are active only in late S phase, regardless of whether those origins are examined in wild-type cells or in checkpoint-mutant cells, and (iii) the correlation between microarray measurements and molecular combing measurements is also excellent.

### Extensive correspondence between replication in HU-treated wild-type and checkpoint-mutant cells

It is evident in Figs. 2 and 7 and in Additional Files 11, 12, 13 that for most probes the signals produced by wild-type cells were similar to those generated by checkpoint-mutant (*cds1*Δ and *rad3*Δ) cells. Clearly, for the regions displayed in these figures, at least, our results for *S. pombe *are strikingly different from the observations in *S. cerevisiae *that 2/3 or more of replication origins fire in HU-treated *rad53*-mutant cells but not in HU-treated wild-type cells [34,35]. To measure the frequency of similar checkpoint-restrained origins in *S. pombe *cells, we examined each putative origin (Fig. 6, Additional File 7) for significant differences between the wild-type signal and the checkpoint-mutant signals. Since our hypothesis was that origins would be regulated by a checkpoint pathway dependent on both Rad3 and Cds1, we required that the signals from *cds1*Δ and *rad3*Δ cells *both *differ from the wild-type signal by more than 20 percent, consistently for at least two of the four (or more) signals that we evaluated for each origin (see Methods for details).

We found only 21 origins (3.2% of the 657 origins classified as "very weak" or stronger) for which the signals in checkpoint-mutant cells were, by these criteria, higher than the signals for wild-type cells (Additional Files 7 and 8). Twenty of the 21 checkpoint-restrained origins found in our study were present on chromosomes 1 and 2; only one was located on chromosome 3 (Additional Files 7 and 8). In Fig. 6, positions indicated by the letter "C" show the locations of these checkpoint-restrained origins. The distribution is non-random, with checkpoint-restrained origins frequently being weak or very weak and being more abundant near chromosome ends. Since chromosome 3 contains a smaller proportion of weak and very weak origins and has ends that are different from those of chromosomes 1 and 2 (the ends of chromosome 3 contain tandem rDNA repeats and are located in nucleoli), it is not surprising that we found only one checkpoint-dependent origin on chromosome 3. An example of a checkpoint-restrained origin near the right end of chromosome 1 is shown in part A of Additional File 14.

We were surprised to discover that a larger number of predicted origins (36, or 5.5% of the total; Additional Files 7 and 8) function significantly better in wild-type cells than in checkpoint-mutant cells. These wild-type-dependent (more accurately, checkpoint-dependent) origins are indicated by the letter "W" in Fig. 6. Checkpoint-dependent origins are found in all three chromosomes, tend to be medium or strong, and are more frequent in chromosome interiors than near chromosome ends. An example of two adjacent checkpoint-dependent origins is provided in part B of Additional File 14.

### Correspondence between telomeric heterochromatin and replication-checkpoint-dependent restraint of replication in HU

**Figure 8:**
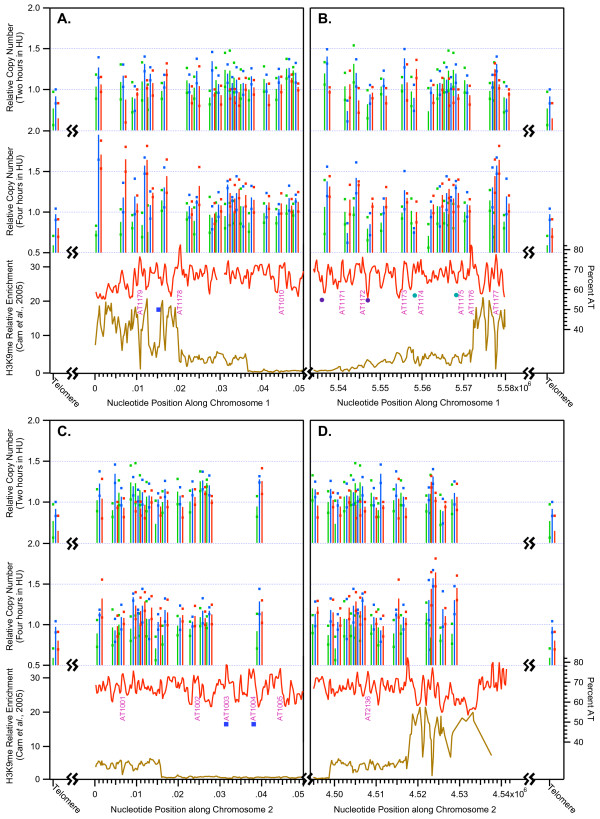
**Replication of subtelomeric heterochromatin in the presence of HU is restrained by the Rad3- and Cds1-dependent replication checkpoint**. Here the microarray results at the ends of the sequenced portions of chromosomes 1 and 2 are shown in comparison with the relative enrichment for methylated histone H3 lysine 9 (H3K9me; gold lines at bottoms of graphs) determined by Cam *et al. *[42]. The "telomere" probe is from the same region of telomere-associated sequences employed by Kim and Huberman [18] for their analysis of telomere replication timing in wild-type and checkpoint-mutant strains. (A) Left end of chromosome 1. (B) Right end of chromosome 1. (C) Left end of chromosome 2. (D) Right end of chromosome 2. The colored symbols representing origins discovered in other laboratories are sparse (A)-(C) or missing (D) in these subtelomeric regions, because the probes used in the other laboratories frequently did not extend to the ends of the 2006 versions of the sequences for *S. pombe *chromosomes 1 and 2.

Figs. 6A and 6B show that there is a higher frequency of checkpoint-restrained origins near the ends of chromosomes 1 and 2 than in their interior. We wondered whether these checkpoint-restrained origins might correlate with the presence of heterochromatin in telomeric regions. Cam *et al. *[42] have used chromatin immunoprecipitation and high-density tiled microarrays ("ChIP on chip") to measure the abundance of heterochromatin markers, including methylated lysine 9 in histone H3 (H3K9me), across the fission yeast genome. They found H3K9Me and other heterochromatin markers at telomeres, centromeres, the silent mating type region, rDNA and, at lower levels, at certain genes. We have downloaded the point-by-point measurements of Cam *et al. *[42] from their Internet site [43] and have graphed (Fig. 8) the abundance of H3K9me for approximately 50 kb near each telomere of chromosomes 1 and 2 together with our own copy number measurements. The relative enrichments of H3K9me [42] are shown by the golden lines at the bottom of each panel in Fig. 8. The other components of Fig. 8 are similar to those of Figs. 2, 7, and Additional Files 11, 12, 13. To interpret Fig. 8, it is important to know that – due to technical problems caused by multiple repeated motifs – the nucleotide sequences for chromosomes 1 and 2 do not extend to the true chromosome ends. Ten kb or more of nucleotide sequence are missing from each end. In addition, although the nucleotide sequences for stretches of 5–15 kb at the true chromosome ends have been determined [44,45], it is not yet known which variation of these true telomere sequences (the variants are extremely similar to each other) is associated with which chromosome end. In this study, in addition to ORF probes we used PCR probes corresponding to true telomere sequences within the 800-bp stretch previously employed as a probe in our 2D gel studies of telomere replication timing [18]. These probes detect telomere-associated sequences immediately adjacent to the simple-sequence telomere repeats at all four ends of chromosomes 1 and 2. The averaged results obtained with these probes are plotted in each panel of Fig. 8 to the left or right of the known left-end or right-end sequences (respectively) of these two chromosomes.

The amount of nucleotide sequence that is missing from each chromosome end is unknown, but it is thought that the known sequence at the left end of chromosome 1 is closest to the true telomere, and it is thought that approximately 10 kb of sequence are missing in this case [42,46]. The microarray probes used by Cam *et al. *[42] detected elevated enrichment (mostly > 10-fold) of H3K9me in the leftmost 20 kb of chromosome 1 (Fig. 8A). It is remarkable that, within the same 20-kb stretch, every one of our six probes detected higher copy number levels in the *cds1*Δ and *rad3*Δ strains than in the wild-type strain at the 4-hour time point. In the stretch between 20 and 36 kb from the left end of chromosome 1, Cam *et al. *found reduced enrichment of H3K9me. In this stretch, we found that five of our nine probes detected elevated copy numbers in checkpoint-mutant strains at 4 hours. Rightward of 36 kb, the enrichment of H3K9me was at background level (approximately 1), and this background level was maintained in most of the rest of chromosome 1, with the exception of the centromere and right telomere [42]. Nowhere else in the chromosome except at the right end (Fig. 8B) – even at the origins identified as checkpoint-restrained (marked "C" in Fig. 6) – is there a stretch with so many *contiguous *probes showing significantly higher copy number at 4 hours in the checkpoint-mutant strains (Additional File 1). At the right end of chromosome 1 (Fig. 8B) our measurements are consistent with those at the left end (Fig. 8A). In the stretch from 5.572 to 5.580 Mb, enrichment for H3K9me is high, and all three probes in this stretch detected elevated copy numbers after 4 hours in HU in the checkpoint-mutant strains. From 5.540 to 5.572 Mb, enrichment of H3K9me is less, and 7 of the 12 probes in this stretch detected elevated copy numbers in checkpoint-mutant strains at the 4-hour time point. To the left of 5.540 Mb, H3K9me is not enriched. Although the probe in this portion of Fig. 8B detected higher copy numbers in the checkpoint-mutant strains at 4 hours, the vast majority of probes further to the left (Additional File 1) did not.

Similar results were obtained at the left and right ends of chromosome 2 (Figs. 8C and 8D). Cam *et al. *[42] detected only moderate enrichment of H3K9me in the stretch from 0 to 15 kb at the left end of chromosome 2. Presumably the absence of higher enrichment is a consequence of the absence of unambiguous nucleotide sequence further to the left. In the stretch moderately enriched for H3K9me, six of nine probes detected enhancement of checkpoint-mutant copy numbers relative to wild-type at the 4-hr time point. In the stretch within Fig. 8C to the right of 15 kb, where H3K9me is not enriched, only one of five probes detected significant checkpoint-mutation-dependent signal enhancement. At the right end of chromosome 2, in the highly H3K9me-enriched stretch from 4.518 to 4.542 Mb, all four probes detected checkpoint-mutation-dependent signal enhancement (Fig. 8D). However, in the moderately enriched stretched from 4.499 to 4.518 Mb, four of the 10 probes were not significantly affected by checkpoint mutations. Although the leftmost probe in Fig. 8D, which is the only probe in this panel from a region not enriched in H3K9me, detected modest copy number enhancement at 4 hours in checkpoint mutant strains, the vast majority of probes further to the left did not (Additional File 2). Thus there is a striking correlation between the distribution of H3K9me at the ends of chromosomes 1 and 2 and the consistency and extent of checkpoint-mutation-dependent replication in HU.

**Figure 9:**
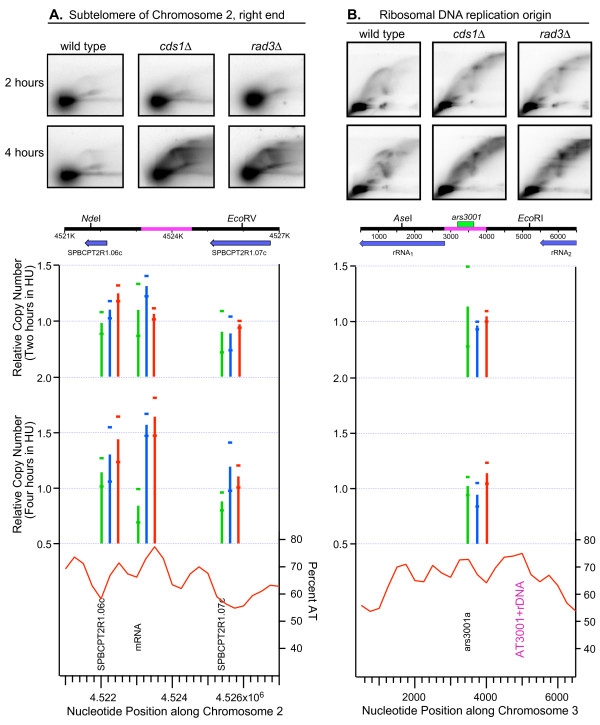
**Checkpoint-mutation-dependent late replication of subtelomeric sequences but not ribosomal DNA in HU-treated fission yeast cells**. The same strains (JLP1164, JLP1257 and JLP1260) were employed as for the microarray experiments. All three strains contain the *cdc25-22 *mutation, which allowed them to be blocked in G2 by incubation at 36.5°C. The cells were released from the G2 block and further incubated at 25°C for two or four hours in the presence of 15 mM HU. DNA was isolated at the indicated times, digested with the indicated restriction enzymes, and processed for 2D gel electrophoresis. The diagrams under the 2D gel panels show 6-kb stretches containing the studied restriction fragments (as in Fig. 7A). The horizontal axes of the graphs were adjusted to correspond in scale to the restriction fragment diagrams. In this figure there are no colored symbols representing the locations of origins discovered in other laboratories [34] [14] [15], because none of the other laboratories employed microarray probes in these regions. (A) The probe detected a restriction fragment centered on nucleotide position ~4.524 Mb, in the heterochromatic region near the right end of chromosome 2 (Fig. 8B, Additional File 12). Strong Y arcs, and a smear of replication intermediates with altered structures, are evident only in the checkpoint-mutant strains at the 4-hour time point. (B) The probe detected a restriction fragment centered on *ars3001 *in the rDNA repeats. Bubble arcs are evident only in the samples from wild-type cells, suggesting that the replication checkpoint is required to prevent loss of bubbles under these experimental conditions.

The induction of replication at the 4-hour time point in the heterochromatic subtelomeric regions of the checkpoint-mutant strains (Fig. 8) seemed so strong that we thought it likely that we would be able to find evidence for subtelomeric origin activation in checkpoint-mutant strains at the 4-hour time point by the appearance of bubble arcs in 2D gel electrophoretic analyses. To test this possibility, we analyzed a *Nde*I-*Eco*RV restriction fragment approximately centered on the peak of A+T near the highly-induced region at nucleotide position ~4.523 Mb at the right end of chromosome 2 (Fig. 8D, Fig. 9A, Additional File 8). As shown in Fig. 9A, the 2D gels confirmed the impressive induction of replication in this region at the 4-hour time point in the checkpoint-mutant strains. However, although Y arcs were easily visible in the checkpoint-mutant strains at 4 hours, no bubble arcs were evident. This failure to detect the anticipated bubble arcs could be a consequence of (i) the origin responsible for replicating this region being located near the ends or outside of this restriction fragment, in which case only Y arcs would be visible, or (ii) breakdown of bubble structures within this restriction fragment during the four-hour incubation in the presence of HU in the absence of the replication checkpoint, one of whose functions is to stabilize replicating DNA.

To distinguish between these possibilities, we examined the stability of bubble structures generated at a known replication origin in the wild-type and checkpoint-mutant strains. We digested some of the same DNA used in Fig. 9A with a different pair of restriction enzymes (*Ase*I and *Eco*RI) to generate a fragment centered on the previously characterized replication origin (*ars3001*; [8,47]), which is located in each of the ~75 ribosomal DNA (rDNA) repeat units, each 10.9 kb in length, at the left and right ends of chromosome III. Based on measurements in unsynchronized cells, *ars3001 *fires in ~50% of repeat units. The other ~50% are passively replicated [47]. However, for the synchronized wild-type cell population studied in Fig. 9B (left panels), the replication forks generated at *Ase*I-*Eco*RI restriction fragments containing functional origins were prevented by HU from reaching the corresponding restriction fragments in neighboring repeat units. Consequently, only a bubble arc and the late portion of a Y arc (due to bubble runoff) are visible at the 2-hour time point. By the 4-hour time point, the small replication bubbles that had populated the early (left, lower) portion of the bubble arc at 2 hours had progressed to larger bubbles that now populated the late (right, upper) portion of the bubble arc. Some of the large bubble structures were converted to late Y structures (descending portion of Y arc on right-hand side), when one of the bubble-structure's replication forks passed one of its restriction sites (bubble runoff). The absence of early bubble or Y signals at the 4-hour time point is an indication that there were no new initiation events between 2 hours and 4 hours and that replication forks from the active origins had not yet progressed into the corresponding restriction fragments in neighboring rDNA repeat units containing inactive origins.

Consistent with the role of the replication checkpoint in maintaining fork stability, at 2 hours the two checkpoint-mutant strains produced no detectable bubble arcs and only very faint Y arcs, plus other structures and a background haze that have not yet been characterized (Fig. 9B, center and right panels, 2 hours). At 4 hours, all of the signals visible at 2 hours (Y arcs, haze, other structures) were stronger, but still no bubble arcs were detectable (Fig. 9B, center and right panels, 4 hours). These results from the well-studied rDNA repeats suggest that a functional replication checkpoint is essential for maintaining replication bubbles when fission yeast cells are treated with HU. Therefore, the absence of a bubble arc from the subtelomeric *Nde*I-*Eco*RV fragment at 4 hours in the checkpoint-mutant strains (Fig. 9A) could well be due to breakdown of bubble structures in the absence of a functional checkpoint. Despite the lack of a bubble arc, however, the strong induction of replication of this restriction fragment after 4 hours of HU treatment in the checkpoint mutant strains (compared to the wild-type strain) is clearly evident (Fig. 9A).

## Discussion

### Quality of results

Here we report the results and conclusions of a microarray-based analysis of DNA replication in fission yeast cells entering S phase in the presence of HU. Our PCR-based microarrays contained probes for all known *S. pombe *ORFs and for hundreds of additional sequences. The reproducibility of our measurements was good (Figs. 2, 7, 8, 9 and Additional Files 1, 2, 3 and 11, 12, 13, 14), and the agreement between our measurements and previous results was excellent. Approximately 83% of the origins theoretically predicted on the basis of high AT content [20] or experimentally predicted based on the presence of a pre-RC [15] coincided with signals suggestive of origin function (greater than our threshold value of 1.1; Additional File 8). Approximately 93% of the origins experimentally identified on the basis of single-strandedness [34] or on the basis of copy number [14] in cells entering S phase in the presence of HU similarly coincided with above-threshold values in our microarrays (Additional File 8). That our measures of replication in HU-blocked cells are more highly correlated with previous measures of replication in HU-blocked cells [34,14] than with predicted AT islands [20] or measured pre-RCs (which were identified in G1-arrested cells, not HU-blocked cells; [15]) is not surprising and is likely due to a subset of potential origins at AT islands and at pre-RCs failing to fire in HU-blocked cells (Fig. 7 and Additional Files 11 and 12). Some of these potential origins that do not fire in HU-treated cells might be active in late S phase or might not be active at all in their native chromatin context.

In addition, our results are in agreement with 90–98% (depending on one's tolerance for rationalizations) of previous 2D gel measurements of origin function at 51 potential origin locations (Fig. 7; Additional Files 10 and 11)). Our results are also in excellent agreement with previous molecular combing measurements [13] of origin locations in two portions of chromosome 3 (Additional File 13). This extensive agreement with previous measurements gives us confidence in the validity of our results and in the value of conclusions derived from them.

### Limitations of results

There are, however, some limitations of our study (and of all the other first-generation microarray studies in fission yeast) that the reader should keep in mind. First, all microarray studies have a sensitivity limit. Origins that fire only rarely are likely to fall below that limit. Fission yeast differs from budding yeast in having a higher proportion of relatively inefficient origins. Therefore, the number of inefficient origins that may have been overlooked by microarray studies in fission yeast could be considerably larger than in budding yeast.

In addition, our investigations reveal that in fission yeast some origins cannot be detected by microarray analyses of HU-blocked cells, because these origins, which ordinarily replicate in late S phase, do not fire in the presence of HU even in checkpoint-mutant cells. These are discussed in more detail below, under "Replication Timing". To find such origins by microarray analyses, one would need to obtain relatively noise-free results, preferably using high-resolution tiled microarrays, at various times during late S phase in the absence of HU.

### Nature of fission yeast DNA replication origins

It is evident from the extensive agreement between our results and the potential origin locations predicted solely on the basis of AT content by Segurado *et al. *[20] (Figs. 2, 6; Additional Files 1, 2, 3, 13, 14) that AT content is a major determinant of origin function in fission yeast. This is also evident when one compares AT content in a 500-bp window (the wavy red line in our graphs) with extent of replication (Fig. 2; Additional Files 1, 2, 3, 13, 14). The high AT content of *S. pombe *origins has also been noted by other investigators [5,7,9,10,20,22].

However, it is also evident that AT content is not the only factor determining origin function. A good example is provided in Additional File 13, where the region centered at 1.97 Mb along chromosome 3, which is not an AT island, displays stronger replication signals than the region centered at 1.87 Mb, which is an AT island (AT3058). Many additional examples can be seen by examining the graphs that cover the three chromosomes (Additional Files 1, 2, 3). Such additional factors – beyond AT content – may be responsible for the regional variations in strengths of replication signals evident in Fig. 6. For example, the weak region from 0.2 to 0.82 Mb in chromosome 1 contains many AT-rich AT islands, yet none of these AT islands scores higher than medium, and many of them score below our detection limit. In contrast, in the region from 3.00 to 4.34 Mb, most AT islands score as medium or strong, and none are below the detection limit (Fig. 6A). It will be interesting in future experiments to identify the factors that allow some large regions to replicate efficiently in HU while other regions are inhibited.

Others [10,13] have suggested that the *S. pombe *genome contains a very large number of potential origins of varying efficiencies and that the patterns of origin usage during any single S phase in any single cell are determined stochastically. Our results are consistent with this hypothesis. We found a large number of potential origins with a wide range of efficiencies. If we lowered our detection threshold, we would have increased the number of origins identified.

### Replication timing

Although evaluation of replication timing was one of our goals, we chose to carry out the analyses of origin function reported here during HU-induced "replication stress" conditions, due to the technical advantages conferred by this treatment. HU slows replication fork movement, limiting it to within ~10 kb of origins. Thus higher copy numbers can accumulate at origin regions, and the signals are not diluted by passive replication as occurs during unperturbed S phase. To find out if measuring origin function during replication stress can provide useful insight into normal S-phase replication, both Heichinger *et al. *[14] and we used copy number to monitor replication during a synchronous "normal" S phase in the absence of HU. Our data showed a strong correlation between origin usage in normal S phase and in HU, but there was substantially greater noise in our normal S-phase experiment (data not shown) than in the HU experiments. Heichinger *et al. *[14] also found strong similarity between their HU experiments and their synchronous normal S-phase data. Thus the copy-number/HU method appears well-suited to providing a clear picture of the function of the majority of replication origins, with analysis of only a few samples. The BrdU incorporation method employed by Hayashi *et al. *[15] appears similarly well suited to evaluating replication origin function. Finally, we, Heichinger *et al. *[14], and Hayashi *et al. *[15] used the *cdc25-22 *mutation to facilitate synchronization of our cultures. Because cell cycle progression is so intimately linked with replication initiation and chromatin structure, it is possible that the *cdc25-22 *mutation itself may have influenced our results.

While acknowledging these limitations of our method for studying replication timing, we anticipated that we would nevertheless be able to identify potentially late-replicating origins by comparing results from wild-type cells with results from checkpoint-mutant cells. This anticipation was based on earlier studies, which showed that a significant fraction of origins in budding yeast cells does not fire in HU in wild-type cells but does fire in checkpoint-mutant cells, and most of these checkpoint-restrained origins are late-replicating [48,49,34,35]. To our surprise, however, our results showed that very few (only ~3%) of the potential origins in *S. pombe *cells treated with 15 mM HU replicate to a significantly greater extent in checkpoint-mutant cells than in wild-type cells. To confirm this unanticipated result, we repeated the experiment with a higher concentration of HU (25 mM). The results (not shown) proved similar to those obtained with 15 mM HU.

Some, perhaps all, of the 3% of potential origins that appear to be checkpoint-restrained may prove to be late-replicating. Of these apparently checkpoint-restrained potential origins, only two have been studied previously. Both replicate in late S phase. One is in the Telomere-Associated Sequences, located in the terminal *Hin*dIII restriction fragments at the ends of chromosomes 1 and 2, which replicate at the end of S phase [18]. The nearby subtelomeric heterochromatic regions also appear to be late-replicating (Figs. 8, 9). The other previously studied potential origin that appears to be checkpoint-restrained is AT2103 (*ars727*; Additional File 11), which is passively replicated in late S phase in wild-type cells [18]. Whether *ars727 *functions as an origin in HU-treated checkpoint-mutant cells has not yet been tested by 2D gel electrophoresis and may be difficult to test, given the instability of replication bubbles in HU-treated checkpoint mutant cells (Fig. 9). In general, the apparently checkpoint-restrained potential origins tend to be weak and to be located near chromosome ends (Fig. 6). Although subtelomeric heterochromatin shows the clearest evidence of region-wide checkpoint restraint (Figs. 8, 9), there are additional origins that appear to be checkpoint-restrained and that are located further from telomeres (Fig. 6).

Although all checkpoint-restrained origins may prove to be late-replicating, our results clearly demonstrate that, under our experimental conditions with limiting dNTPs, some *S. pombe *late-replicating origins are not restrained solely by the replication checkpoint. For example, AT1045 is replicated (and even functions as an origin) in late S phase, and it does not replicate much in HU-treated cells, whether they are wild-type or checkpoint-mutant (Fig. 7A, B). The same is true of the region called "Ori6" on chromosome 2 (Fig. 7D). It is likely to be true for AT2067 (also on chromosome 2; Fig. 7C), and is also likely to be true for many of the weak/late pre-RCs identified by Hayashi *et al. *where no significant incorporation of BrdU was detected in HU-treated wild-type or checkpoint-mutant cells [15]. Based on the results of Hayashi *et al*., about 1/3 of all *S. pombe *pre-RCs must be either late-replicating or inefficient or both [15]. Note that our results do not exclude the possibility that the replication checkpoint may be one of two or more parallel pathways restraining these late/inefficient origins in HU-treated cells. What our results prove is that there is at least one additional pathway that prevents these late/inefficient origins from firing in HU-treated cells, even in the absence of the replication checkpoint. The additional pathway may be something as simple as the concentration of dNTPs – perhaps the late/inefficient origins cannot fire if the dNTP concentration is too low.

Rhind [50] has proposed that there may be no specific replication timing program in *S. pombe*. Instead, Rhind suggests that the general probability of origin firing may increase during S phase. Thus efficient origins, which can fire even when the general probability of origin firing is low, will tend to fire in early S phase, while inefficient origins that are located far from efficient origins (so they won't be passively replicated by forks coming from efficient origins) will tend to fire in late S phase [50]. If this view is correct (note: none of the results presented here is inconsistent with this view), then chromosomal regions containing mostly inefficient origins (Fig. 6A,B) may prove to be late-replicating. It is interesting that AT1045 (which replicates late; previous paragraph) is located in a short region, at about 1.15 Mb on chromosome 1, that contains four weak or very weak potential origins (Fig. 6A), while *ars2-2 *(which also replicates late) is located in a broader region (4.21–4.43 Mb on chromosome 2) with a mixture of weak, very weak, and below-limit potential origins (Fig. 5B). These observations tend to support Rhind's hypothesis [50]. If Rhind's hypothesis proves correct, then the two questions that will need to be answered to understand replication timing will be (i) what factors lead to regional variations in origin efficiency, and (ii) what is the mechanism by which the probability of origin firing increases during S phase? It is interesting that the concentration of dNTPs increases during S phase in mammalian cells [51]. So far as we are aware, the intracellular concentrations of dNTPs have not been measured during the cell cycle in fission yeast. Another factor that may affect replication timing is Dfp1, the fission yeast regulatory subunit of the DDK kinase, which is essential for initiation at all replication origins (reviewed in [1]). Brown and Kelly [52] have shown that the concentration of Dfp1 increases four-fold during S phase. It is likely that the concentration of CDK, another potentially rate-limiting initiation factor, also increases during S phase.

### Checkpoint-Dependent Origins

We were surprised to find that about 5% of the origins we evaluated replicated to a significantly greater extent in wild-type cells than in checkpoint-mutant cells (Fig. 6, Additional Files 8, 14). These origins, therefore, appear to require a functional replication checkpoint. We had not anticipated this observation, but during the course of our studies Raveendranathan *et al. *[35] reported a low frequency of checkpoint-dependent origins in budding yeast, similar to what we observe in fission yeast. Raveendranathan *et al*. attributed these unusual origins to the stalling or collapse of replication forks (due to the absence of the replication checkpoint) so close to the origins where the forks were created that little or no replication signal was generated. The replication checkpoint is similarly necessary for replication fork stability in fission yeast. This is dramatically demonstrated in Fig. 9 (4-hour time point, checkpoint-mutant strains) where such a large variety of structurally altered replication intermediates is generated in the checkpoint-mutant strains that the region of the 2D gel coinciding with, below, and to the right of the region normally occupied by simple Y- and bubble-arcs contains a dense haze of signal. It is easy to imagine that genomic regions that are normally difficult to replicate would prove much more difficult to replicate in checkpoint-mutant strains. If these regions were located very close to origins, they would undoubtedly lead to reduced signals in checkpoint-mutant strains from the probes flanking the origin, as proposed by Raveendranathan *et al. *[35].

Raveendranathan *et al. *[35] also noticed that the effects of a *rad53 *mutation on origin firing in the presence of HU were not completely identical to the effects of a *mec1 *mutation. The authors suggested that the differences in results between *rad53 *and *mec1 *mutant strains were likely due to the existence of both Mec1-dependent, Rad53-independent and Rad53-dependent, Mec1-independent pathways that could affect origin usage. Similarly, we noticed that many of our probes produced signals differing significantly between the *cds1*Δ and *rad3*Δ strains in fission yeast. Examples can be seen in Figs. 2, 7, 8, and Additional Files 11, 12, 13, 14. Most of these differences occurred at single probes and were not repeated in adjacent probes and are therefore likely to be due to noise. However, in a few cases the differences appeared to be consistent among neighboring probes (Additional Files 1, 2, 3). In future experiments, it will be interesting to test the reproducibility of these apparently consistent differences between *cds1*Δ and *rad3*Δ strains, and, if they prove reproducible, attempt to identify the pathways involved.

### Checkpoint-dependent inhibition of origin firing is unlikely to play a significant role in maintaining genome stability in fission yeast

In this section, we discuss the implications of our finding that only about 3% of the origins that we evaluated were significantly induced by deletion of the *rad3 *or *cds1 *checkpoint gene in HU-treated cells (Fig. 6; Additional Files 7, 14). We interpret this to mean that the level of replication for 97% of origins was not significantly increased in HU-treated checkpoint-mutant cells compared to HU-treated wild-type cells. In the absence of independent information regarding the extents of replication in HU-treated cells, however, one might argue that – since microarray results are always normalized to the genome average – *all *origins may be induced in HU-treated checkpoint-mutant cells, and 3% of them may be induced to a level that is significantly higher than the genome average. We think this possibility is unlikely for two reasons. First, Fig. 1B shows – by flow cytometry – that the extent of replication for the genome as a whole was in fact significantly less for the checkpoint-mutant cells at the 4-hour time point than for the wild-type cells. Second, by using another independent method – 2D gel electrophoresis – we showed (Fig. 9B) that, for a restriction fragment containing the rDNA replication origin, for which the microarray signals were similar between the wild-type and checkpoint-mutant strains, the total amount of replication intermediates of all sorts (intact and broken) increased only modestly from 2 to 4 hours, and it increased by roughly the same ratio for wild-type and checkpoint-mutant cells. The most likely interpretation therefore is that, for most origins, the extent of firing in HU-treated cells was affected only slightly, if at all, by the replication checkpoint.

Our observation that only about 3% of fission yeast origins are significantly induced by abrogation of the replication checkpoint in HU-treated cells is consistent with Heichinger *et al.*'s finding [14] that only about 2% of origins were induced in HU by deletion of the *rad3 *gene. At first glance these results appear inconsistent with Feng *et al.*'s finding [34] that more than one third of *S. pombe *origins appeared to be induced in HU by deletion of the *cds1 *gene. This apparent discrepancy can be explained by the fact that Feng *et al. *measured amounts of single-stranded DNA. Because the Cds1- and Rad3-dependent replication checkpoint is required to stabilize replication forks and prevent the generation of large amounts of single-stranded DNA in HU-treated cells, in fact much larger amounts of single-stranded DNA were generated in the *cds1*Δ cells than in the wild-type cells employed by Feng *et al. *[34]. We think it is likely that the greatly increased single-stranded DNA at each origin in the *cds1*Δ cells permitted the detection of more origins in the *cds1*Δ cells than in the wild-type cells. This hypothesis is reinforced by our observation that the origins detected in wild-type cells by Feng *et al. *correspond more frequently to our "strong" and "medium" origins than do the origins that Feng *et al. *detected in *cds1*Δ cells (Fig. 6; Additional File 8; [34]). The hypothesis is also consistent with the absence of significant co-localization between origins identified in *cds1*Δ cells by Feng *et al. *(orange dots in Fig. 6; [34]) and the checkpoint-restrained origins identified by us ("C" in Fig. 6) or by Hayashi *et al. *(red or dark blue squares rather than red or dark blue circles in Fig. 6; [15]). If our view is correct, then most – perhaps all – of the origins detected in *cds1*Δ cells that were not also detected in wild-type cells by Feng *et al. *[34] were not checkpoint-restrained but simply did not generate enough single-stranded DNA in the wild-type cells to be detected.

The result obtained by us and by Heichinger *et al. *[14], that only a few percent of origins is checkpoint-restrained in fission yeast, also appears (at first glance) to be contradicted by Hayashi *et al.*'s observation that incorporation of BrdU at 22% of pre-RCs was somewhat greater in HU-treated *cds1*Δ cells than in HU-treated wild-type cells [15]. But Hayashi *et al. *also noted that in most cases the increase in incorporation in *cds1*Δ cells was only slight, so the only way to detect it was by calculating the ratio of signal strength in *cds1*Δ cells to the corresponding signal strength in wild-type cells. In fact, the only region where this calculation was not required to detect a difference between wild-type cells and *cds1*Δ cells was in the subtelomeric regions, where Hayashi *et al.*, like us, observed significant enhancement of replication in HU-treated *cds1*Δ cells compared to wild-type cells [15]. Thus the difference between Hayashi *et al. *[15] on the one hand (22% of origins checkpoint-restrained) and us and Heichinger *et al. *on the other (2–3% of origins checkpoint-restrained) appears to be largely semantic. Our criteria for identifying checkpoint-restrained origins (and the criteria of Heichinger *et al. *[14]) appear to be somewhat stricter than the criteria employed by Hayashi *et al*. [15]. All three groups are in agreement that checkpoint mutations produce very little change in the replication pattern of HU-treated fission yeast cells, except at the telomeres.

When fission yeast DNA is treated with the alkylating agent MMS, progression through S phase is slowed in a replication checkpoint-dependent manner [26-29]. If most origins in MMS-treated cells are as unaffected by replication checkpoint mutations as they are in HU-treated cells, then the checkpoint-dependent slowing of S phase by MMS is likely to be accomplished largely in some other manner – not by inhibition of origin firing. The only other way to inhibit replication, other than by inhibiting origins, is to inhibit replication fork movement. In mammalian cells with damaged DNA, replication is slowed both by origin inhibition and by fork retardation [16,31-33]. In budding yeast, replication is slowed only by inhibiting origins; no checkpoint-dependent fork slowing is detectable [25]. In contrast, the consensus of the microarray results (our results; [14,15]) leads to the conclusion that origin inhibition may not be the primary mechanism for checkpoint-dependent slowing of S phase in fission yeast. The primary mechanism may prove to be inhibition of replication fork movement. In addition to the primary mechanism, origins at the telomeres and at a few other locations in the fission yeast genome *are *inhibited by the replication checkpoint, and that inhibition is expected to contribute, even if the contribution is limited to just ~3% of the total, to the overall slowing of S phase when DNA is damaged.

### Strong correlation between checkpoint restraint and subtelomeric heterochromatin

Another surprise emerging from our results is the striking correlation between subtelomeric heterochromatin, located by "ChIP on chip" experiments [42], and checkpoint-mutation-dependent replication at the late time point (4 hours) in HU (Figs. 8, 9). The fact that checkpoint-restrained replication is evident in the 4-hour time point but not in the 2-hour time point is consistent with previous 2D-gel measurements showing that deletion of the *cds1 *or *rad3 *genes permits replication of Telomere-Associated Sequences in HU, but only at late times [18]. In other words, the replication checkpoint inhibits the replication of telomeric DNA but is not solely responsible (if it is responsible at all) for the late timing of such replication.

In contrast to its strong effect on the replication of telomeric and subtelomeric DNA in the presence of HU, the replication checkpoint has no detectable effect on the replication in HU of the other forms of heterochromatin in fission yeast (centromeric, mating-type, and ribosomal DNA (rDNA); Fig. 9B; Additional Files 1, 2, 3. Note: two of our PCR probes, "L-domain" and "matmc", were specific for the mating-type heterochromatic region between IR-L and IR-R). These other forms of heterochromatin also differ from telomeric and subtelomeric heterochromatin in the sense that they are early-replicating [18,19], while telomeric and subtelomeric heterochromatin are late-replicating ([18]; Figs. 8, 9A; Additional File 12).

With respect to its late replication, telomeric and subtelomeric heterochromatin in fission yeast resembles most heterochromatin in animal cells. However, examples of early-replicating heterochromatin (similar to fission yeast centromeric, mating-type and rDNA heterochromatin) are also known in animal cells (reviewed in [19]). The fact that some heterochromatin replicates early while other heterochromatin replicates late shows that it is not the condensed state of heterochromatin that makes most of it replicate late in S phase in animal cells (or in fission yeast telomeric regions). Instead, there must be something different about late-replicating heterochromatin, something that distinguishes it from early-replicating heterochromatin. The fact that both early- and late-replicating heterochromatin are present and easily studied in fission yeast cells provides a unique opportunity to identify the factor(s) responsible for the differences between them. It is possible that whatever distinguishes late-replicating from early-replicating heterochromatin in fission yeast will also prove to distinguish checkpoint-restrained replication origins from those that are not checkpoint-restrained.

### Note added in proof

After our manuscript was submitted for publication, another relevant manuscript was published by Eshaghi *et al. *[53], who used an ORF-based microarray to measure copy number changes when fission yeast cells that had accumulated in early S phase as a consequence of HU treatment were released from the HU block and permitted to continue through S phase. Consistent with Rhind's hypothesis [50], Eshaghi *et al. *found that the overall efficiency of replication increased during S phase.

## Conclusion

Here we have presented the results of the fourth in a recent series of independent microarray analyses of DNA replication as fission yeast cells enter S phase in the presence of HU. With respect to the locations and relative efficiencies of replication origins, our results are in good agreement with those obtained in the previous studies [34,14,15]. Our summary and comparison of our findings with those from previous studies (Fig. 6) provides a useful guide to all four analyses. Like the previous three analyses, our results confirmed earlier studies [20,10] suggesting that AT content is a major determinant of origin function in fission yeast. The results obtained in all four microarray studies also indicated that, in addition to AT content, other factors can influence origin function. Some of these other factors operate regionally, because origins of similar AT content initiate replication with very different efficiencies in different regions.

We were surprised to find that approximately 5% of replication origins functioned significantly more efficiently in HU-treated wild-type cells than in HU-treated checkpoint-mutant (*cds1*Δ or *rad3*Δ) cells. In other words, these origins appeared to require a functional replication checkpoint for full function. Raveendranathan *et al. *[35] recently observed similar checkpoint-dependent origins in budding yeast, and they attributed the apparent checkpoint-dependency of these origins to a requirement for a functional checkpoint for replication forks to move through difficult-to-replicate DNA near the origin. The apparently checkpoint-dependent origins that we observed in fission yeast may similarly reflect the presence of nearby difficult-to-replicate DNA.

In budding yeast, there is an excellent correlation between late-replicating origins and origins that are restrained by the replication checkpoint from firing at their normal times in HU-treated cells [54,49,34,35,40]. However, our results and those of Hayashi *et al. *[15] suggest that some fission yeast replication origins that are capable of firing in late S phase are restrained from firing at their normal times in HU-treated cells even when those cells have defective replication checkpoints. This observation suggests the existence in fission yeast of at least one checkpoint-independent mechanism capable of restraining the firing of late origins in HU-treated cells.

After accounting for the effects of checkpoint mutations on the sensitivity of the single-stranded-DNA-based origin-detection method [34] and after accounting for different criteria for significance in different studies [14,15], we were able to conclude that all four of the recent fission yeast microarray studies are consistent with the conclusion that only a small proportion (about 3% according to our criteria and those of Heichinger *et al. *[14]) of fission yeast origins are significantly restrained by the replication checkpoint alone from firing at their normal times in HU-treated cells. This small effect of the replication checkpoint on origin firing times in HU-treated fission yeast cells contrasts strikingly with the major impact of the replication checkpoint on origin-firing times in HU-treated budding yeast cells [34,35] and demonstrates that different eukaryotic organisms have evolved different strategies for maintaining genome stability when replication forks are slowed by dNTP depletion. Furthermore, the relatively minor impact of the replication checkpoint on origin-firing kinetics in fission yeast suggests that, when progress through S-phase is slowed by the replication checkpoint in MMS-treated cells [26-29], the slowing may be accomplished primarily by reduction of the rate of replication fork movement rather than by restraining replication origins.

Interestingly, our results also revealed that those replication origins that were checkpoint-restrained were concentrated near the heterochromatic telomeres of chromosomes 1 and 2. Indeed, we detected a striking correlation between the extent of checkpoint restraint and the degree of heterochromatinization at all four of these telomeres. In contrast, we were unable to detect any checkpoint restraint at the other heterochromatic portions of the fission yeast genome (centromeres, silent mating type region, and rDNA). Thus fission yeast telomeric heterochromatin must differ in some structural or compositional way from other forms of heterochromatin. Discovering what that difference is and elucidating how that difference leads to such a large distinction in checkpoint response are two exciting projects that remain for the future.

## Methods

### Strains

The strains employed were JLP1164 (*h*^+^*cdc25-22*) [55], JLP1257 (*h*^-^*leu1-32 ura4-D18 cdc25-22 cds1::ura4*^+^), and JLP1260 (*h*^+^*leu1-32 ura4-D18 cdc25-22 rad3::ura4*^+^).

### Generation of double-mutant strains

Strains JLP1257 and JLP1260 were isolated from tetrad dissections of crosses of *h*^+^*leu1-32 ura4-D18 cdc25-22 *from Paul Russell with strains 1562 (*h*^- ^*cds1*::*ura4 leu1-32 ura4-D18*) and 6G (*h*^- ^*rad3*::*ura4 ade6-704 leu1-32 ura4-D18*) from Anthony Carr, respectively.

### Cell culture and synchronization

Cells were grown in rich medium, (YES; [56]) at permissive temperature (25°C) to an OD_600 _of approximately 0.2 and then shifted to their restrictive temperature, 36.5°C, to arrest cells in G2. After 4 hours at 36.5°C, cells were shifted back to 25°C and hydroxyurea (HU; Fluka) was added to a final concentration of 15 mM. Cells were washed and harvested at 0, 2, and 4 hours after temperature downshift (release from G2 arrest). Cells were rapidly cooled during harvesting by adding ice, centrifuging at 4°C, washing with cold water, and quick-freezing with liquid nitrogen before storing at -80°C. Frozen cells were used for DNA extraction (see below). Cell samples for flow cytometry were removed before freezing, fixed with 70% ethanol, and stored at 4°C. To check cell synchrony, measurements of double cells, cells with septa, and single cells per 100 cells were made by phase contrast microscopy every 10 to 15 minutes after release from G2.

### Microarrays

The microarrays used in these experiments were created by the Leatherwood/Futcher microarray facility at the State University of New York at Stony Brook. Each microarray consisted of 5,407 spots of PCR products (0.1–1.2 kb) printed onto glass slides coated with aminopropylsilane (Erie Scientific). The PCR products spotted onto the microarray consisted of 4,824 predicted mRNAs, 504 predicted other RNAs and selected intergenic regions, 79 introns, 90 non-*S. pombe *DNAs with 60–80%, or less than 20%, identity to *S. pombe *sequences, and 503 spotting controls consisting of Cy3 and Cy5 labeled oligos, dilution series of highly expressed genes, controls for testing 3' labeling bias, and the same DNAs in multiple positions on the array.

### DNA processing, labeling, and hybridizations

Frozen cell pellets were washed with water and repelleted at room temperature. The cells were then resuspended in breaking buffer (2% Triton X-100, 1% sodium dodecyl sulfate (SDS), 100 mM NaCl, 10 mM Tris-Cl, pH 8.0, and 1 mM EDTA, pH 8.0). An equal volume of phenol/chloroform/isoamyl alcohol (PCI) and glass beads was added to the cells, and the suspension was vigorously shaken with a mini-beadbeater (BioSpecs Products) for 1 minute, then put on ice for 2 minutes. The shaking and ice incubations were repeated twice more. One half-volume of 10 mM Tris, 1 mM EDTA, pH 8.0 (TE) was added to the samples and mixed briefly (Vortex). The samples were then centrifuged at 16,100 × g for 8 min at room temperature. The aqueous layers were then transferred to pre-spun phase-lock tubes (Eppendorf), equal volumes of PCI were added, and the samples were shaken briefly (Vortex). Samples were clarified by centrifugation. The supernatants were transferred to new tubes, an equal volume of chloroform/isoamyl alcohol was added, and samples were mixed briefly (Vortex). After clarification by centrifugation, the supernatants were transferred to new tubes, an equal volume of cold 100% ethanol, and NaCl to a final concentration of 50 mM were added. The samples were mixed briefly (Vortex), then incubated at -80°C for at least 30 mins. The precipitated DNA was pelleted by centrifugation for 15 minutes at 16,100 × g (4°C). Supernatants were aspirated, and the DNA pellets were dried before resuspension in TE. RNA was removed by addition of RNAse A (Sigma) at a final concentration of 0.1 μg/μl and incubation at 37°C for at least 30 min. To remove contaminating proteins, Proteinase K (Roche) was added at a final concentration of 0.4 μg/ml, and samples were incubated at 55°C for 30 min. To selectively precipitate the DNA, ammonium acetate was added to 0.1 M followed by two volumes of cold 100% ethanol, mixing (Vortex), incubation at -80°C for 30 minutes, and centrifugation for 15 minutes at 16,100 × g (4°C). The supernatants were aspirated and the DNA pellets were washed with 70% ethanol and centrifuged again. The supernatants were removed, the pellets were dried, and the DNA was resuspended in TE.

Next the isolated DNA was labeled with aminoallyl-dUTP (aa-dUTP). The bead-beating with glass beads (previous paragraph) was sufficient to shear the DNA into fragments of ~500 bp, an appropriate size for random-primed labeling. Reactions containing 4 μg isolated genomic DNA, 10 μg random hexamers (MWG), and Klenow buffer (60 mM Tris-Cl, pH 7.0, 6.0 mM MgCl_2_, and 12 mM β-mercaptoethanol (Sigma)) were incubated at 100°C for 10 minutes, then quick-cooled in ice-water for 5 minutes. Then dNTPs were added (0.36 mM dATP, dGTP, dCTP (Invitrogen); 0.12 mM dTTP (Invitrogen), 0.24 mM aa-dUTP (Ambion) and 25 units of Klenow Fragment (3' → 5' exo-; New England Biolabs) were added. The final reactions were mixed briefly and incubated at 37°C overnight.

Labeled DNA was recovered using the Qiaquick PCR purification kit (Qiagen), following the manufacturer's protocol with the following modifications. The Qiagen-supplied phosphate wash and elution buffers contain free amines which compete with the Cy-dye coupling reaction, so these buffers were substituted by homemade buffers (phosphate wash buffer: 5 mM potassium phosphate, pH 8.5, 80% ethanol; elution buffer: 4 mM potassium phosphate, pH 8.5). After the labeled DNA was purified, it was pelleted and dried in a SpeedVac (Savant). Then the labeled DNA was coupled to either Cy3 or Cy5 (Amersham) by resuspension of the aa-dUTP-labeled DNA in 4.5 μl of 0.1 M Na_2_CO_3_, pH 9.0, with an equal volume of NHS-ester Cy-dye, followed by an hour incubation at room temperature. The control DNA, JLP1164 at time 0 hrs (when the cells were in G2 phase), was coupled to Cy5, and the experimental DNA was coupled to Cy3. Uncoupled dye was removed using the Qiaquick PCR purification kit (Qiagen), following the manufacturer's instructions.

For hybridizations, experimental DNA with 80 pmol Cy3 plus control DNA with 80 pmol Cy5 was resuspended in a hybridization solution consisting of 25% formamide, 5 × SSC, 0.1% SDS, and 100 μg/ml of sonicated salmon-sperm DNA. Hybridizations were performed under lifter cover slips (Erie Scientific) at 50°C in a humidified chamber for 16–20 hrs. Hybridized arrays were washed by gently shaking in the following solutions for the times and temperatures indicated: two quick washes with 2 × SSC/0.1% SDS at 50°C, two 10-min washes with 2 × SSC/0.1% SDS at 50°C, two 10-min washes with 0.1 × SSC/0.1% SDS at 50°C, and four quick washes with 0.1 × SSC at room temperature. Arrays were dried by centrifugation and scanned using an Axon 4000B scanner, controlled by GenePix Pro 6.0 software with a pixel size of 10 μm. Photomultiplier tube gains were subjectively adjusted during pre-scan to maximize effective dynamic range and to limit image saturation.

### Microarray data extraction and analysis

Data from microarray scans was extracted as previously described [55]. Background was subtracted from each signal. The experimental to control ratios (Cy3 to Cy5) were then normalized to a value of 1.0 for the genome average. For each point, the results from multiple (up to five) independent hybridizations were averaged. The final values are available online as Additional Files 4, 5, 6. We employed the version of the fission yeast genome that was available from the Sanger Centre in May, 2006 [57]. This version had stretches of 1000 N's inserted into the chromosomal sequences to fill in each of the five gaps between contigs that were present at that time. Graphs were prepared using Igor Pro 5 software (WaveMetrics).

To classify potential origin signals according to their apparent strengths, the following procedure was used. Since the locations of AT islands and pre-RCs were precisely specified by Segurado *et al. *[20] and Hayashi *et al. *[15], respectively, and since all AT islands and pre-RCs are located between genes, and our probes are mostly located within genes, we were able to evaluate AT islands and pre-RCs simply by examining the relative copy numbers of the two probes flanking each AT island or pre-RC at the 2- and 4-hr time points in three strains (wild-type, *cds1*Δ and *rad3*Δ). Thus we examined 12 different copy number values to classify each AT island or pre-RC. However, since we anticipated that the *rad3*Δ and *cds1*Δ strains should behave in the same way, we always used the lower of the two corresponding *rad3*Δ and *cds1*Δ values when classifying potential origins. Thus, if either a single wild-type value (at any of the 4 position/time combinations under consideration) or *both *of the *rad3*Δ and *cds1*Δ values at any of the 4 position/time combinations under consideration exceeded 1.5, *and *at least one other wild-type value or *rad3*Δ/*cds1*Δ combination exceeded 1.3, then the potential origin was classified as "strong". Similarly, if at least one wild-type value or a combination of *rad3*Δ and *cds1*Δ values exceeded 1.3 *and *at least one wild-type or one combination of *rad3*Δ and *cds1*Δ values exceeded 1.2, then the potential origin was classified as "medium". The "weak" classification required one value (wild-type or *rad3*Δ/*cds1*Δ combination) greater than 1.2 and *two *values greater than 1.1. Finally, "very weak" required only one value (wild-type or *rad3*Δ/*cds1*Δ combination) greater than 1.1. If none of the signals from probes flanking the AT island or pre-RC was greater than 1.1, then the AT island or pre-RC was classified as below limit and indicated by a "0" in Additional File 7 and Fig. 6. If both probes flanking the AT island or pre-RC seemed too far away (usually > 8 kb) to permit evaluation of the AT island or pre-RC, then the AT island or pre-RC was classified as ambiguous and indicated with a "?" in Additional File 7 and Fig. 6.

In contrast to AT islands and pre-RCs, the positions of origins in the studies of Feng *et al. *[34] were calculated by data smoothing using a 12-kb sliding window. For that reason, when evaluating putative origins identified by Feng *et al., *we took into consideration the copy number values of probes within 6 kb on either side of the predicted origin. In the case of origins identified by Heichinger *et al. *[14], we took into consideration the copy number values of all of our probes between the 5' gene and the 3' gene specified by Heichinger *et al. *as flanking their origins (see the Origin List provided by Heichinger *et al*. in their supplementary data). For both types of predicted origin, this procedure sometimes resulted in a larger number of values to be examined than for AT islands or pre-RCs. However, the criteria for classification were identical to those for AT islands and pre-RCs. For this reason, the origins predicted by Feng *et al. *and by Heichinger *et al. *may have achieved somewhat higher classifications than AT islands or pre-RCs of equivalent strengths. While scanning the chromosomes, we also noted 22 positions where our data strongly suggested the presence of an origin, even though no origin had been mapped to that site by other laboratories. We included these additional potential origins in our analyses, and they were classified by the same criteria.

### Flow cytometry

Cells were fixed in 70% ethanol and stored at 4°C until needed. For flow cytometry, cells were resuspended in 0.1 M HCl containing 2 mg/ml pepsin (Sigma) and incubated for one hour at room temperature in order to eliminate possible cell-end staining by Sytox Green. Then cells were washed once in 50 mM sodium citrate, pH 7.0, resuspended in 50 mM sodium citrate, pH 7.0, supplemented with 500 μg/ml of RNaseA (Sigma), and incubated for two hours at 37°C. Cells were next stained in 50 mM sodium citrate, pH 7.0, supplemented with 1 μM Sytox Green (Molecular Probes) and immediately analyzed on a FACScan flow cytometer (Becton Dickinson).

### Two-dimensional analysis of potential origins

For Fig. 7A, log-phase wild-type (strain 975; [56]) cells were incubated with 25 mM HU for 3 hours at 30°C. Then the HU was removed and incubation was continued for the times indicated in Fig. 7A. Samples were removed at these times and prepared for 2D gel electrophoresis as previously described [18]. For Fig. 9, the same strains used for the microarray experiments (JLP1164, JLP1257, JLP1260) were synchronized by temperature-block-and-release into medium containing 15 mM HU in the same manner as for the microarray experiments. Samples were removed at the indicated times and prepared for gel electrophoresis as previously described [18].

## Authors' contributions

KLM helped plan the experiments, carried out most of the microarray experiments and analyses, and contributed to writing the manuscript. SR carried out some 2D gel experiments and contributed to writing the manuscript. AR and AO made the arrays and helped plan and establish microarray-based copy-number detection for this study, and they helped plan some of the experiments. AO carried out some of the microarray experiments, and AR contributed to statistical analyses of the microarray data. AC, CY and DS all carried out some of the 2D gel experiments. JL and JAH helped plan the experiments and analyses and contributed to writing the manuscript. JAH carried out most of the analyses of individual origins as well as the analyses integrating data from multiple studies. All authors read and approved the final manuscript.

## Supplementary Material

Additional file 1 Graphs of microarray measurements of copy number changes throughout chromosome 1. A multi-page PDF file, with graphs for chromosome 1 based on the data in Additional File 4. The results are shown at 150 kb per page, with 10-kb overlaps between pages. The symbols are explained in the legend to Figure 2. Click here for file

Additional file 2 Graphs of microarray measurements of copy number changes throughout chromosome 2. Similar to additional file 1, but for chromosome 2 Click here for file

Additional file 3 Graphs of microarray measurements of copy number changes throughout chromosome 3. Similar to additional file 1, but for chromosome 3 Click here for file

Additional file 4 Microarray measurements of copy number changes throughout chromosome 1. In this table, the column "Probe Center" shows the positions, on the fission yeast nucleotide sequence of May, 2006, of the centers of all our PCR probes. Since the telomere sequences detected by our "telomere" probe are beyond the range of the sequenced genome, they are arbitrarily shown in these tables at positions -10,000 (at the left ends of chromosomes 1 and 2) and at positions equivalent to 10,000 bp beyond the ends of the sequenced chromosomes at the right ends of chromosomes 1 and 2. The next column, "Gene Name", shows the names of the probes. In most cases, these are the names of the ORFs containing the probes. Most probes were located in the 3' ends of ORFs. The columns headed "AVG_XXXX_Yhr_15 mM" (where XXXX and Y are numbers) show the normalized, averaged relative copy numbers for the indicated probe in strain XXXX at Y hours after release from the temperature block in the presence of 15 mM HU. The three strains are JLP1164 (wild type), JLP1257 (*cds1*Δ), and JLP1260 (*rad3*Δ). The relative copy numbers shown here are plotted in Additional File 1 (for chromosome 1). Click here for file

Additional file 5 Microarray measurements of copy number changes throughout chromosome 2. Similar to Additional File 4, but for chromosome 2 Click here for file

Additional file 6 Microarray measurements of copy number changes throughout chromosome 3. Similar to Additional File 4, but for chromosome 3 Click here for file

Additional file 7 Classification, based on our microarray results, of putative origins throughout the fission yeast genome. For each evaluated origin, the first two columns in this table show the start (left side) and end (right side) of the positions of the PCR probes (see Additional Files 4, 5, 6) located in the genes flanking the origin. The next two columns show the names of those two genes. In the case of AT islands and pre-RCs, these two genes are adjacent to each other, but in the case of origins identified by us, by Feng *et al. *[34], or by Heichinger *et al. *[14] the two genes may be separated from each other by several intervening genes. When the origin being evaluated in a given row is an AT island, the next two columns show the name of the AT island (according to [20]) and its functional classification (see Methods). The following two columns show the classifications of origins if identified by Feng *et al. *in wild-type or in *cds1*Δ cells. For origins identified by Heichinger *et al.*, the following two columns show the Ori number assigned to the origin and the efficiency of the origin during a mitotic S phase as evaluated by Heichinger *et al. *[14]. For pre-RCs identified by Hayashi *et al. *[15], the next two columns show the number of the pre-RC and whether the pre-RC was scored by Hayashi *et al. *as strong/early (1) or weak/late (0). The next column shows our (Mickle *et al.*) classification of the origin. The penultimate column contains a "1" if the origin had significantly greater activity in checkpoint-mutant cells than in wild-type cells or a "0" if it did not. Similarly, the last column contains a "1" if the origin had significantly greater activity in wild-type cells than in checkpoint-mutant cells or a "0" if it did not. Click here for file

Additional file 8 Distributions on chromosomes of putative origins of various classifications. This table summarizes some of the results from Additional File 7. The columns show the numbers and percentages for various classifications of origins in the individual chromosomes and in the whole genome. The various types and classifications of origins are listed along the left side of the table. The bottom two rows were obtained by summing the "1" entries in the rightmost two columns of Additional File 7. Click here for file

Additional file 9 Overlaps between various definitions of origins. This table is similar to Additional File 8. However, the values shown are the numbers and percentages of published origins that were identified by two or more independent sets of criteria. For example, the uppermost set of values, under the heading "Overlap between Segurado-AT-Islands and Feng-WT-Oris", consists of the numbers and percentages of putative origins that were identified both as AT islands [20]* and *as origins in wild-type cells, on the basis of generation of single-stranded regions in HU-blocked cells [34]. Click here for file

Additional file 10 Comparison of evaluations of origin function by microarray and by 2D gel analysis. This table lists the putative origins that have been evaluated both by 2D gel analysis and by microarray analysis. For each such origin, the start and end positions, the start and end genes, the AT number, and our functional classification are shown, as in Additional File 7. The "2D gel bubble arcs" column indicates whether the 2D gel test revealed bubble arcs in a restriction fragment centered on the origin (Yes) or not (No). The "Comment" column specifies whether the indicated origin is discussed in the main text or in an additional file. The "Reference" column indicates the publication describing the 2D gel evaluation of the indicated potential origin: **(a) **Segurado M, de Luis A, Antequera F (2003) EMBO reports 4: 1048–1053. **(b) **Segurado M, Gomez M, Antequera F (2002) Mol Cell 10: 907–916. **(c) **Gomez M, Antequera F (1999) EMBO J 18: 5683–5690. **(d) **Okuno Y, Okazaki T, Masukata H (1997) Nucleic Acids Res 25: 530–536. **(e) **Kim SM, Huberman JA (2001) EMBO J 20: 6115–6126. **(f) **Sanchez JA, Kim SM, Huberman JA (1998) Exp Cell Res 238: 220–230. **(g) **Dubey DD, Zhu J, Carlson DL, Sharma K, Huberman JA (1994) EMBO J 13: 3638–3647. **(h) **Sunita Ramanathan and Joel A. Huberman, unpublished results. **(i) **Dubey DD, Srivastava VK, Pratihar AS, Yadava MP (2007) Submitted for publication (personal communication from DD Dubey). Click here for file

Additional file 11 Microarray signals at two AT islands that do not appear to serve as replication origins according to previous 2D gel electrophoretic studies. Discussion of apparent disagreement between 2D gels and microarrays at AT1156 and AT2103 Click here for file

Additional file 12 Microarray analysis of two late-replicating, weak origins. Discussion of the microarray results for *ars2-2 *and Telomere-Associated Sequences Click here for file

Additional file 13 Extensive correspondence between origins detected by microarrays and molecular combing analyses. Comparison of our microarray results with the molecular combing results of Patel *et al. *[13] Click here for file

Additional file 14 Examples of checkpoint-restrained and checkpoint-dependent origins. Graphs of microarray results for a checkpoint-restrained and a checkpoint-dependent origin Click here for file
