# Supplementary figures and images for: Checkpoint independence of most DNA replication origins in fission yeast
Source: BMC Mol Biol. 2007 Dec 19;8:112. doi: 10.1186/1471-2199-8-112 (PMC2235891; doi:10.1186/1471-2199-8-112)

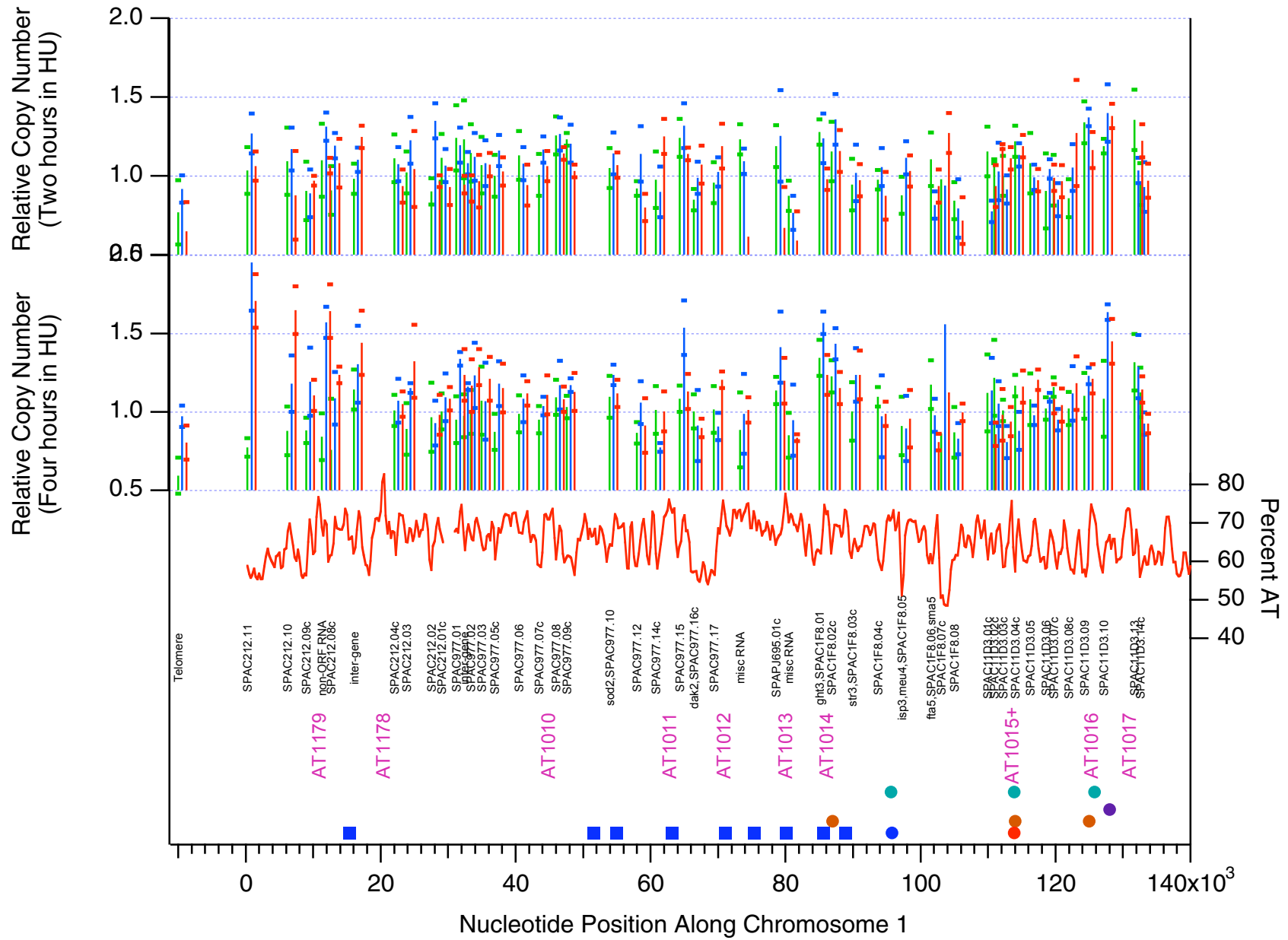

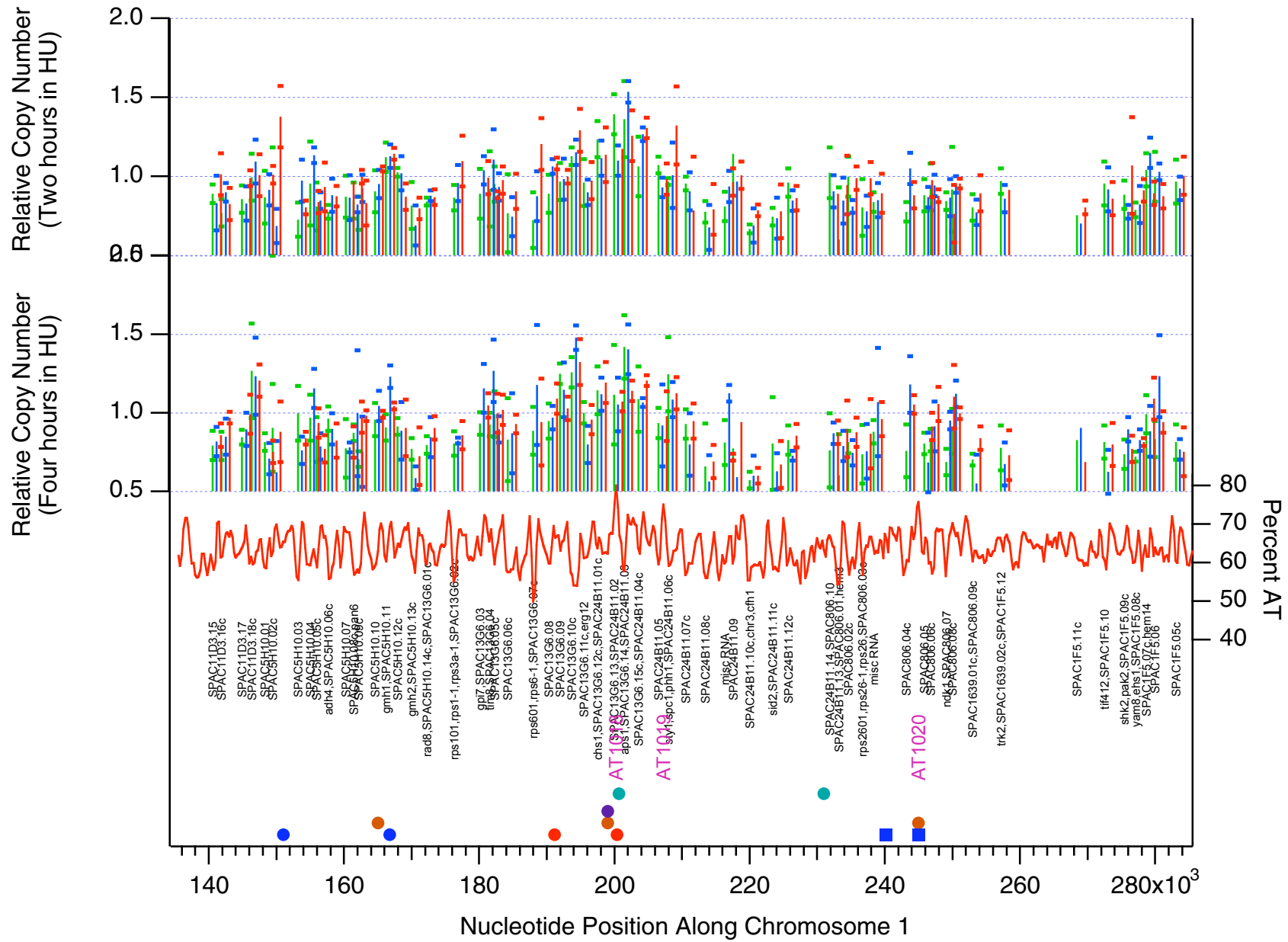

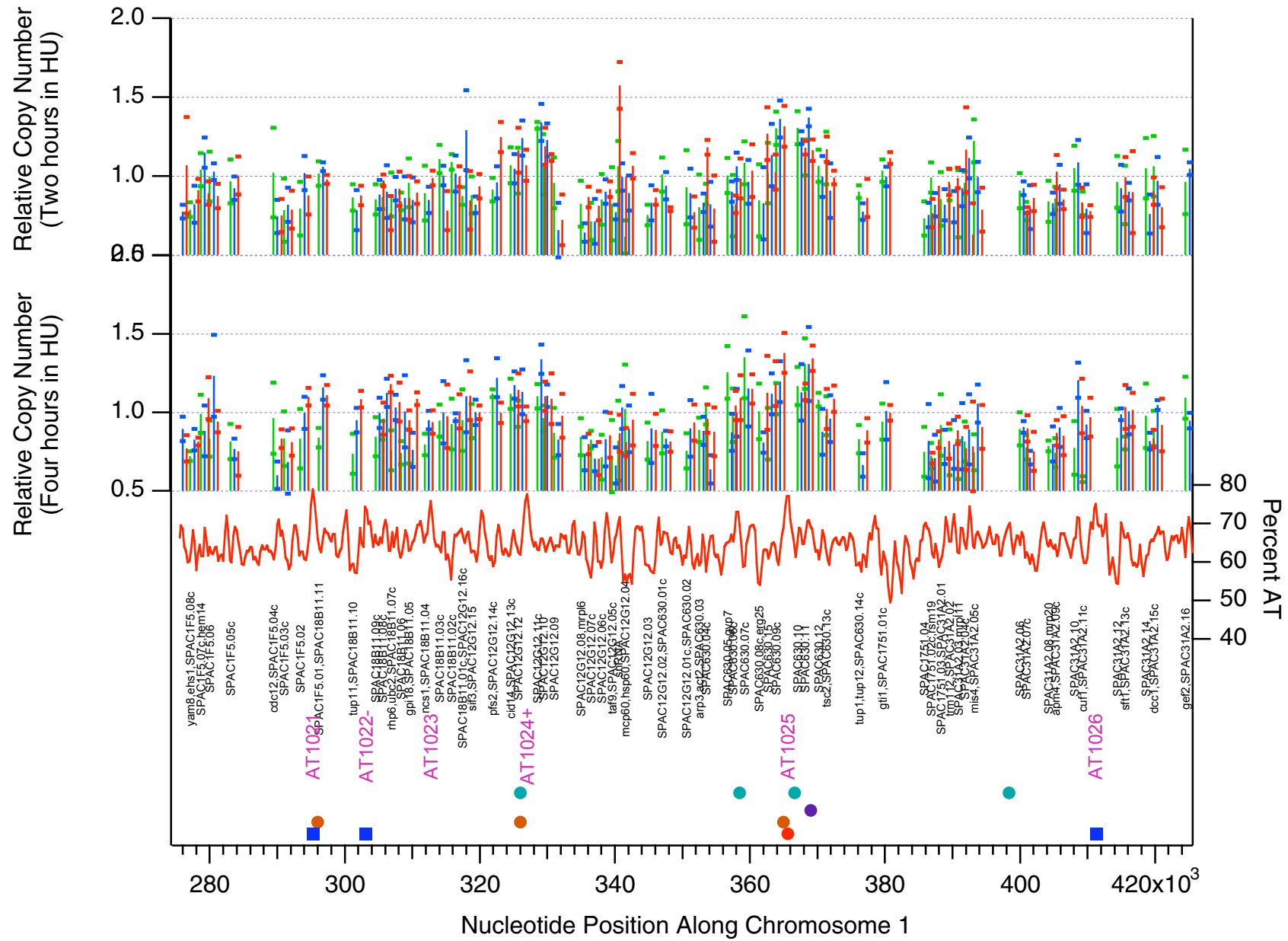

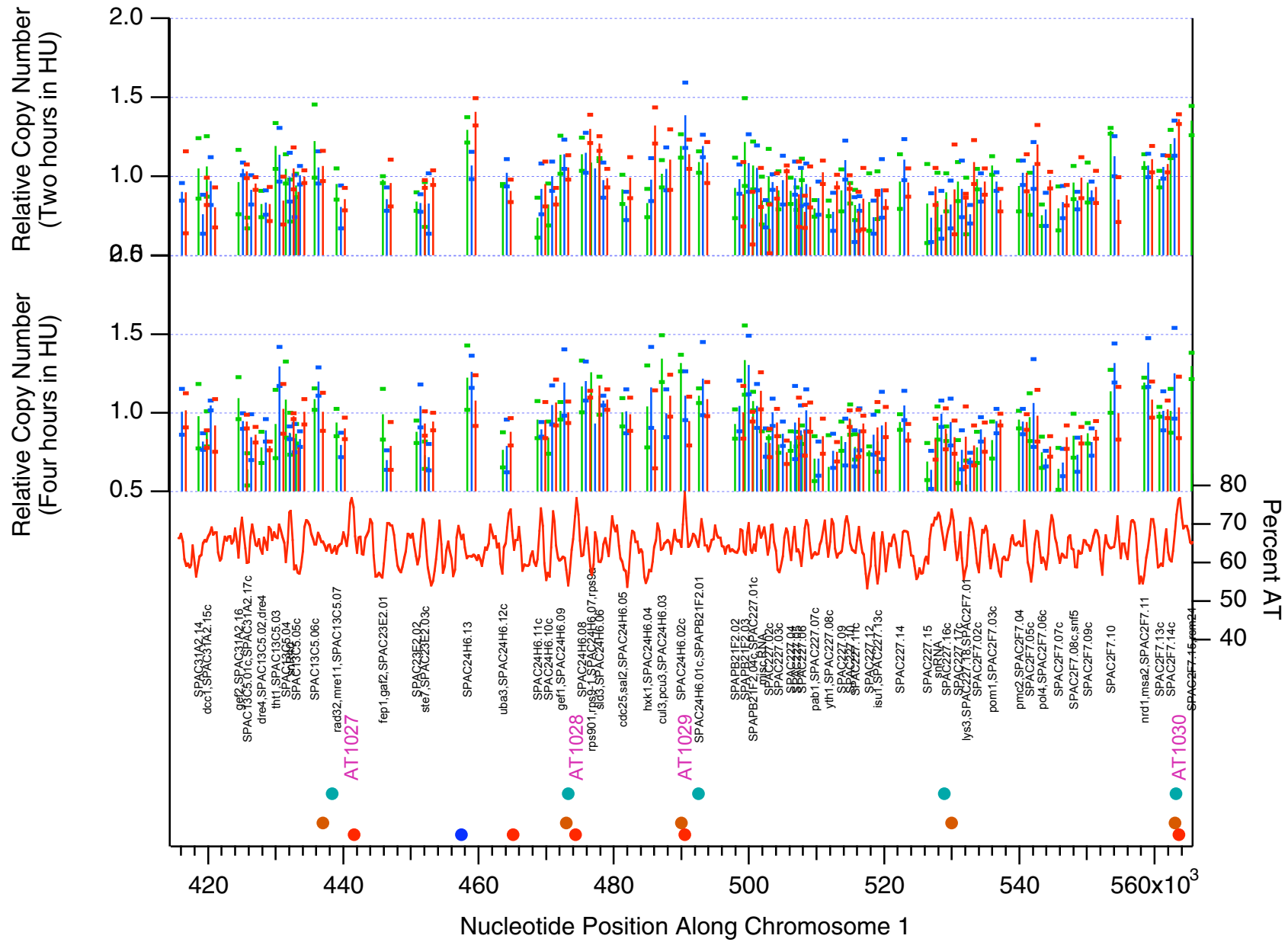

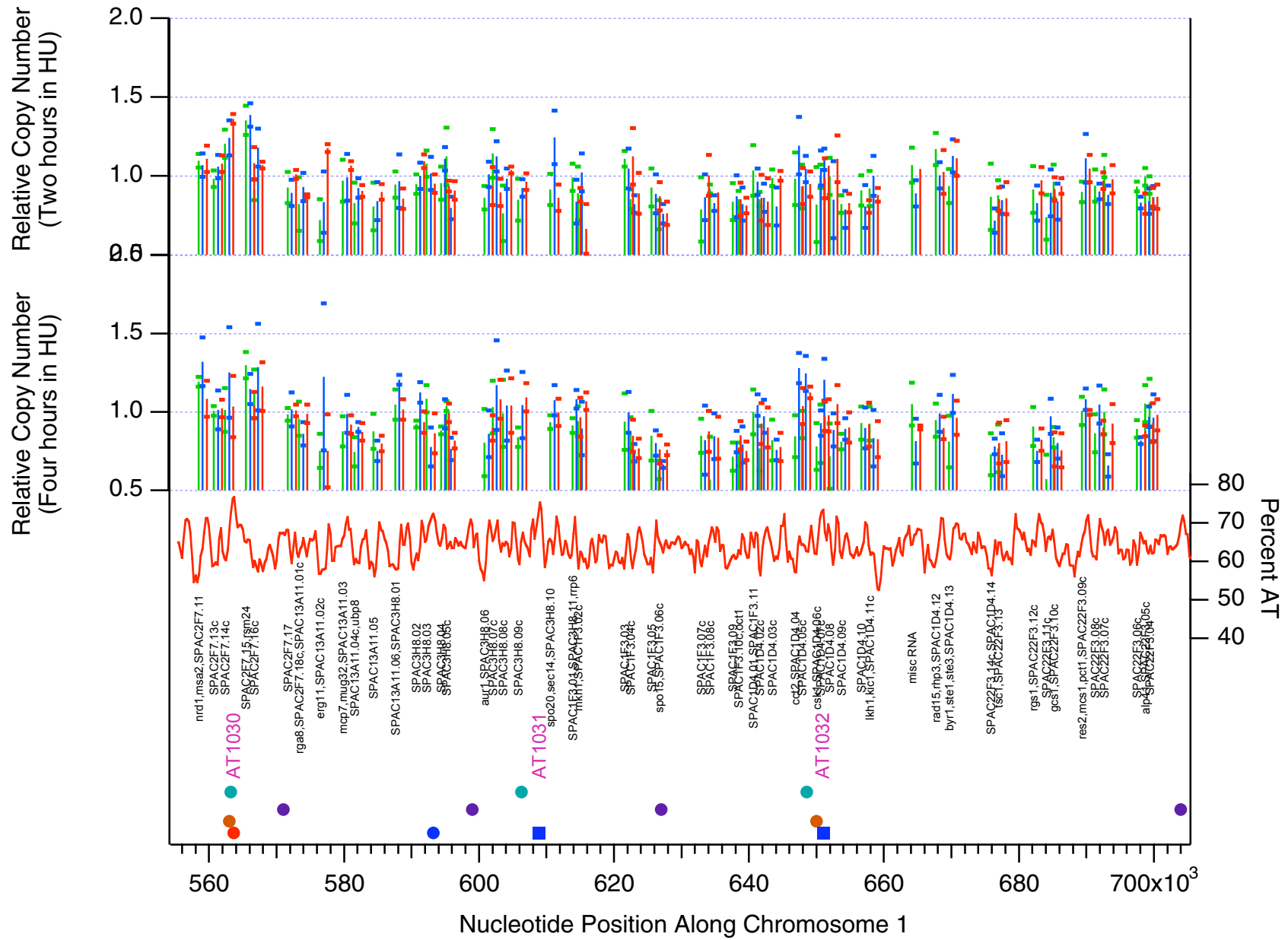

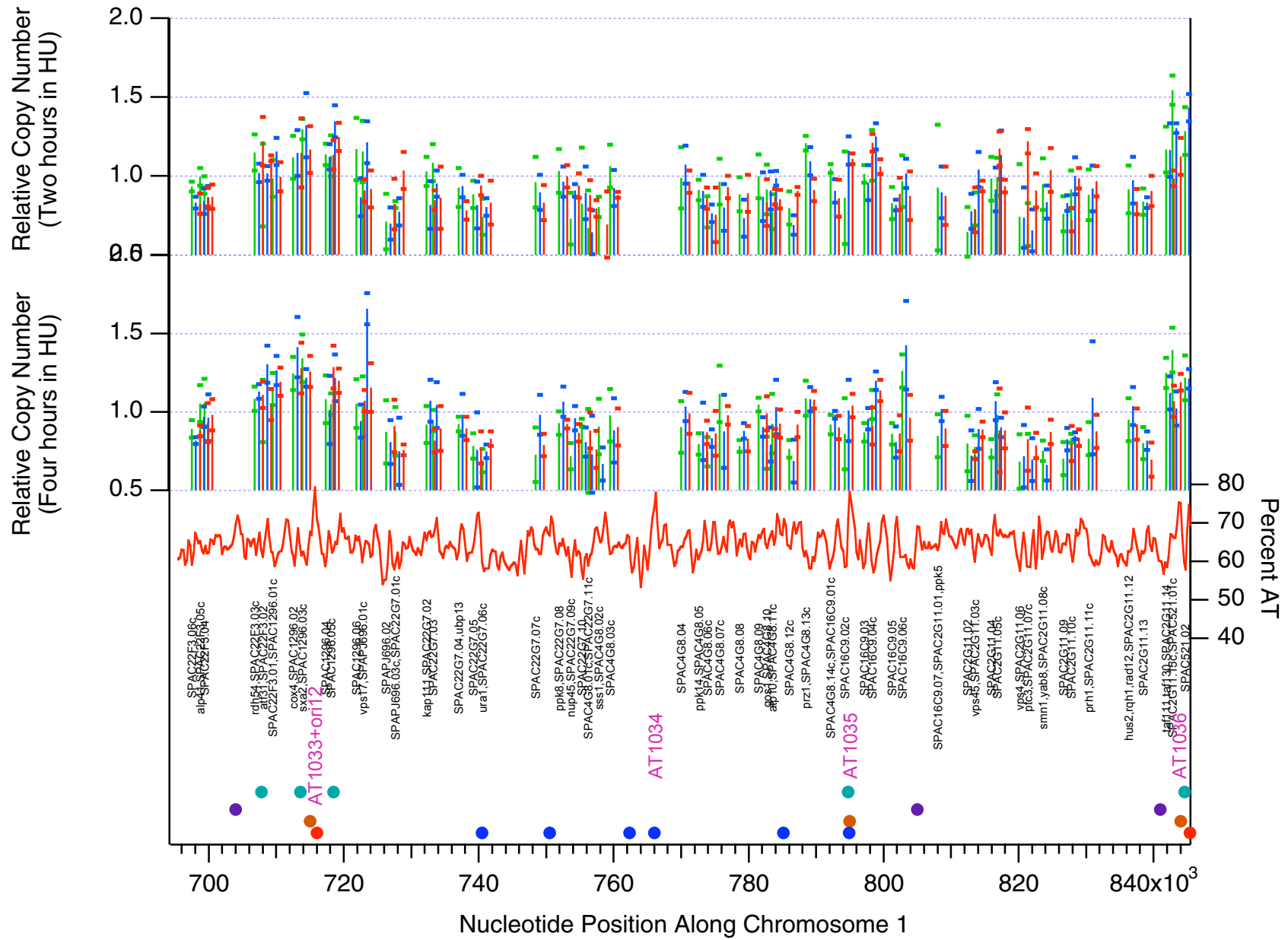

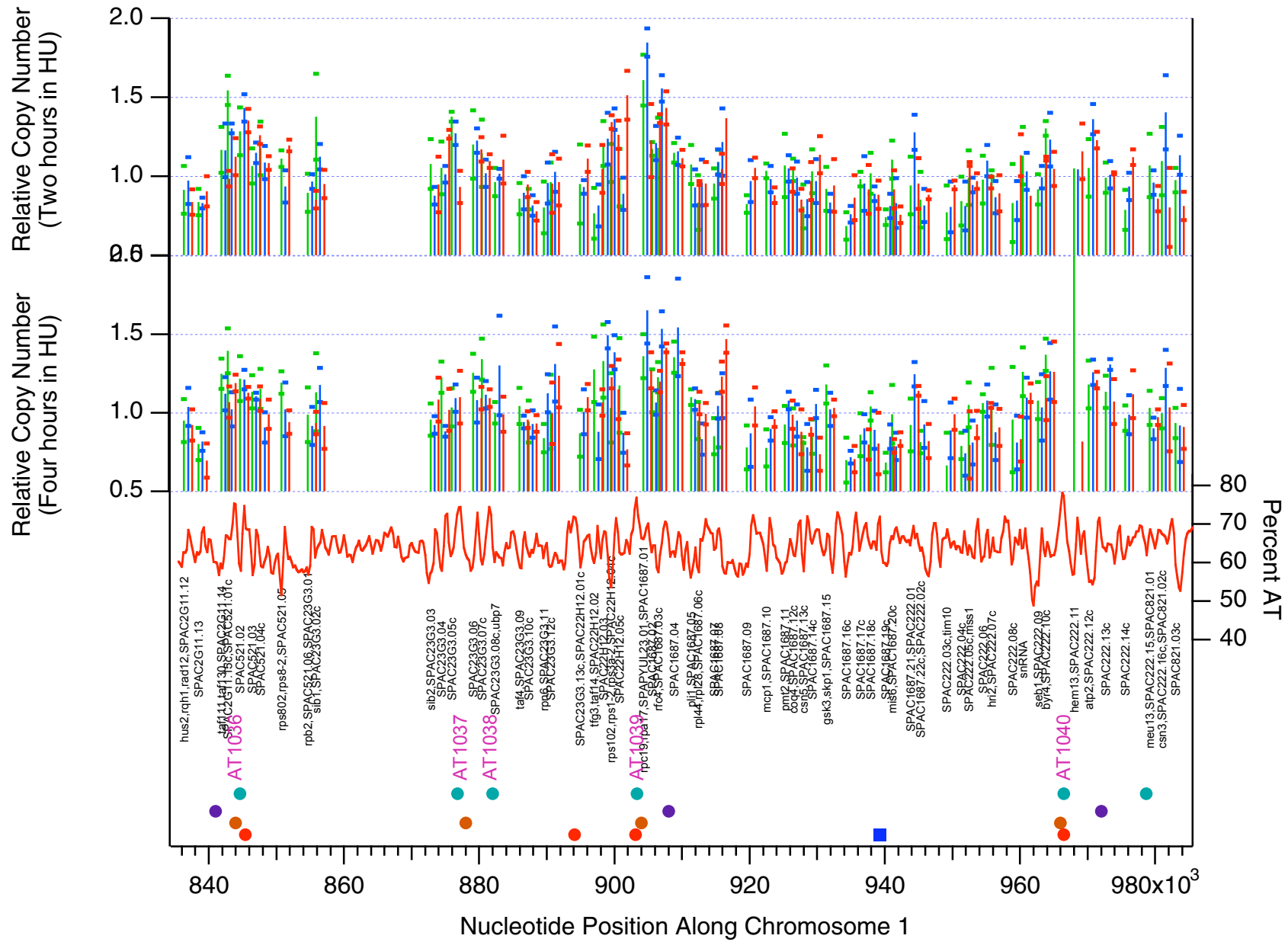

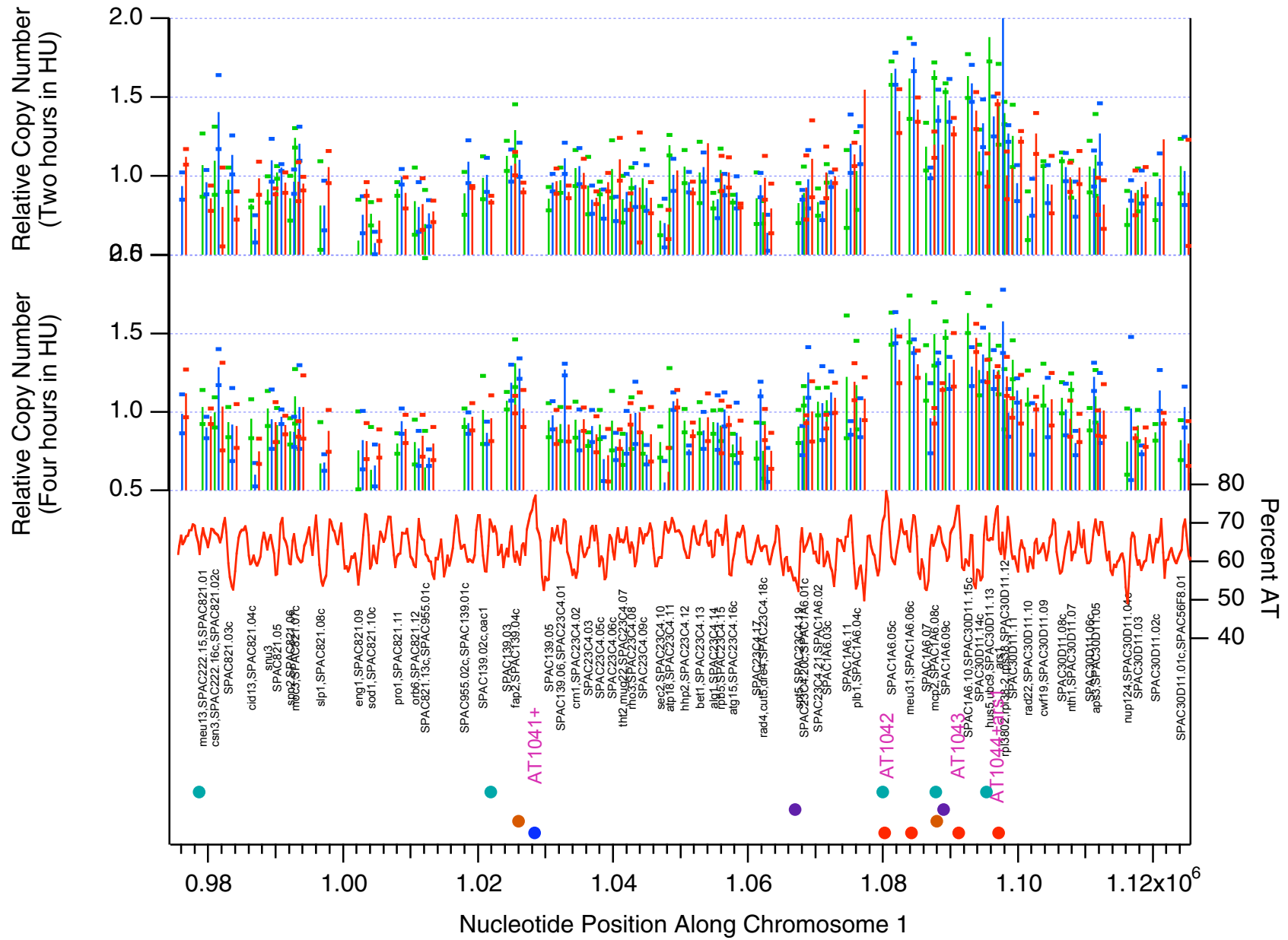

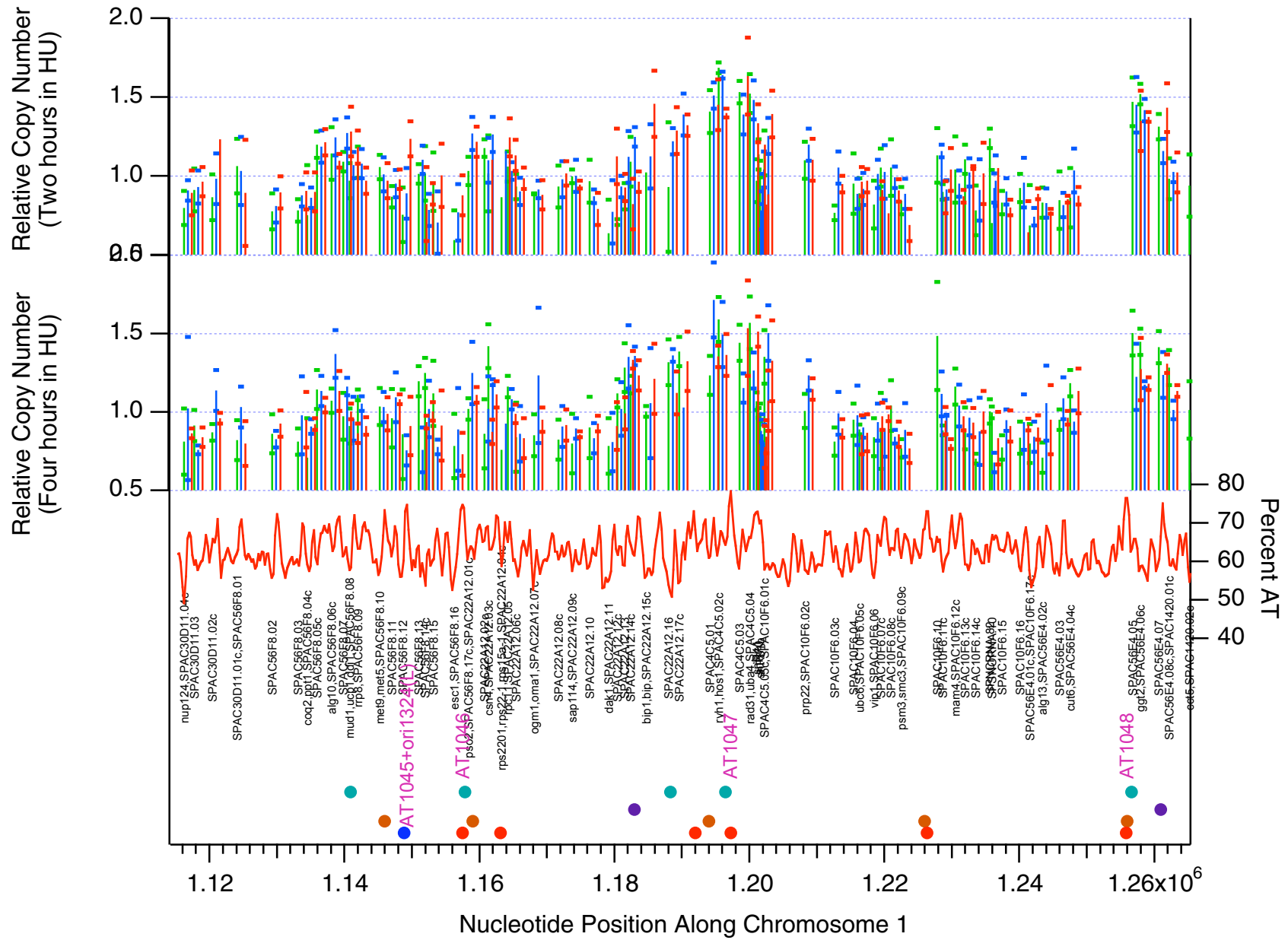

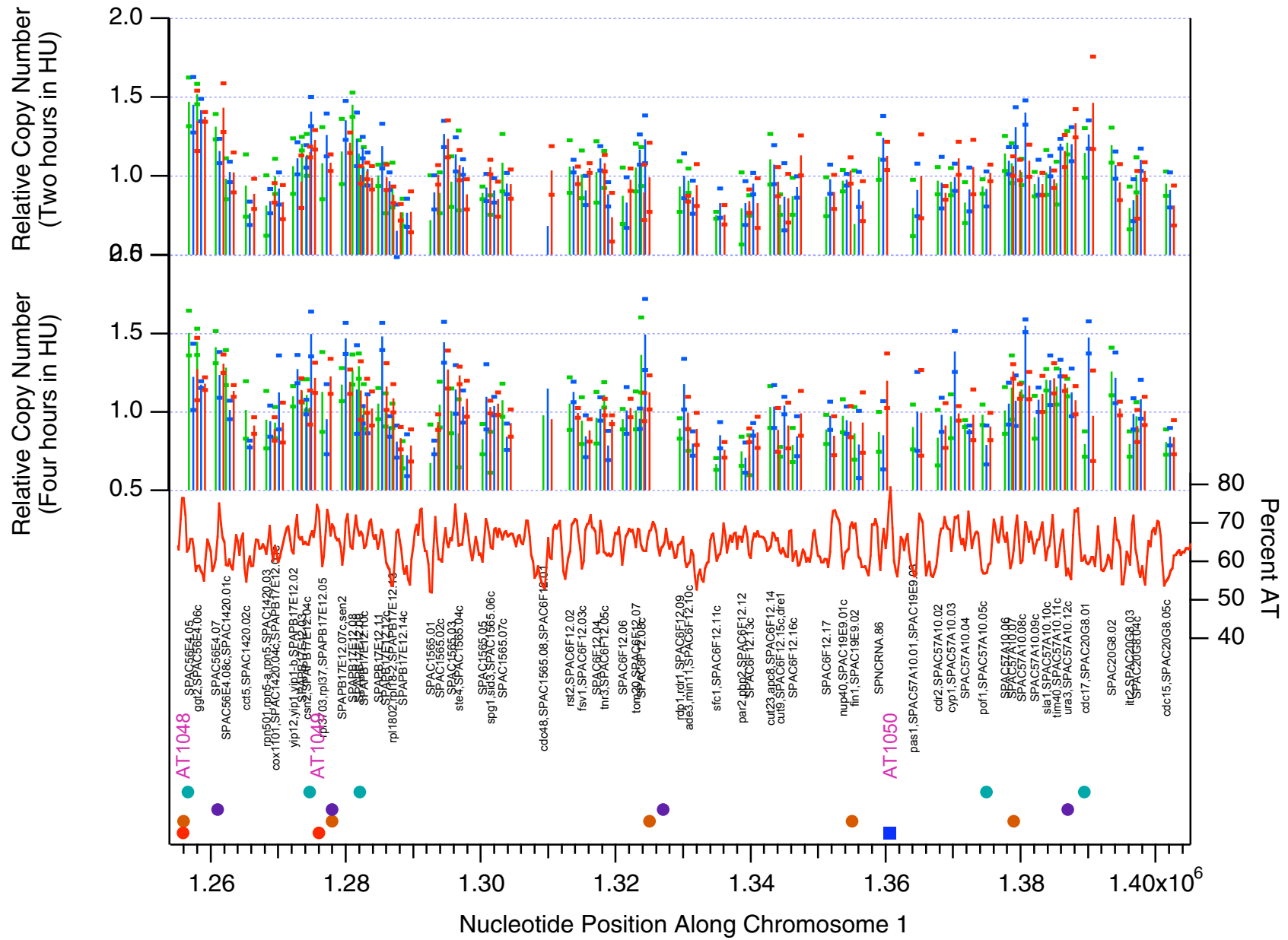

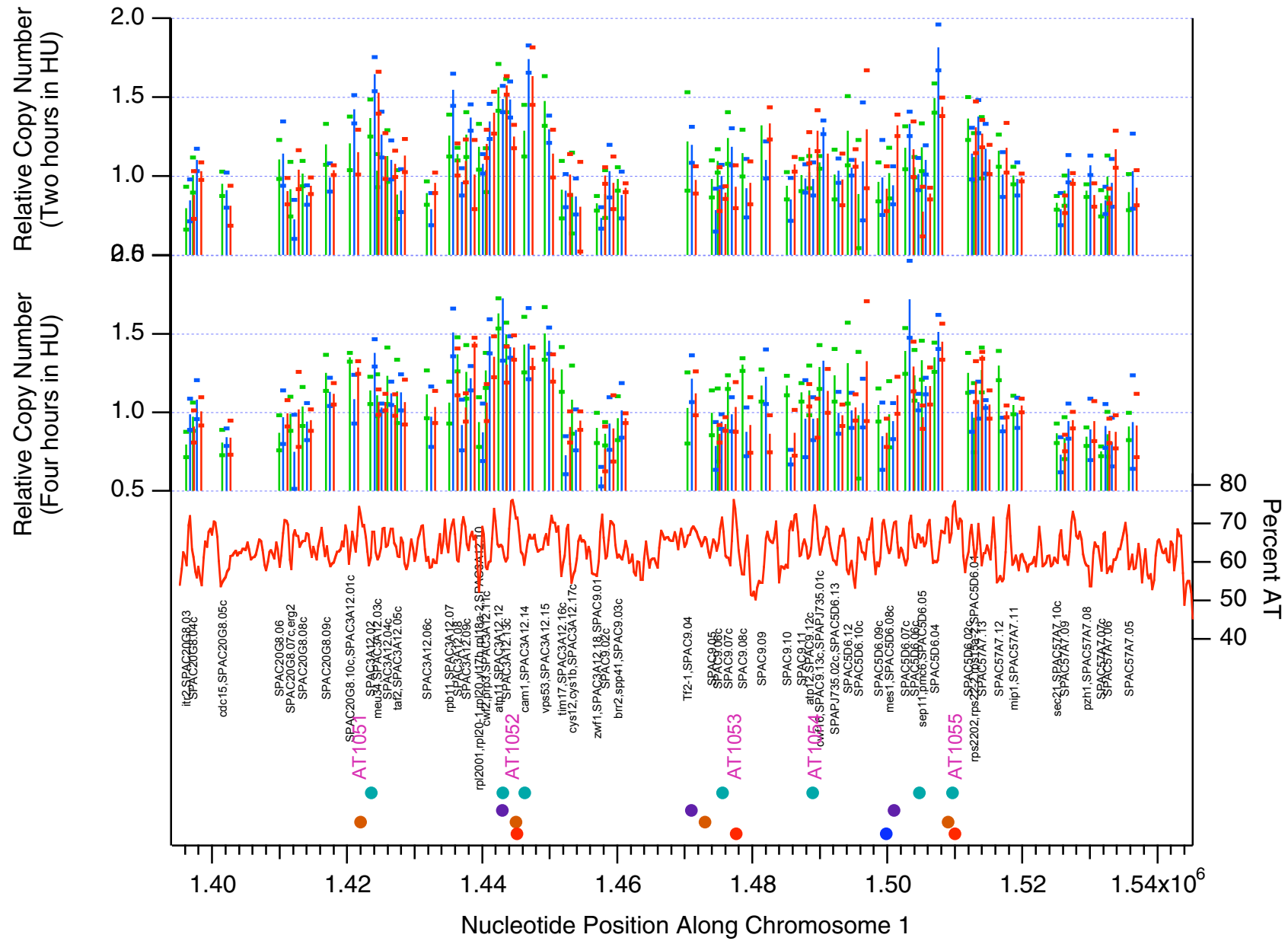

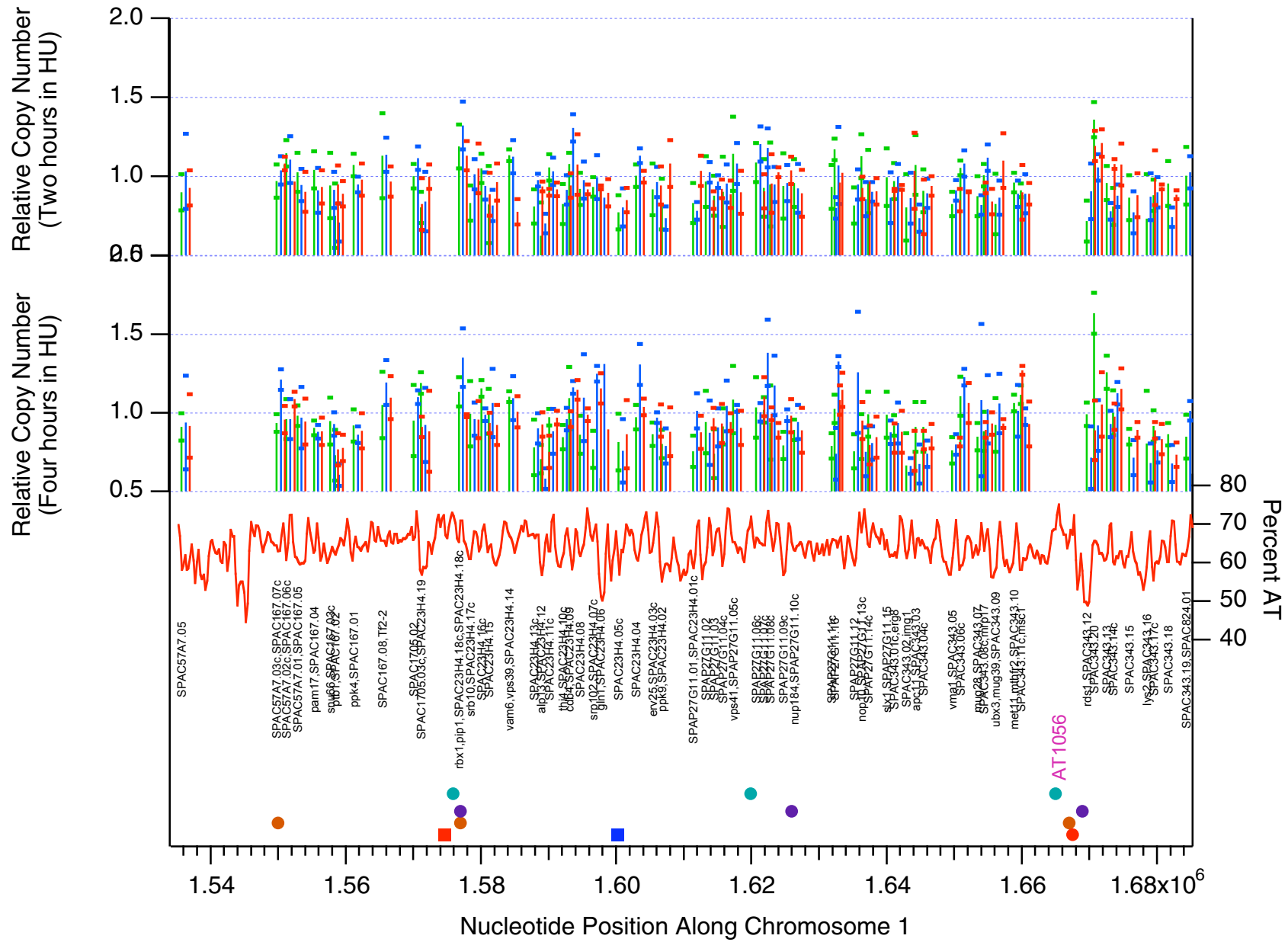

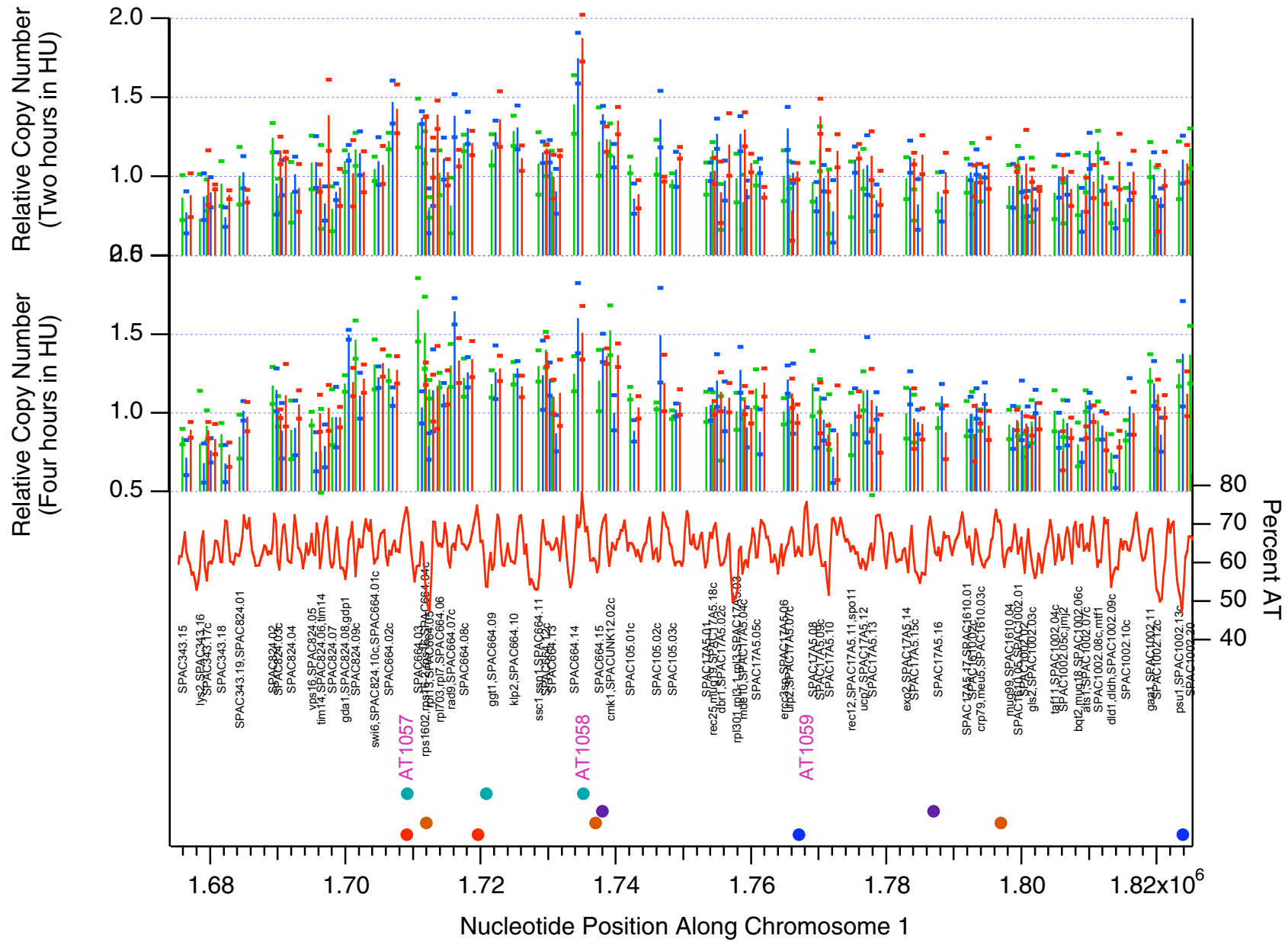

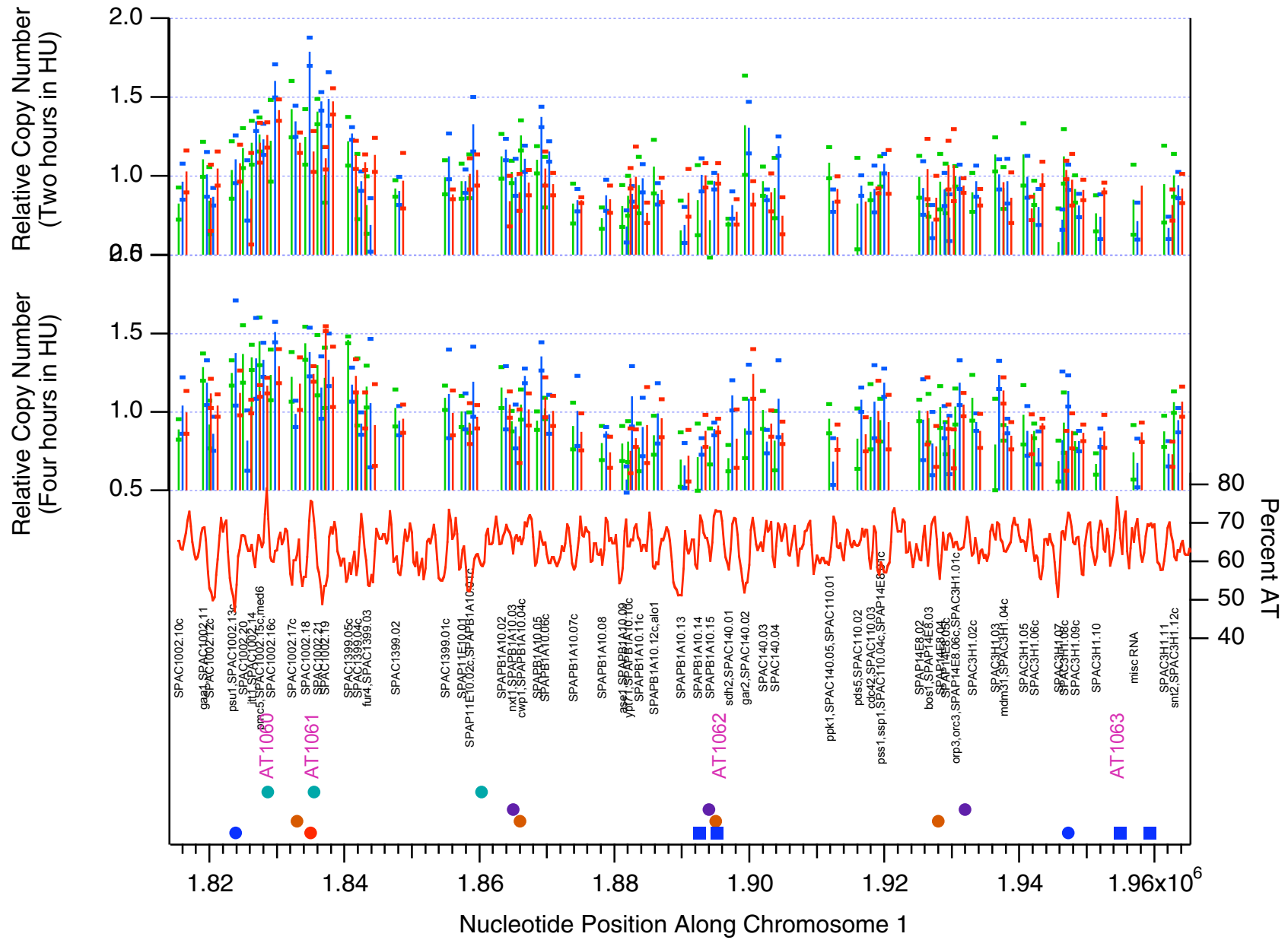

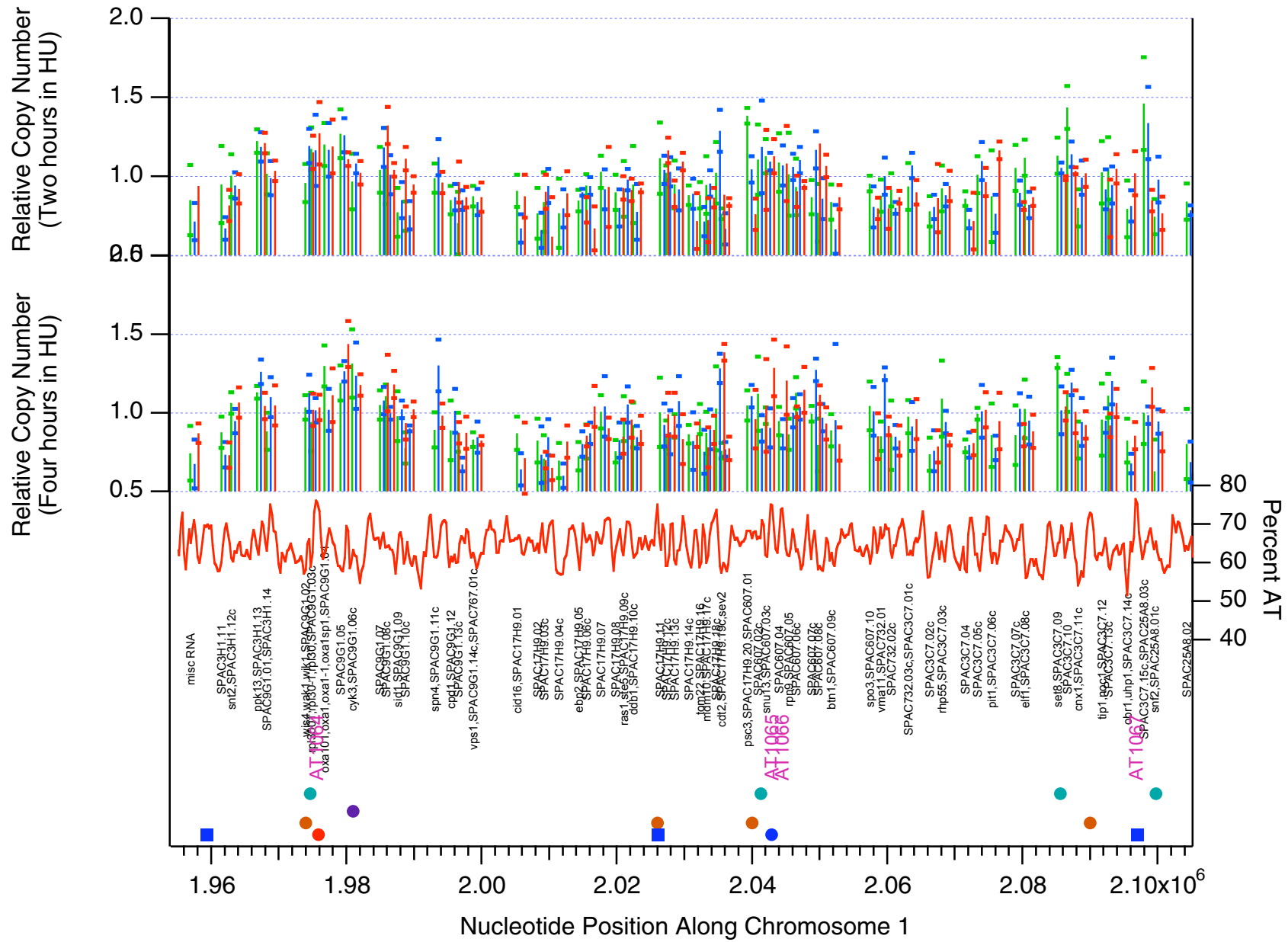

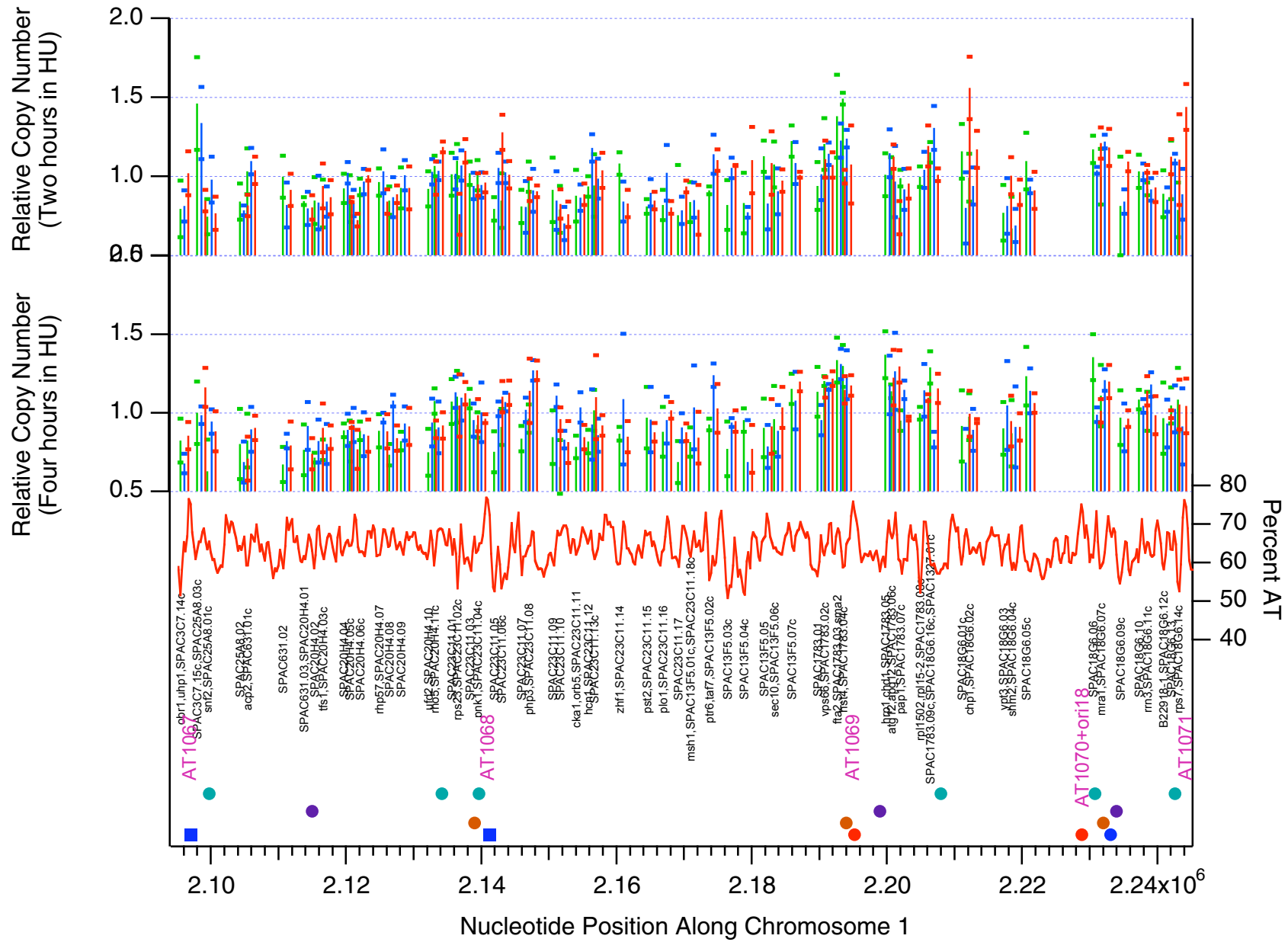

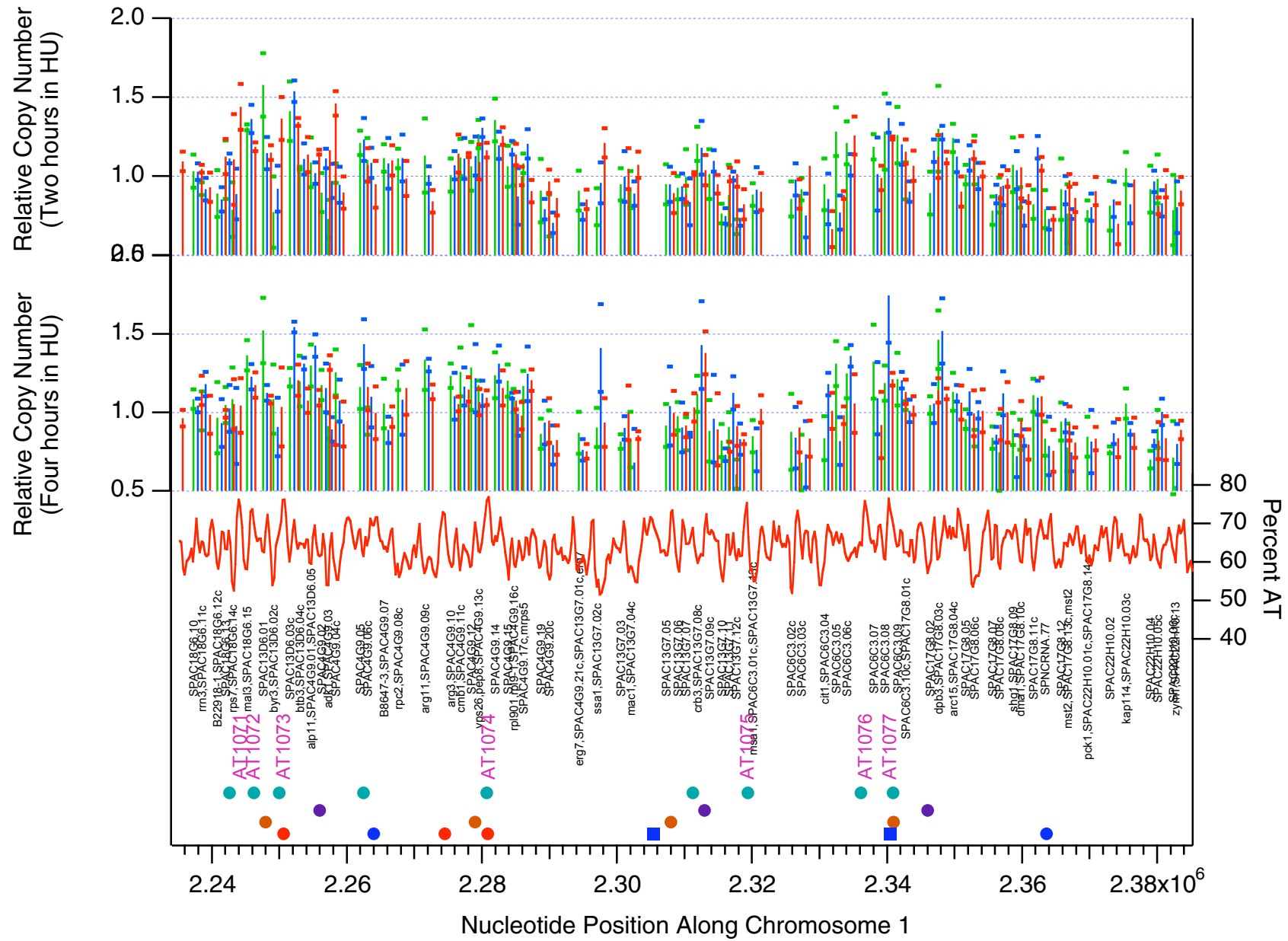

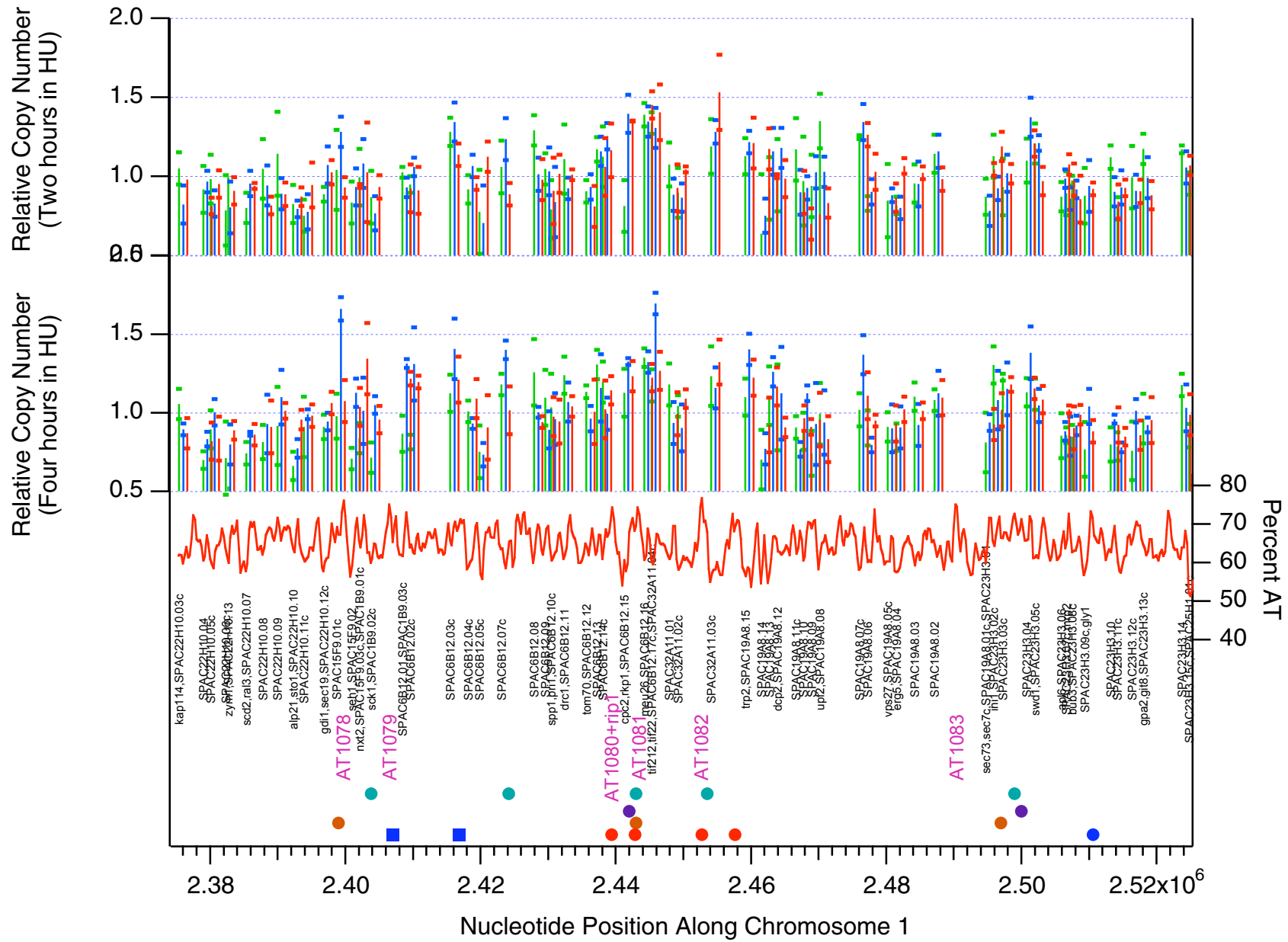

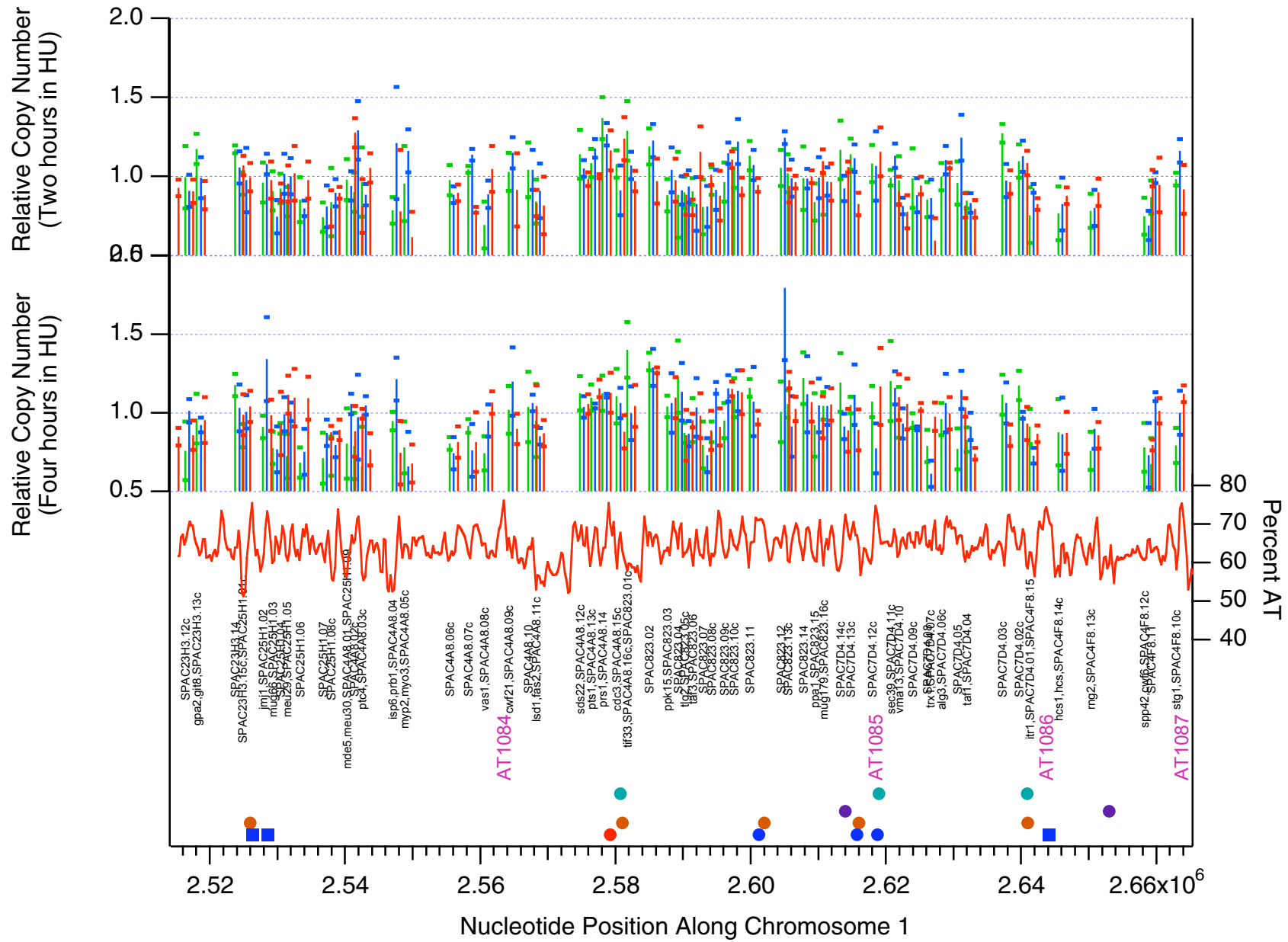

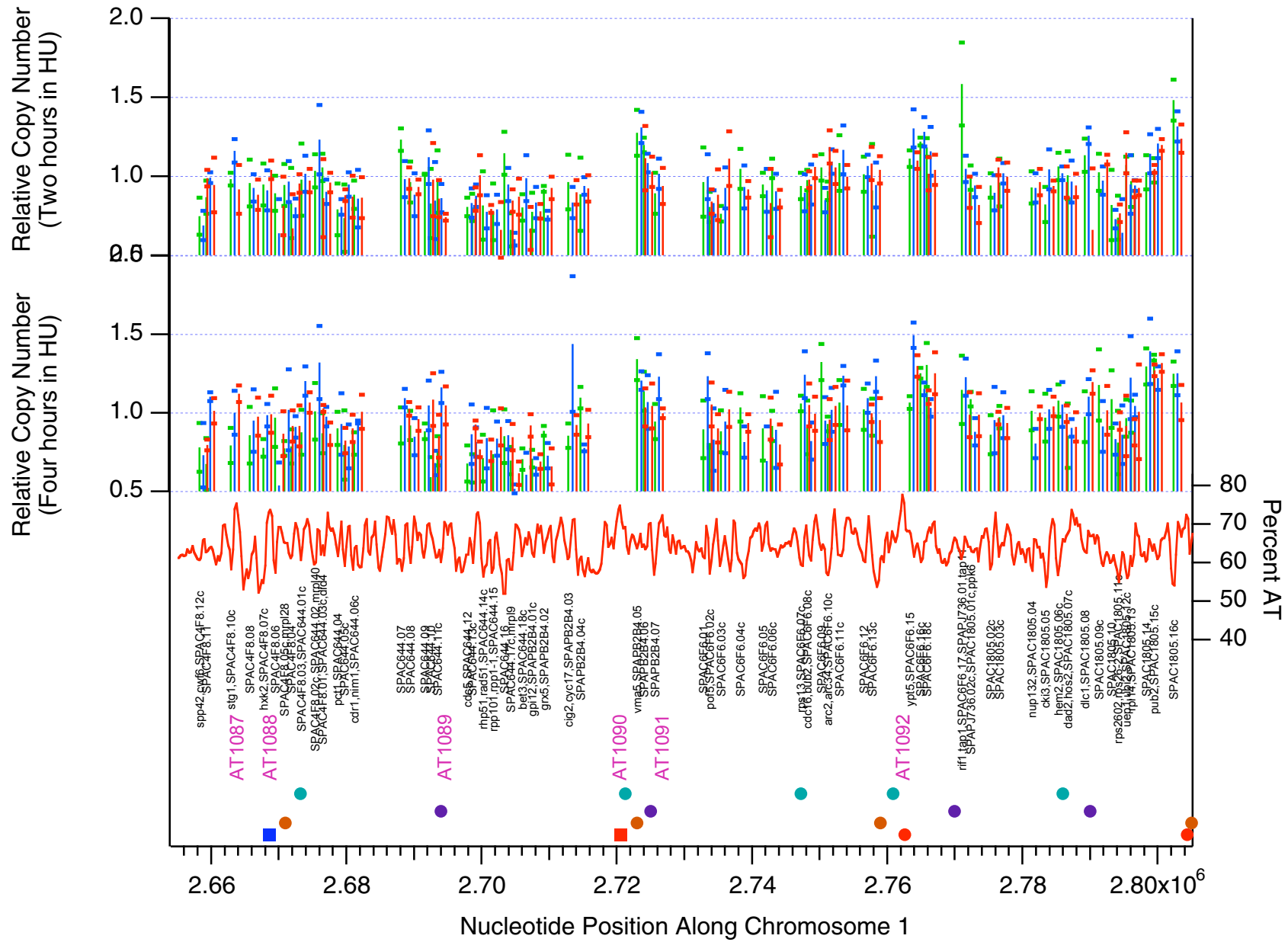

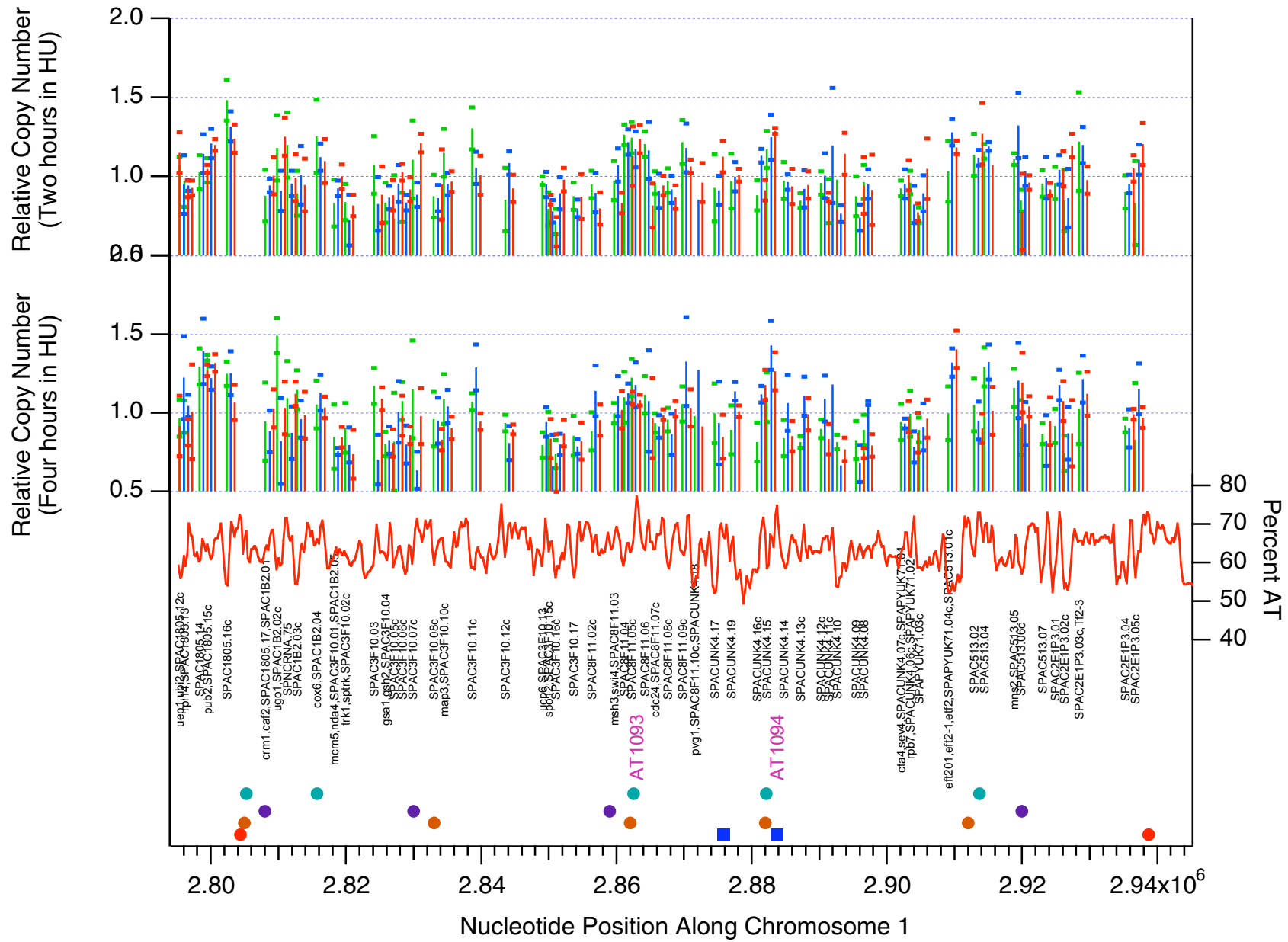

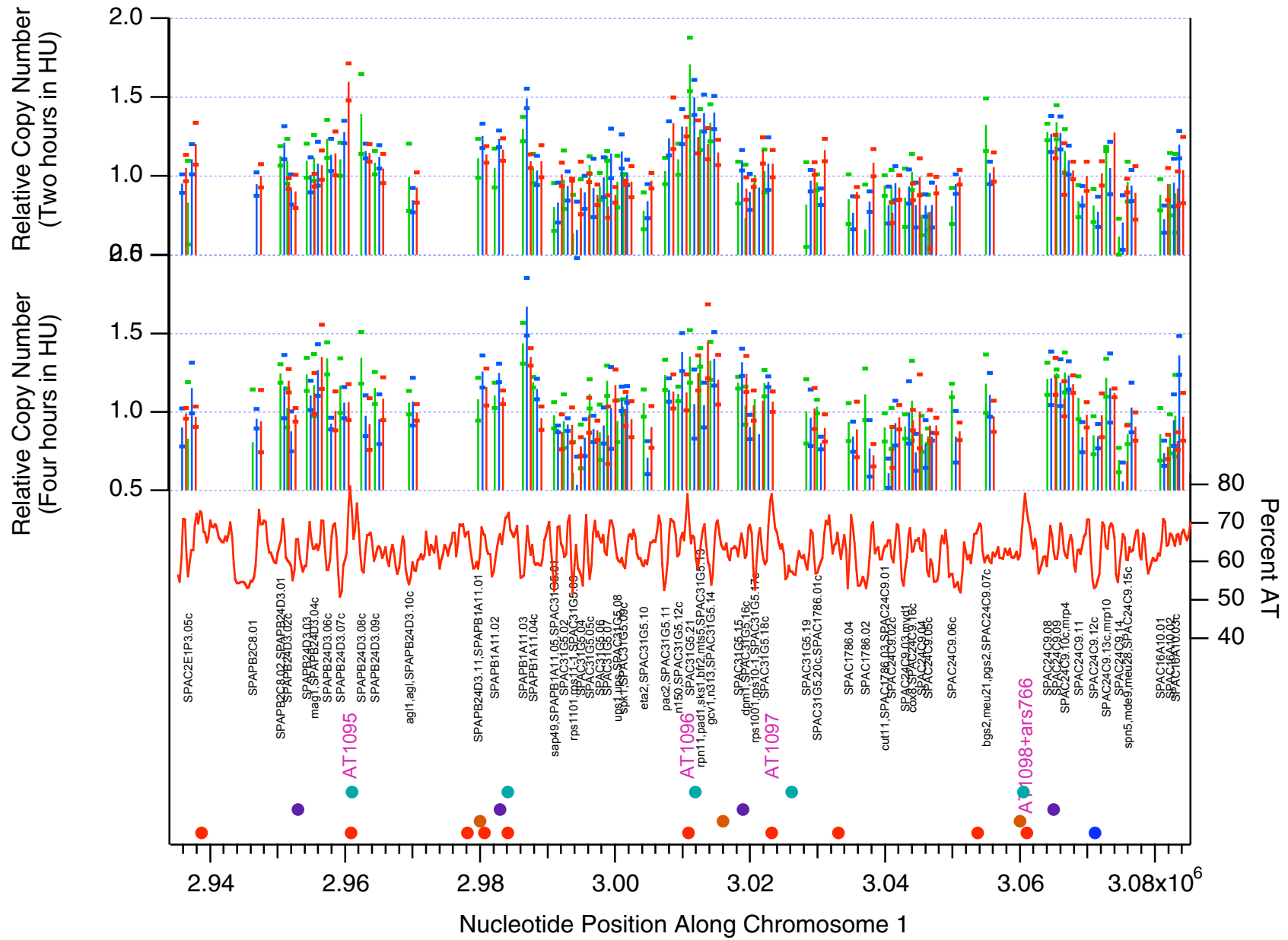

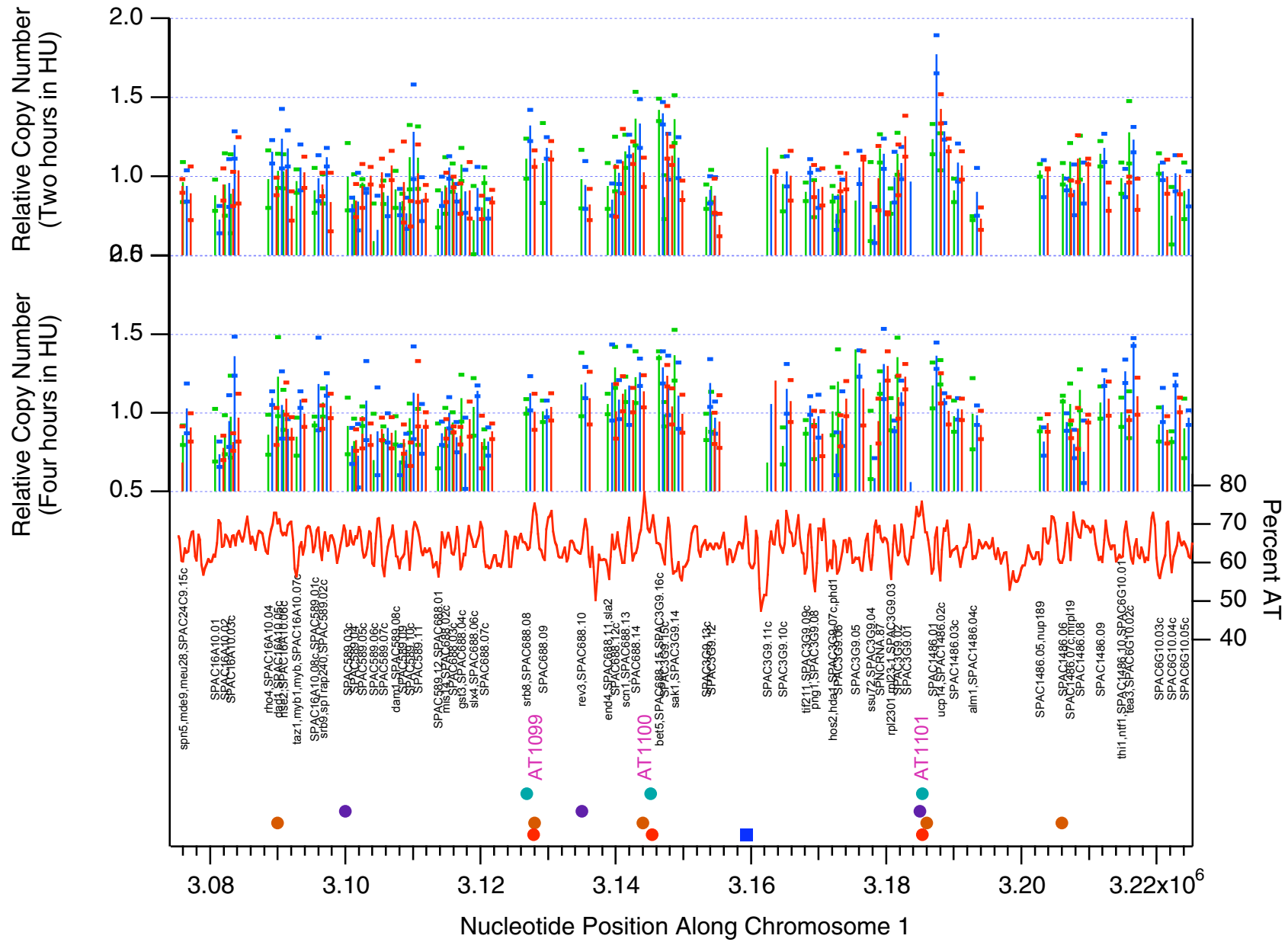

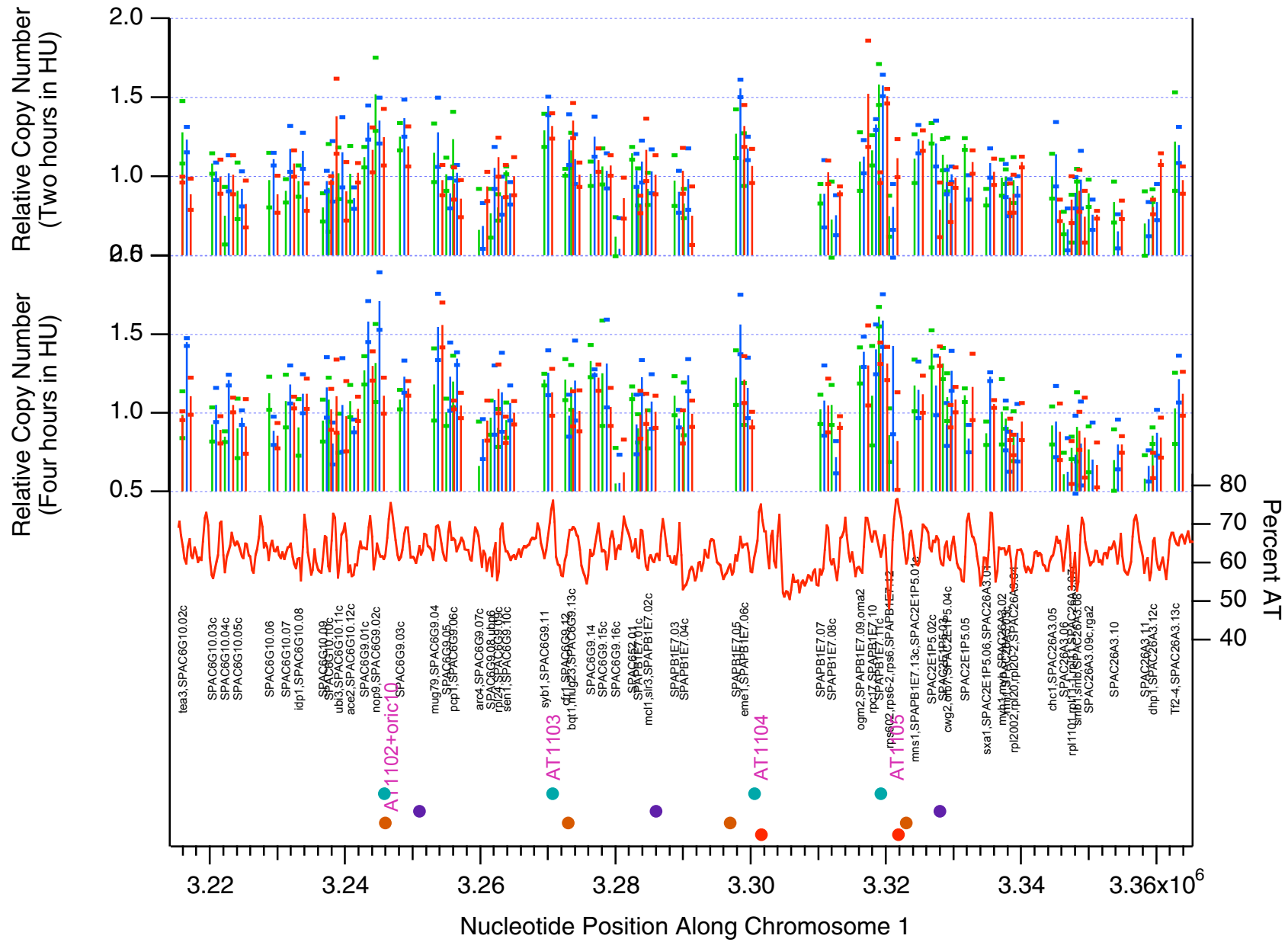

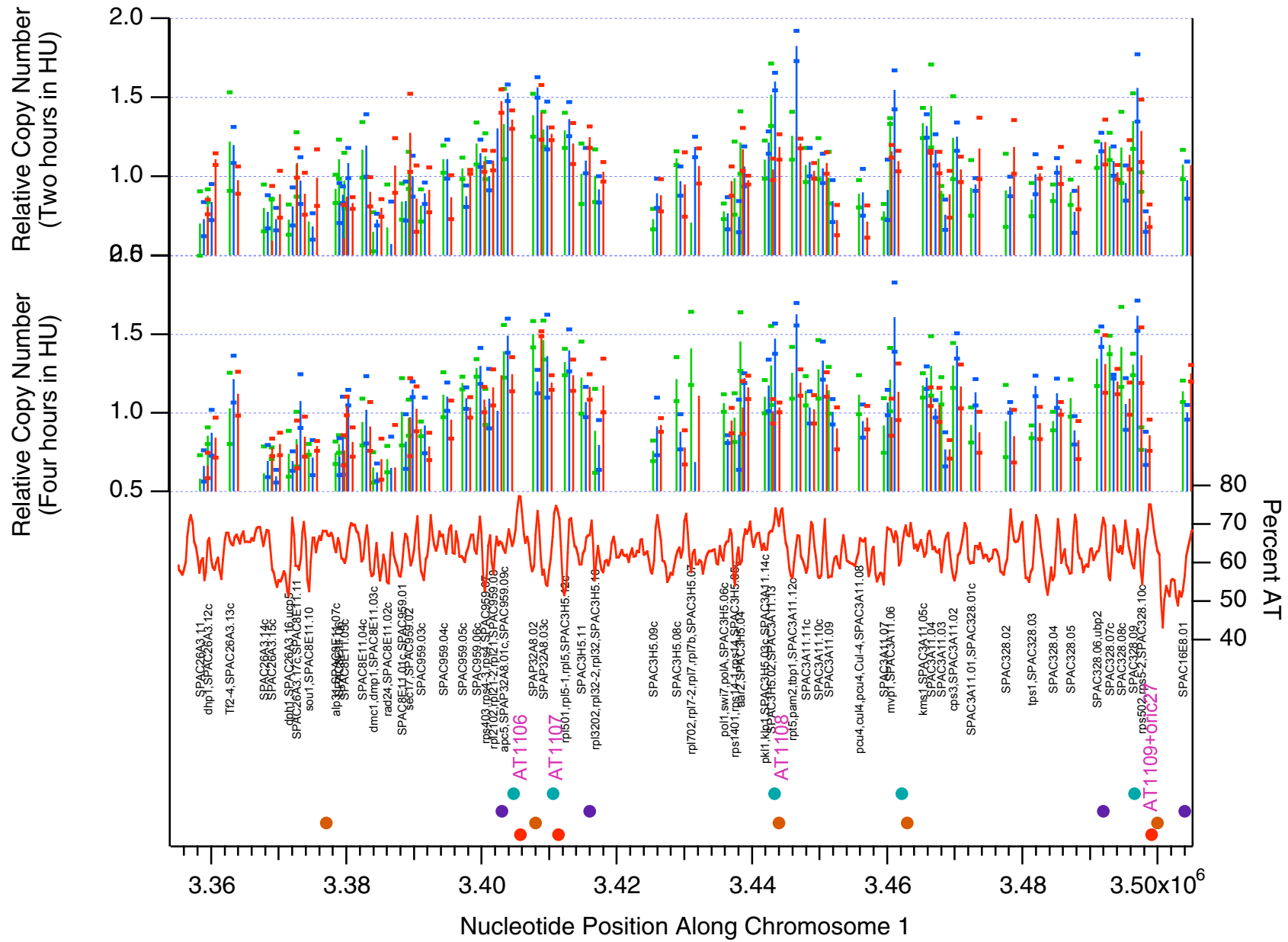

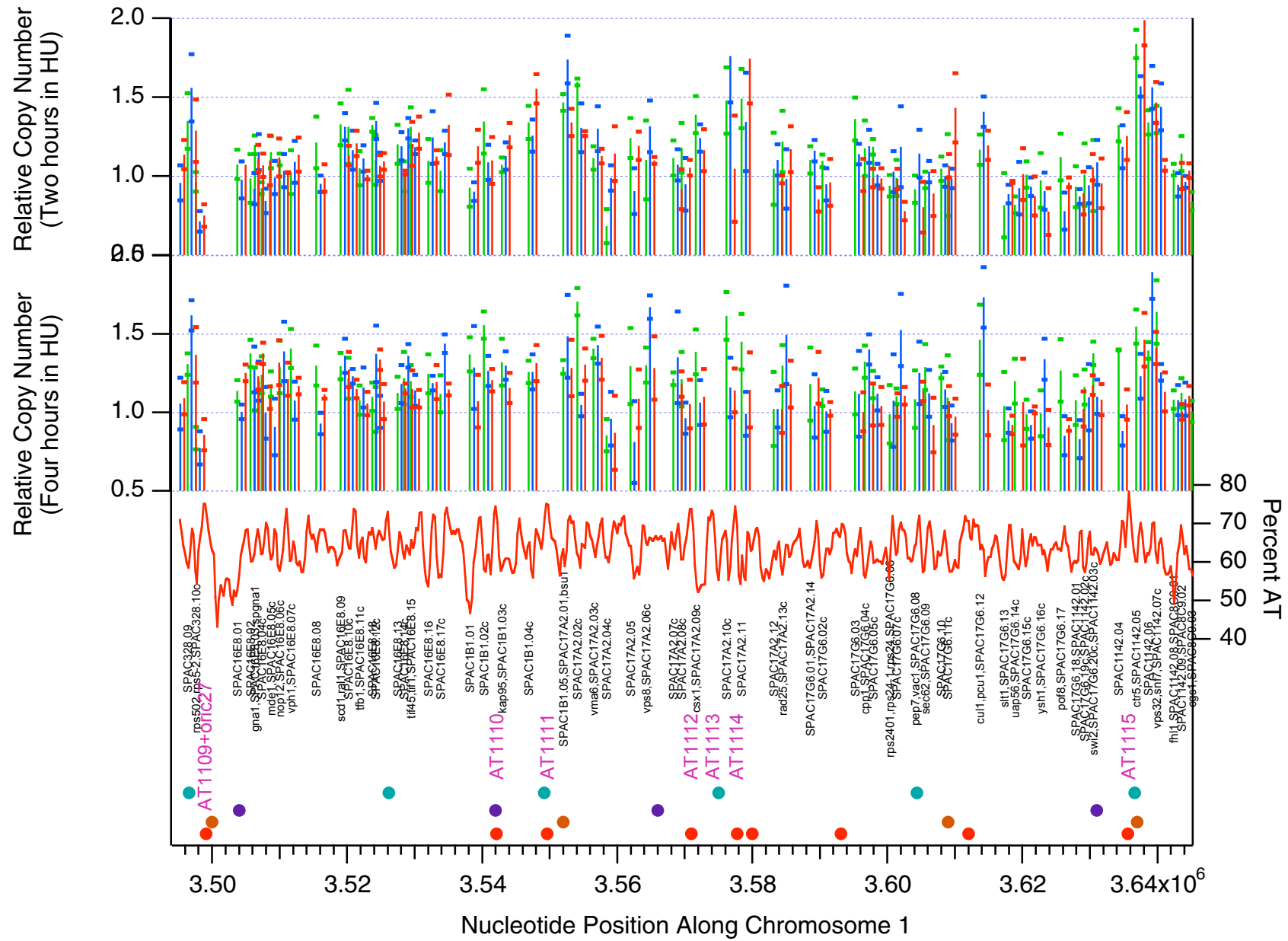

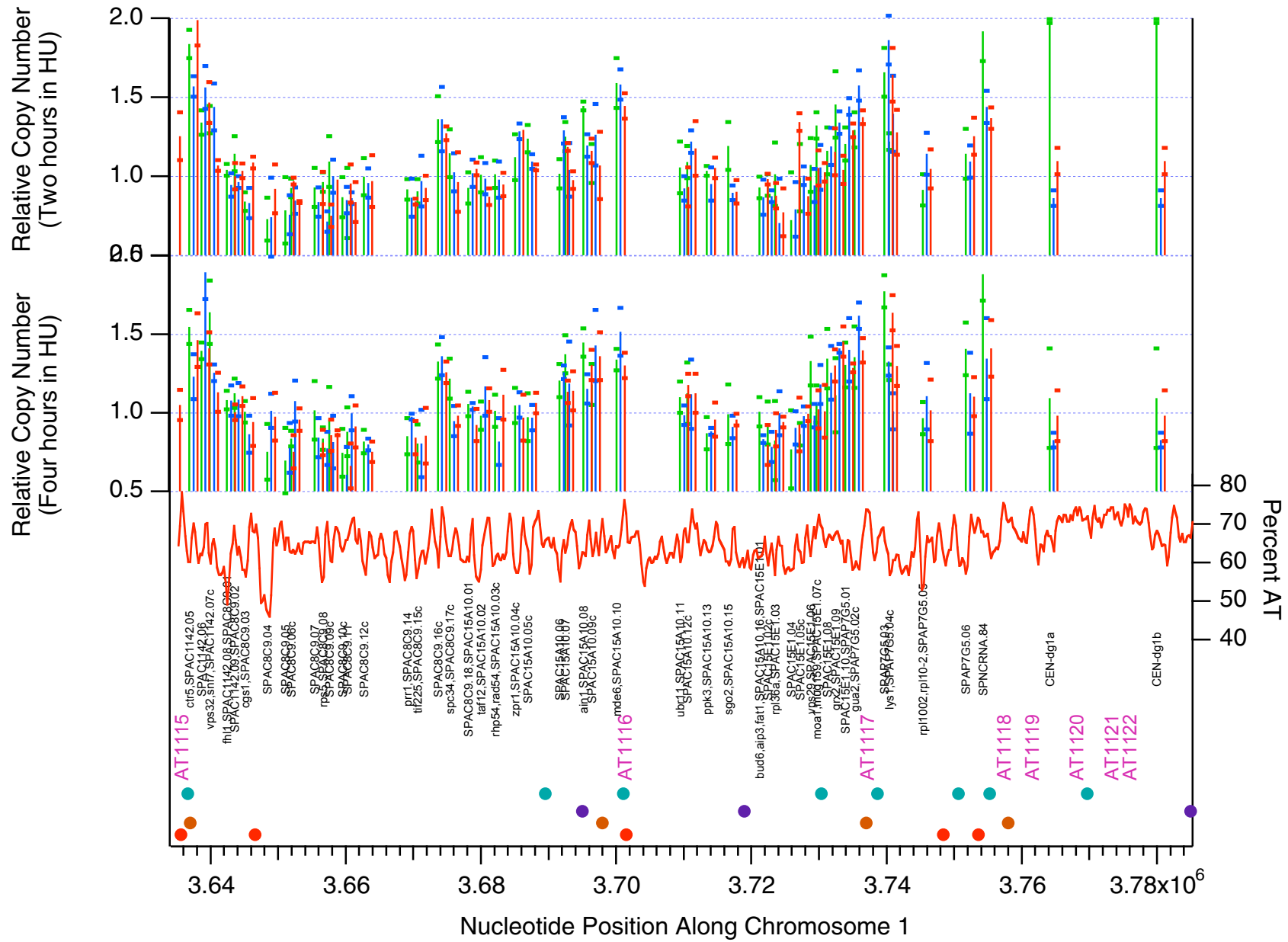

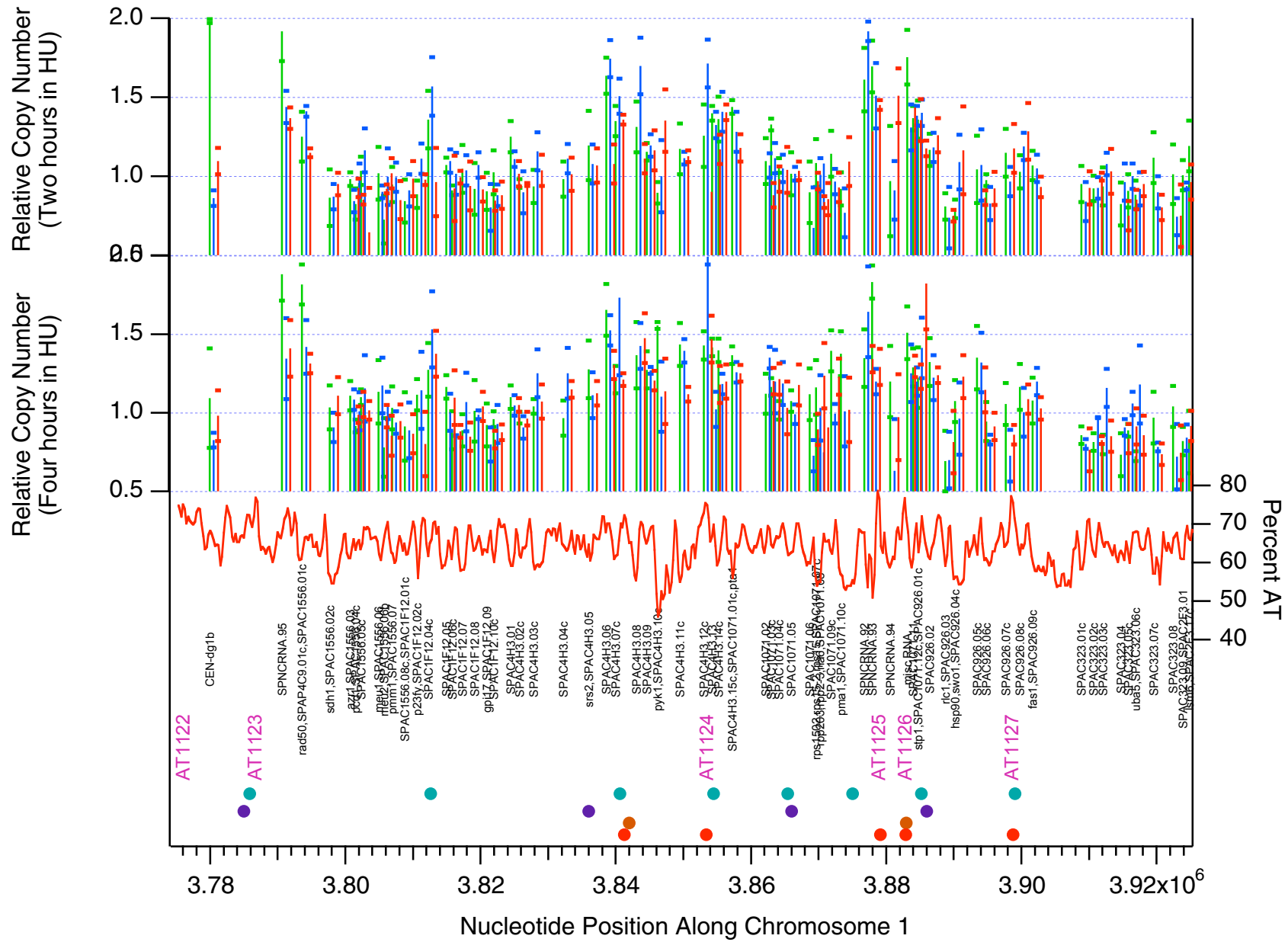

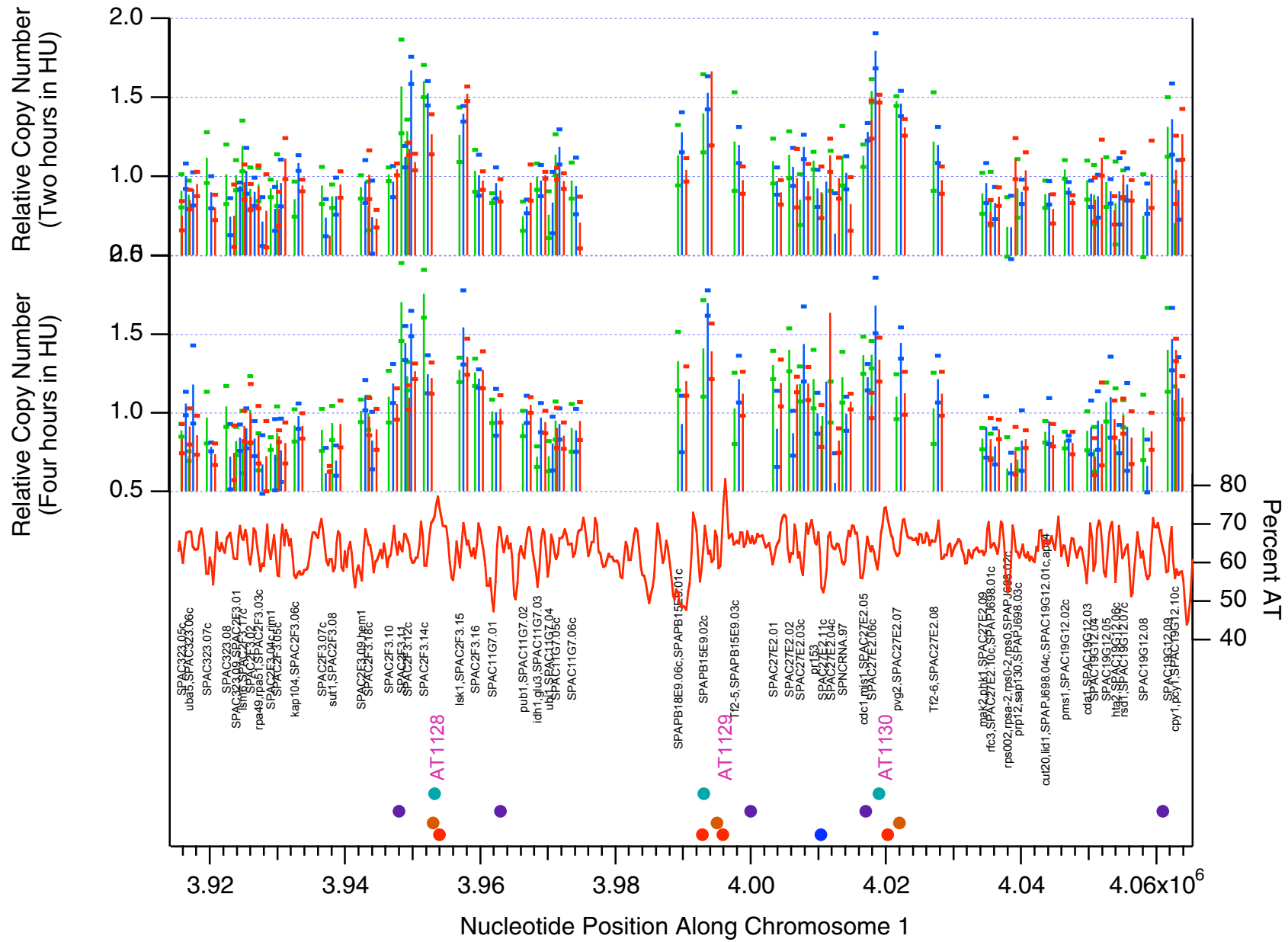

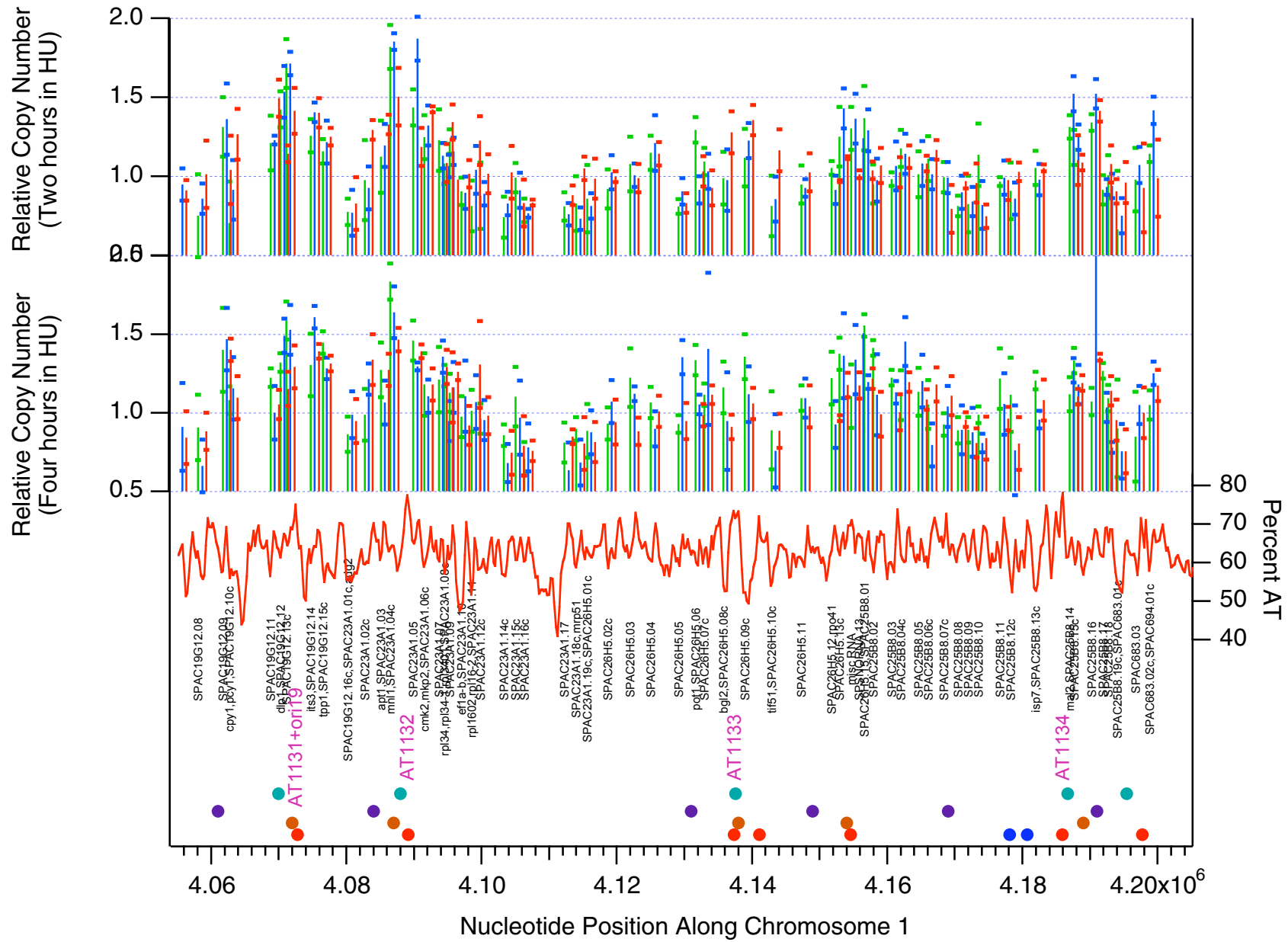

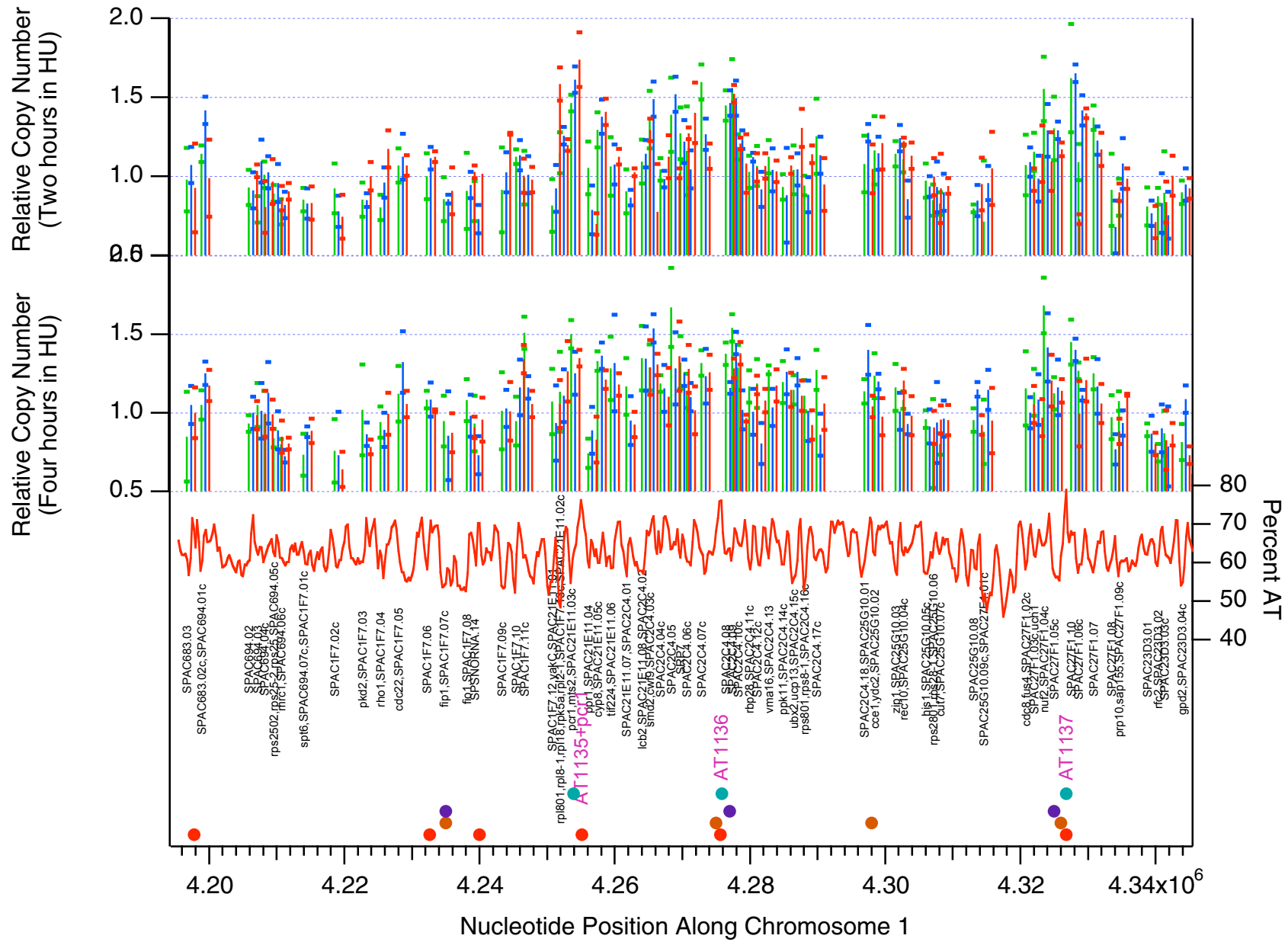

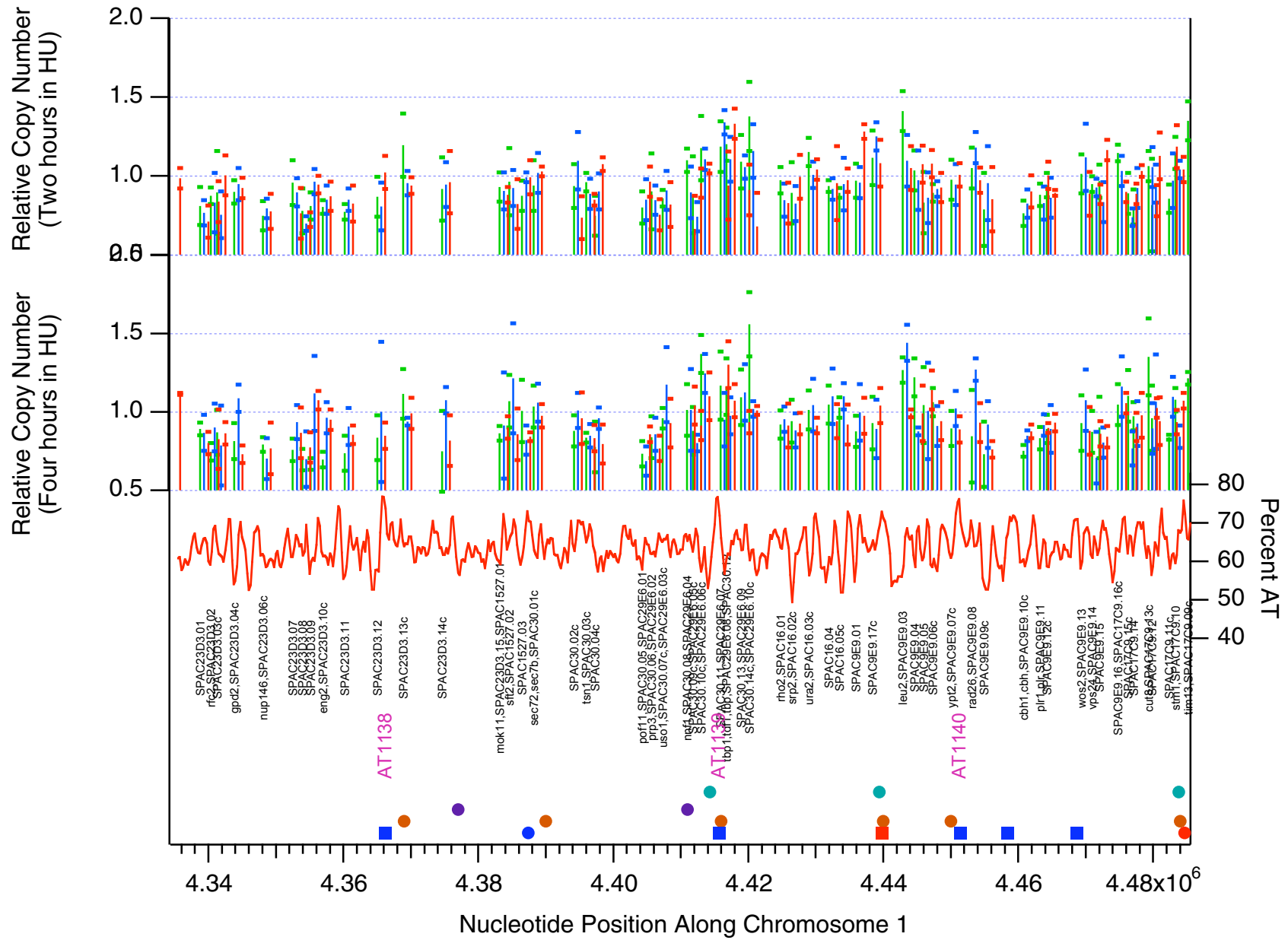

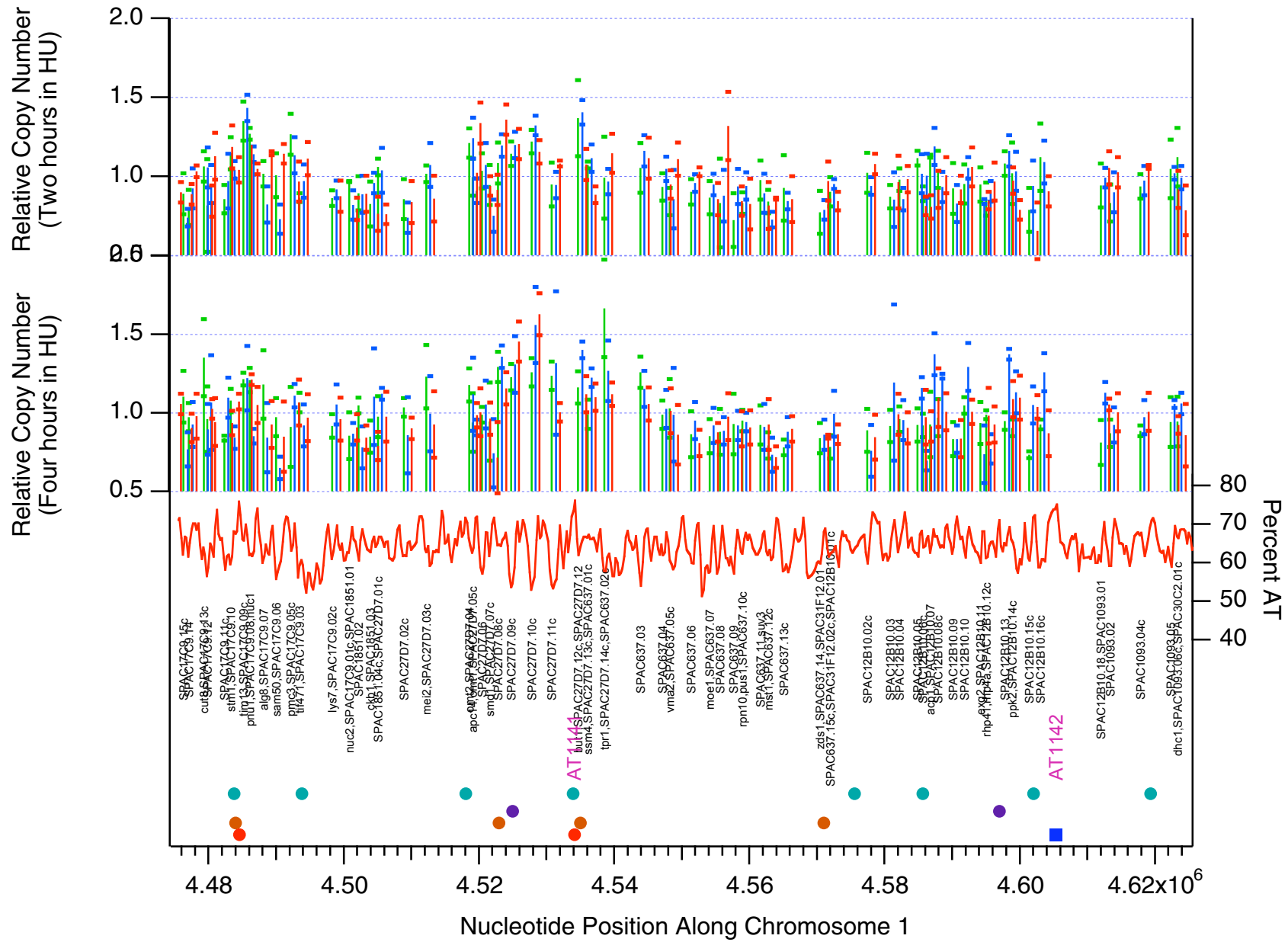

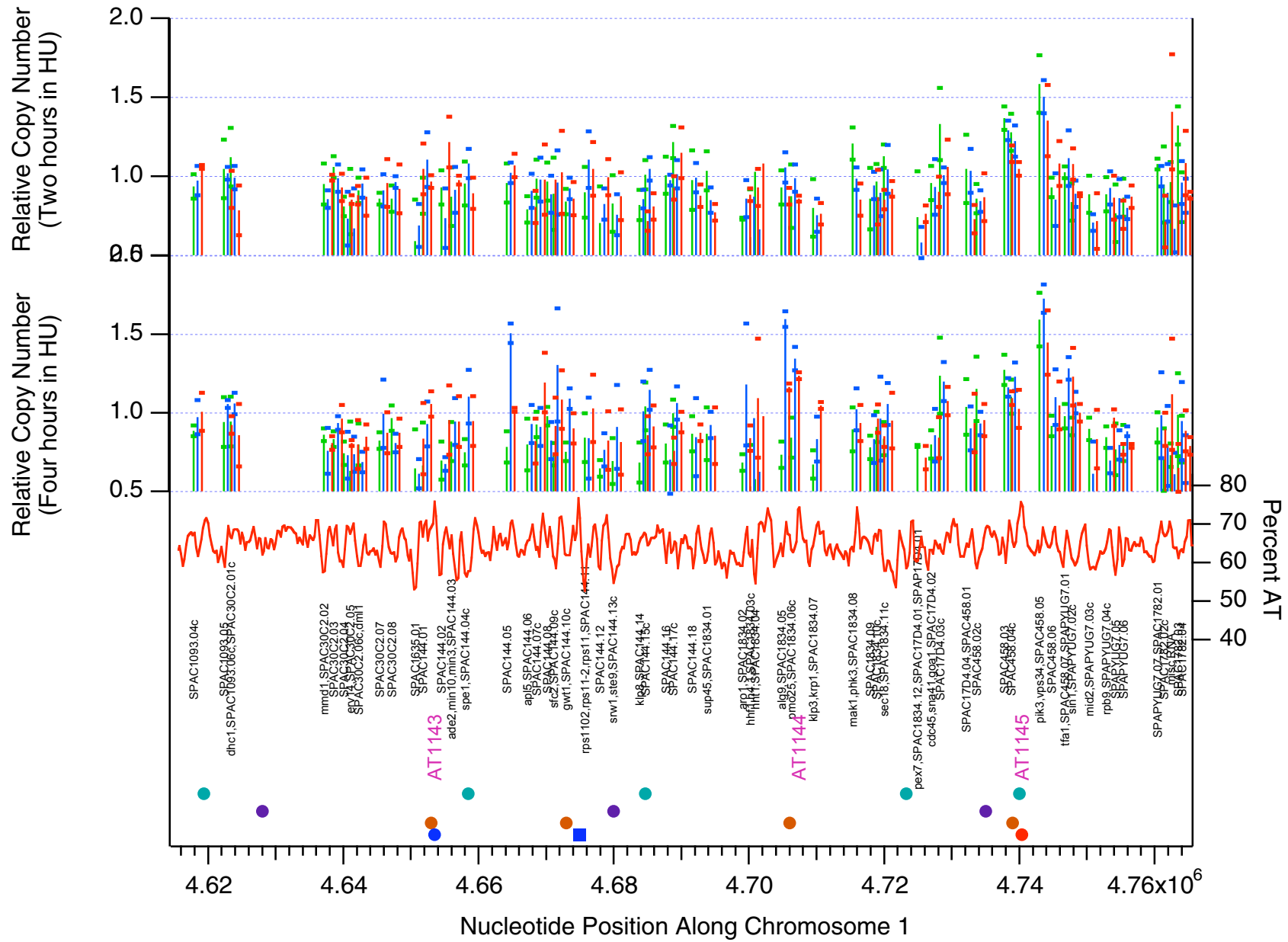

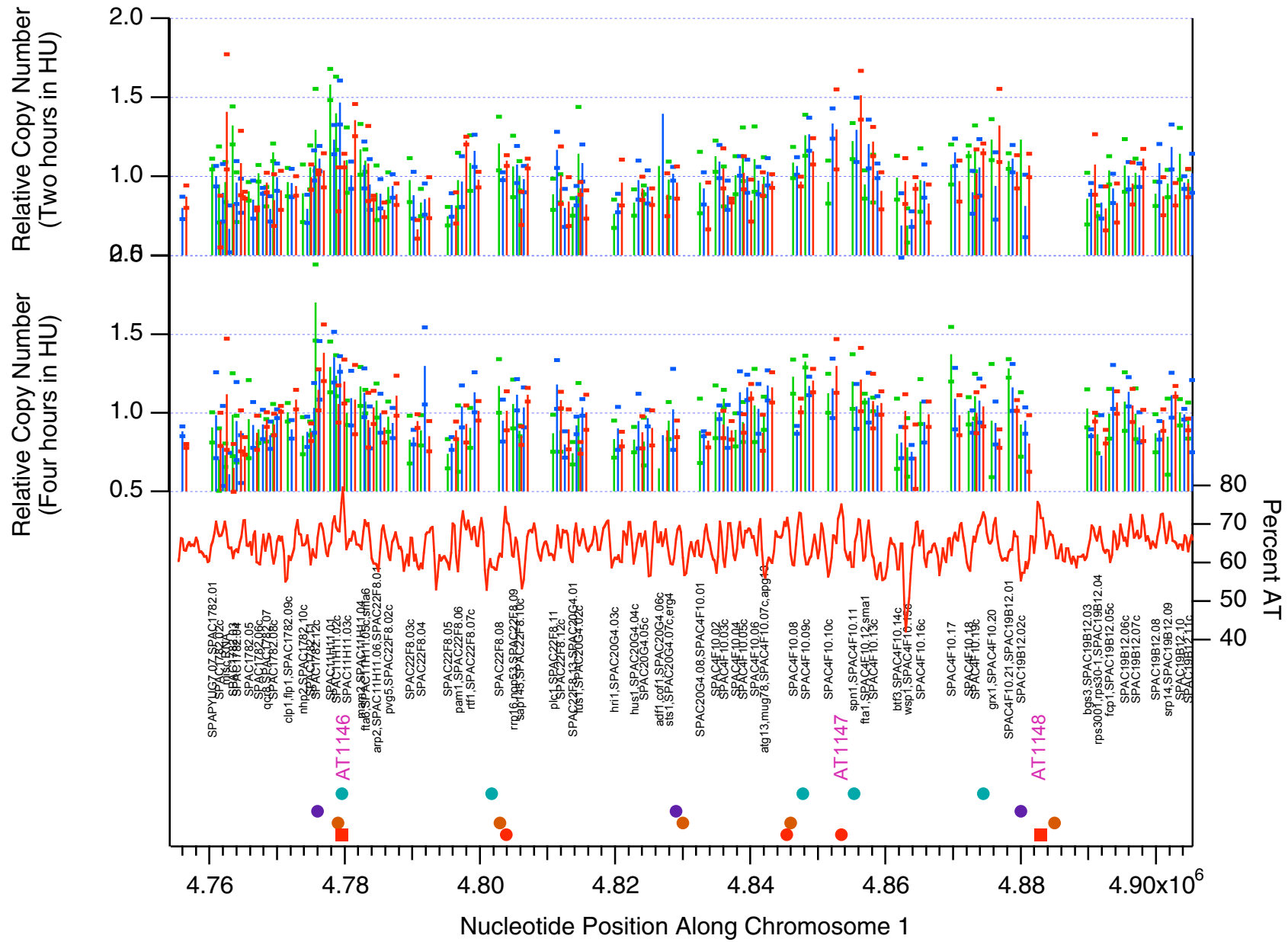

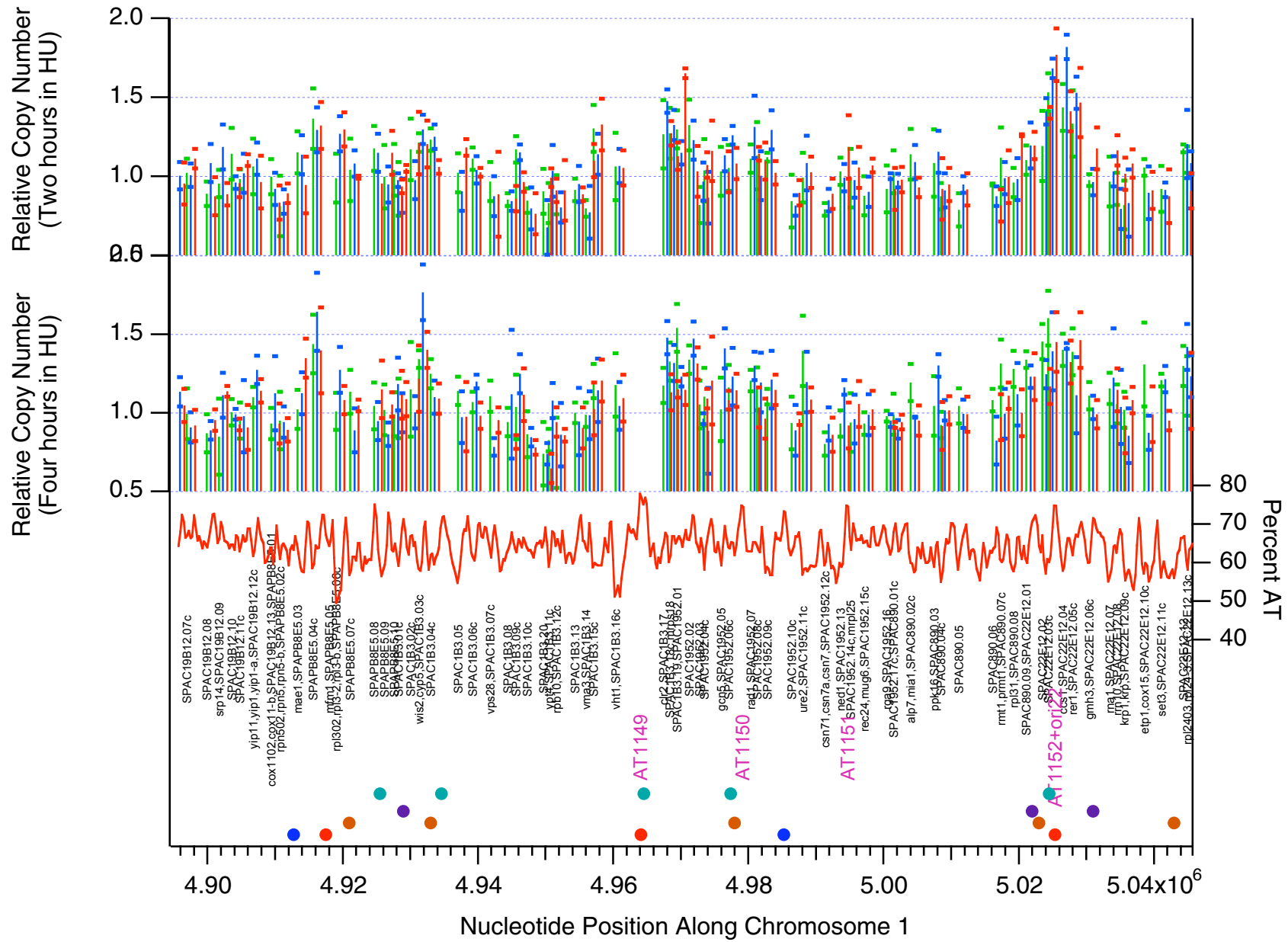

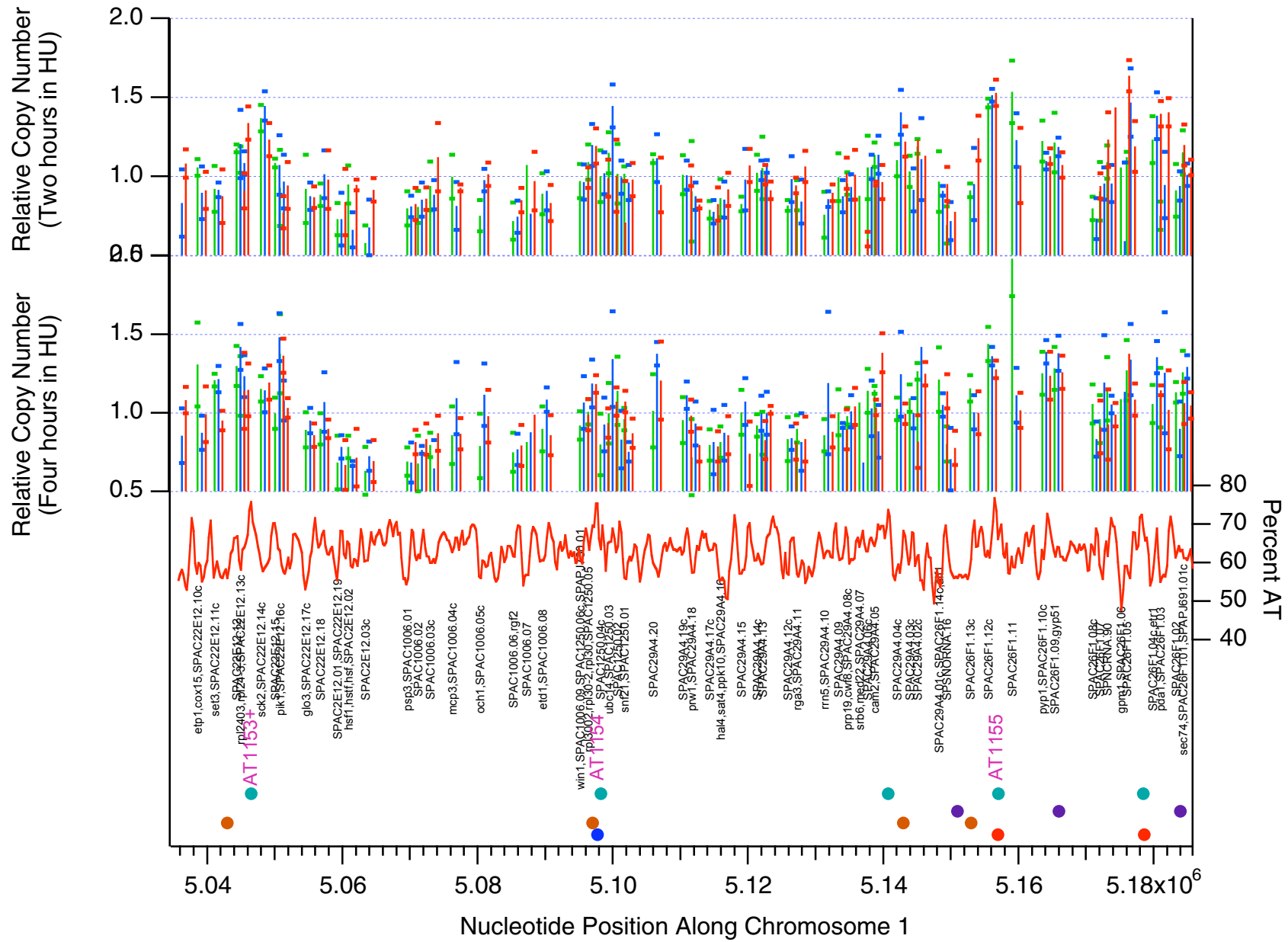

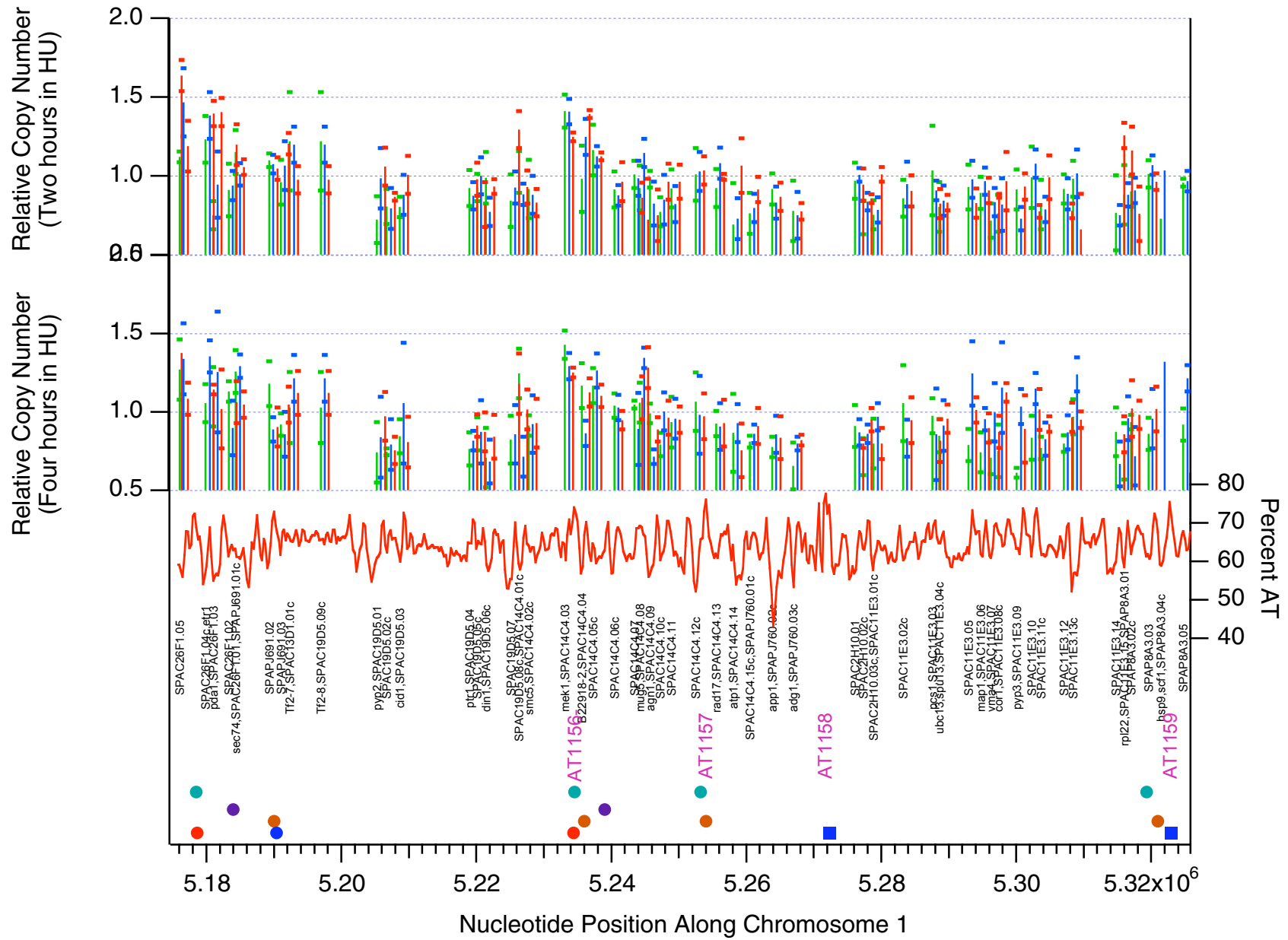

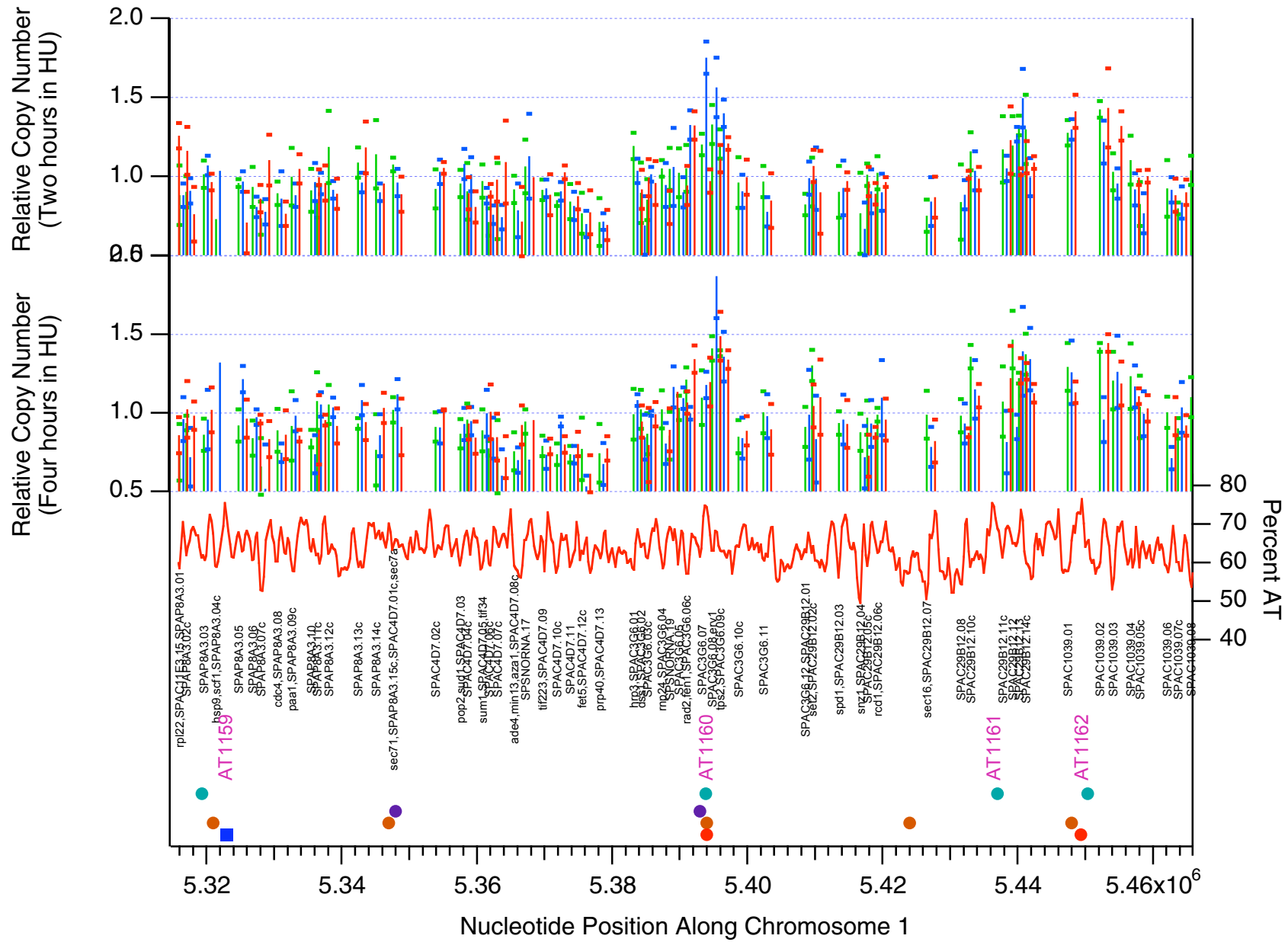

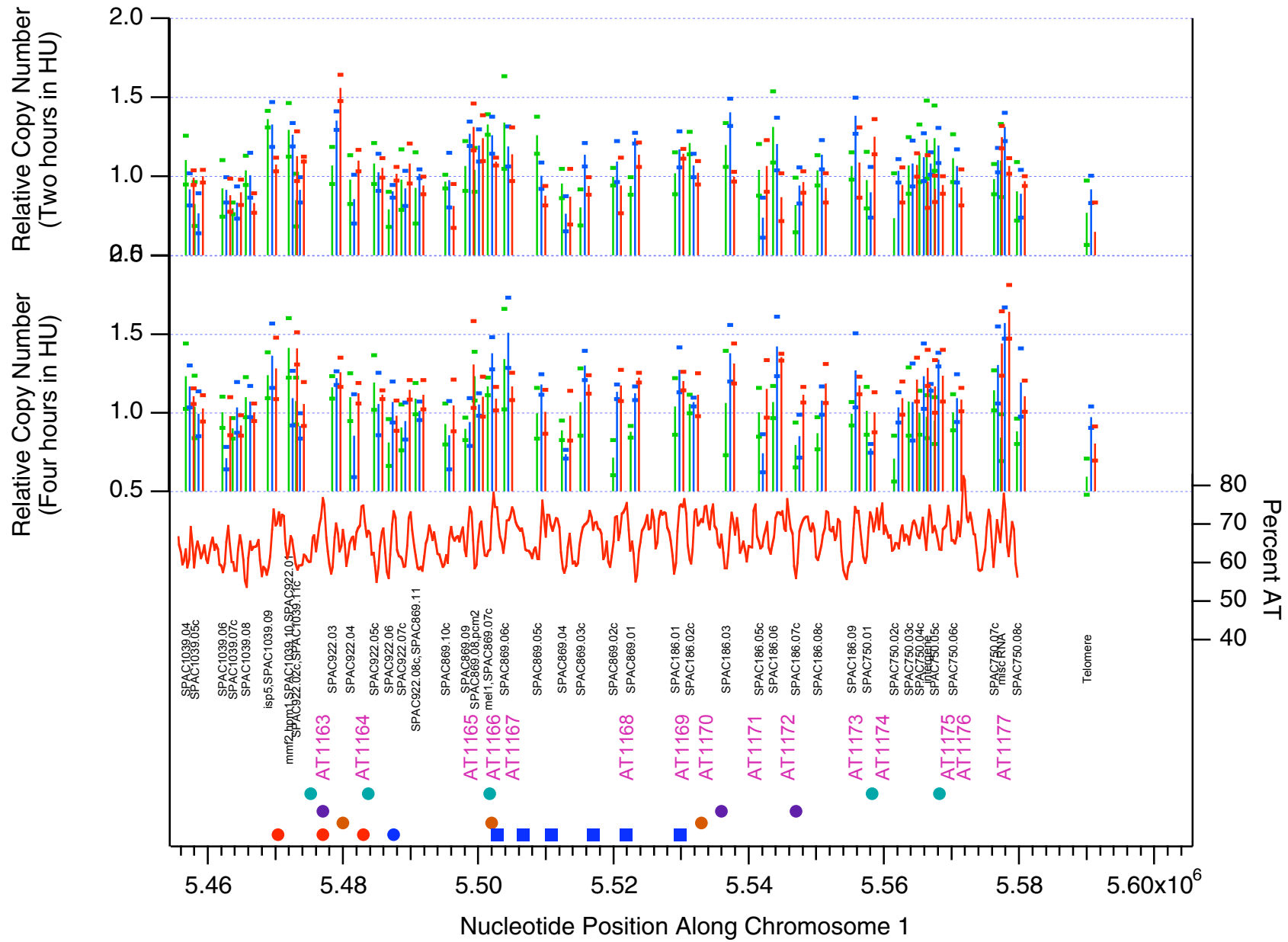

Supplement: Additional file 1 — Graphs of microarray measurements of copy number changes throughout chromosome 1. A multi-page PDF file, with graphs for chromosome 1 based on the data in Additional File 4. The results are shown at 150 kb per page, with 10-kb overlaps between pages. The symbols are explained in the legend to Figure 2. [file 1471-2199-8-112-S1.pdf]

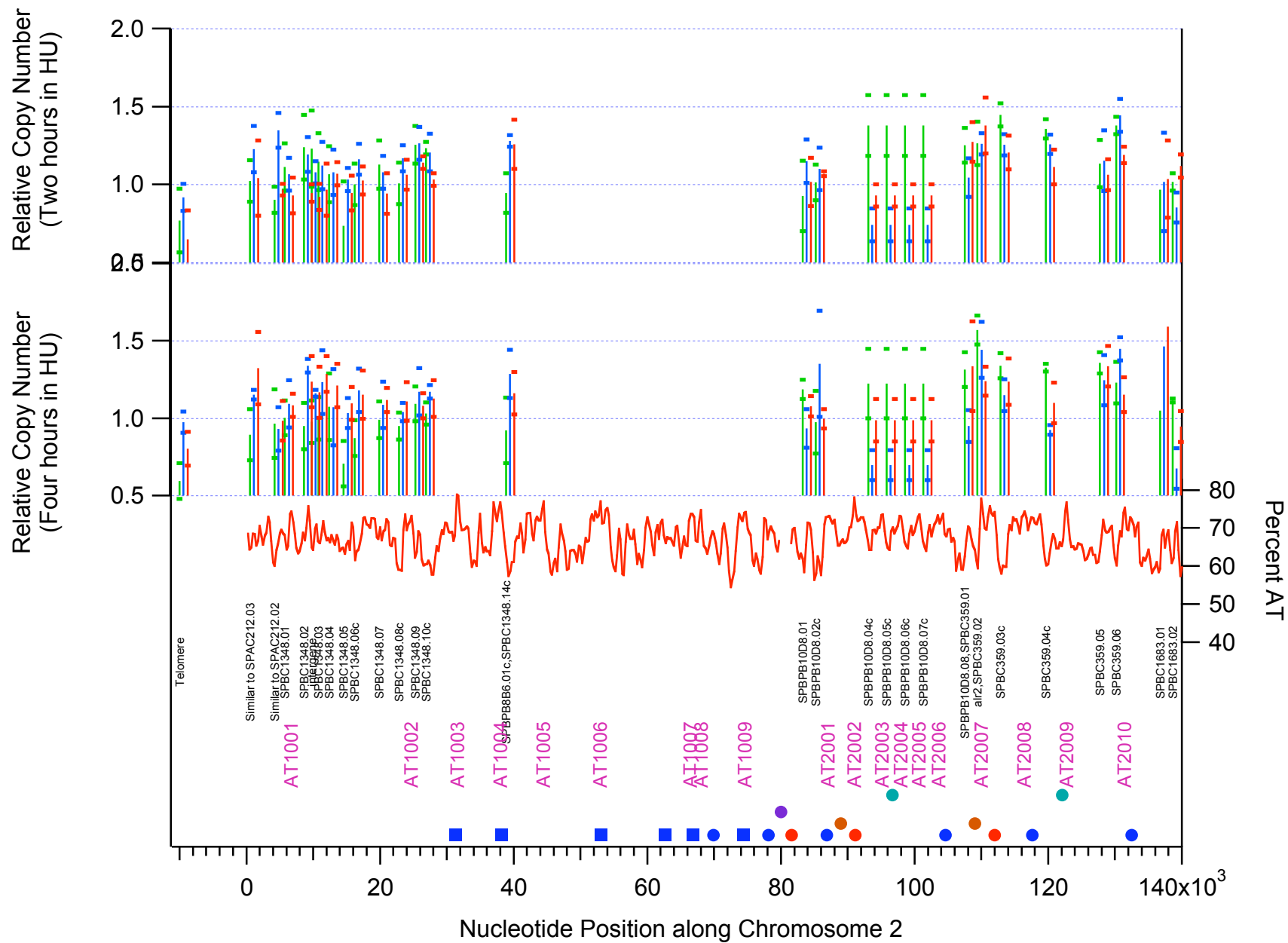

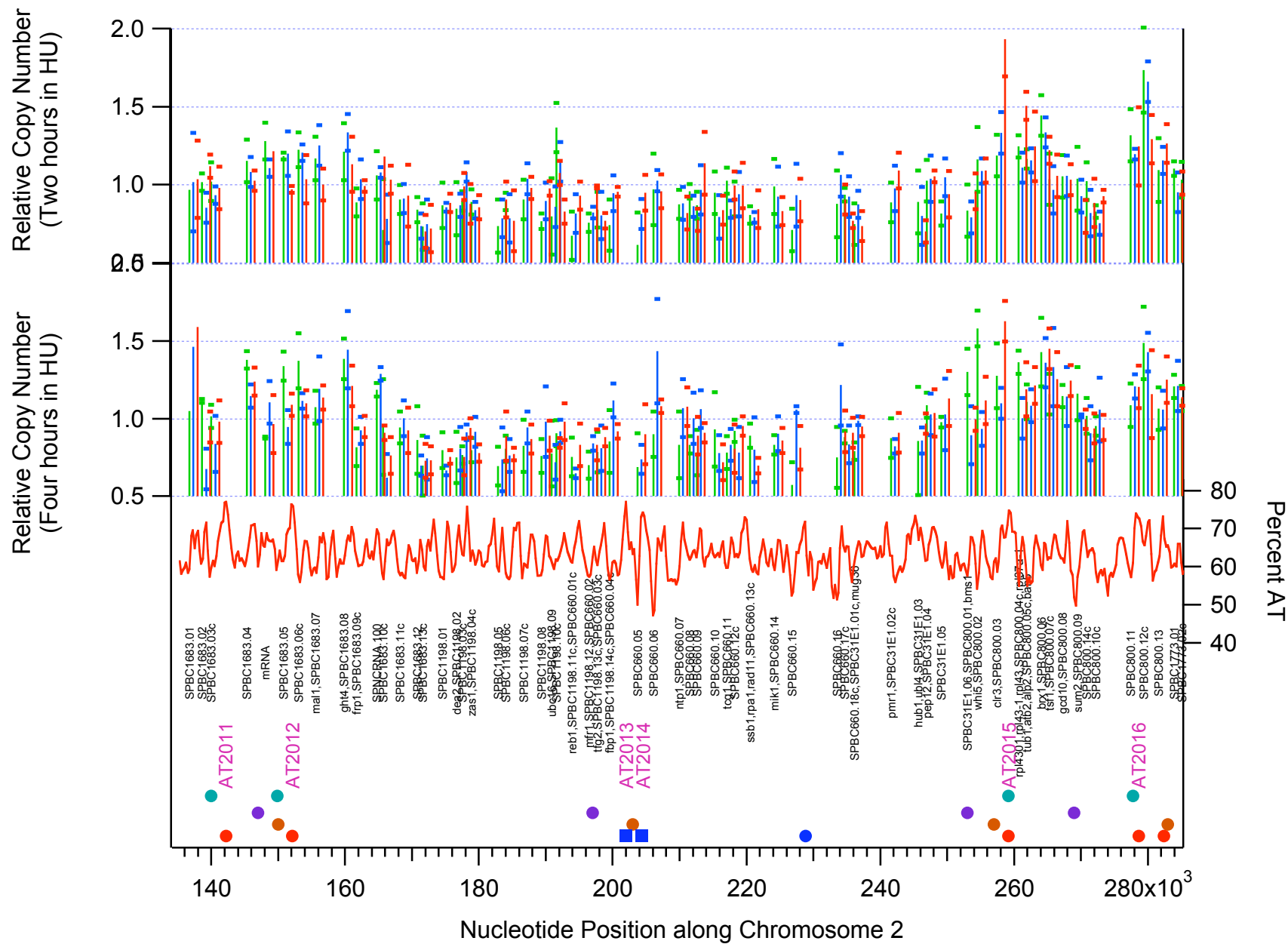

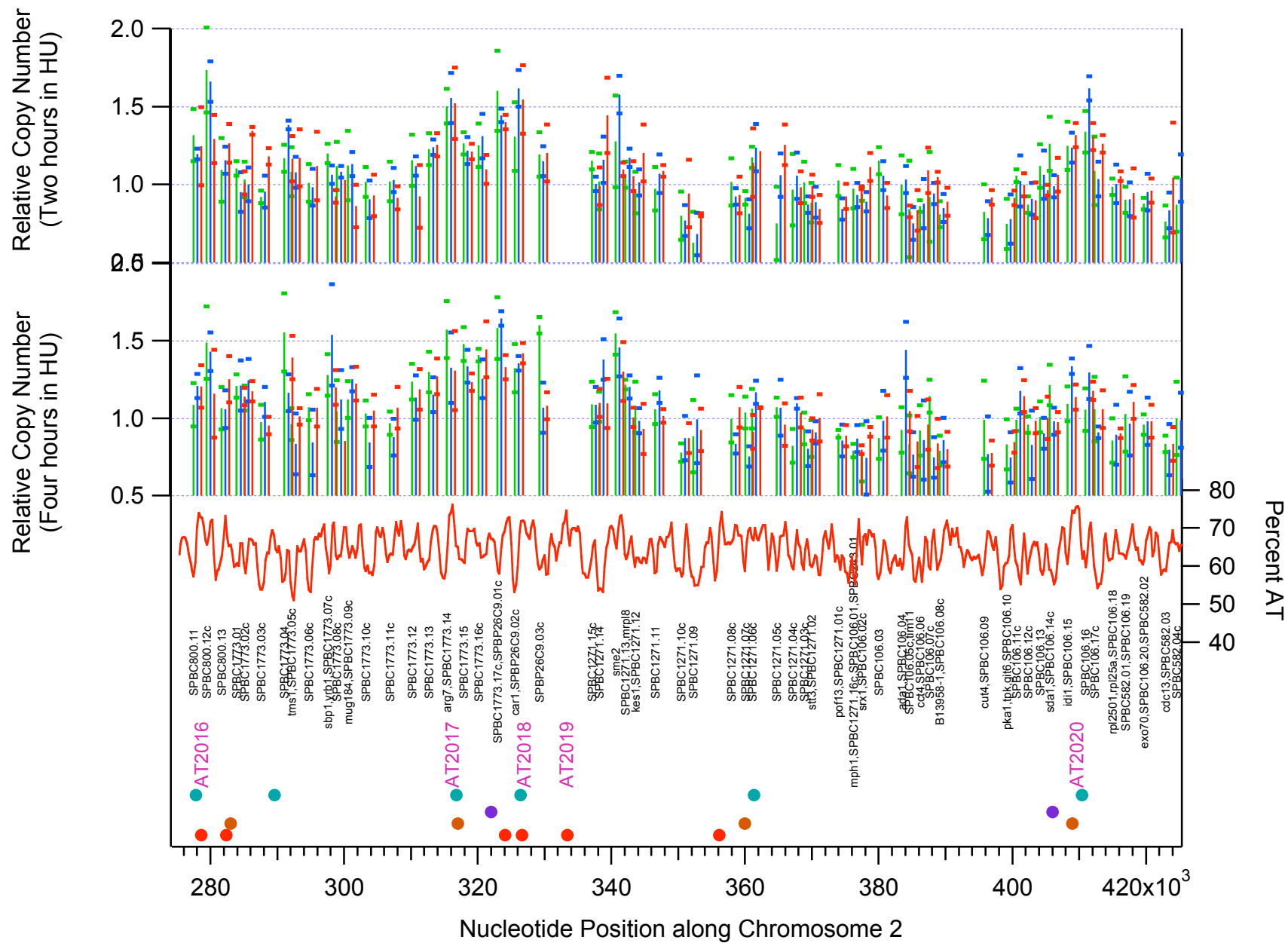

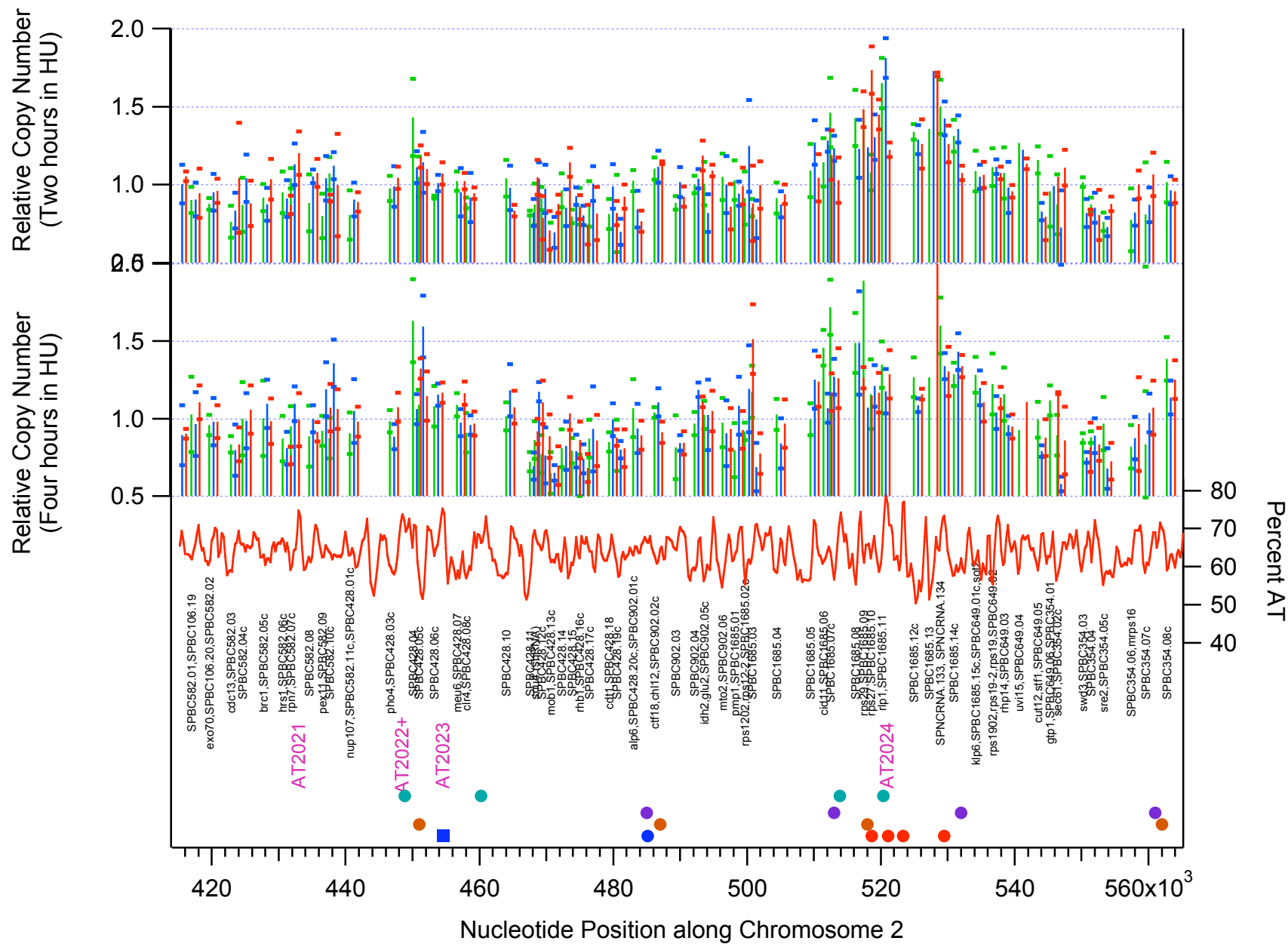

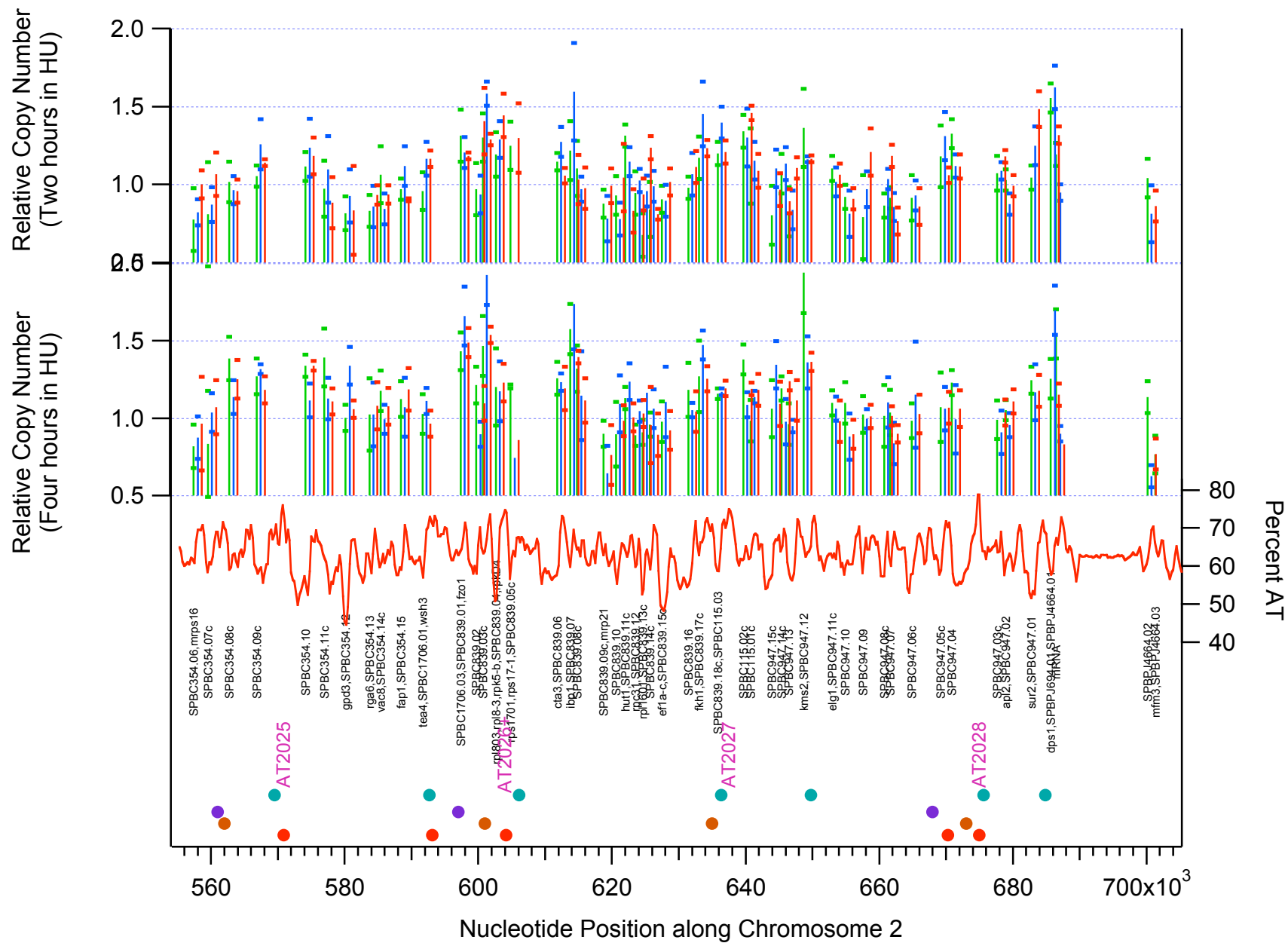

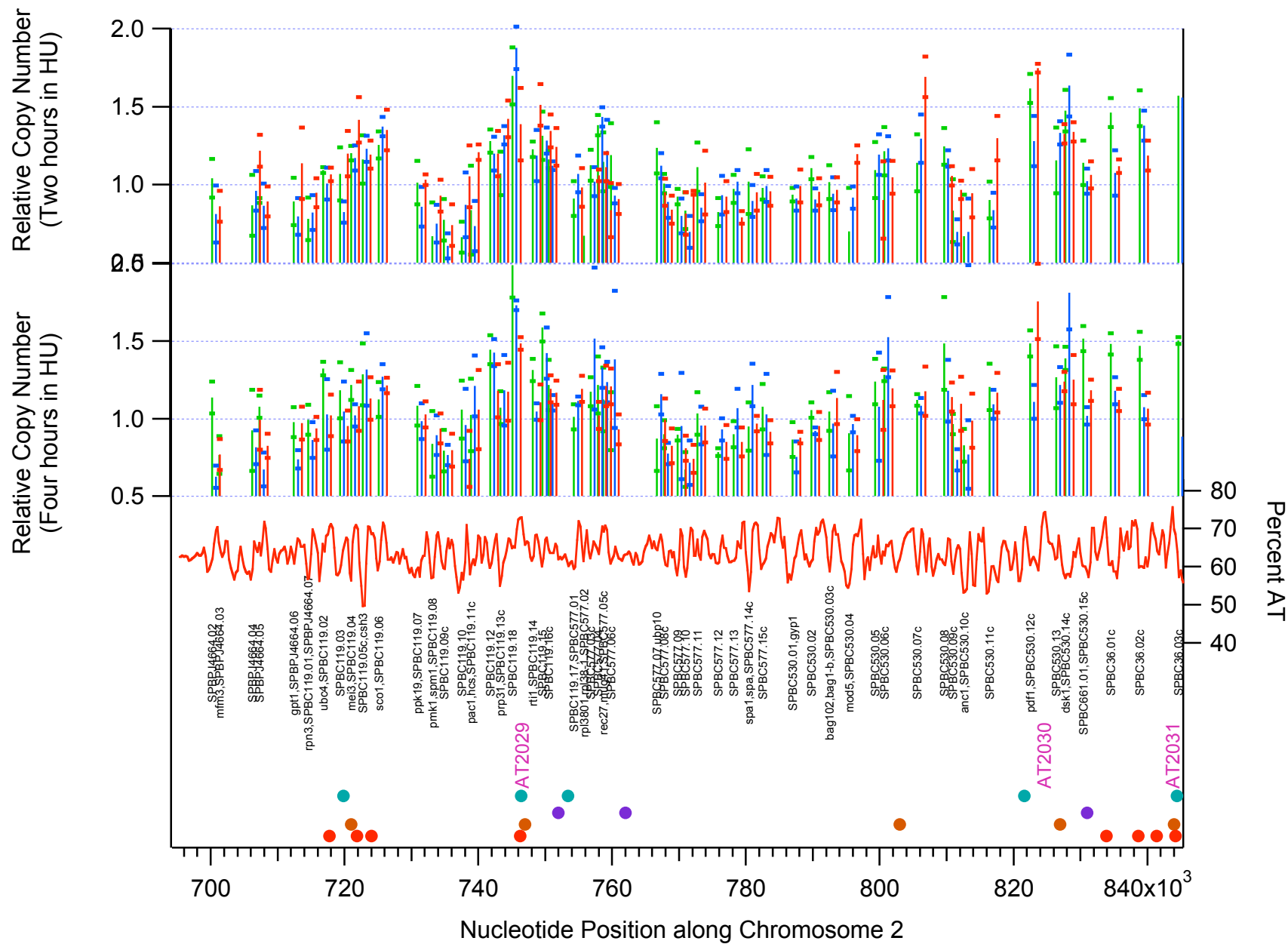

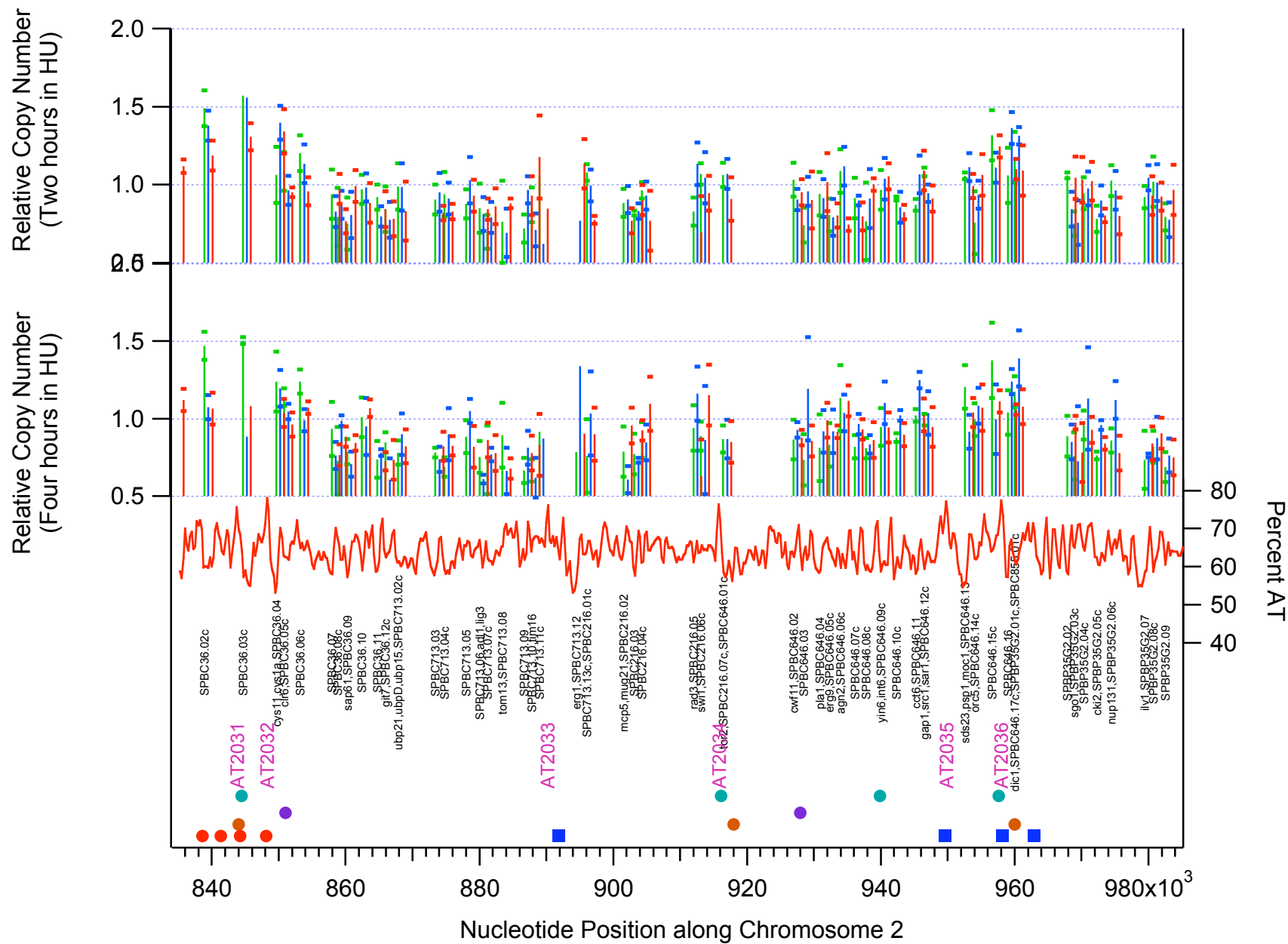

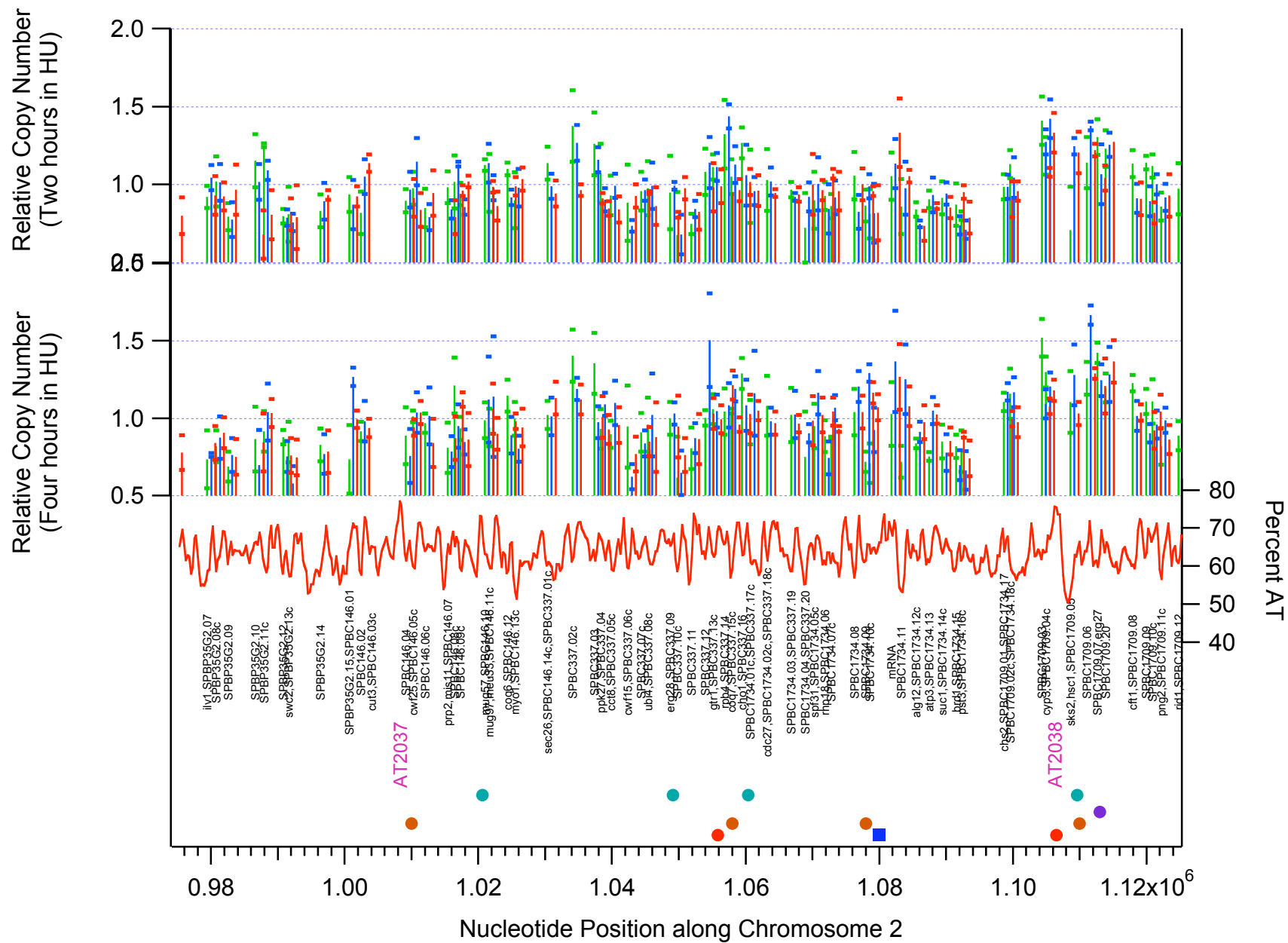

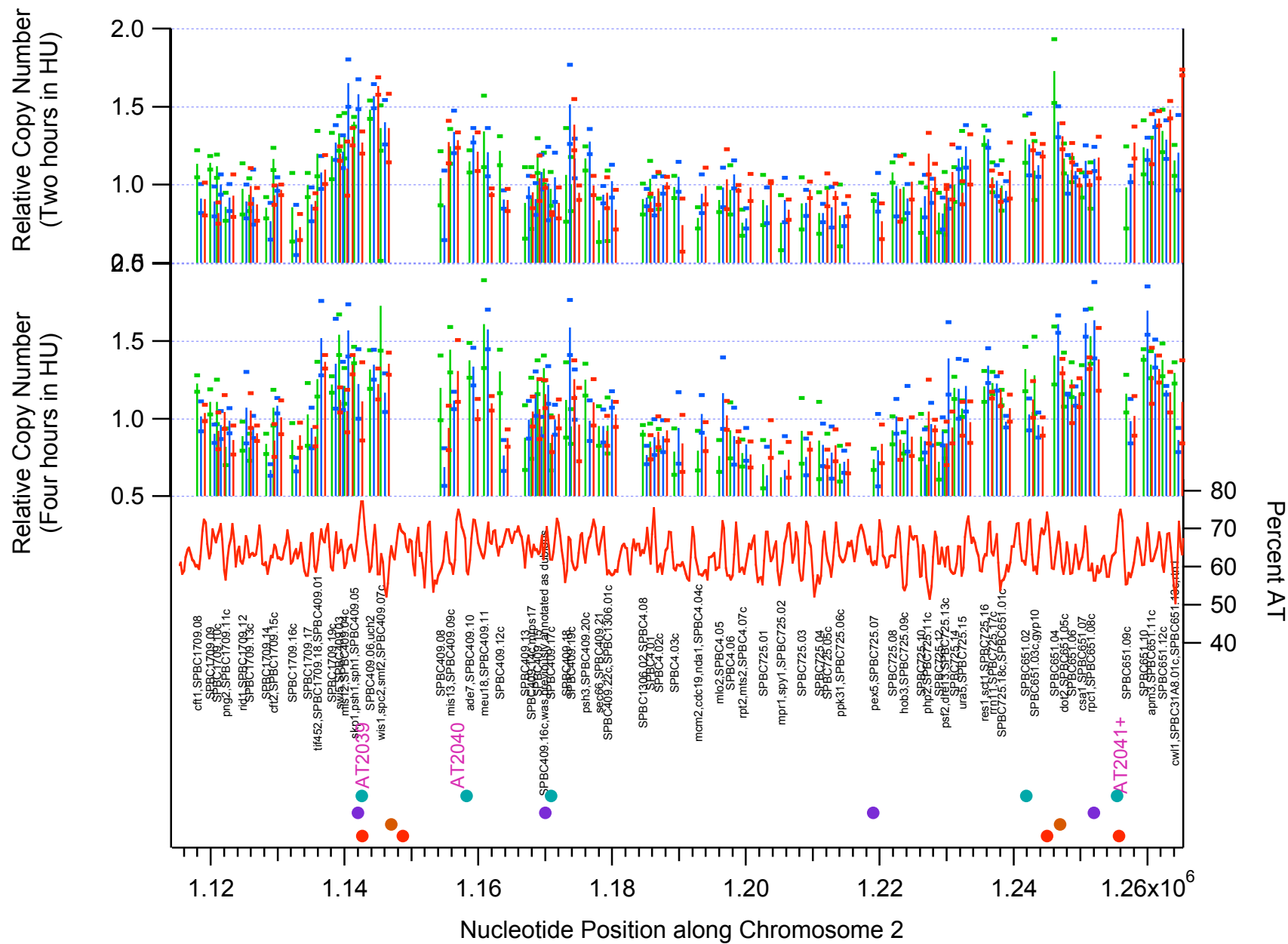

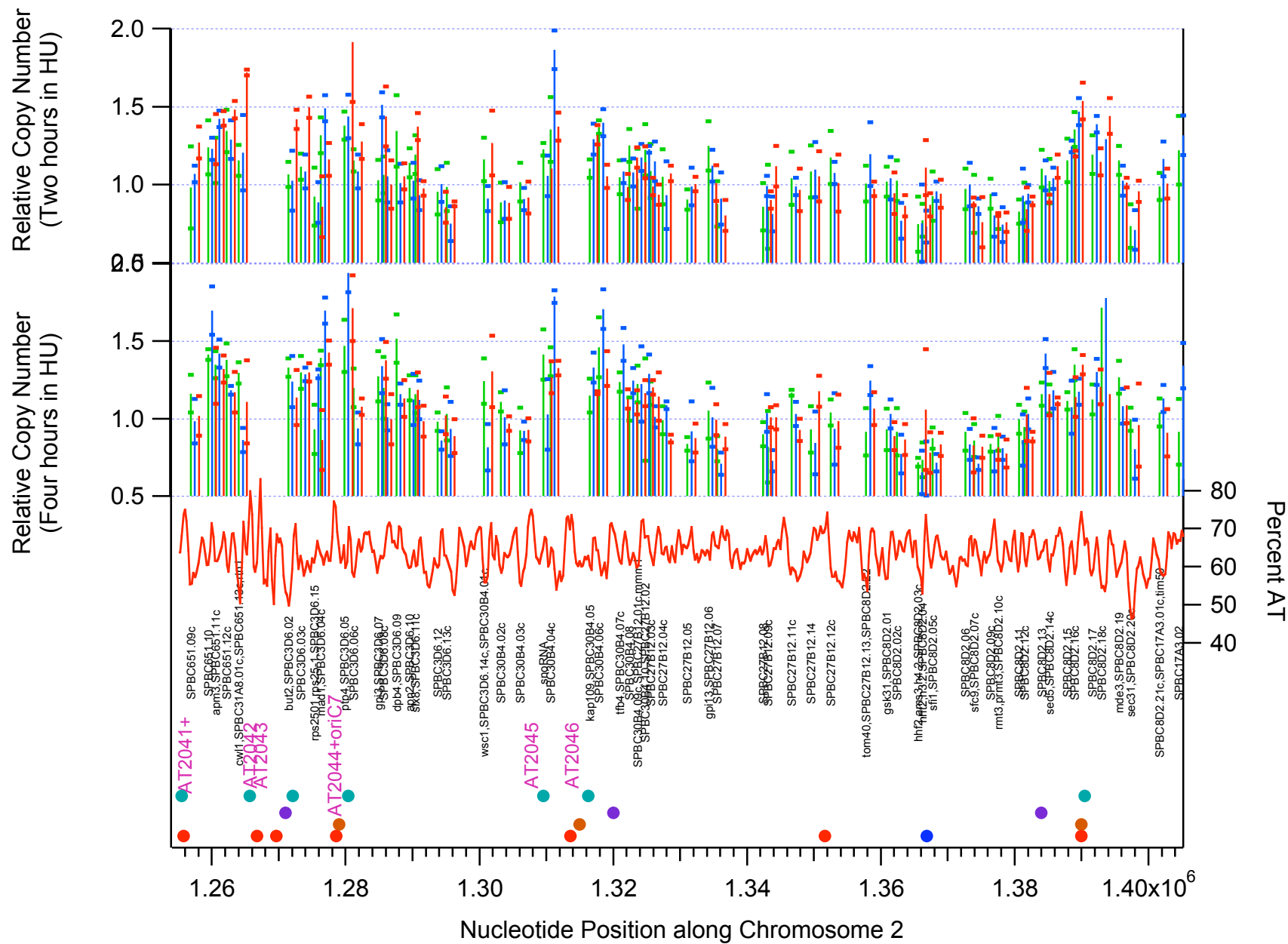

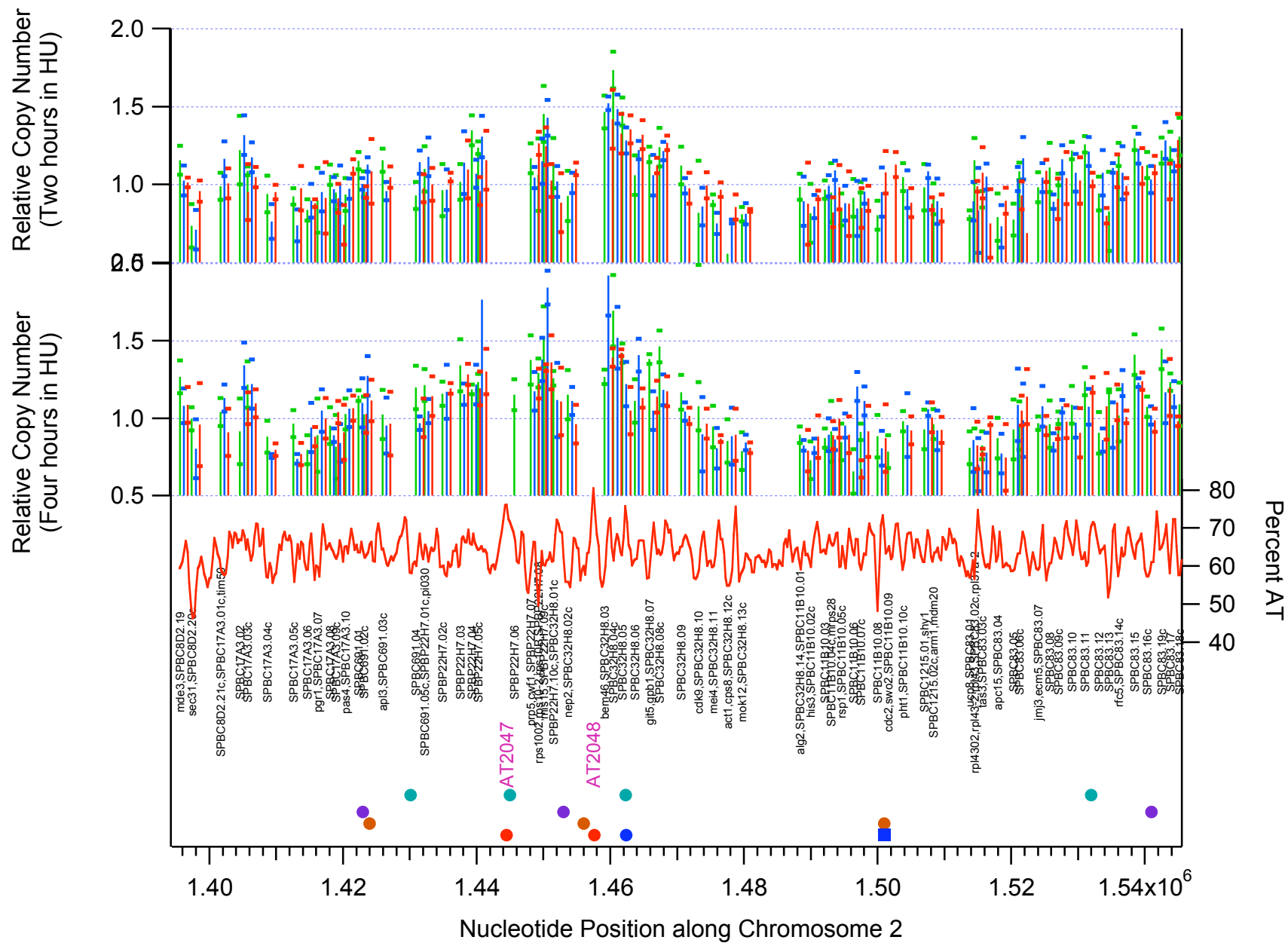

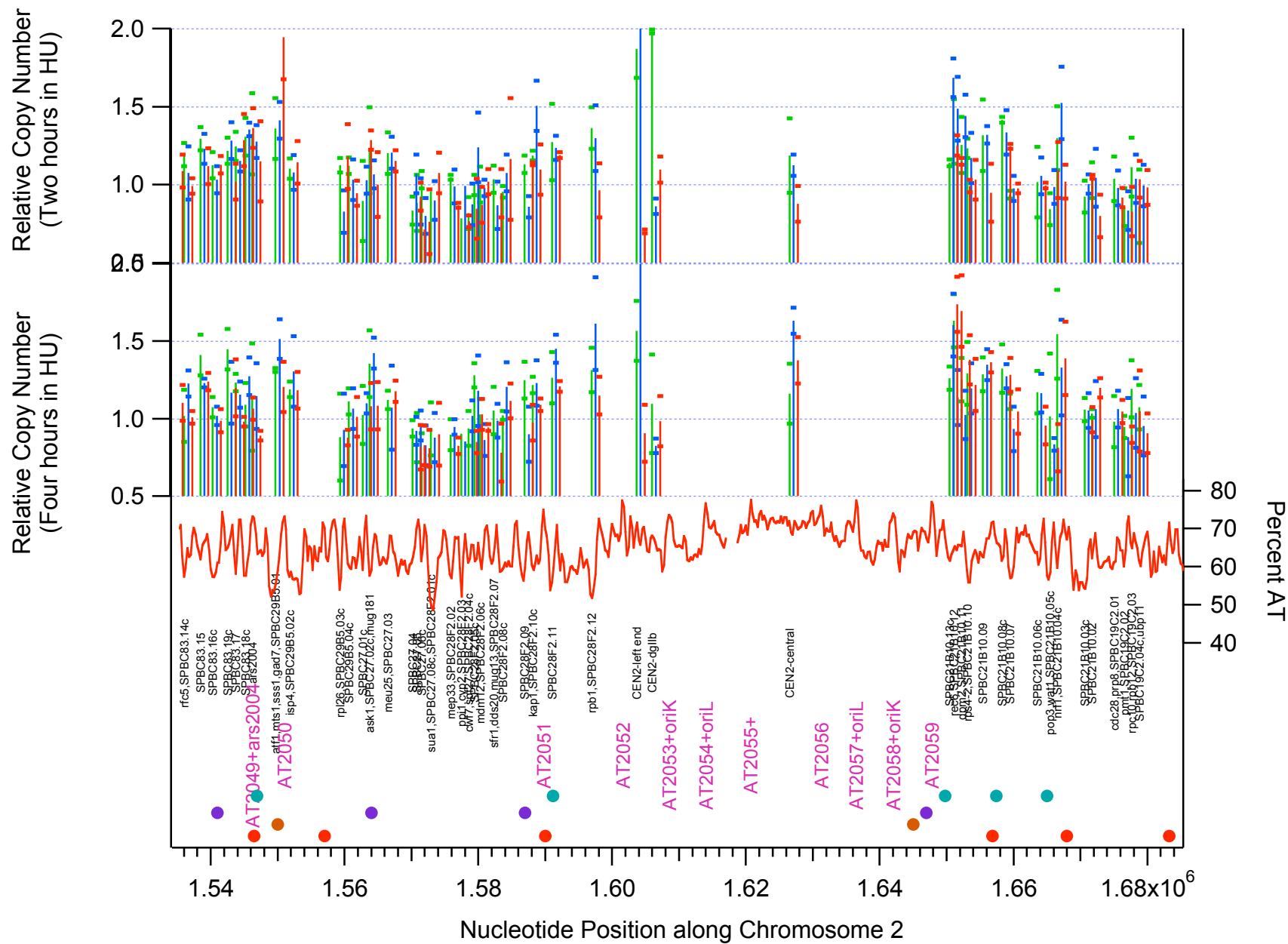



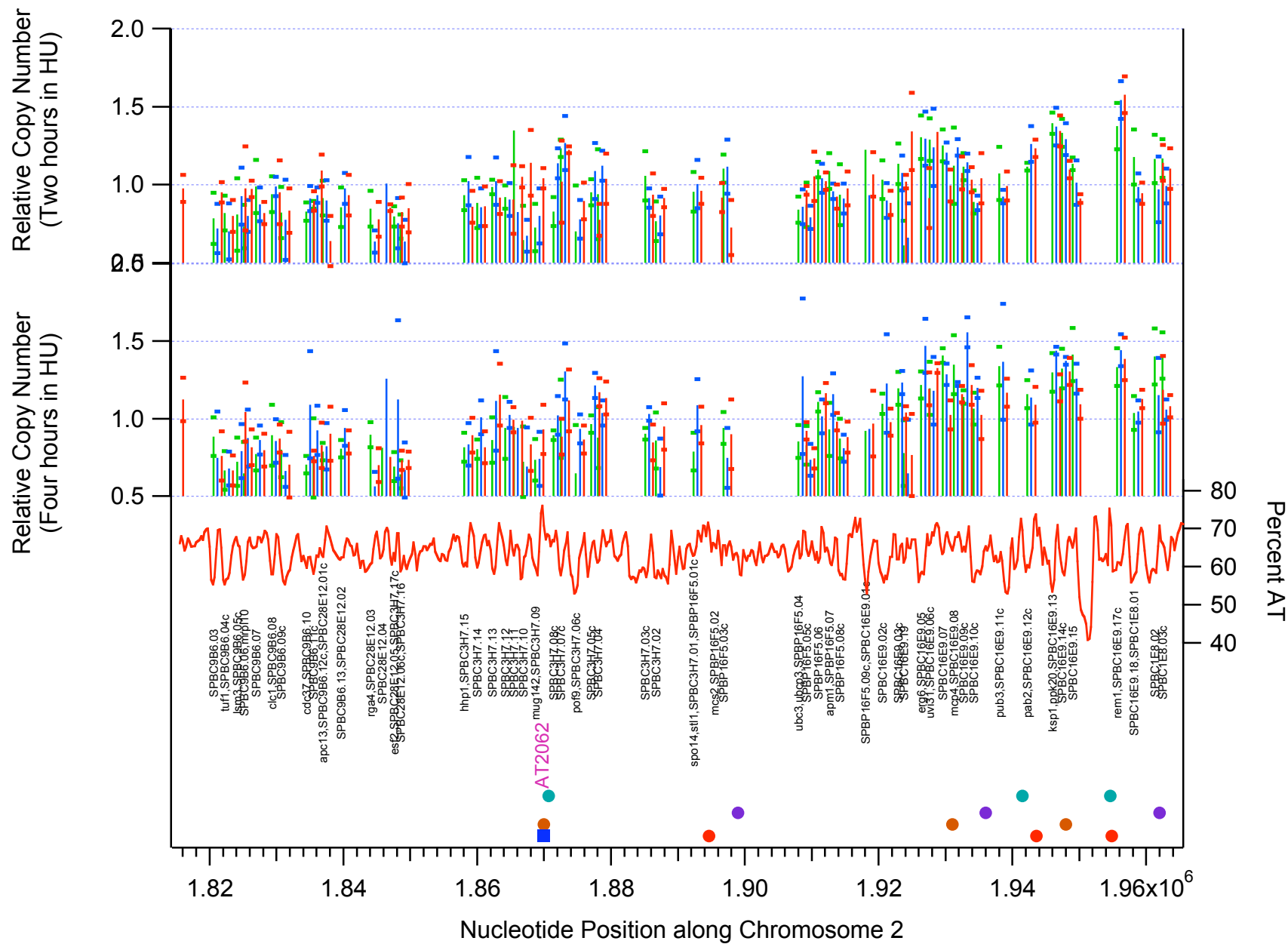

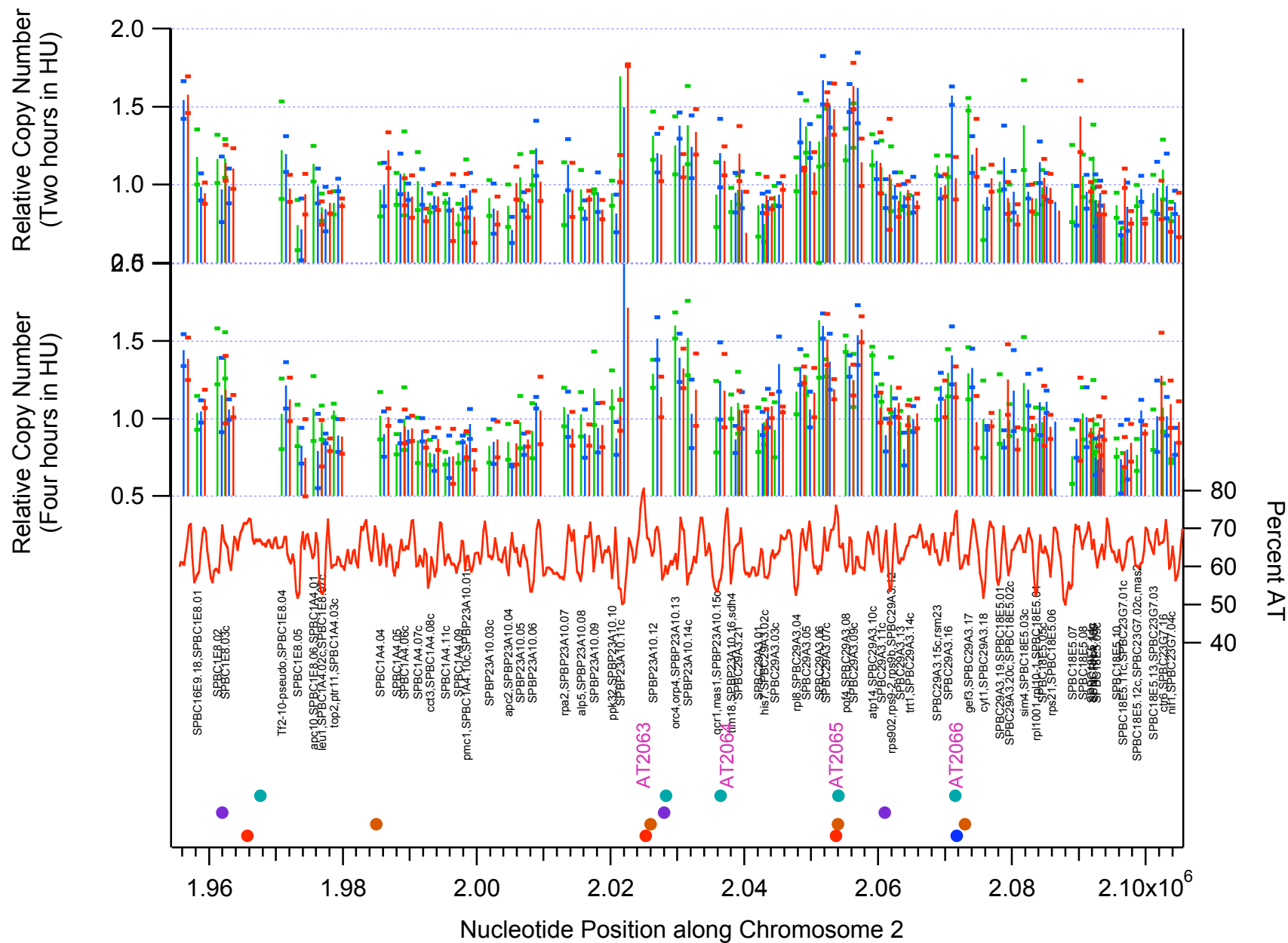



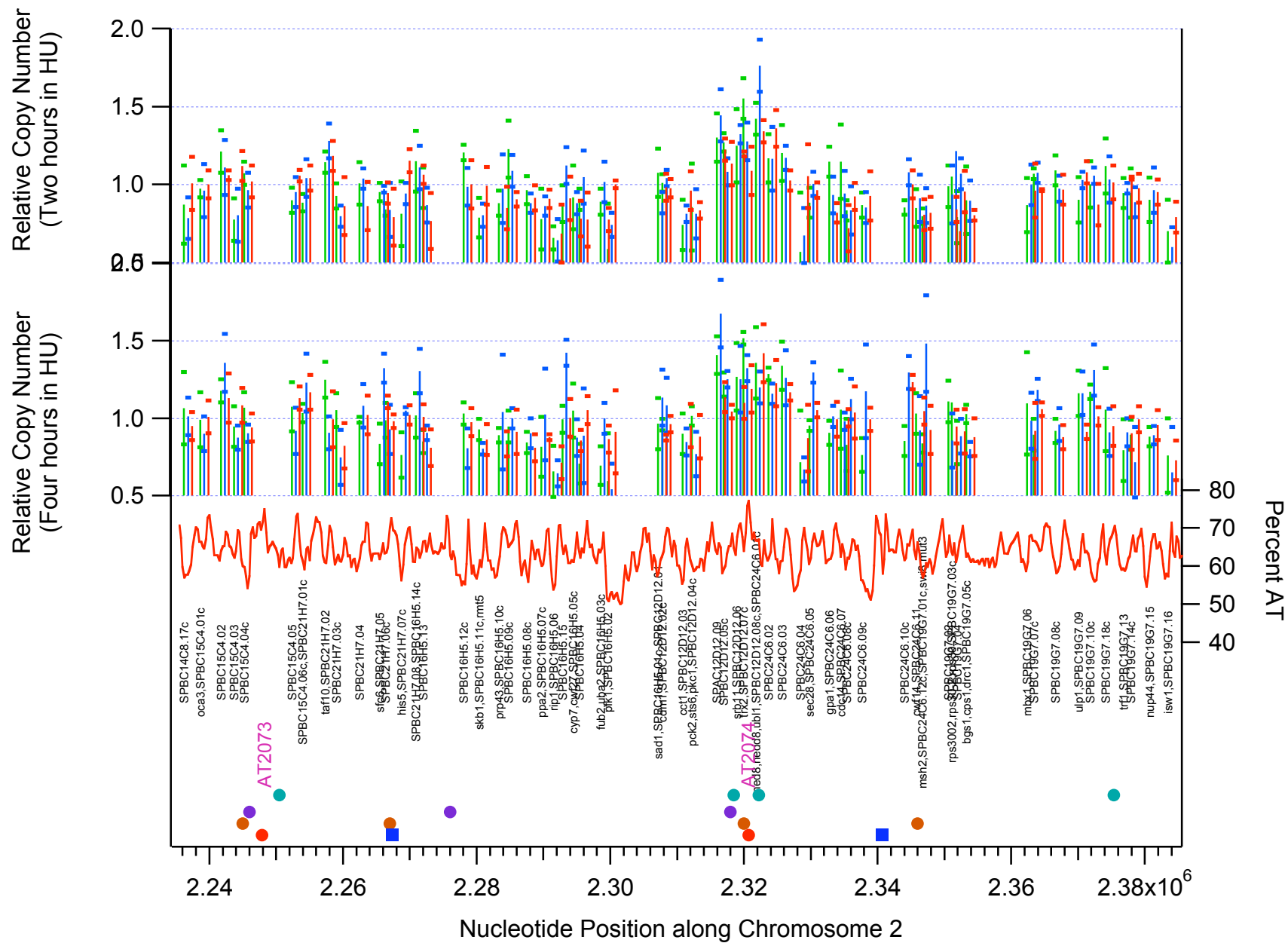

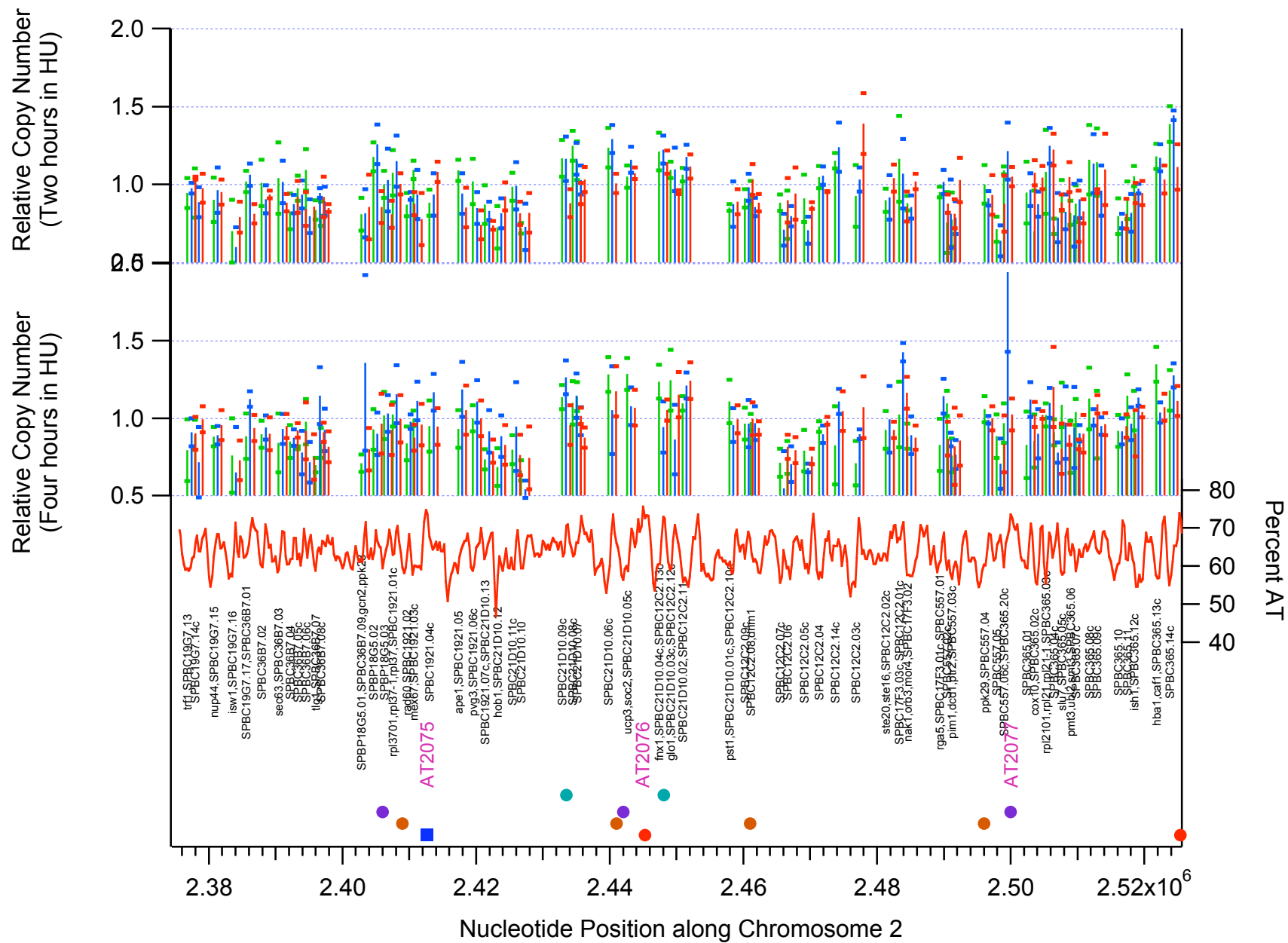

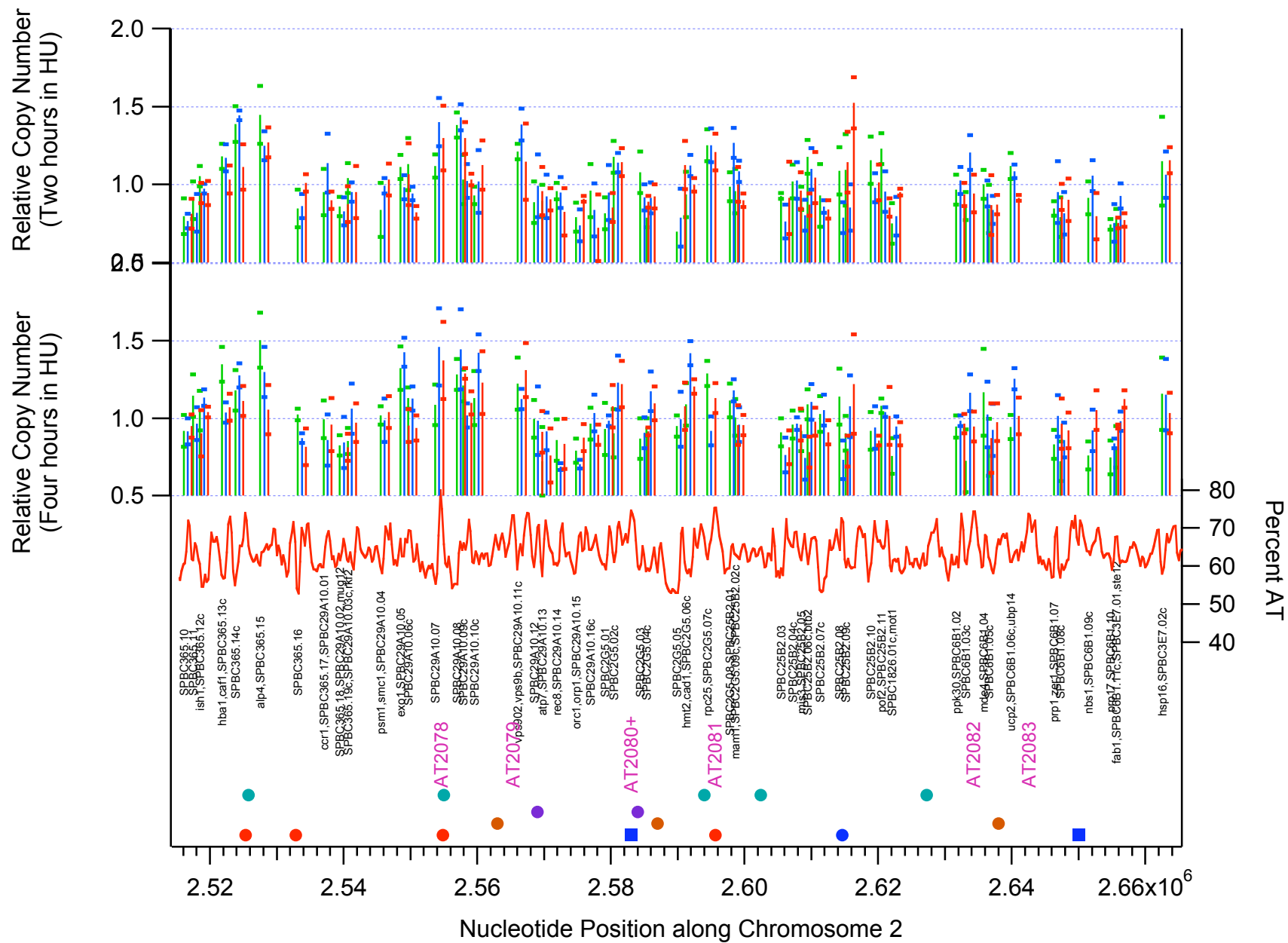

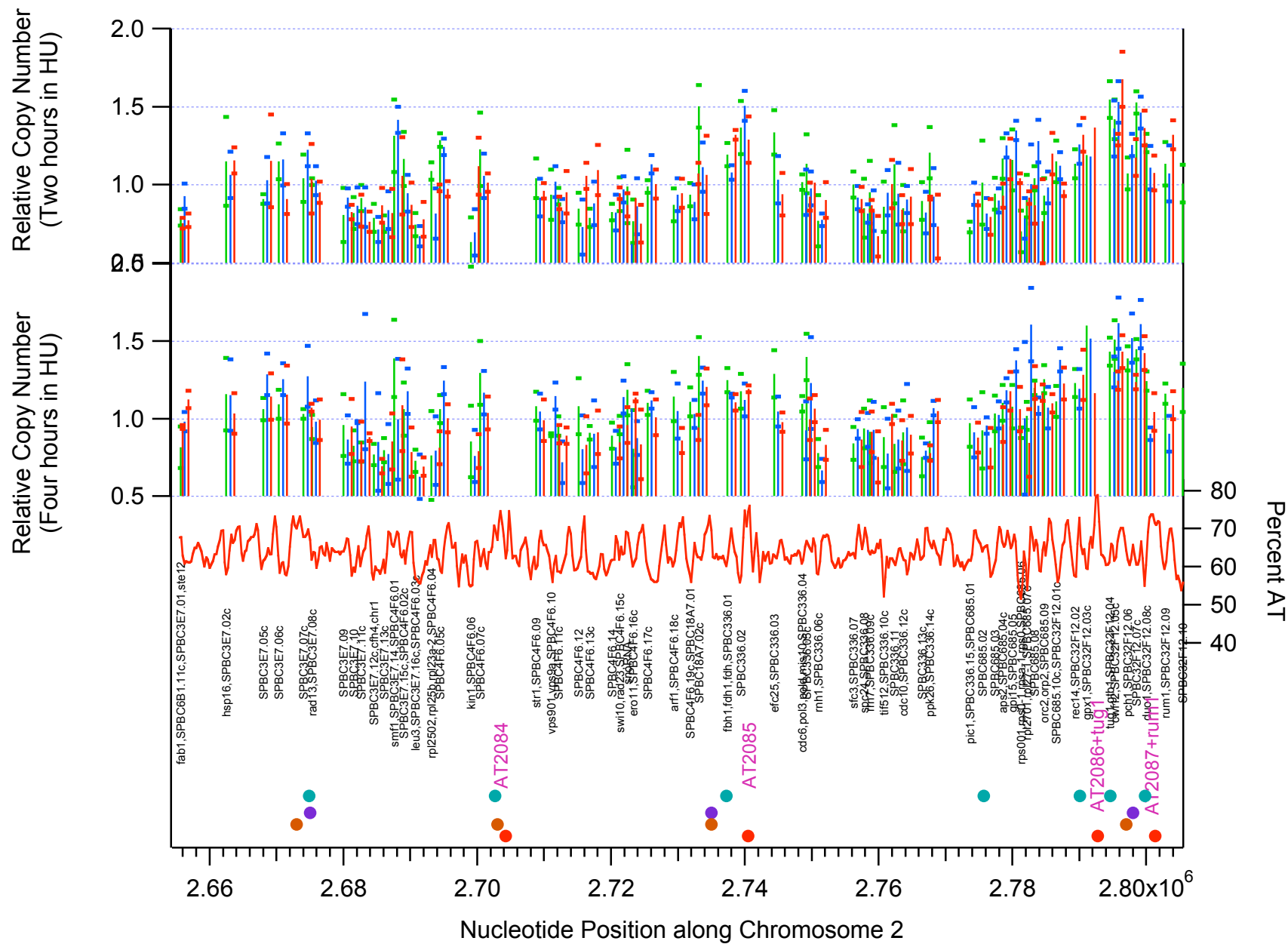

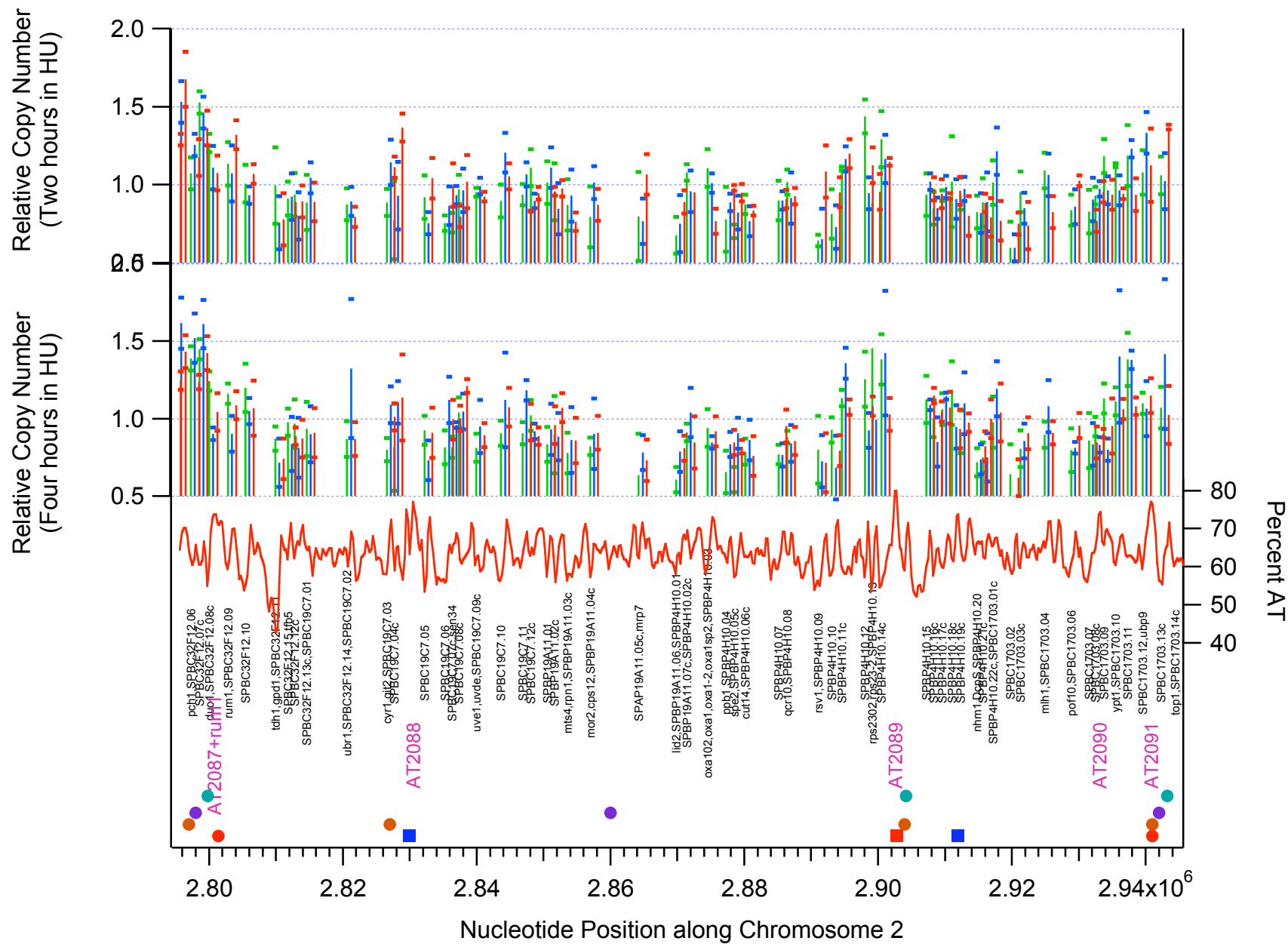

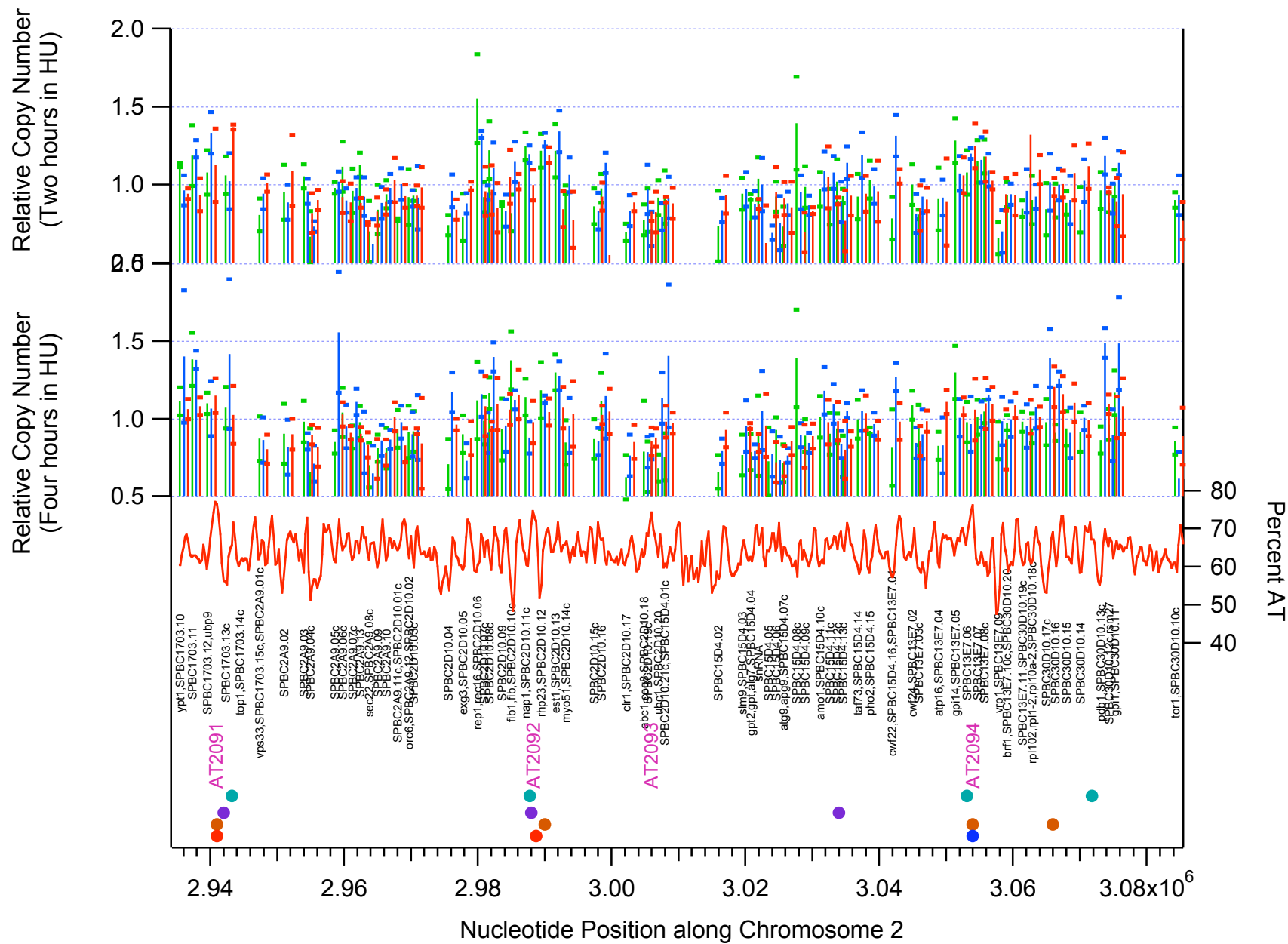

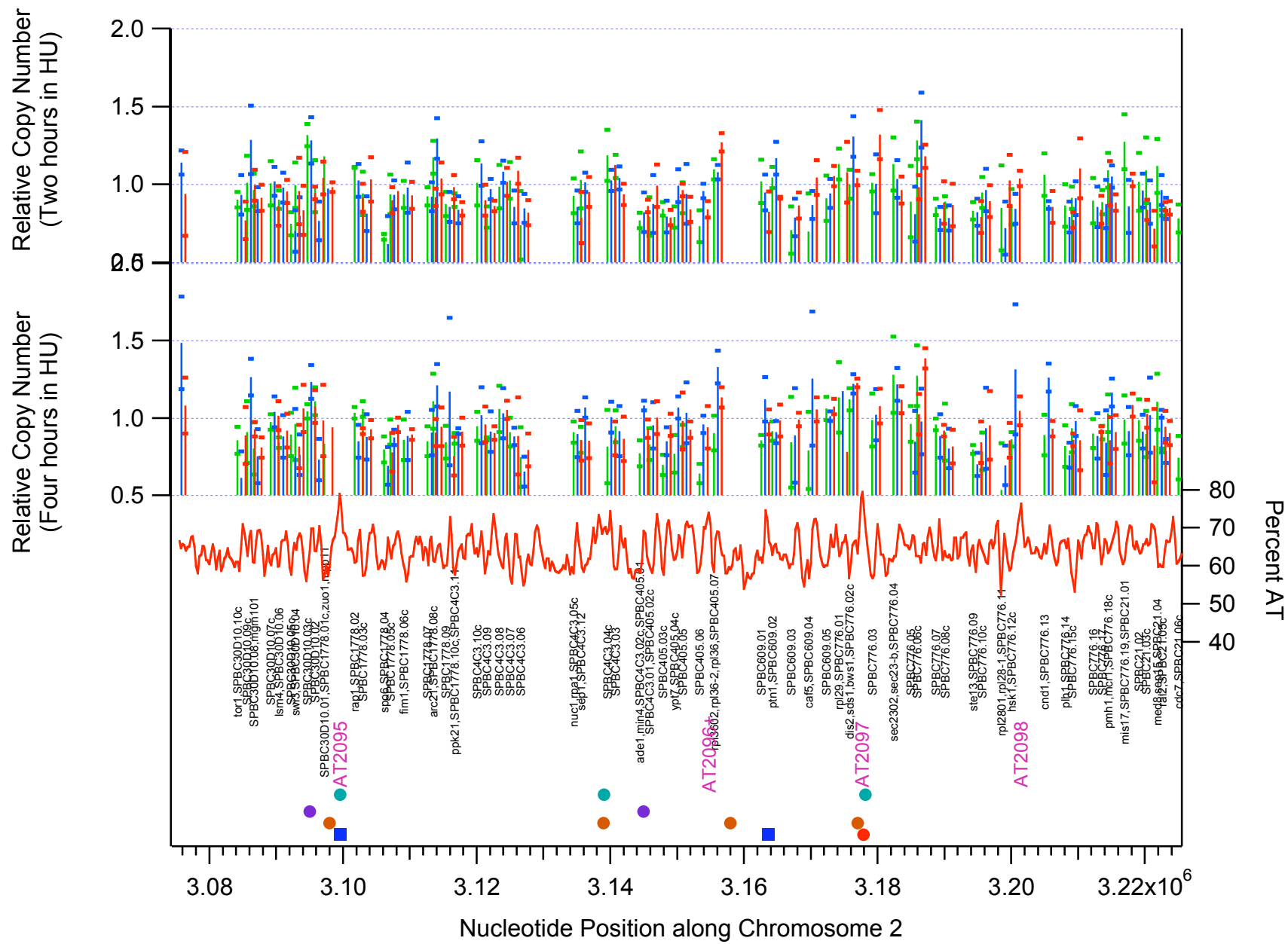

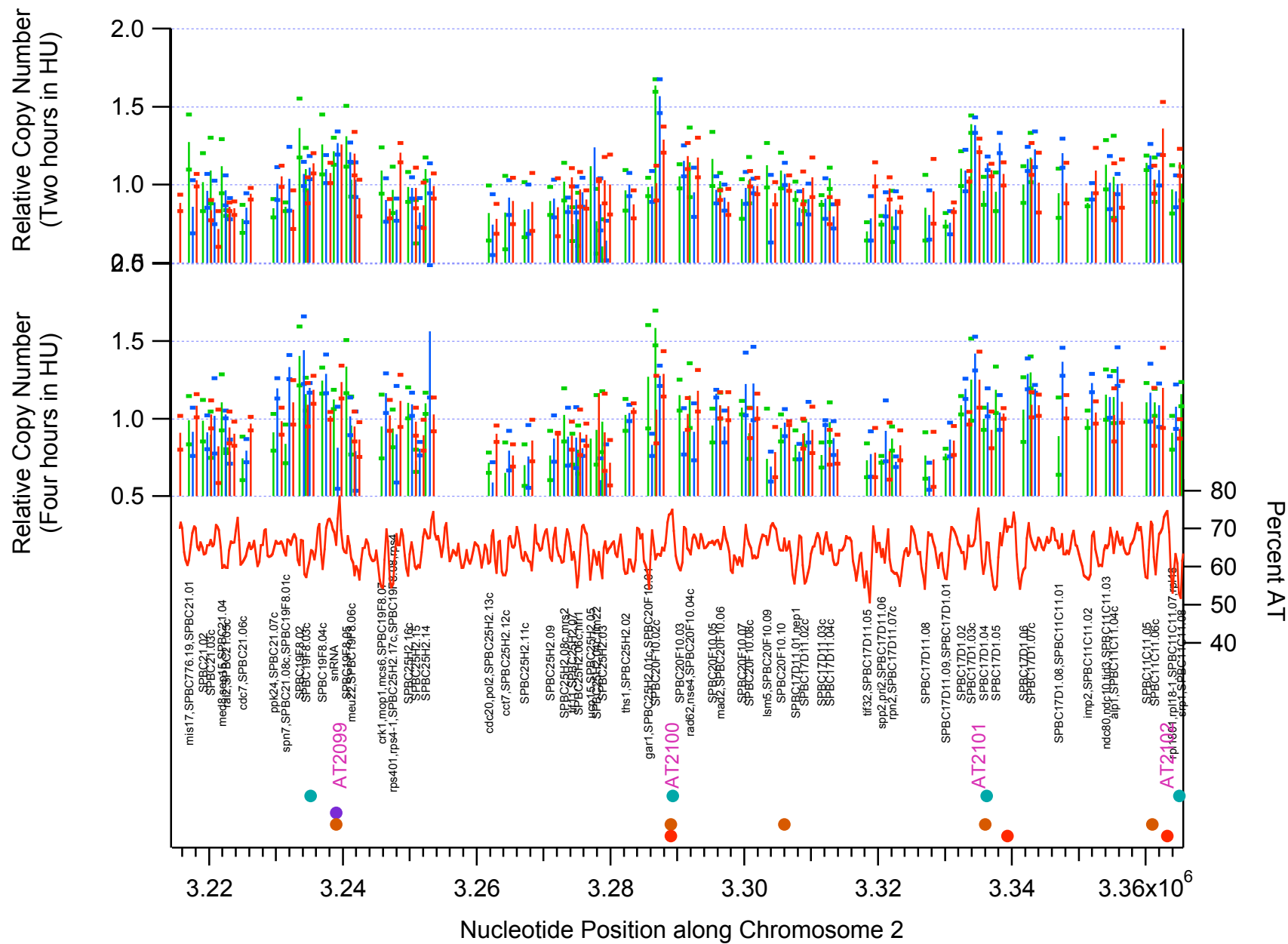







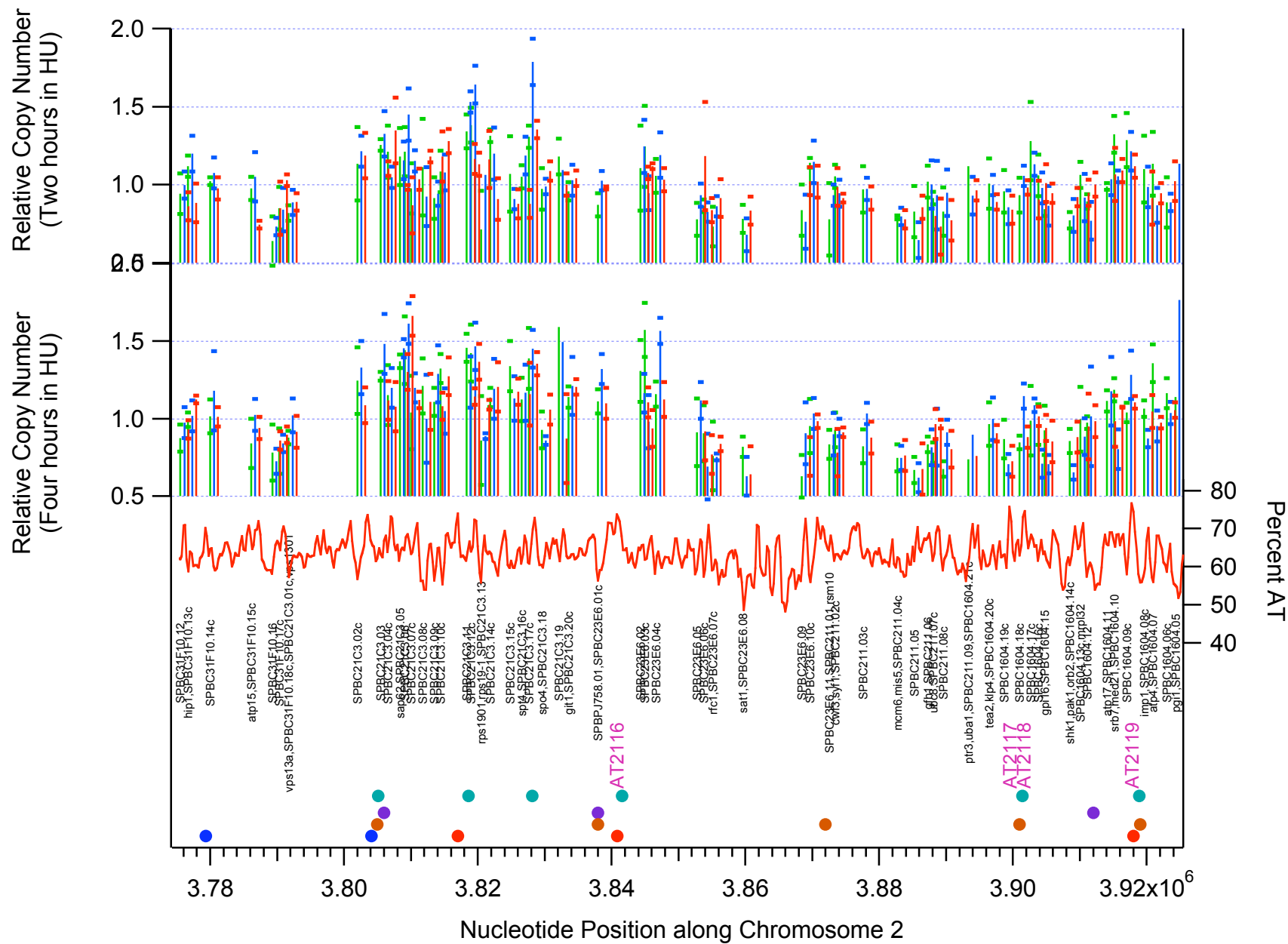

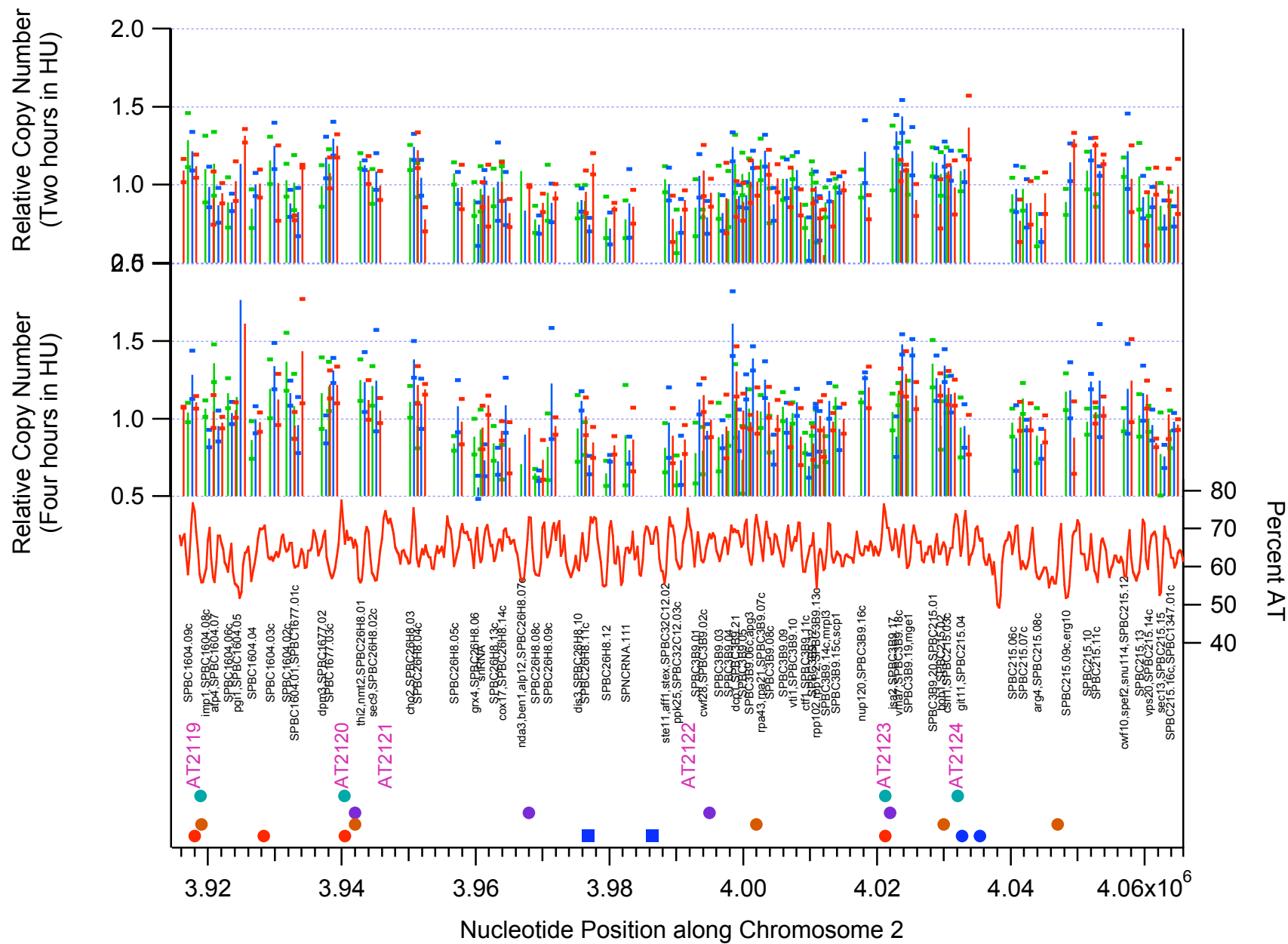

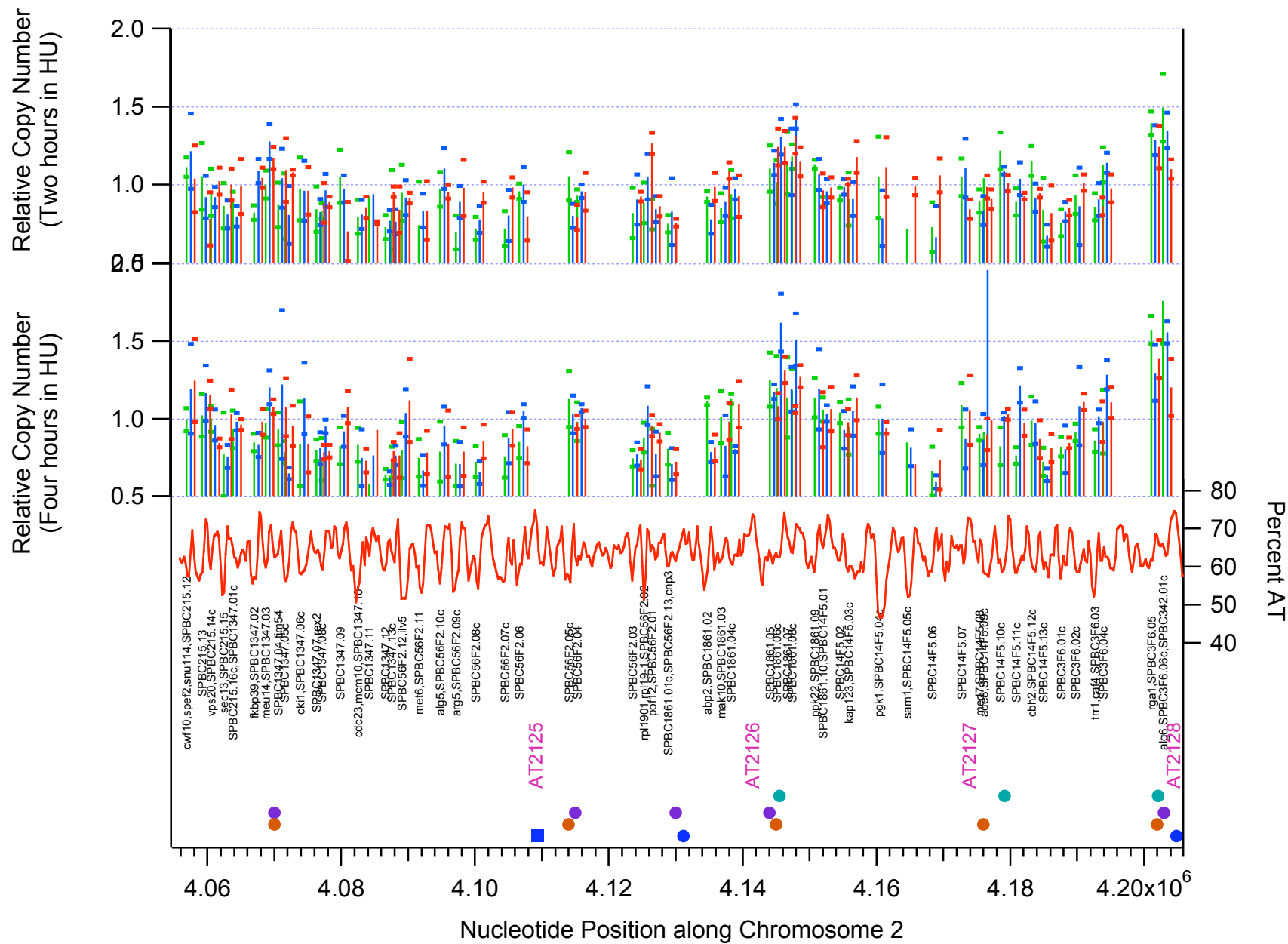



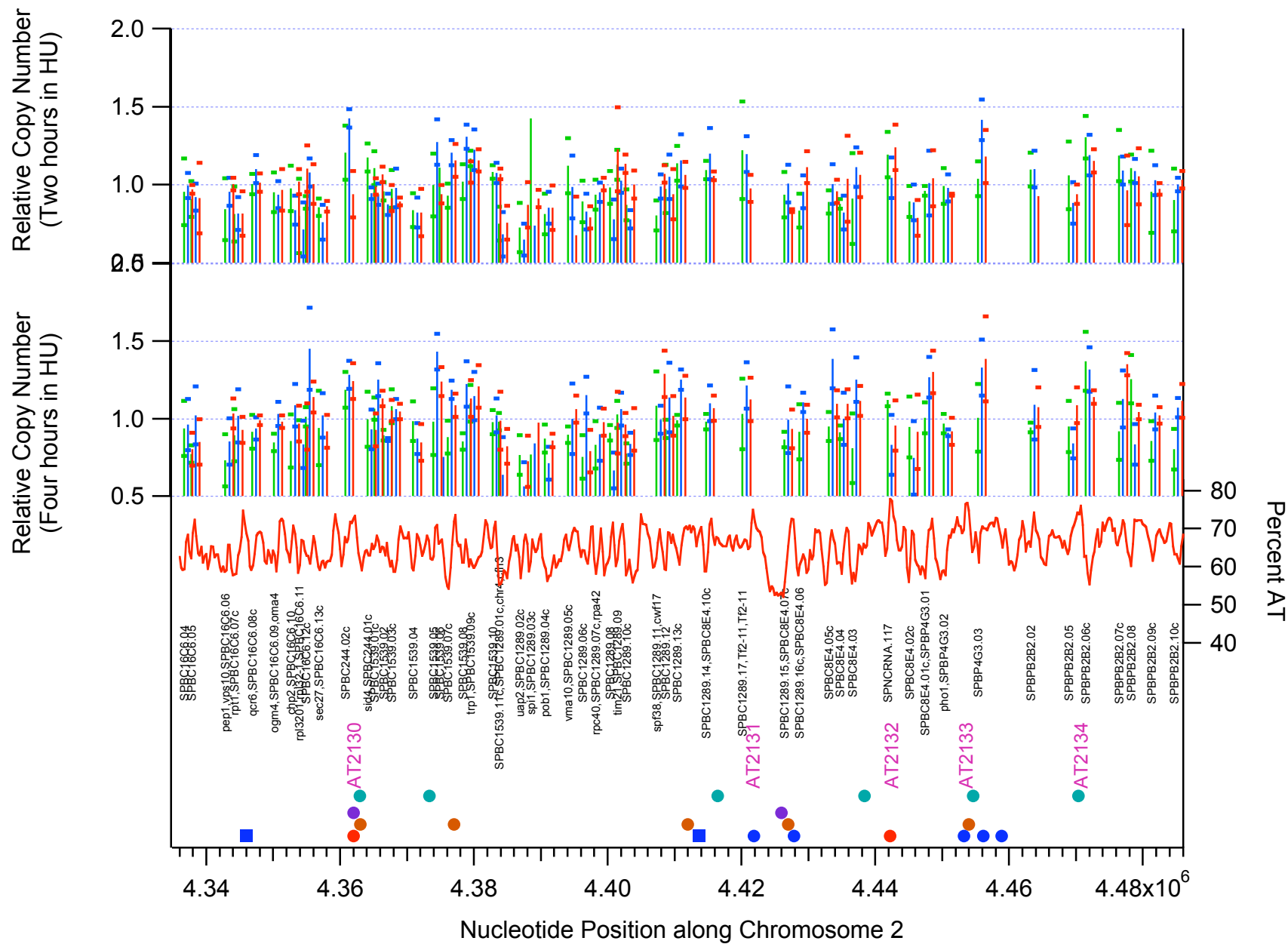

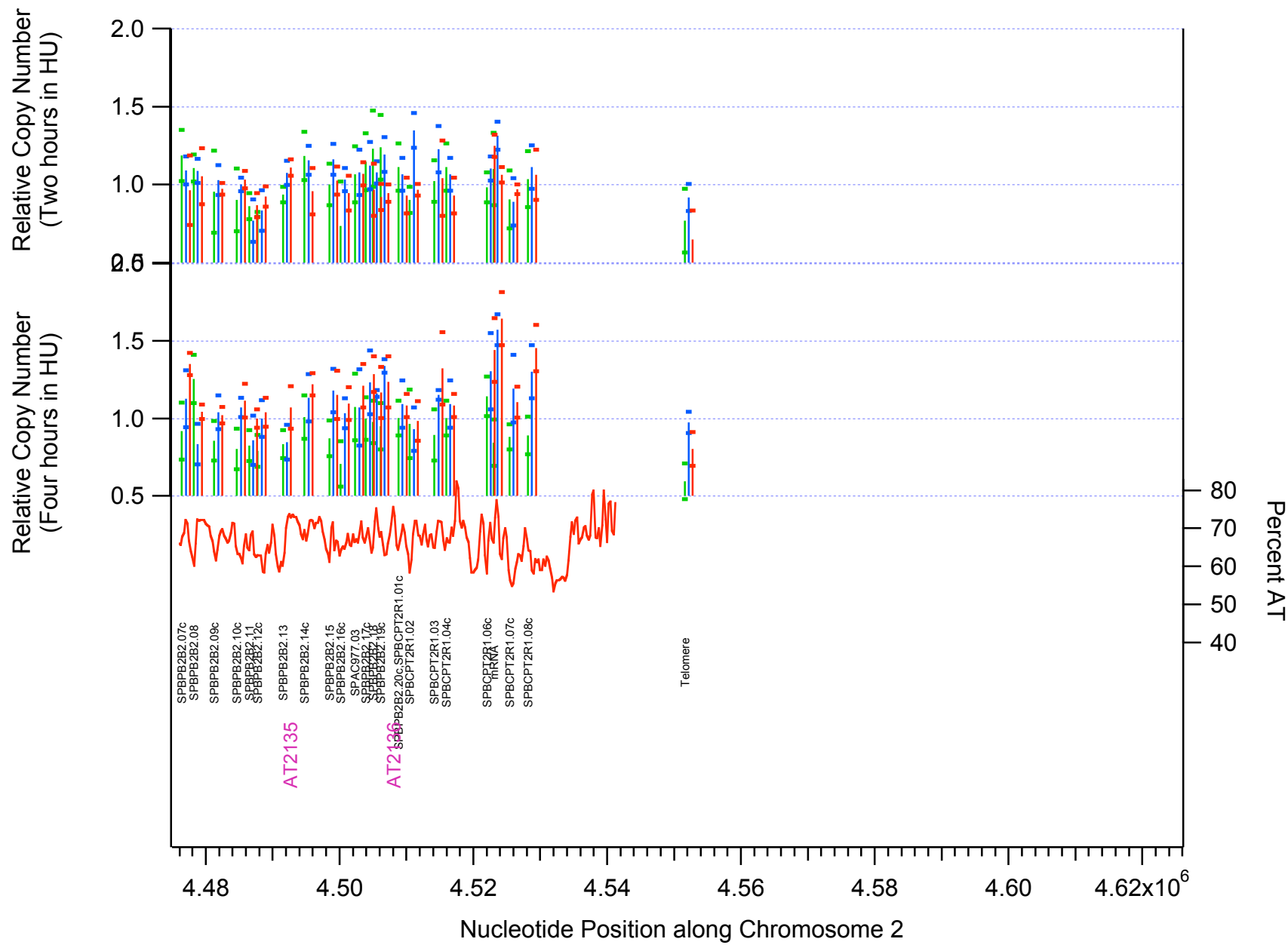

Supplement: Additional file 2 — Graphs of microarray measurements of copy number changes throughout chromosome 2. Similar to additional file 1, but for chromosome 2 [file 1471-2199-8-112-S2.pdf]

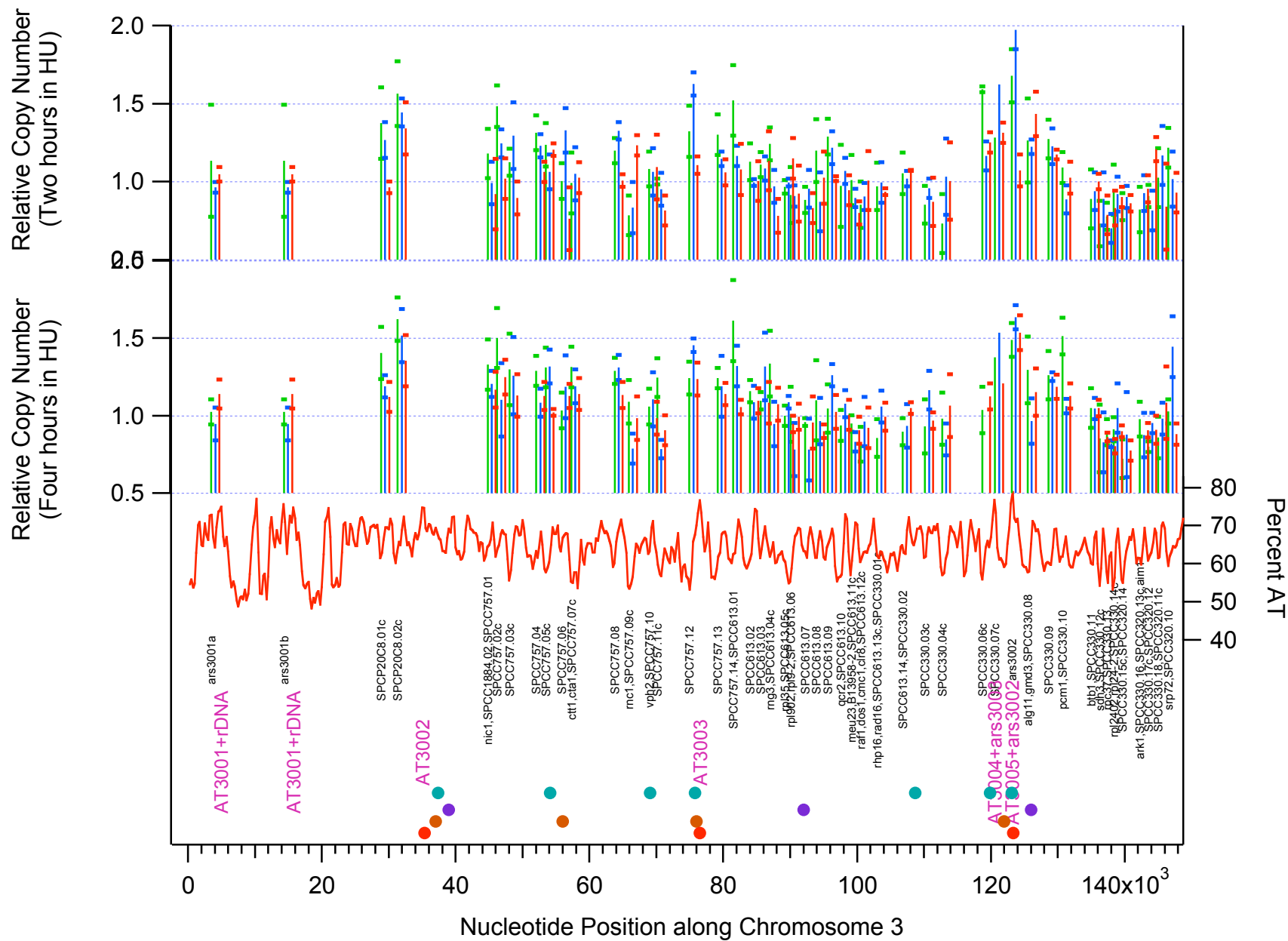

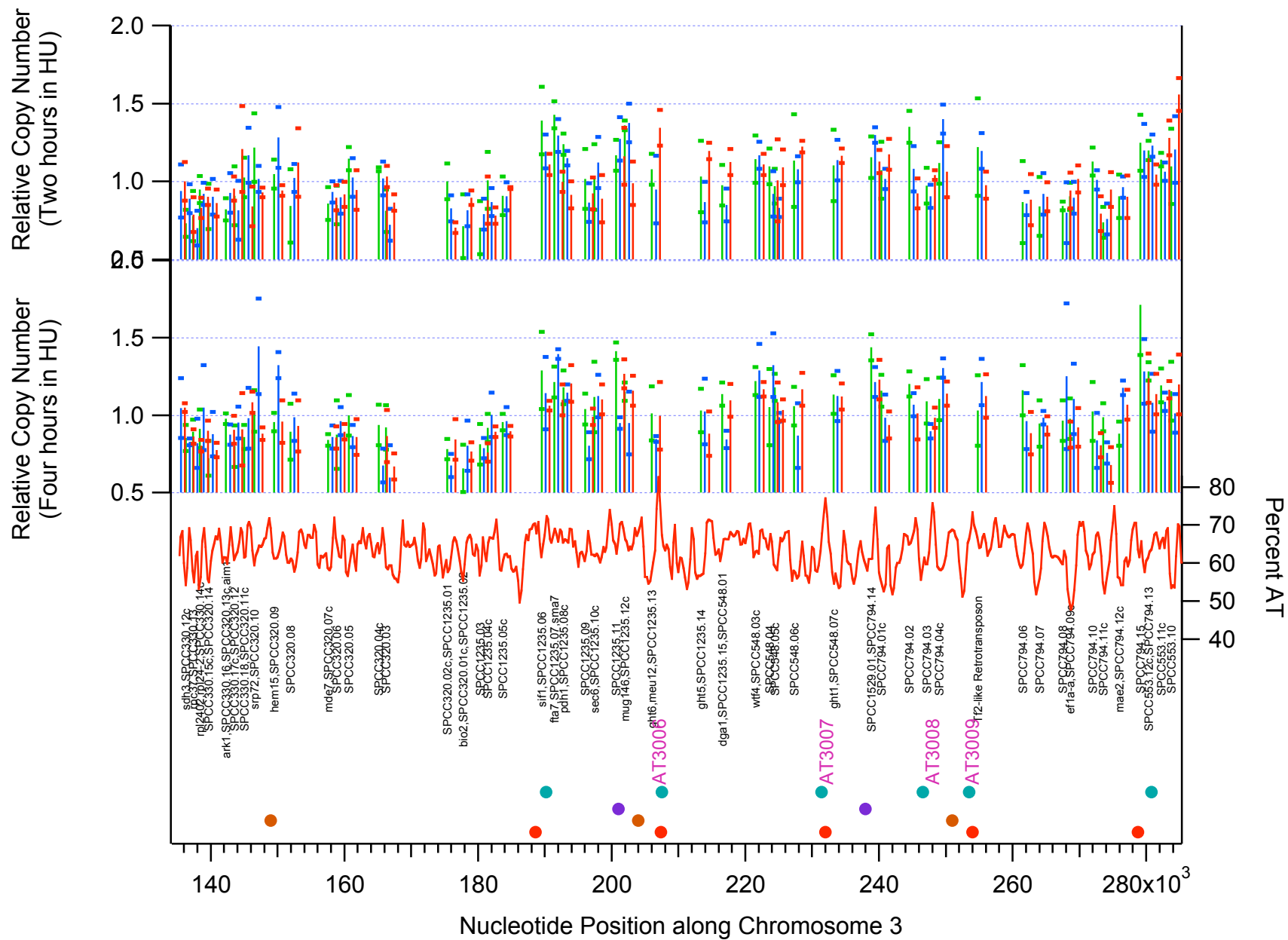

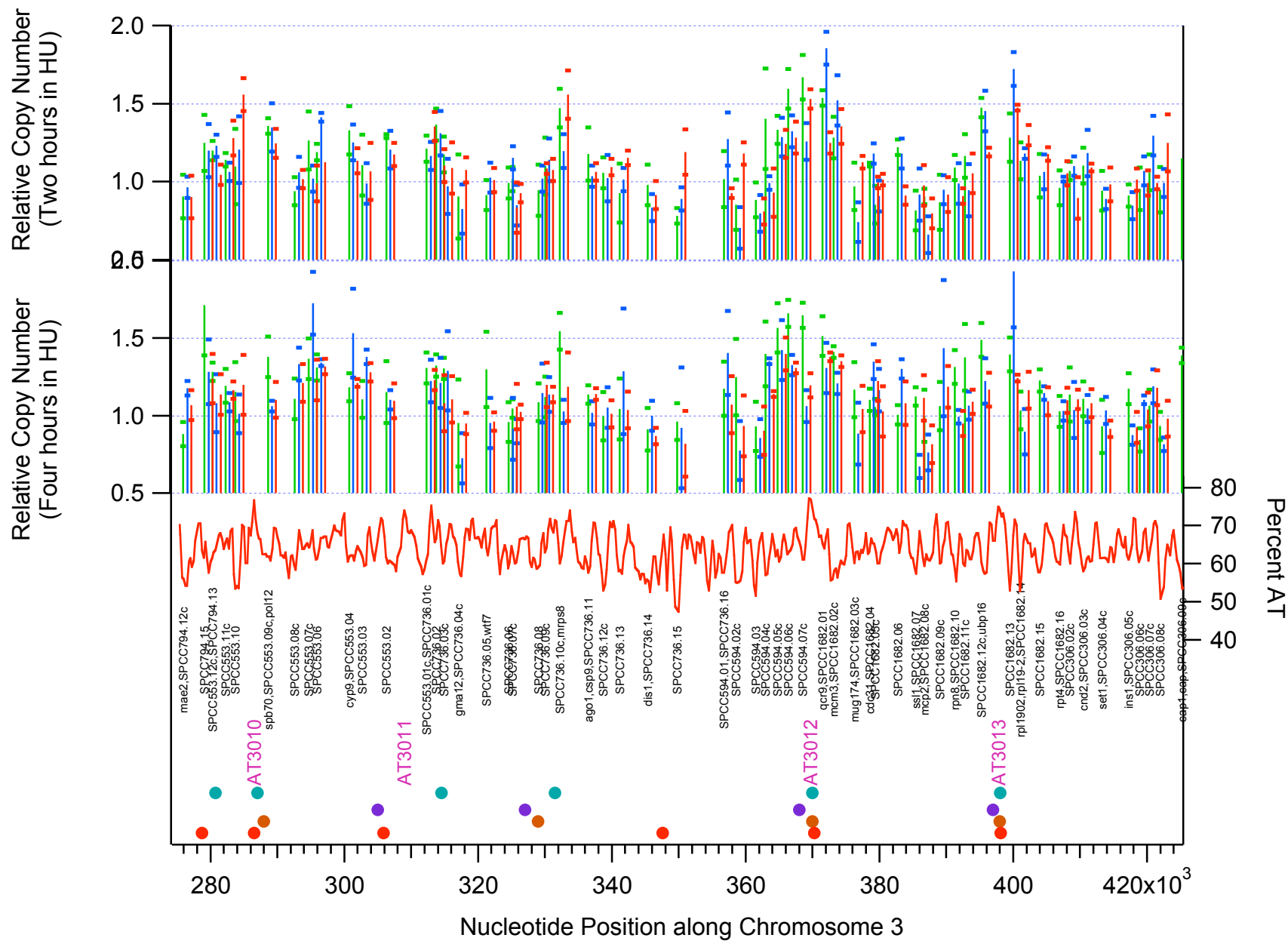

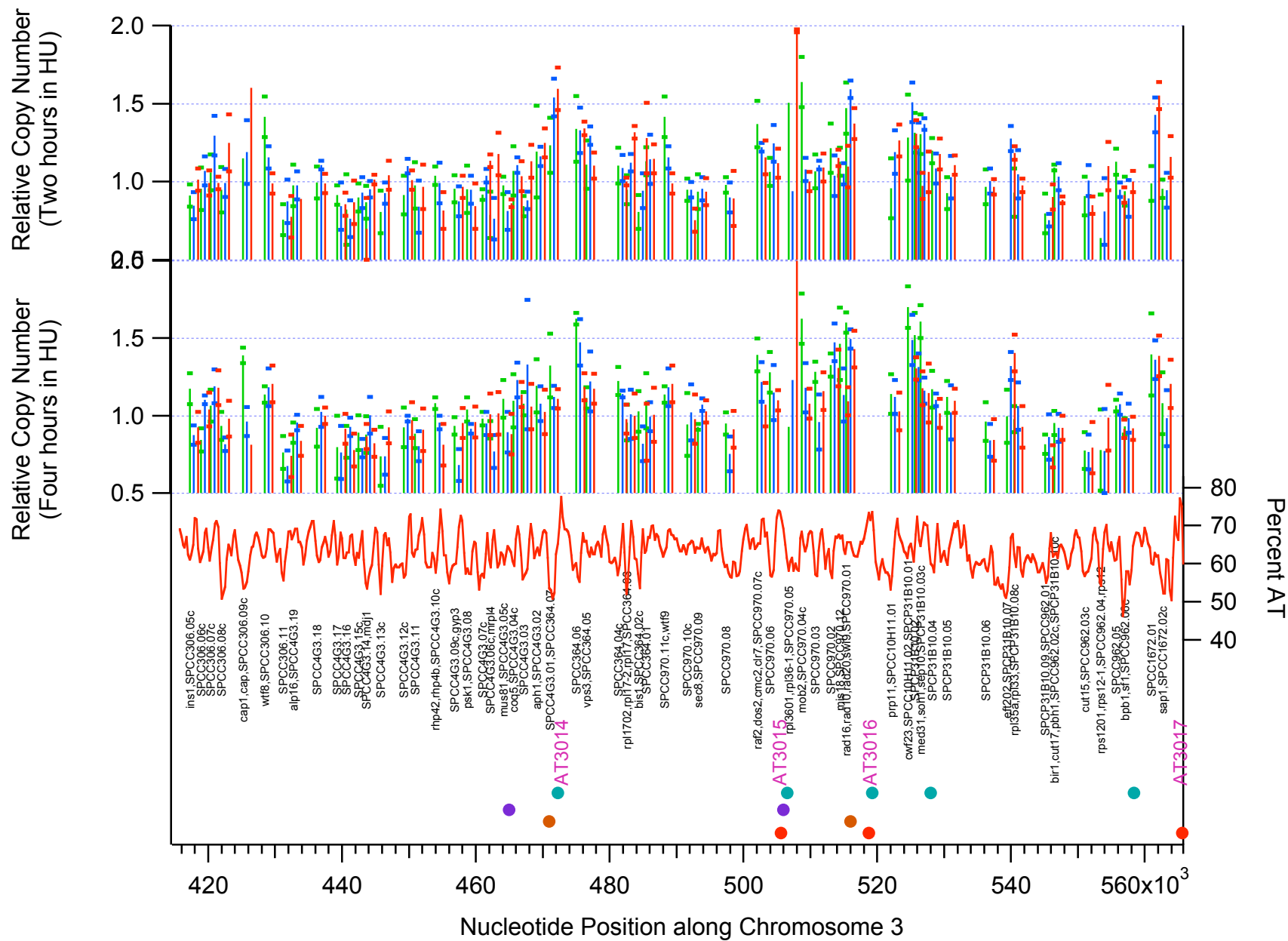

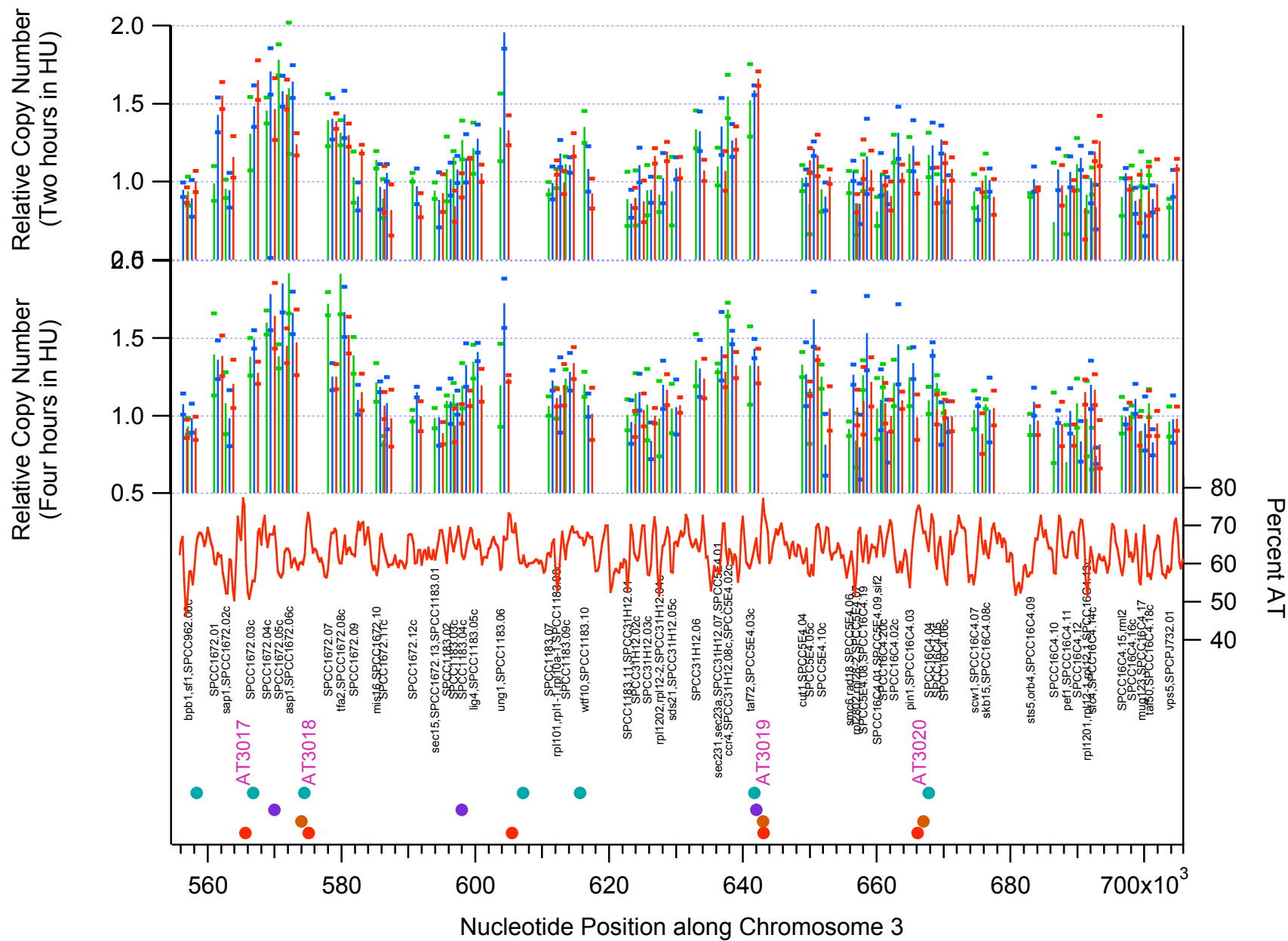

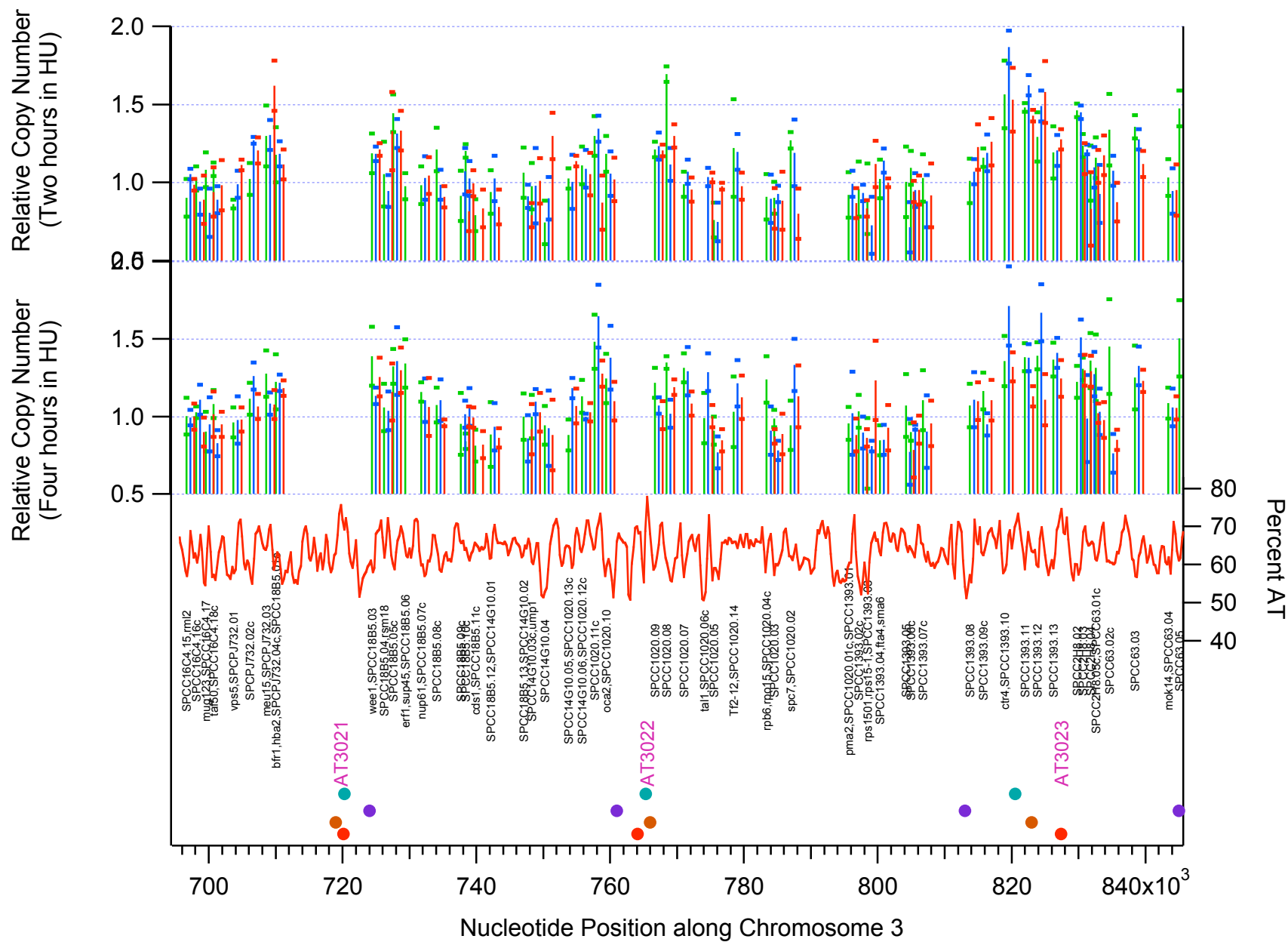

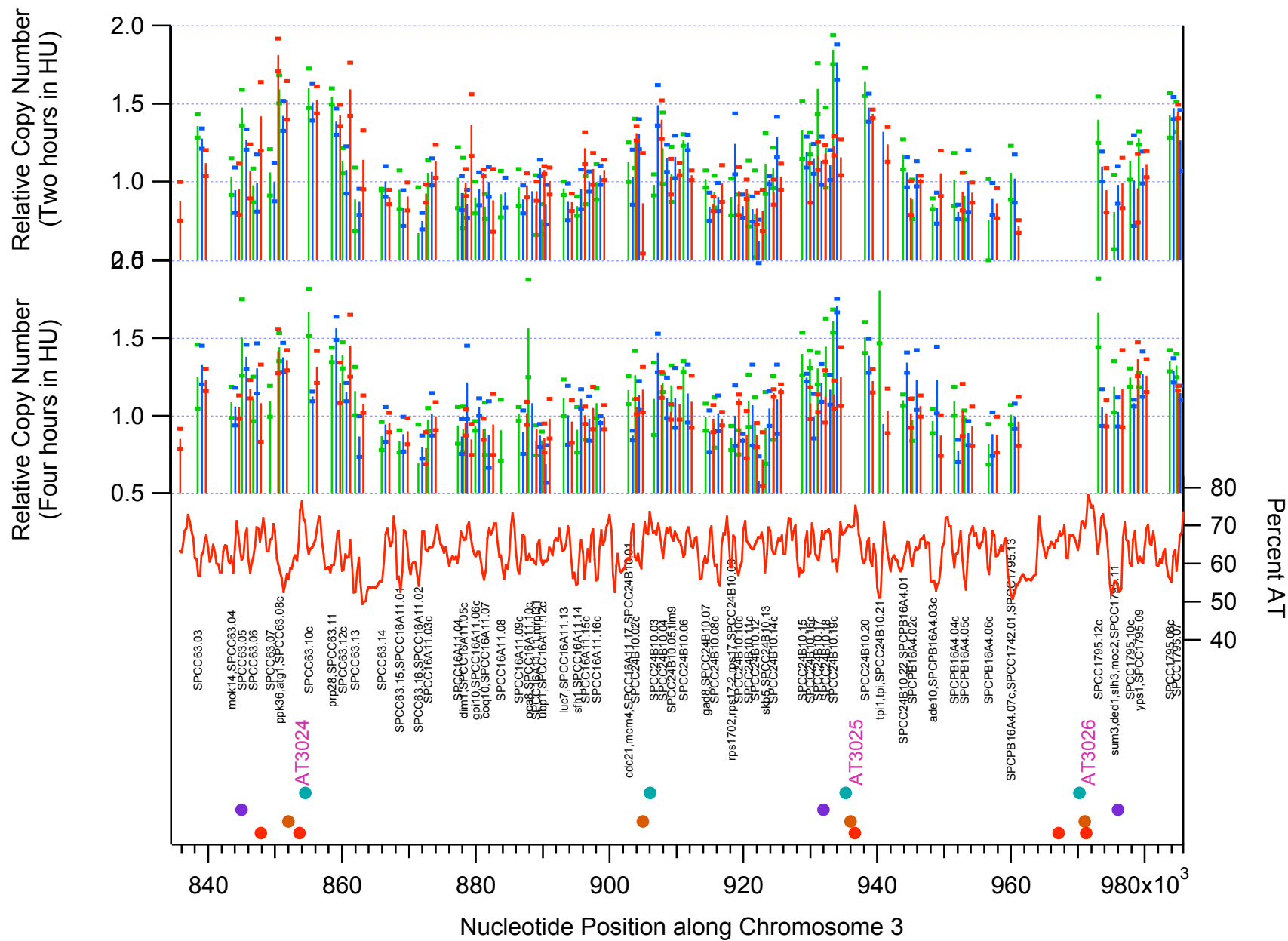

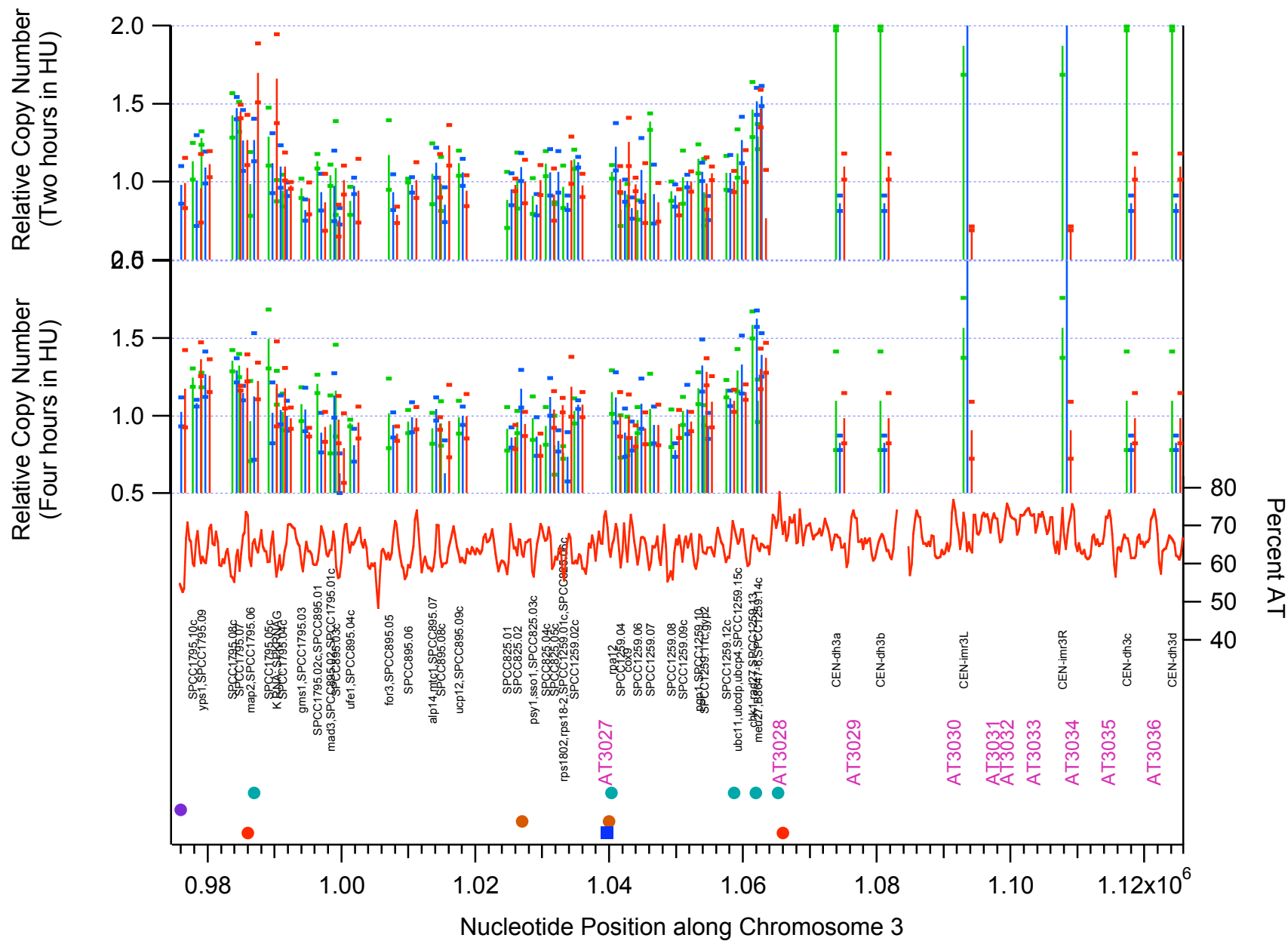

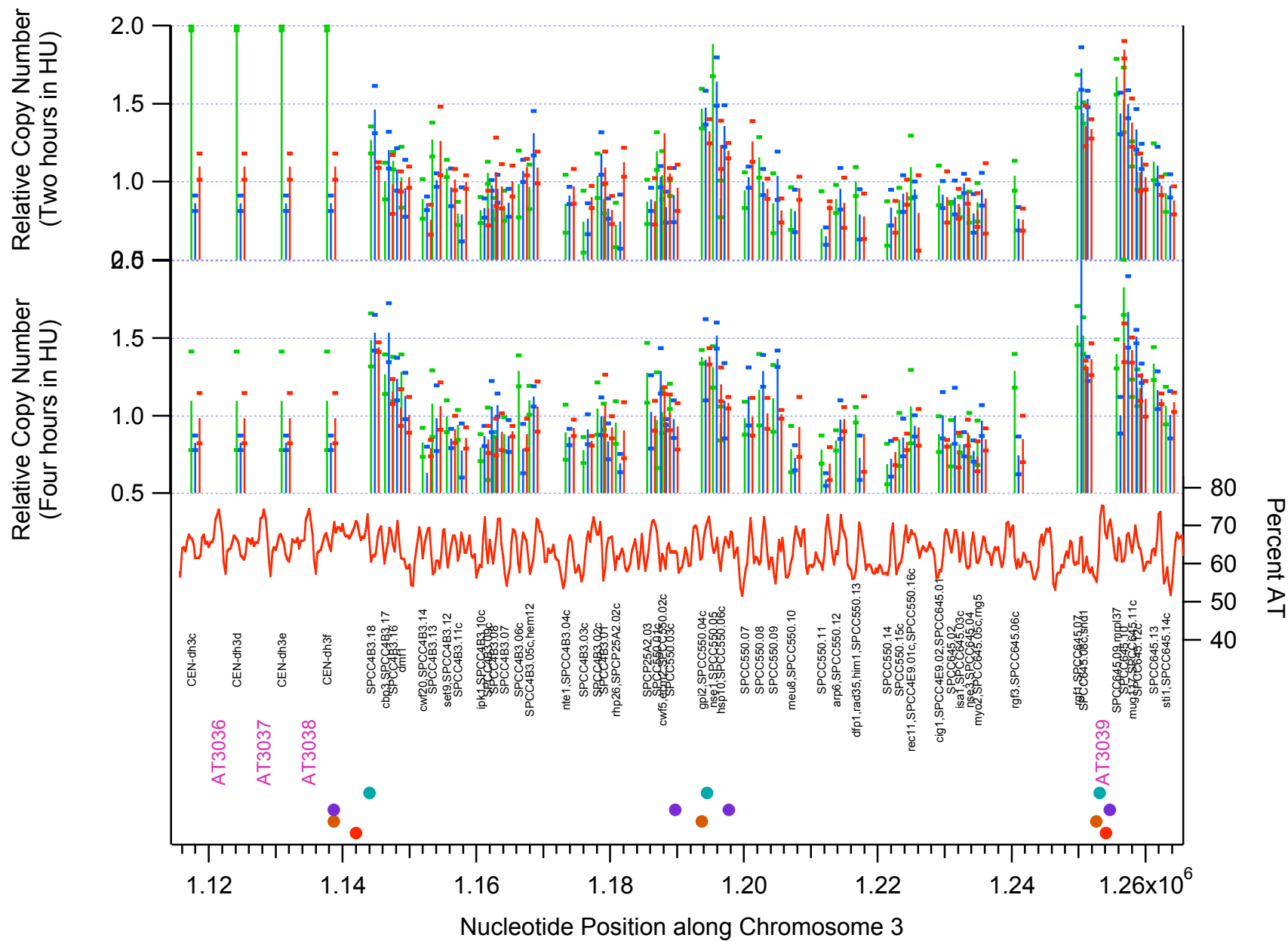

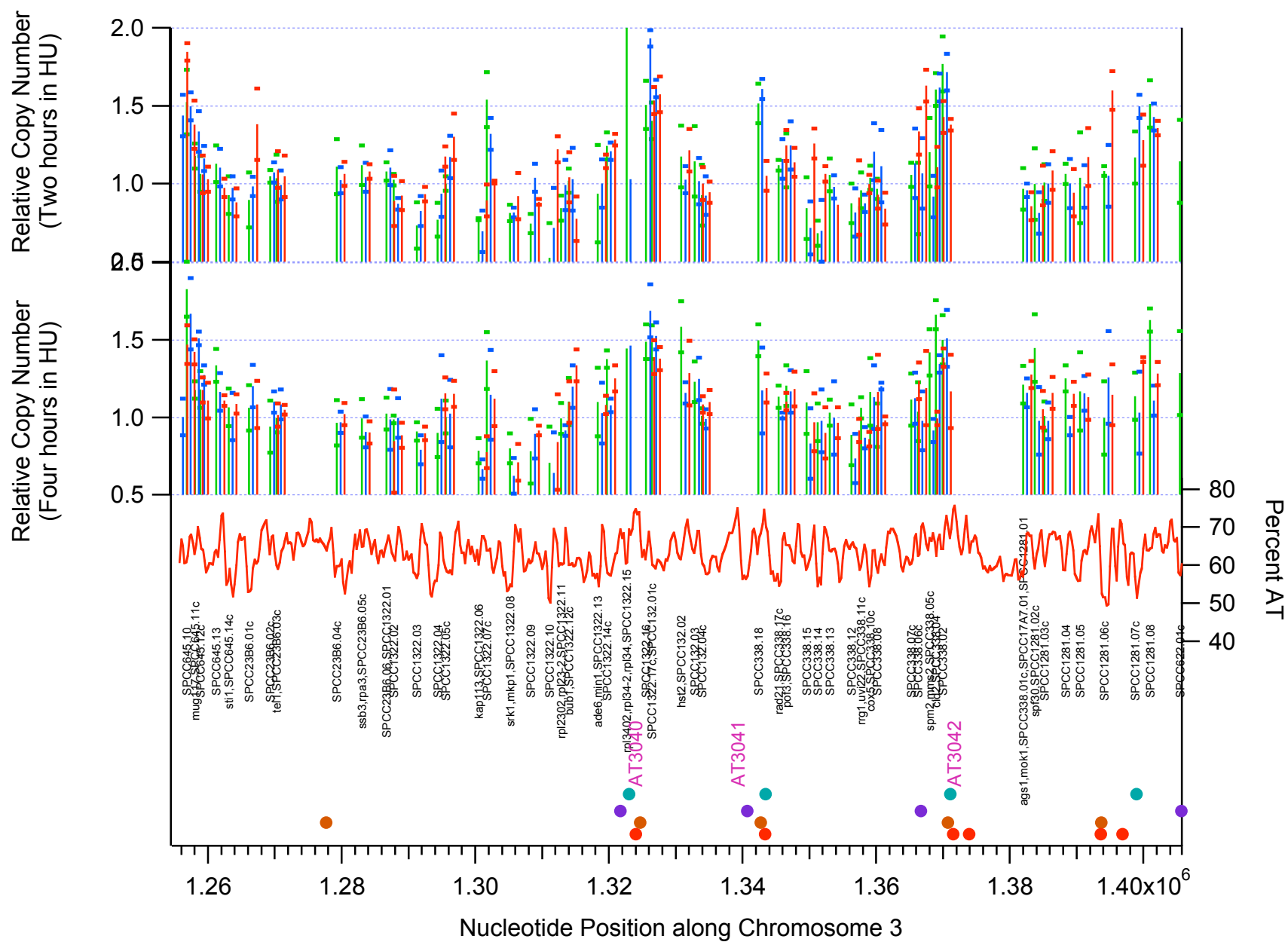

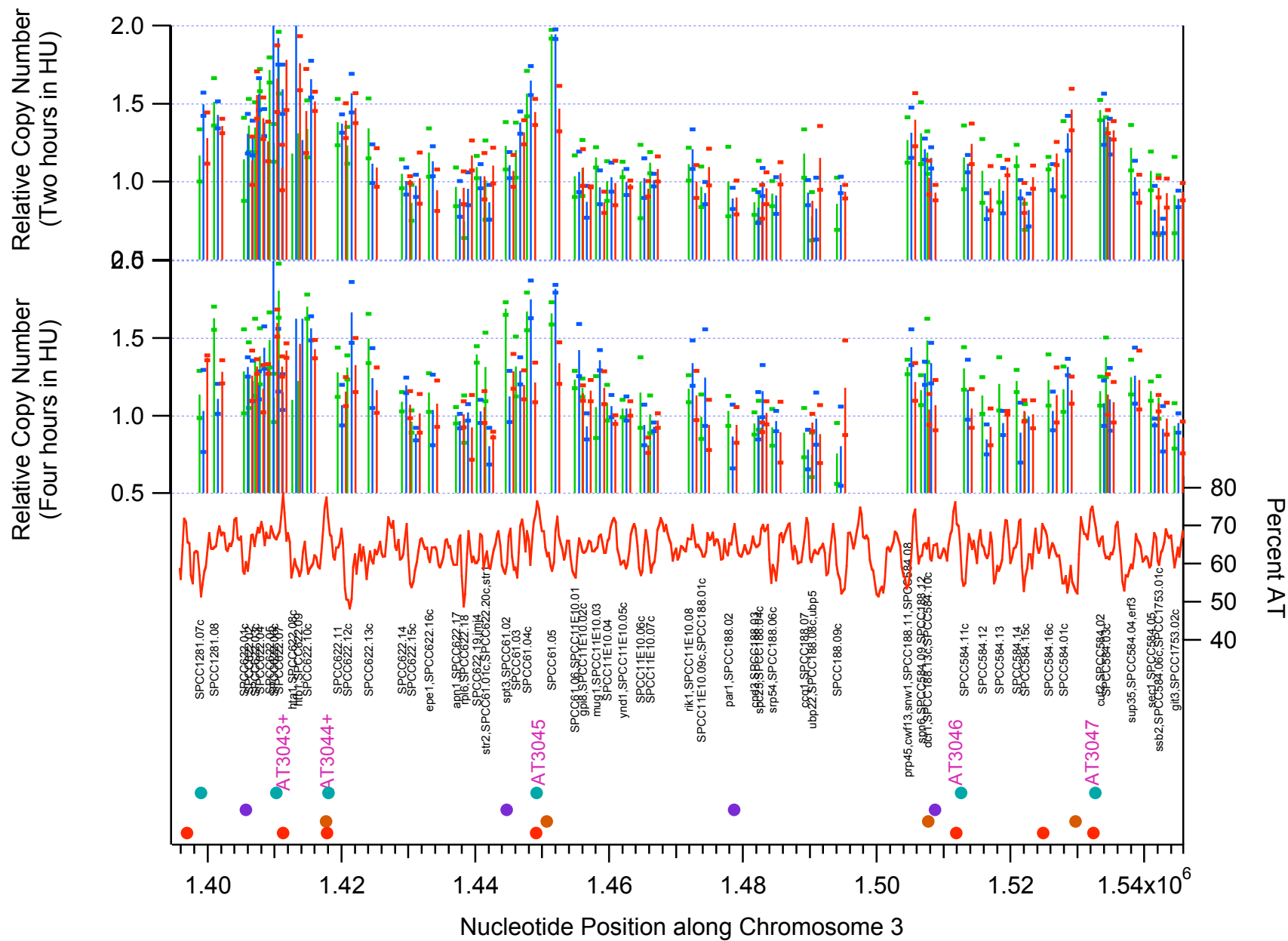

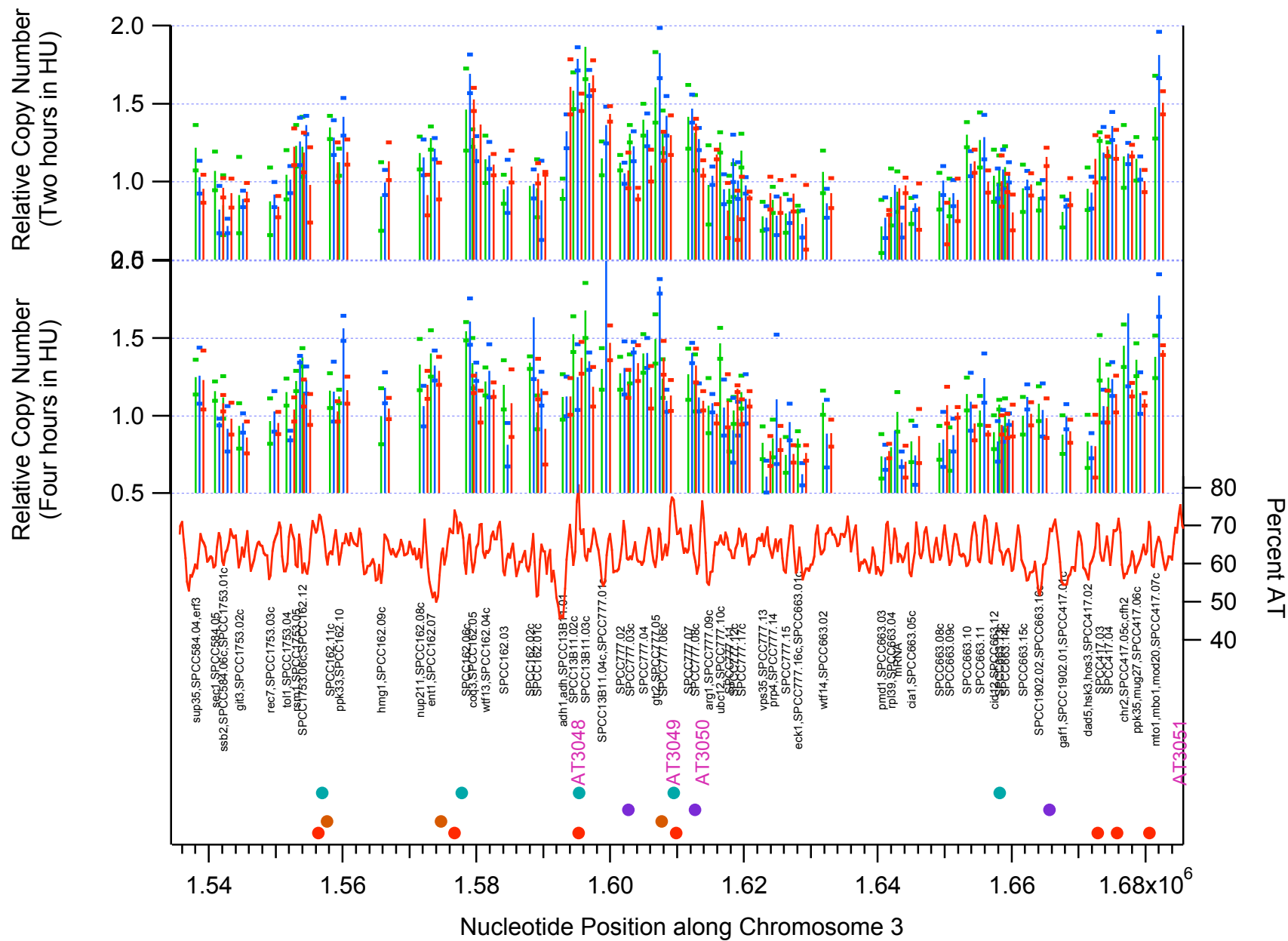

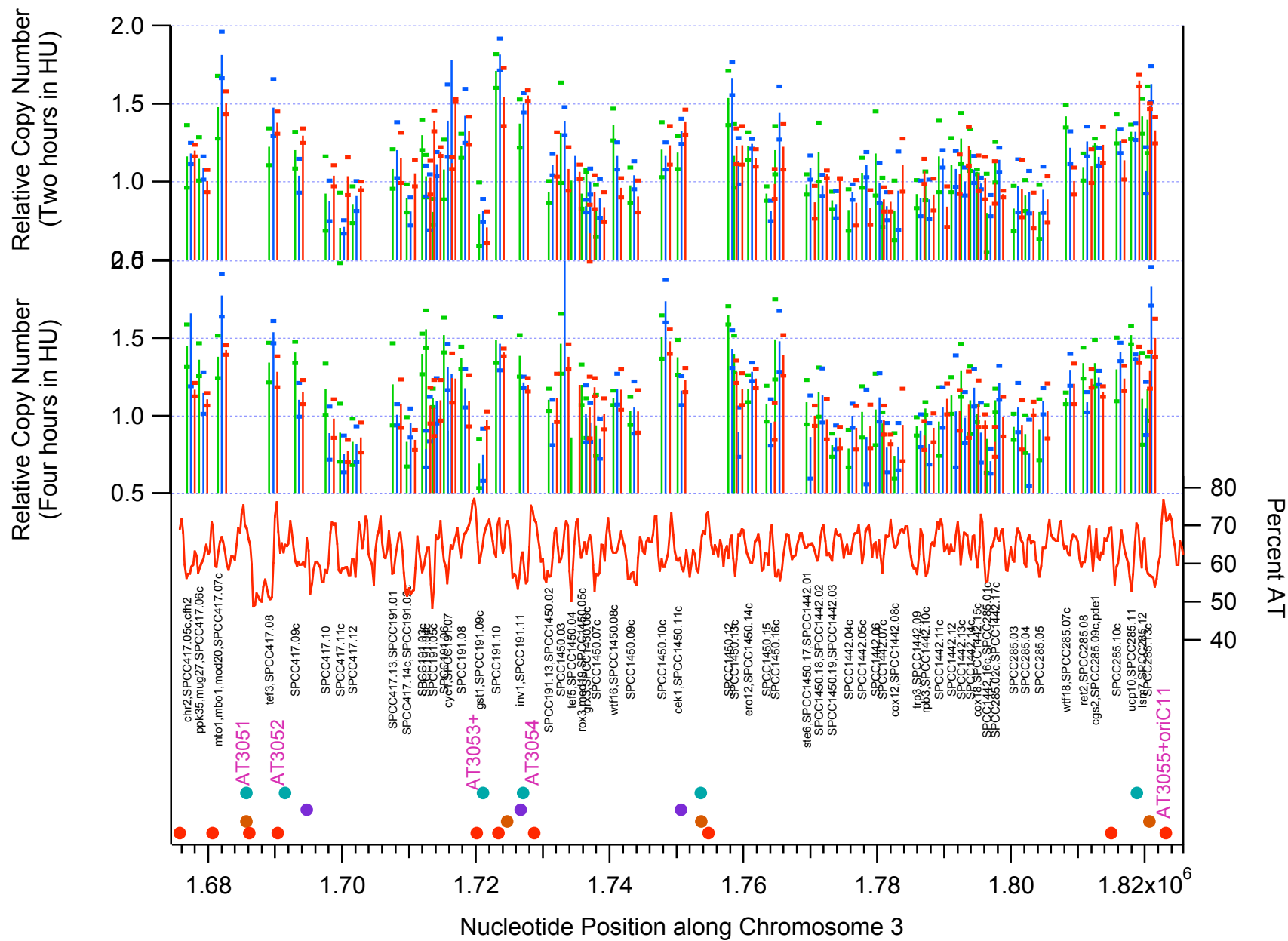

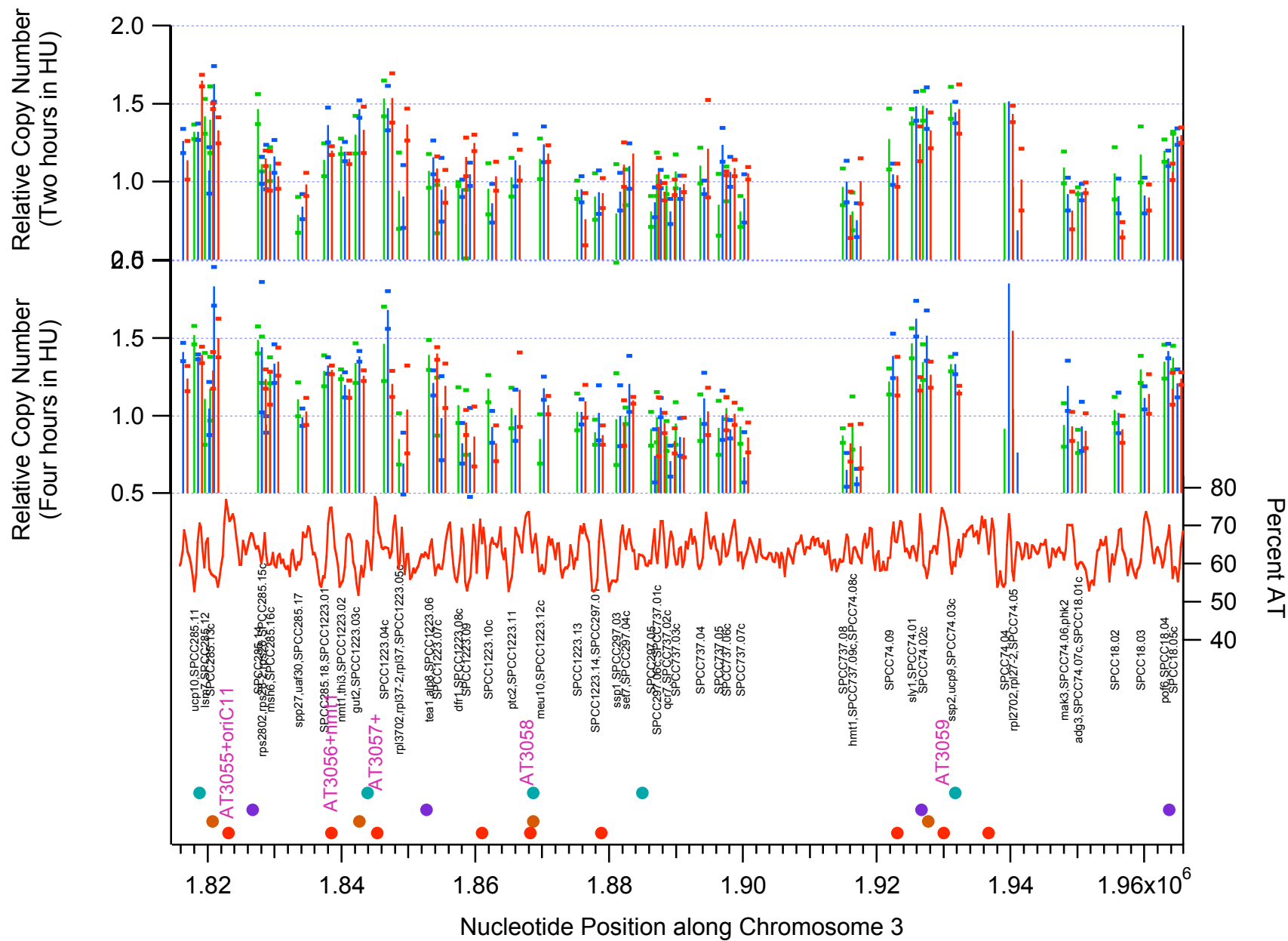

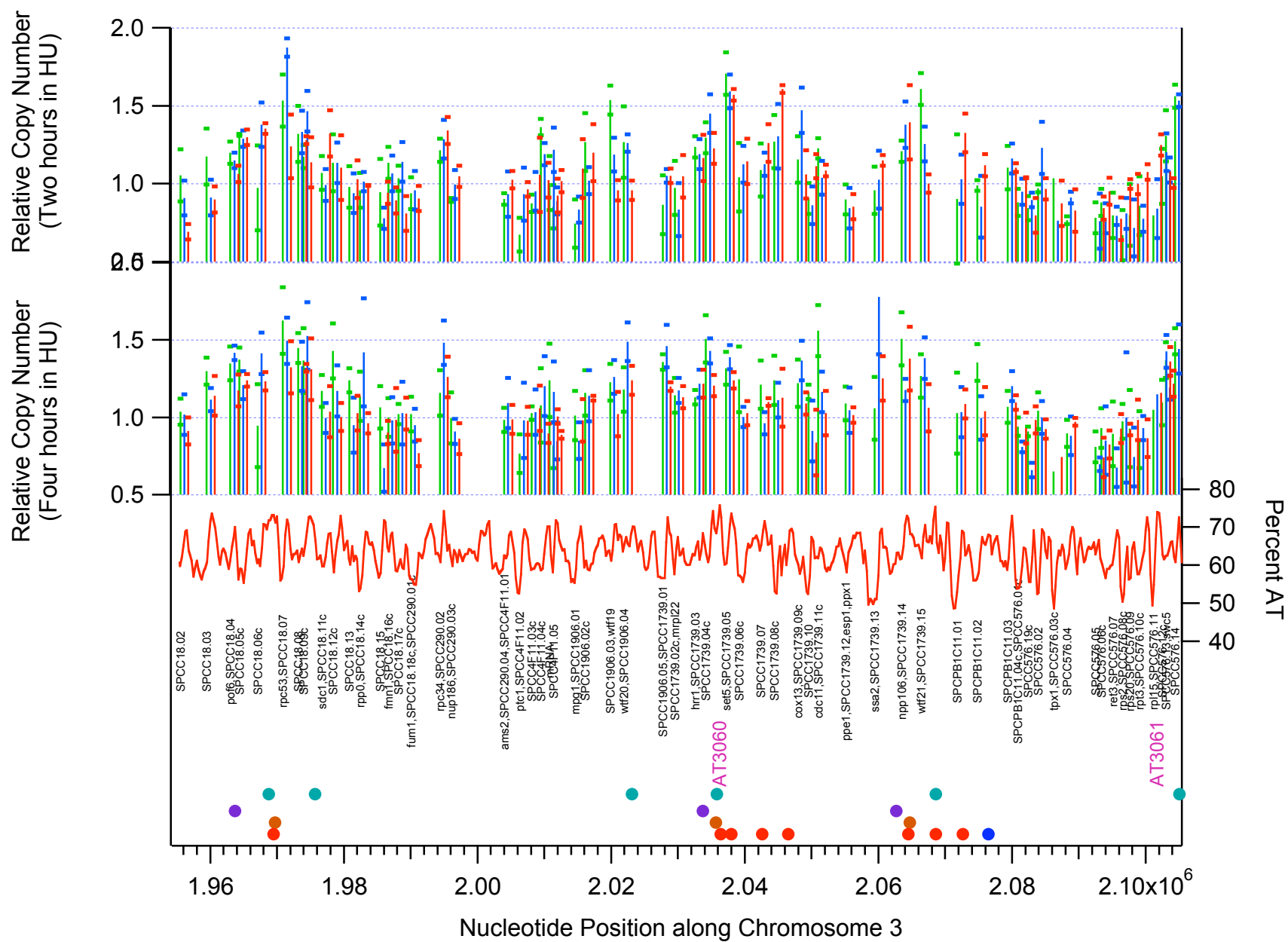

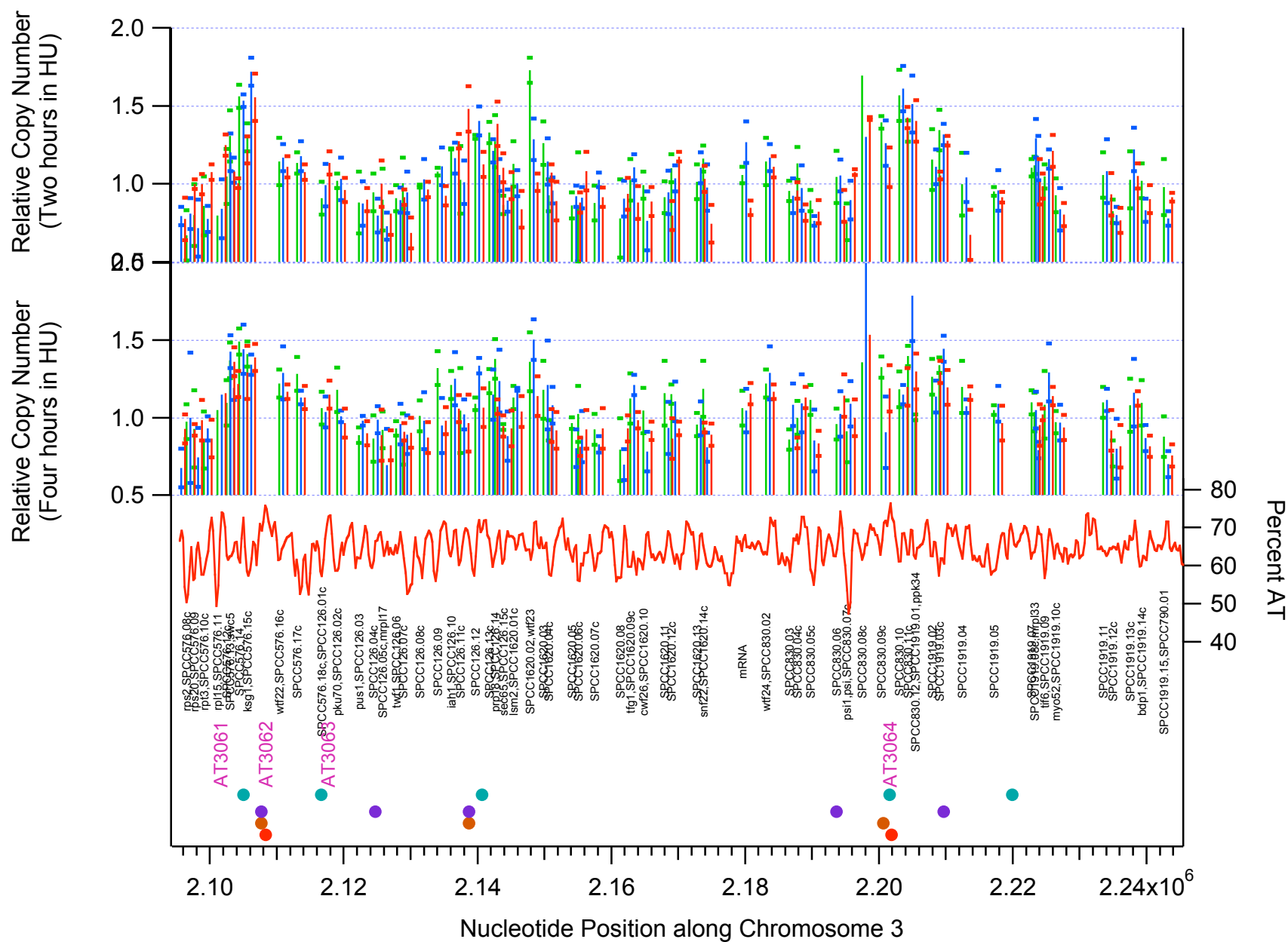

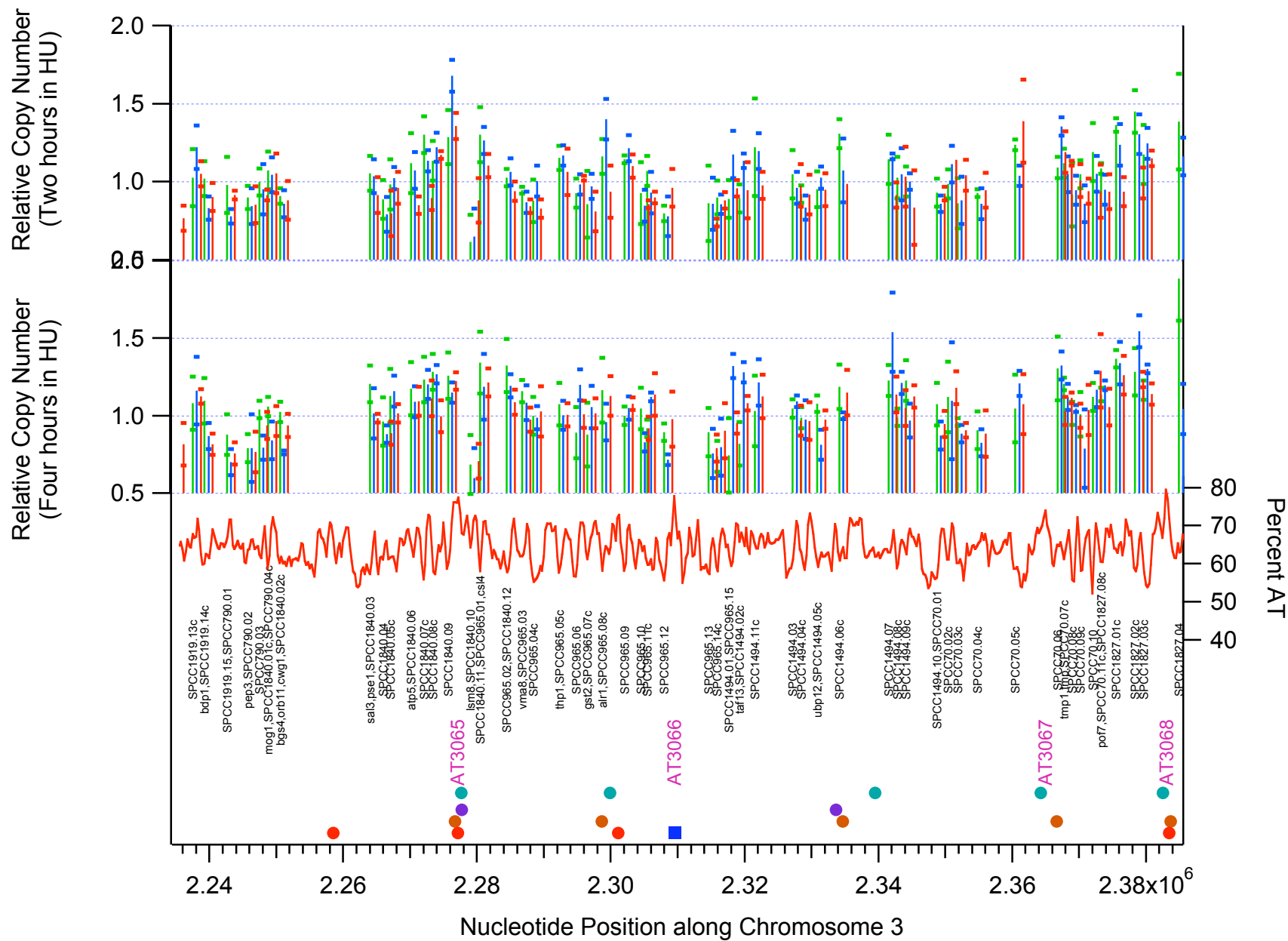

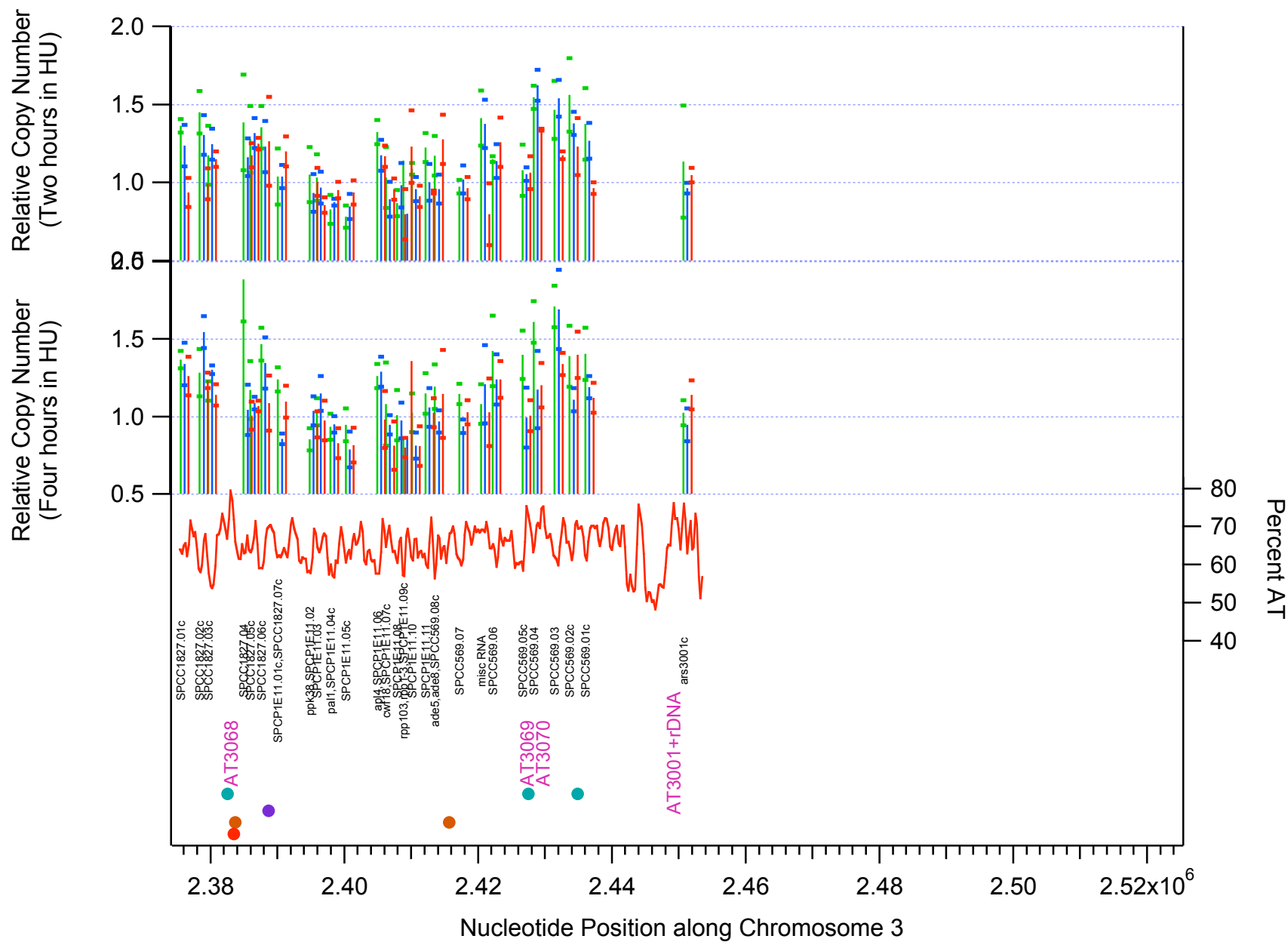

Supplement: Additional file 3 — Graphs of microarray measurements of copy number changes throughout chromosome 3. Similar to additional file 1, but for chromosome 3 [file 1471-2199-8-112-S3.pdf]
