# Supplementary material for: Checkpoint independence of most DNA replication origins in fission yeast
Source: BMC Mol Biol. 2007 Dec 19;8:112. doi: 10.1186/1471-2199-8-112 (PMC2235891; doi:10.1186/1471-2199-8-112)
Supplement: Additional file 11 — Microarray signals at two AT islands that do not appear to serve as replication origins according to previous 2D gel electrophoretic studies. Discussion of apparent disagreement between 2D gels and microarrays at AT1156 and AT2103 [file 1471-2199-8-112-S11.pdf]

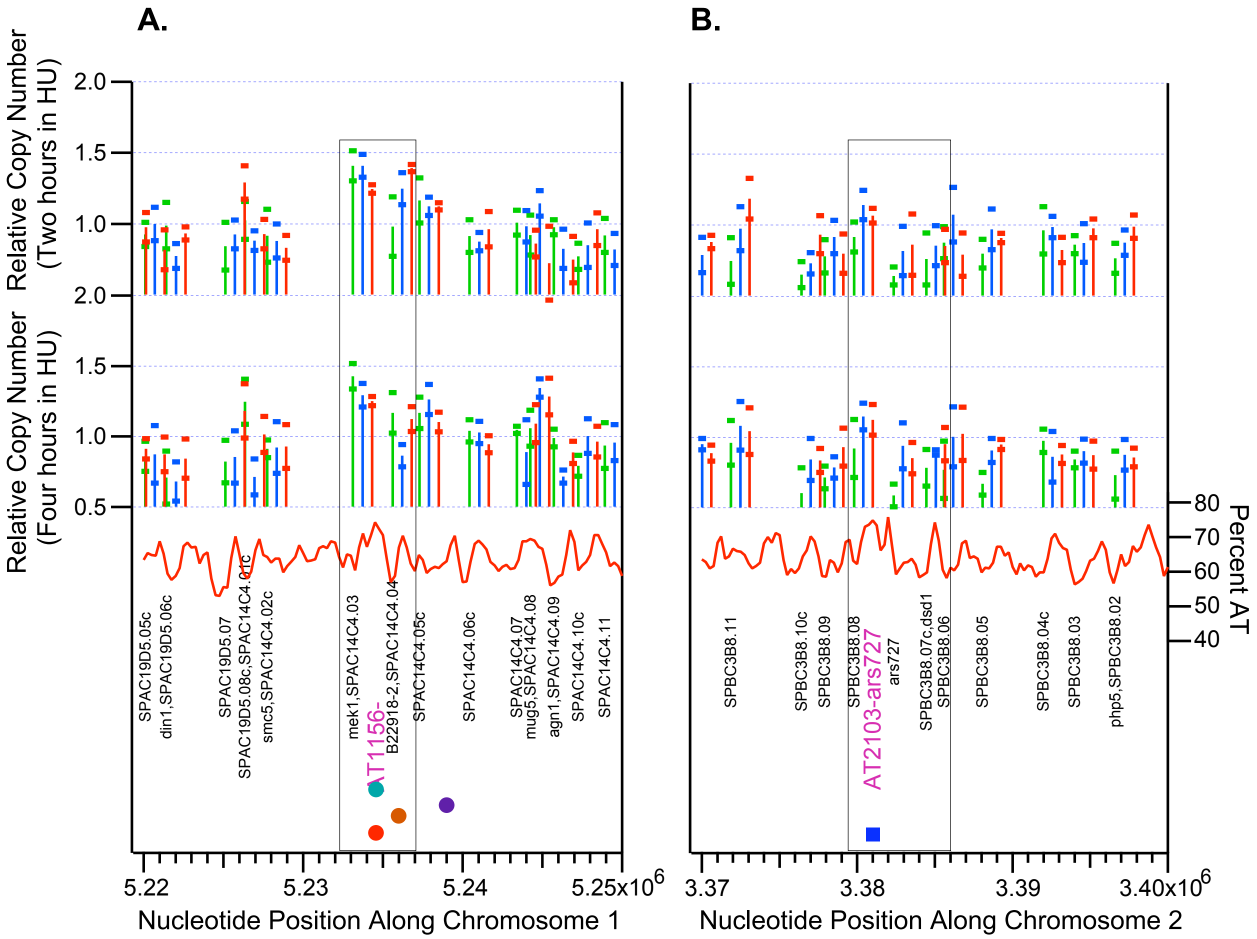

Explanatory text:

AT1156 is an apparent exception to the general correspondence between detection of bubble arcs by 2D gel electrophoresis and replication above the detection threshold (relative copy number of 1.1) in our microarray studies. In this case, the AT island scored as "medium" in our microarray experiment (additional files 10-11) but did not display a bubble arc when tested by 2D gel electrophoresis of unsynchronized cells [1]. Panel A shows the microarray results for the region around AT1156. The two probes immediately flanking AT1156 are contained in the box. The evidence for AT1156 being a functional origin seems compelling. The probe on its left scored above threshold at both the 2-hr and 4-hr time points in all three tested strains, and AT1156 is within 6 kb of putative origins detected in both wild-type and *cds1*Δ cells by accumulation of ssDNA [2] and in wild-type cells by copy-number measurements [3]. In addition, an active pre-RC was detected by Hayashi *et al.* [4] at the position of AT1156 (Panel A). We suspect that in this case the 2D gel analysis failed for some reason to detect a bubble arc. This sometimes happens as a consequence of shearing during DNA preparation or due to single-strand-specific endonuclease contamination of the restriction enzyme.

AT2103, which is also known as *ars727*, is another apparent exception. Previous 2D gel studies were not able to detect a bubble arc associated with *ars727*, yet this region was classified

as a “very weak” potential origin in our current studies. A closer look at our data (Panel B) shows that classification of *ars727* as “very weak” comes from evidence of replication in checkpoint-mutant cells but not in wild-type cells. Since the 2D gel measurements were done with wild-type—not checkpoint-mutant—cells [5], there is in fact no contradiction between the microarray results and the 2D gel measurements.

Legend:

(A) The box contains the probes flanking AT1156. The probe to the left of AT1156 detected signals that were greater than the threshold of 1.1 from both wild-type and checkpoint-mutant cells at both the 2-hour and 4-hour time points. (B) The box contains the two probes that flank and the single probe more centrally located on AT2103, which is also known as *ars727*. For all three probes, the wild-type (green) signal is below 1.0 at both time points. Since the 2D gels that previously detected no origin activity at *ars727* used DNA from wild-type cells [5], there is no discrepancy between the 2D gel result and the microarray result.

References

1. Segurado M, de Luis A, Antequera F: **Genome-wide distribution of DNA replication origins at A + T-rich islands in Schizosaccharomyces pombe.** *EMBO Reports* 2003, **4:**1048-1053.
2. Feng W, Collingwood D, Boeck ME, Fox LA, Alvino GM, Fangman WL, Raghuraman MK, Brewer BJ: **Genomic mapping of single-stranded DNA in hydroxyurea-challenged yeasts identifies origins of replication.** *Nat Cell Biol* 2006, **8:**148-155.
3. Heichinger C, Penkett CJ, Bähler J, Nurse P: **Genome-wide characterization of fission yeast DNA replication origins.** *EMBO J* 2006, **25:**5171-5179.
4. Hayashi M, Katou Y, Itoh T, Tazumi M, Yamada Y, Takahashi T, Nakagawa T, Shirahige K, Masukata H: **Genome-wide localization of pre-RC sites and identification of replication origins in fission yeast.** *EMBO J* 2007, **26:**1327-1339.
5. Kim SM, Huberman JA: **Regulation of replication timing in fission yeast.** *EMBO J* 2001, **20:**6115-6126.
