# Supplementary material for: Checkpoint independence of most DNA replication origins in fission yeast
Source: BMC Mol Biol. 2007 Dec 19;8:112. doi: 10.1186/1471-2199-8-112 (PMC2235891; doi:10.1186/1471-2199-8-112)
Supplement: Additional file 12 — Microarray analysis of two late-replicating, weak origins. Discussion of the microarray results for ars2-2 and Telomere-Associated Sequences [file 1471-2199-8-112-S12.pdf]

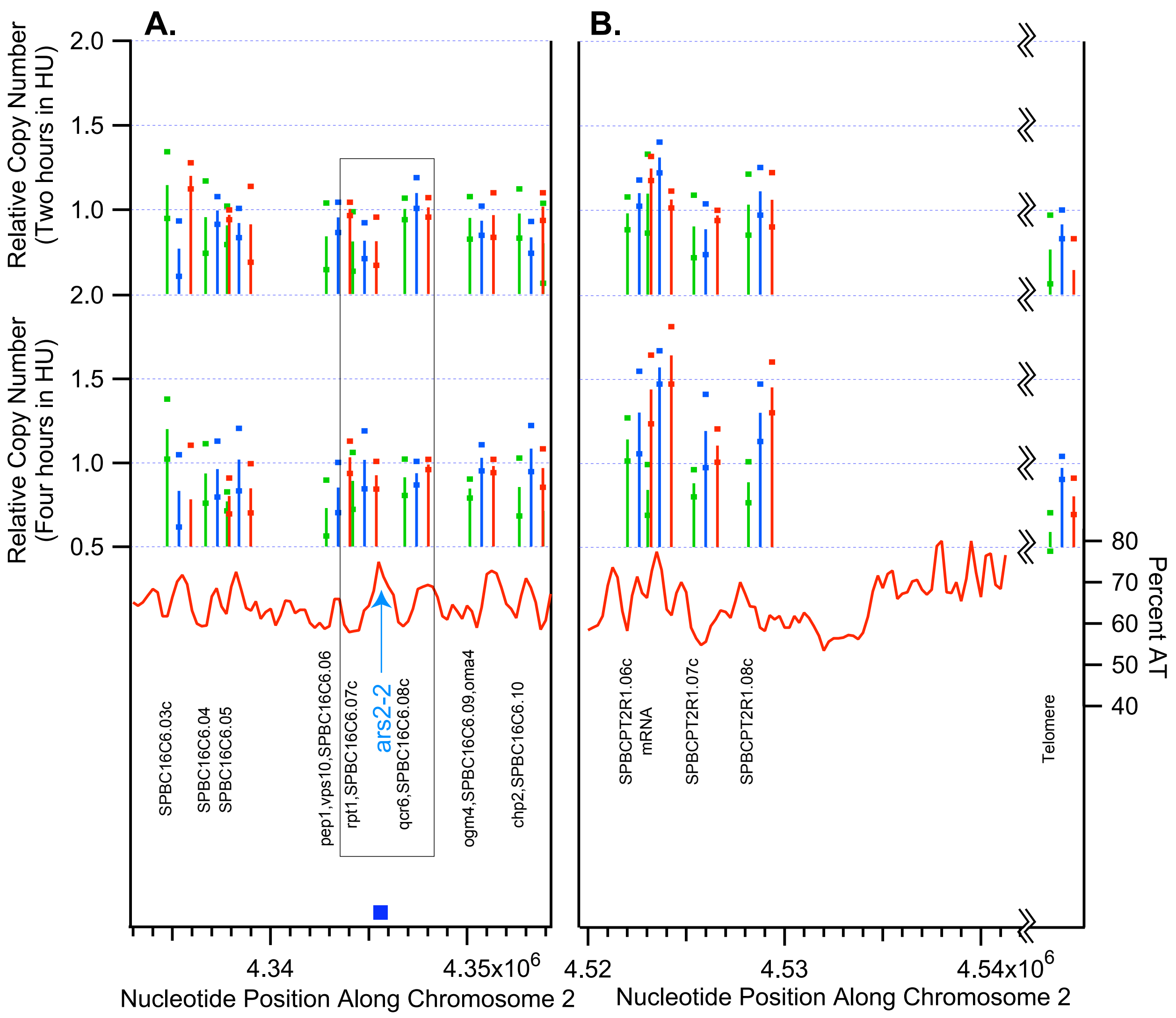

Explanatory text:

The two late-replicating origins that were previously characterized by 2D gel electrophoresis, *ars2-2* and telomeric origins [1], produced signals that were below the threshold for origin function both in our microarray assays (Additional files 10 and 12) and also in the microarray studies of Feng *et al.* [2] and Heichinger *et al.* [3]. However, Hayashi *et al.* [4] detected a pre-RC at the chromosomal position of *ars2-2*. They found that this pre-RC was inactive in wild-type cells but somewhat more active in *cds1*Δ cells [4]. In addition *ars2-2* and the telomeres did not satisfy the criteria for AT islands [5]. By 2D gel electrophoresis, these regions generated weak bubble arcs only in late S phase, only after HU was removed from HU-blocked cells [1]. The 2D gel results indicated that these regions were replicated only slightly if at all in cells blocked in hydroxyurea for 3 hours, consistent with the under-replication (most values of relative copy number less than 1.0) of the probes flanking *ars2-2* (box in panel A) and of the telomere-associated-sequence probe (rightmost probe in panel B), even after 4 hours. However, both regions showed slightly greater replication in checkpoint-mutant than in wild-type cells at four hours after release.

Legend:

The diagrams are explained in the legend to Figure 2 (main text). (A) The box contains the signals from the probes flanking *ars2-2*. The position of *ars2-2* is indicated with blue text and a blue arrow. (B) The right end of chromosome 2, showing the last 4 probes in the sequenced portion of the chromosome plus the telomere-associated sequence (TAS) probe, which detects sequences within 1 kb of the simple-sequence repeats at the telomere. The zig-zag lines indicate a gap in the known nucleotide sequence at the indicated position.

References

1. Kim SM, Huberman JA: **Regulation of replication timing in fission yeast.** *EMBO J* 2001, **20:**6115-6126.
2. Feng W, Collingwood D, Boeck ME, Fox LA, Alvino GM, Fangman WL, Raghuraman MK, Brewer BJ: **Genomic mapping of single-stranded DNA in hydroxyurea-challenged yeasts identifies origins of replication.** *Nat Cell Biol* 2006, **8:**148-155.
3. Heichinger C, Penkett CJ, Bähler J, Nurse P: **Genome-wide characterization of fission yeast DNA replication origins.** *EMBO J* 2006, **25:**5171-5179.
4. Hayashi M, Katou Y, Itoh T, Tazumi M, Yamada Y, Takahashi T, Nakagawa T, Shirahige K, Masukata H: **Genome-wide localization of pre-RC sites and identification of replication origins in fission yeast.** *EMBO J* 2007, **26:**1327-1339.
5. Segurado M, de Luis A, Antequera F: **Genome-wide distribution of DNA replication origins at A + T-rich islands in Schizosaccharomyces pombe.** *EMBO Reports* 2003, **4:**1048-1053.
