# Supplementary material for: Checkpoint independence of most DNA replication origins in fission yeast
Source: BMC Mol Biol. 2007 Dec 19;8:112. doi: 10.1186/1471-2199-8-112 (PMC2235891; doi:10.1186/1471-2199-8-112)
Supplement: Additional file 13 — Extensive correspondence between origins detected by microarrays and molecular combing analyses. Comparison of our microarray results with the molecular combing results of Patel et al. [13] [file 1471-2199-8-112-S13.pdf]

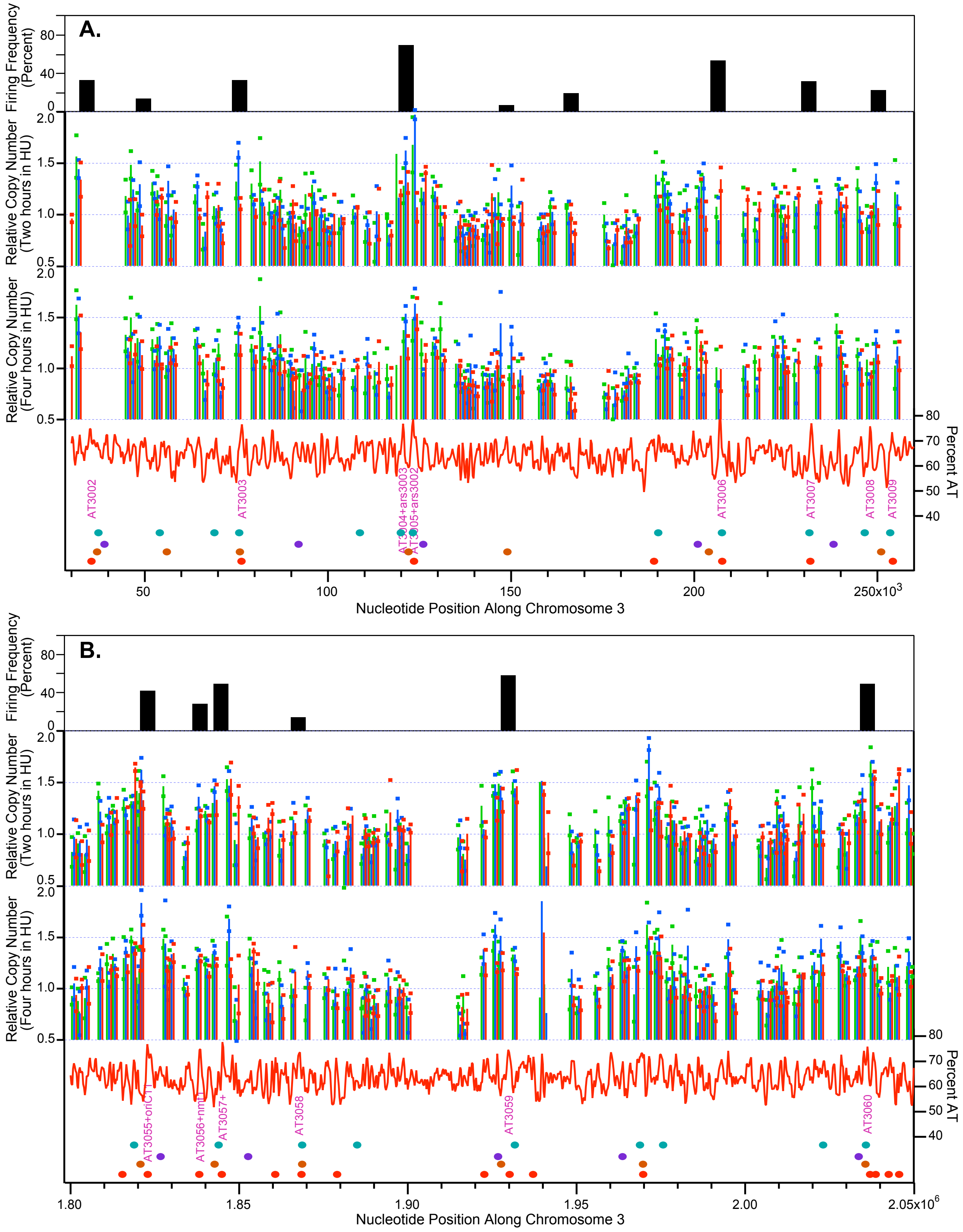

Explanatory text :

Although molecular combing (DNA fiber fluorography) cannot be used to identify specific ori-

gins genome-wide in a single experiment, it can be employed to characterize specific origins within any region (which can be hundreds of kb in length) that can be distinguished by a characteristic fluorescent in-situ hybridization (FISH) pattern of specific probes. Patel *et al.* [1] used this approach to localize and measure the firing frequencies of origins utilized by HU-treated *S. pombe* cells. They analyzed two regions, one near the left end and the other near the right end of chromosome 3. The measurements of Patel *et al.* [1] are displayed in this figure, in comparison with our measurements of relative copy numbers in HU-treated cells in the same two regions, and in comparison with the results from other genome-wide studies [2-5]. For the region from 30–250 kb on chromosome 3 (panel A), Patel *et al.* [1] noted that 82% of initiation events coincided with AT islands. Three other locations (one between AT3002 and AT3003; two others between AT3005 and AT3006) accounted for an additional 13% of signals. The remaining 5% of initiation sites were scattered. The black bars in the top portion of panel A show the frequencies with which Patel *et al.* [1] detected initiations at these locations. For most locations, there is good correspondence with our data and with the other microarray studies. The correspondence is especially strong in the case of AT3004 and AT3005 (which were not resolvable in the molecular combing experiment), where both molecular combing and microarray hybridization reveal unusually high firing efficiency. The locations for which the results of the two methods appear inconsistent are (i) AT3006, which shows high firing efficiency by molecular combing but was below limit in our assays, and (ii) the region close to the left of AT3006 (coordinates 190–205 kb) which is replicated according to the microarray assays but not according to the molecular combing data (panel A). These locations were relatively far from the FISH probes used for molecular combing studies. Therefore, due to technical limitations, Patel *et al.* [1] may have attributed initiation events in the 190-205 kb region to initiation at AT3006 (N. Rhind, personal communication).

In the case of panel B, Patel *et al.* [1] reported initiations only in AT islands. For these locations, their results are in excellent agreement with the microarray measurements. All of the microarray analyses also detected significant replication in the interval 1.96–1.98 Mb, which is far from an AT island. Patel *et al.* also noticed initiation in this region but did not report it, since it was not located at an AT island (N. Rhind, personal communication). Therefore this interval appears to contain replication origins active in HU-treated cells. The general correspondence between the molecular combing results and the microarray measurements lends additional support to the validity of the microarray measurements.

Legend:

Here we have appropriately scaled the frequencies of origin firings at two locations on chromosome 3, as determined by Patel *et al.* [1], and displayed them alongside our corresponding microarray results at the same horizontal scale. We also show the positions of AT islands [2] as well as origins [3, 4] and pre-RCs [5] identified in other microarray studies. The symbols employed here are the same as in Fig. 2. (A) a region near the left end of chromosome 3, containing the

frequently studied origins named *ars3002* and *ars3003*. (B) a region near the right end of chromosome 3, containing the *nmt1* gene.

References

1. Patel PK, Arcangioli B, Baker SP, Bensimon A, Rhind N: **DNA replication origins fire stochastically in fission yeast.** *Mol Biol Cell* 2006, **17:**308-316.
2. Segurado M, de Luis A, Antequera F: **Genome-wide distribution of DNA replication origins at A + T-rich islands in Schizosaccharomyces pombe.** *EMBO Reports* 2003, **4:**1048-1053.
3. Feng W, Collingwood D, Boeck ME, Fox LA, Alvino GM, Fangman WL, Raghuraman MK, Brewer BJ: **Genomic mapping of single-stranded DNA in hydroxyurea-challenged yeasts identifies origins of replication.** *Nat Cell Biol* 2006, **8:**148-155.
4. Heichinger C, Penkett CJ, Bähler J, Nurse P: **Genome-wide characterization of fission yeast DNA replication origins.** *EMBO J* 2006, **25:**5171-5179.
5. Hayashi M, Katou Y, Itoh T, Tazumi M, Yamada Y, Takahashi T, Nakagawa T, Shirahige K, Masukata H: **Genome-wide localization of pre-RC sites and identification of replication origins in fission yeast.** *EMBO J* 2007, **26:**1327-1339.
