# Supplementary material for: Checkpoint independence of most DNA replication origins in fission yeast
Source: BMC Mol Biol. 2007 Dec 19;8:112. doi: 10.1186/1471-2199-8-112 (PMC2235891; doi:10.1186/1471-2199-8-112)
Supplement: Additional file 14 — Examples of checkpoint-restrained and checkpoint-dependent origins. Graphs of microarray results for a checkpoint-restrained and a checkpoint-dependent origin [file 1471-2199-8-112-S14.pdf]

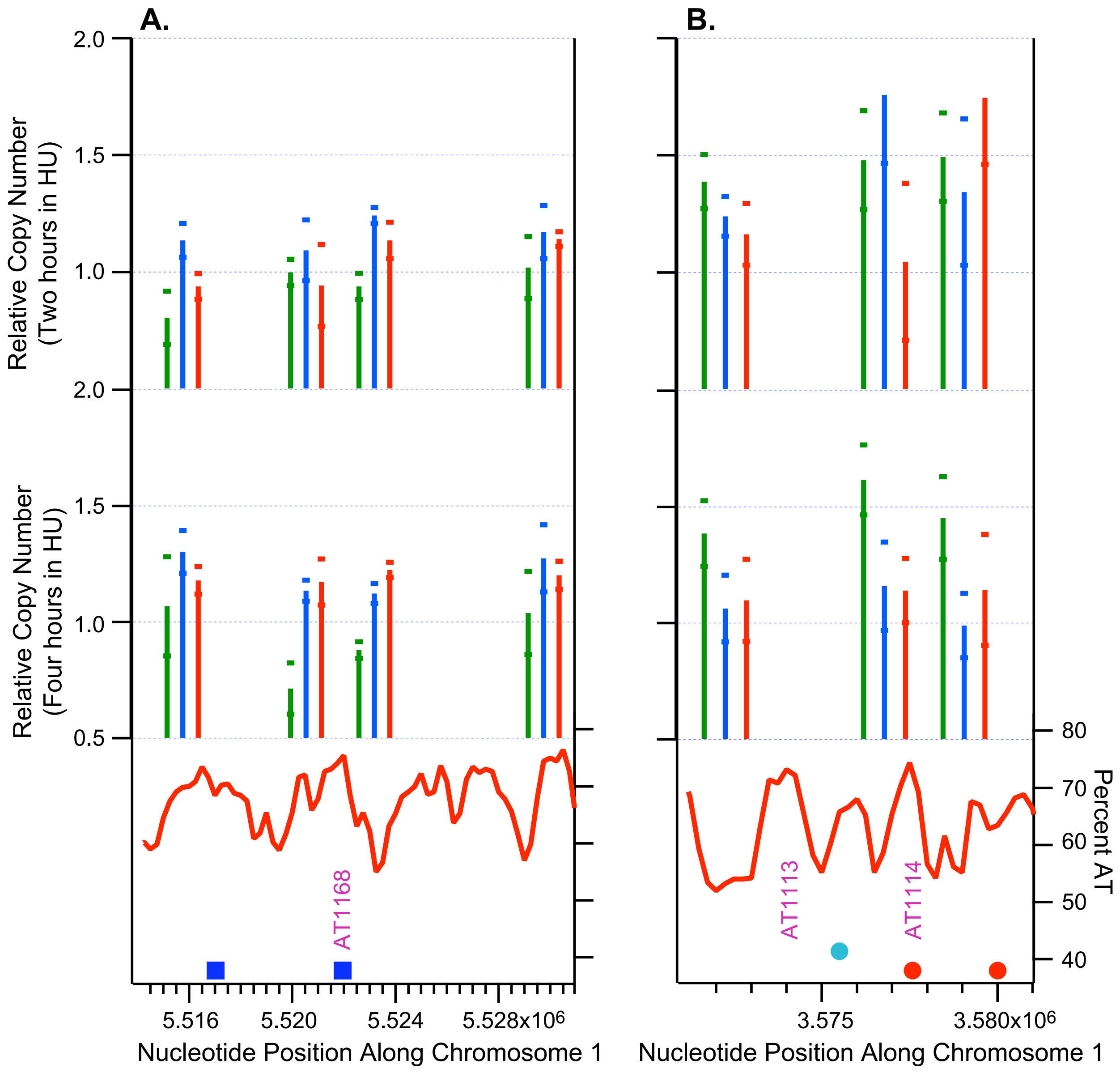


The symbols in this figure are explained in the legend to Fig. 2. (A) An example of a checkpoint-restrained origin. In this case (AT1168), the origin is near the right end of chromosome 1. Both AT1168 (along with its pre-RC) and the pre-RC to the left of AT1168 scored as “very weak” in our classification, but only AT1168 satisfied our criteria for a checkpoint-restrained origin. (B) An example of checkpoint-dependent origins, in the left arm of chromosome 1 but near the centromere. In this case, the two AT islands were classified as both “strong” and “checkpoint-dependent”. The pre-RC to the right of AT114 was classified as “medium” and checkpoint-independent.
